# Outcome of Community-Based Early Intervention and Rehabilitation for Children with Cerebral Palsy in Rural Bangladesh: A Quasi-Experimental Study

**DOI:** 10.3390/brainsci11091189

**Published:** 2021-09-10

**Authors:** Tasneem Karim, Mohammad Muhit, Israt Jahan, Claire Galea, Catherine Morgan, Hayley Smithers-Sheedy, Nadia Badawi, Gulam Khandaker

**Affiliations:** 1 CSF Global, Dhaka 1213, Bangladesh; mmuhit@hotmail.com (M.M.); israt.jahan@cqumail.com (I.J.); 2 Asian Institute of Disability and Development (AIDD), University of South Asia, Dhaka 1213, Bangladesh; 3 Specialty of Child and Adolescent Health, Sydney Medical School, University of Sydney, Sydney, NSW 2145, Australia; cmorgan@cerebralpalsy.org.au (C.M.); HSmithersSheedy@cerebralpalsy.org.au (H.S.-S.); nadia.badawi@health.nsw.gov.au (N.B.); gulam.khandaker@health.nsw.gov.au (G.K.); 4 School of Health, Medical and Applied Sciences, Central Queensland University, Norman Gardens, QLD 4701, Australia; 5 Cerebral Palsy Alliance Research Institute, University of Sydney, Sydney, NSW 2050, Australia; cgalea@cerebralpalsy.org.au; 6 Central Queensland Public Health Unit, Central Queensland Hospital and Health Service, Queensland Government, Brisbane, QLD 4700, Australia

**Keywords:** cerebral palsy, children, early intervention, community-based, low- and middle-income country, Bangladesh

## Abstract

We evaluated the outcome of a community-based early intervention and habilitation for children with cerebral palsy (CP) in Bangladesh. Children registered on the Bangladesh CP Register (BCPR) were recruited in two groups for this study: Group A received a comprehensive six-month long community-based caregiver-led intervention program at the “Shishu Shorgo” (Bengali title, which translates to ‘Children’s Heaven’) Early Intervention and Rehabilitation Centres developed to support participants from the BCPR. Group B received standard care. A quasi-experimental study was conducted. Data were obtained at baseline, at the end of the program (i.e., 6 months), and at a 12-month follow-up. Outcome measures for children included gross motor functional measure (GMFM-66), Communication Function Classification System (CFCS), and Viking Speech Scale (VSS) and, for adult caregivers, the depression, anxiety, and stress scale (DASS 21). Between October 2016 and March 2017, 156 children with CP were recruited (77 in Group A and 79 in Group B). The total score of GMFM-66, CFCS level, and VSS level significantly improved statistically in Group A (*p <* 0.05 for all) and deteriorated in Group B (*p <* 0.001, *p =* 0.095, *p =* 0.232). The intervention showed promising outcomes particularly for children with CP under five years of age. There is a need for caregiver-led community-based programs for children with CP in LMICs.

## 1. Introduction

Cerebral palsy (CP) is the most common physical disability of childhood. The most recent and widely used definition of CP is “a group of permanent disorders of the development of movement and posture, causing activity limitation, that are attributed to non-progressive disturbances that occurred in the developing fetal or infant brain. The motor disorders of cerebral palsy are often accompanied by disturbances of sensation, perception, cognition, communication, and behaviour; by epilepsy, and by secondary musculoskeletal problems” [1]. While knowledge of the antecedents and preventive strategies for CP have advanced considerably over time, our ability to determine the prognosis of CP in terms of type and severity of everyday functioning, particularly in low resource settings, remains poorly understood [2,3,4]. Severity of brain lesions, age at the time of diagnosis, and first access to evidence-based interventions are all some of the important predictors of functional outcomes for children with CP, e.g., communication function and gross motor function [5].

Recent studies from low- and middle-income countries (LMICs) report a greater burden and severity of CP as well as associated impairments [6,7,8]. Children with CP reach 90% of their gross motor potential within the first five years of life, and even earlier for those with severe CP [9,10,11]. Early intervention is thus crucial for optimizing both motor and functional outcomes of children with CP [12]. Owing to the heterogeneity of CP in terms of etiology, brain injury, severity of impairments, and co-occurring conditions, children with CP have diverse needs that call for a comprehensive intervention program. These programs also need to address a range of medical, social, and cultural barriers prevalent in rural and remote communities, especially in LMICs [13]. A recent systematic review highlights the lack of evidence on efficacy for the majority of the interventions in use and the dire need for further research to address the existing research–practice gaps [14]. This is particularly true in LMICs such as Bangladesh where children with CP and their families are faced with numerous barriers in addition to those still prevailing in high income settings [15].

Barriers to intervention for children with CP include (a) delayed diagnosis of CP beyond the critical window of neuroplasticity; (b) poor or no access to evidence-based early intervention; (c) inadequate state funded support and initiatives further propagating the disability and poverty cycle and widening the prevailing inequities; (d) inability to afford services; (e) poor accessibility owing to lack of disability inclusive infrastructure and public transport systems; (f) health workforce crisis particularly of allied health workers required to support children with CP; and (g) prevailing misconceptions, stigma, and social exclusion. It is imperative that these factors are considered in the development of interventions with enhanced relevance for children with CP and their families in low-resource settings [6,13,15,16,17,18].

In this study, we aimed to evaluate the outcomes of an existing program for children with CP and their primary caregivers in a rural subdistrict of Bangladesh.

## 2. Materials and Methods

### 2.1. Study Design, Setting, and Participants

This was a pragmatic design quasi-experimental study conducted in a Northern sub-district of Bangladesh. The study participants included a subset of the BCPR study population. The BCPR is an ongoing surveillance of children (0–18 years), which includes children with CP from rural communities. The BCPR uses the “key informant method (KIM)” for identification of children with CP from the community. The KIM involves identification of children with suspected CP from the community by trained local volunteers (i.e., key informants (KIs)), followed by detail neurodevelopmental assessment by a multidisciplinary team including a physician and physiotherapist for clinical confirmation of diagnosis of CP and data collection. The detailed study protocol and findings have been described in previous publications [6,19].

The rehabilitation needs of the children are assessed and documented as part of the BCPR study. The CSF “Shishu Shorgo” Rehabilitation and Early Intervention Centre’s services were offered to all children with CP who had an identified need for therapy between October 2016 and March 2017. However, not all families were able to access these services to personal or family circumstances.

The CSF “Shishu Shorgo” Early Intervention and Rehabilitation Centres offer a six-month program consisting of intensive episodes of care for children with CP with two intakes per year. Children enrolled at the February and August 2016 intake (referred to throughout this manuscript as Group A) were recruited for this study. Additionally, data were collected on another group (to be identified throughout this manuscript as Group B) of BCPR registrants who were not able to participate in the program. This group received standard care only, which consisted of basic advice provided by the BCPR team including a physician and physiotherapist at the time of registration into the BCPR. Primary caregivers of all the children were also interviewed for assessment of their emotional state. All assessments were conducted as part of the services provided at the CSF “Shishu Shorgo” Early Intervention and Rehabilitation Centres for Group A. Assessments for Group B were conducted during home visits by an assistant physiotherapist trained in study procedures.

We collected outcome data on both groups during three time points; at baseline (prior to enrolment into the program), at the end of the program (i.e., 6 months), and at a follow-up at 12 months (endline).

### 2.2. Intervention Received by Group A

The program at CSF “Shishu Shorgo” serves the local community as the backbone for intervention for children with CP. It is a family-centered program largely run by primary caregivers along with two community therapists (CTs) in each center. The CTs are local community members who receive 15 days of structured training from a qualified physiotherapist on the provision of family centered services for children with disability and at least three months of supervised practice in a community rehabilitation and early intervention centre before they can lead a group under the supervision of the training physiotherapist.

A manual (See Supplementary Material S1: Transition Program Manual) was developed to assist the CTs to run the early intervention program, which aims to optimize neurocognitive outcomes among children with CP in rural communities. It also promotes participation of the children with CP in their family, school, and the community.

Twenty children were enrolled in each intake per centre, a morning group and afternoon group each with ten children. The children at CSF “Shishu Shorgo” undergo assessment and individual goal setting by the primary caregivers and the CT at enrollment. They attend three hourly sessions a day, five days a week at CSF “Shishu Shorgo” Early Intervention and Rehabilitation Centers. The early intervention program is comprised of the following key components:

a. Group therapy: The group therapy focuses on the development of the following skills: activities of daily living (toileting, dressing, eating), language and communication, movement, cognition, and social skills.

b. Community follow-up: Throughout the six-month long program, the CTs provide community follow up for each child and family. The goals of this community follow-up are (i) provision of strategies and assistive devices to assist the child at home with activities of daily living (washing, using the toilet, eating, and dressing); (ii) supporting the child’s local school to enable their admission to school and participation in school activities; (iii) increasing awareness about disability and the child’s abilities and rights to facilitate the child’s family and community develop support networks and increase opportunities for the child to participate in the community; and (iv) supporting the child to develop a meaningful vocation in their family and community, particularly for those children unable to attend school.

c. Primary caregiver training, peer support, and education: The children attend the sessions with at least one primary caregiver who is engaged in all elements of the program to develop skills on day-to-day care of the children with CP using the CSF “Shishu Shorgo” program manual. Parent support and education is provided through involving the child’s primary caregivers in all elements of the program. The CTs involve the parent in group therapy and community follow-up by providing family-centred care, keeping the primary caregiver informed about the child’s progress, and providing recommendations and empowering primary caregivers to advocate for and facilitate their child’s participation in their home, community, school, and vocation. Through this program, the primary caregivers form support networks/peer group with other caregivers of children with disabilities.

### 2.3. Intervention Received by Group B

Group B did not opt for the intervention at the CSF “Shishu Shorgo” Early Intervention and Rehabilitation Centres and received standard care, i.e., no structured intervention; they only received advice provided by the multidisciplinary team at the assessment camps during recruitment into the BCPR.

### 2.4. Outcome Measures Assessed for Both Groups A and B

The children and their primary caregivers were assessed at baseline at the time of enrollment, after a six-month period to measure the immediate outcome of the intervention, and after twelve months to evaluate long-term effects of the intervention. A qualified physiotherapist was trained by the study investigators on the assessments. The following instruments were used for assessment of the children and their primary caregivers:

Motor function: Gross Motor Function Measure (GMFM-66) and Gross Motor Function Classification System (GMFCS) were used to evaluate changes in motor function of the children with CP [20,21]. The GMFM is a criterion referenced assessment designed and validated to measure change in gross motor function over time in children with CP. GMFCS evaluates movement skills such as sitting, walking, and use of mobility devices and categorizes gross motor function into five levels. We reported and analyzed GMFCS as ordinal and GMFM scores as continuous variables. GMFM-66 data were entered, stored, and analyzed using the Gross Motor Ability Estimator (GMAE-2) Scoring Software for the GMFM [22].

Communication: Communication Function Classification System (CFCS) was used to classify the everyday communication of the children with CP into one of five levels according to effectiveness of communication, which considers means of communication including speech, gesture, facial expression, and augmentative and alternative communication [23]. A child classified at Level I is a more effective communicator than a child classified at Level V. The CFCS level was assessed for children aged 2 years and above. We reported and analyzed CFCS as an ordinal variable.

Speech: Viking Speech Scale (VSS) is used to classify children’s speech production, The VSS level was assessed for children aged 4 years and above. We reported and analyzed the VSS as an ordinal variable [24].

Emotional state of primary caregivers: Depression Anxiety Stress Scale (DASS-21), a 21-item self-report instrument, was used to measure depression, anxiety, and stress of the primary caregivers of the children with CP [25]. The primary caregivers were asked to use four-point severity/frequency scales to rate the extent to which they have experienced each state over the past week. DASS-21 scores were summed into ‘depression’, ‘anxiety’, and ‘stress’ scale and categorized as ‘normal’, ‘mild’, ‘moderate’, ‘severe’, or ‘extremely severe’ as per instrument protocol for analysis and interpretation. We reported and analyzed depression, anxiety, and stress scores as continuous variable for Friedman analysis.

### 2.5. Ethical Considerations

Ethics approval for this study was obtained from the Bangladesh Medical Research Council (BMRC) (Ref: BMRC/NREC/2016-2019/469) and Asian Institute of Disability and Development (AIDD) (southasia-irb- 2016-1-07). Informed written consent was obtained from the primary caregivers of the children.

### 2.6. Statistical Analysis

Descriptive methods were used to summarize the cohort. Normality was checked using Shapiro–Wilk and visual inspection of the box plots. Normality assumptions failed; therefore, the non-parametric Friedman test with post hoc pairwise comparisons were used and reported using median and interquartile range (IQR). However, data were also reported using the mean (SD) for clinical interpretation. There was a statistically significant difference between ages for Group A and Group B; therefore, we could not run any regression analysis using age as a covariate. Analysis was carried out using SPSS version 24 (IBM Corporation, Chicago, Illinois, USA). A *p*-value of <0.05 was considered statistically significant to report any difference in outcome measure between the two groups or to illustrate relationship between different variables. Subgroup analysis was conducted to gain insights into each group separately.

## 3. Results

**Figure 1:**
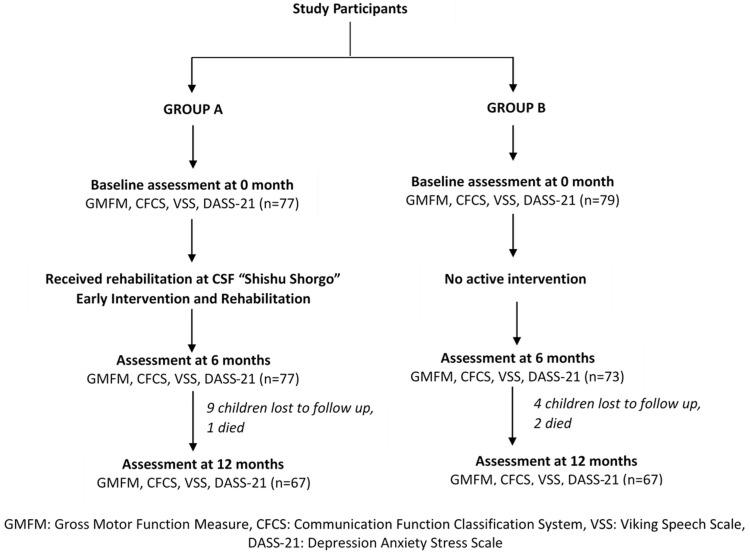
CONSORT Flow Diagram.

Between October 2016 and March 2017, 156 children with CP were recruited to the study. Seventy-seven children with CP enrolled in the CSF “Shishu Shorgo” formed Group A and 79 children with CP who received standard care (i.e., basic education with no structured intervention) formed Group B (Figure 1). Mean (SD) age at baseline (i.e., 0 month), Group A versus Group B: 4.3 (2.9) versus 11.1 (4.0), *p*-value < 0.001. Post hoc analysis to investigate the age differences were performed and comparison within groups were also conducted where groups were not matched by age and sample size.

The demographic characteristics of the study participants are summarized in Table 1. The male/female ratio in Group A and Group B was 1.4:1 and 1.5:1, respectively (*p =* 0.448). Both groups had similar sociodemographic characteristics; no significant (*p* > 0.05) difference was observed between the groups in terms of type of accommodation, source of drinking water, sanitation, paternal education, and occupation. However, the median monthly family income and maternal educational level were significantly lower in Group B compared with Group A (*p =* 0.005 and *p =* 0.010, respectively). These notable differences between the two groups and their impact on the outcome measures are outlined in the subsequent sections.

### 3.1. Group A

#### 3.1.1. GMFM Score

The median GMFM total score significantly improved between baseline and endline (median [IQR]: 34.8 [16.0, 46.5] vs. 44.6 [30.5, 52.3]; *p* < 0.001) in Group A. A similar significant improvement was observed for children aged less than five years (median [IQR] between baseline vs. endline: 30.2 [14.8, 46.0] vs. 46.1 [27.2, 52.4]; *p* < 0.001); however, for children aged five years and more, the scores remained unchanged between baseline and 1 (median [IQR]: 43.6 [17.8, 51.0] vs. 43.6 [29.0, 52.6]; *p =* 0.093) (Table 2 and Table 3).

#### 3.1.2. GMFCS Level

Overall, 42.16% children had GMFCS level V at baseline, which reduced to 24.76% at 6 months and again slightly increased to 28.4% at 12 months (*p <* 0.001) (Table 2). When disaggregated by age, the GMFCS level significantly improved among children aged less than five years between 0 and 6 months (*p <* 0.001), and then slightly deteriorated between 6 and 12 months (*p <* 0.001). Whereas for children aged five years and above in this group, the GMFCS level remained similar between 0 and 6 months (*p =* 0.285) and significantly deteriorated between 6 and 12 months (*p =* 0.792) (Table 3).

#### 3.1.3. CFCS Level

Overall, the CFCS level significantly improved between baseline and 12 months of the study (*p =* 0.003) (Table 2). Among the children aged less than five years, the proportion of children with CFCS level V reduced from 35.3% (*n =* 18) to 24.5% (*n =* 12), whereas the CFCS level I increased from 25.5% (*n =* 13) to 26.5% (*n =* 13) between baseline and 12 months (*p =* 0.045). A similar less pronounced change was observed among the children aged five years and above (*p =* 0.705) (Table 3).

#### 3.1.4. VSS Level

Overall, a significant improvement in VSS level was observed (*p <* 0.001) (Table 2). Upon disaggregation by age, the children aged less than five years showed significant improvement between baseline and 6 months (*p =* 0.041), 6 and 12 months (*p <* 0.001), and baseline and 12 months (*p <* 0.001). Whereas the children aged five years and above showed a significant improvement between baseline and 6 months (*p =* 0.014), followed by deterioration between 6 and 12 months (*p =* 0.070); there was an overall significant change across all three time points (*p =* 0.023) (Table 3).

#### 3.1.5. Primary Caregiver DASS 21: Depression

At baseline, overall, 37.7% (*n =* 29) of the caregivers’ depression scores were in the mild to extremely severe range. Although this percentage decreased to 19.7% (*n =* 15) at 6 months of the study, we observed a marked increase (50.8%, *n =* 34) in the proportion of caregivers with scores in mild to extremely severe range for the depression subscale at 12 months (*p <* 0.001) (Table 2). These scores significantly improved among caregivers of children aged less than five years between baseline and 6 months (*p <* 0.001), followed by a significant increase between 6 and 12 months (*p <* 0.001). However, the overall change between baseline and 12 months was not significant. A similar pattern was observed for scores of caregivers of children aged five years and above (Table 3).

#### 3.1.6. Primary Caregiver DASS 21: Anxiety

Overall, 41.6% (*n =* 32) of caregivers had anxiety scores in the mild to extremely severe range at baseline, which decreased to 26.3% (*n =* 20) at 6 months followed by a sharp rise to 94.0% (*n =* 63) at 12 months (*p <* 0.001) (Table 2). Among caregivers of children aged less than five years, the median (IQR) anxiety score slightly improved between baseline and 6 months (*p =* 0.046), but it increased significantly between 6 and 12 months (*p <* 0.001). A similar observation was made among caregivers of children aged five years and above (Table 3).

#### 3.1.7. Primary Caregiver DASS 21: Stress

Overall, a substantial improvement in caregiver stress subscale scores was observed between baseline and 6 months; however, this was followed by a deterioration between 6 months and 12 months (*p <* 0.001) (Table 2). The median (IQR) score on the stress subscale reduced significantly between baseline and 6 months among the caregivers of children aged less than five years (*p <* 0.001), and caregivers of children aged above five years (*p =* 0.002). However, the score increased significantly between 6 and 12 months for both groups (Table 3).

### 3.2. Group B

#### 3.2.1. GMFM Score

The GMFM total score significantly deteriorated between baseline and 12 months among children in Group B (median [IQR]: 46.9 [16.0, 59.3] vs. 42.2 [22.7, 54.6]; *p <* 0.001). When disaggregated by age, the median [IQR] GMFM total score deteriorated between baseline and 6 months among both children aged less than five years (36.0 [10.4, 58.6] vs. 33.7 [11.1, 64.1]; *p =* 0.600) and children aged five years and above (48.5 [16.0, 59.9] vs. 47.7 [30.0, 66.7]; *p <* 0.001). The pattern remained unchanged between 6 and 12 months (median [IQR]: 33.7 [11.1, 64.1] vs. 29.8 [10.4, 50.2]; *p =* 0.345 for children aged less than five years and 47.7 [30.0, 66.7] vs. 44.4 [24.0, 55.1]; *p <* 0.001 for children aged five years and above) (Table 2 and Table 3).

#### 3.2.2. GMFCS Level

Overall, significant deteriorations in GMFCS level between baseline and 12 months were also observed for children in Group B (GMFCS level III-V at baseline vs. 12 months: 71.0% vs. 80.6%; *p <* 0.001). However, the changes were not significant for children aged less than five years (*p =* 0.097), but were significant for children aged five years and above (*p =*< 0.001) (Table 2 and Table 3).

#### 3.2.3. CFCS Level

Overall, the CFCS level deteriorated between baseline and 12 months (*p =* 0.095). Upon disaggregation by age, no significant difference was observed in CFCS levels among children aged less than five years. Among children aged five years and above, the CFCS level deteriorated significantly between 6 and 12 months (*p =* 0.015) (Table 2 and Table 3).

#### 3.2.4. VSS Level

Overall, the VSS level slightly improved among children between baseline and 6 months, but then deteriorated between 6 and 12 months; however, this change between these timepoints was not statistically significant (*p =* 0.232). When disaggregated by age, a slight improvement in VSS level was observed for children aged less than five years between baseline and 6 months (*p =* 0.180), 6 and 12 months (*p =* 1.00), and baseline and 12 months (*p =* 0.144). Children aged 5 years and more showed an improvement in VSS level between baseline and 6 months (*p =* 0.011); however, it slightly deteriorated between 6 and 12 months (*p =* 0.197) (Table 2 and Table 3).

#### 3.2.5. Primary Caregiver DASS 21: Depression

The proportion of caregivers with elevated depression sub-scale scores gradually increased across the three timepoints (*p <* 0.001). The depression subscale scores decreased between baseline and 6 months (*p =* 0.197) and increased between 6 and 12 months (*p =* 0.042) among caregivers of children aged less than five years. However, the median score remained unchanged between baseline and 6 months for caregivers of children aged five years and above (*p =* 0.725), but significantly increased between 6 and 12 months (*p <* 0.001) (Table 2 and Table 3).

#### 3.2.6. Primary Caregiver DASS 21: Anxiety

The proportion of caregivers with scores in mild to extremely severe range gradually increased between baseline, 6, and 12 months (*p <* 0.001) among primary caregivers of children. When disaggregated by age of children, the anxiety score increased between baseline and 6 months (*p =* 0.144) and between 6 and 12 months (*p =* 0.173) among caregivers of children aged less than five years. However, among caregivers of children aged five years and above, the median anxiety score increased between baseline and 6 months (*p <* 0.001) and 6 and 12 months (*p <* 0.001) (Table 2 and Table 3).

#### 3.2.7. Primary Caregiver DASS 21: Stress

The proportion of caregivers with scores in the mild to extremely severe range of stress decreased between baseline and 6 months, but increased between 6 and 12 months (*p <* 0.001) among the primary caregivers. The median stress score decreased between baseline and 6 months for both subgroups (*p =* 0.206 for children aged less than five years and *p <* 0.001 for children aged five years and above). This was followed by an increase between 6 and 12 months in both age groups (*p =* 0.066 and *p <* 0.001, respectively) (Table 2 and Table 3).

### 3.3. Mortality

Three children died during the study period: one from Group A (male, age: 7.2 years) and two from group B (female, age: 10.2 years and male, age: 7.5 years). All three children had severe motor (GMFCS Level V), communication (CFCS level V), and speech impairments (VSS level IV). All three families were living below the poverty line and residing in mud houses. All three mothers were unemployed, even though two of them had a higher level of education compared with their husbands who were employed.

## 4. Discussion

To the best of our knowledge, this is one of few studies to systematically examine the outcome of a community-based interventional program for children with CP in Bangladesh and similar economy countries. The intervention in this study incorporated several key components to address some of the barriers to intervention in low-resource settings [6,13,15,16,17,18]. It is scalable and easily replicable in other parts of Bangladesh and similar settings in other LMICs. The intervention is provided free of cost and accompanied by a transportation service. It is delivered by community health workers; therefore, it is sustainable in the absence of highly skilled allied health workers. The intervention concurrently aims to empower the primary caregivers to be able to continue therapy beyond the scope of the study and better equip them for the caregiving role of their child’s lifelong condition. The intervention commences with goal setting for each child with the primary caregivers at the time of enrollment—this is best practice for CP particularly owing to the heterogeneous nature of the condition and it enables individualization of care for optimal outcomes [26,27].

The children who received the intervention showed significant improvement in GMFM total scores in the first six months. This improvement was sustained among children who were aged less than five years. However, although not statistically significant, there was a decline observed among those who were aged five years and above. In contrast, among those who did not receive intervention, a decline in the median GMFM scores was observed across the three timepoints. This decline was statistically significant for children aged five years or more.

This finding in Group A contrasts to some extent with what has been previously published on motor function trajectories of children with CP; with the exception of GMFCS V, children with CP typically continue to increase in GMFM scores until about 5–7 years and then reach a plateau [28,29]. It is later in adolescence that a decline in motor function for those GMFCS III-V occurs [28,29]. However, in our study, an earlier deterioration was observed. This potentially reflects fundamental differences in interventions available to our study cohort and the children in Canada whose data were used for the development of the reference curves [30]. In low-resource settings, the burden and severity of associated impairments and malnutrition is greater among children with CP, and there are wider gaps in services [8,31,32,33,34]. There is an overall lack of available intervention to manage secondary musculoskeletal impairments (i.e., hip surveillance programs and use of botox for spasticity management), lack of practice environment for motor skills, limited access to the assistive devices, and poor wheelchair accessibility [6,35,36,37,38]. The findings from this study reaffirm the need for cautious interpretation of comparisons between LMICs and high-income settings. Further research to better understand the motor function trajectories among children with CP in LMICs is essential for normative interpretations in low-resource settings.

There was significant improvement in communication and speech among the children who received the intervention. Upon stratification by age, better outcomes were observed among children aged less than five years. This finding reinforces the importance of early intervention before five years of age. Meanwhile, a different pattern was observed among the children who did not receive the intervention in Group B. Those who were under five years of age did not show any significant change in communication, while the children over five years of age deteriorated significantly over time. There was no significant change observed for speech in this group. However, interpretations should be made with caution as the sample size for children under the age of five years in this group was considerably smaller than those above five years of age (i.e., 7, 6, and 6 vs. 71, 67, and 61 at 0, 6, and 12 months, respectively). Currently, there are 0.9 speech and language therapists for every million population in Bangladesh [31]. There is a dire need for increased number of speech and language therapists in countries like Bangladesh to support the inclusion of speech therapy in early intervention programs for children with CP.

Communication impacts several key areas including social participation, education, employment, and quality of life of individuals with CP. In addition to the motor speech impairment, communication difficulties among children with CP can also be due to accompanying intellectual impairment, resulting in complex communication needs [39]. Augmentative and alternative communication (AAC) is vital to support the development of communication skills among children with CP [40]. While there is a range of AAC systems available for children with CP [41], there is a prevailing need for support to implement AAC and to develop feasible AAC systems for children with CP and their communication partners in low-resource settings.

Interventions for children with CP are also strongly linked to caregiver wellbeing [42]. This has been evidenced in studies from LMICs including Bangladesh and Ghana [43,44]. The findings from a study in Zimbabwe have previously also reported that caregivers of children with CP had poor health-related quality of life; high levels of depression, anxiety and stress; and felt overwhelmed by the economic burden and their caregiving role [45]. In our study, there were significant improvements in the DASS scores of the primary caregivers of the children in the intervention group. This potentially reflects the beneficial effects of peer-to-peer support through the formation of caregiver networks within the intervention program at CSF “Shishu Shorgo” Early Intervention and Rehabilitation Centres. Having a child with CP impacts the entire family, particularly mothers who bear the majority of the burden of caregiving. Cultural factors, misconceptions, and stigma around disability add further to the plight of mothers. Furthermore, 86% and 82% of mothers in group A and B were unemployed, respectively. According to World Bank data, the female unemployment rate in Bangladesh is ~6%. This marked difference is most likely owing to their primary role as caregivers.

In recent times, there has been an increasing shift towards greater participation of primary caregivers in therapy for their children [46,47]. Studies have reported improved child and caregiver outcomes through this approach. However, caregiver emotional wellbeing is crucial for them to effectively deliver such interventions [48]. Several studies report that interventions for children with CP significantly improve parental wellbeing [46]. Our findings support the recommendations from a previous study conducted in Bangladesh that highlighted the importance of supporting caregiver mental wellbeing when designing interventions for individuals with CP [43]. Furthermore, interventions including holistic measures for poverty alleviation and improvement of social and economic capital of the families can also potentially yield better functional outcomes for children with CP [49].

Population-based surveillance in Bangladesh has found increased vulnerability to mortality with greater motor severity and more severe associated impairments [50]. This is consistent with the findings in our study, where all three children who died during the study period had severe gross motor, communication, and speech impairment. Moreover, all three families were living in impoverished conditions and their family income was below the poverty line. These findings highlight the relationship between health outcomes and social determinants of health and the importance of addressing the prevailing inequities to reduce preventable deaths among children with CP.

### Study Limitations

Our study demonstrated short-term improvements in child and primary caregiver outcomes among those who received the intervention, with greater improvements observed among children under five years of age. However, as the groups were not matched for age (i.e., child’s age at the time on enrolment into the “Shishu Shorgo” program), some of the findings need to be interpreted with caution including outcomes for those children who were above five years of age and did not show marked changes, and even deteriorated in some areas.

One of the major limitations of the study was that Group A and Group B were significantly different in terms of age (mean age: 3.7 vs. 9.6 years), which inhibited the statistical analysis; there was also variation in the baseline status of their motor and communication functions. These factors may have collectively influenced the study findings. There was also bias introduced due to the method of recruitment of study participants. The intervention was offered to all children with CP in need of therapy identified through the BCPR during the study period. Those who were able to take up the provided intervention formed Group A, whereas the families that were unable to take this opportunity owing to personal or family circumstances formed Group B. Therefore, interpretation of the differences in outcome between the two groups should be made with caution considering that there are several differences between the children who received the intervention and those who did not including their age, severity of impairments, and some sociodemographic characteristics of importance (i.e., monthly family income and maternal education) at baseline. These notable differences between the two groups and their potential confounding effect on the study outcome measures limited further analysis; only subgroup analysis has been conducted to generate insights into each group separately.

Despite these differences, both groups showed short-term improvement in the study outcomes. The improvement among the children who did not receive the intervention (i.e., Group B) could possibly be because of the primary caregiver education provided at the time of registration in the BCPR; the registered children are also ensured access to assistive devices and have access to weekly physiotherapy clinics in the surveillance area. It could additionally be because of attrition bias as some of the more severe cases were lost to follow up or died during the study period. Moreover, the heterogeneous nature of CP poses a challenge in the application and evaluation of interventions. Further exploration through large-scale randomized controlled trial using the BCPR as the sampling frame to evaluate whether the beneficial effects of the intervention are sustained over long-term is needed to generate more robust population-level evidence.

Despite these limitations, this study was based on a population-based cohort (unlike institutional cohort subject to selection bias) and, therefore, represents the true nature and likely outcomes of community-based intervention programs in LMICs. As a pragmatic quasi experimental study, findings from this study including our reported limitations can inform clinicians and researchers in developing more holistic programs and well-designed experimental studies including randomized controlled trials.

## 5. Conclusions

The outcomes of the intervention in the present study showed promise, particularly for young children with CP under five years of age. There is a need for such caregiver-led community-based programs for children with CP in LMICs such as Bangladesh, particularly in rural settings, where services and trained health workers are scarce. This will ensure access to services to one of the most marginalized populations and make optimal use of the limited available resources. This work can potentially underpin the development of a sustainable model of interventions for children with CP in low-resource settings, which can one day be implemented at scale.

## Figures and Tables

**Table 1:** Sociodemographic characteristics of the study participants.

Characteristic	Group A (*n* = 77)	Group B (*n =* 79)	*p*-Value ^b^
**Age**			
Mean (SD)	4.3 (2.9)	11.1 (4.0)	**<0.001 ^c^**
Median [IQR]	3.4 (2.4, 5.4)	11.6 (7.9, 14.1)	**<0.001 ^d^**
**Sex**			
Male	45 (58.4)	48 (60.8)	0.448 ^e^
Female	32 (41.6)	31 (39.2)	
**Monthly family income (BDT)~[USD] ^a,f^**			
Mean (SD)	9870.5 (6390.7) [~84.8 (75.3)]	8256.4 (6289.9) ~97.3 (74.1)	0.119 ^c^
Median [IQR]	8000.0 [6000.0, 10,000.0] ~94.3 [70.7, 117.9]	6000.0 [5500.0, 8250.0] ~70.7 [64.8, 97.2]	**0.005 ^d^**
**Monthly family income in BDT (USD) ^a^**			
Below 10,000 (below ~120)	49 (66.2)	60 (76.9)	0.052
10,000–19,999 (~120–241)	15 (20.3)	15 (19.2)	
20,000–29,999 (~241–361)	9 (12.2)	1 (1.3)	
30,000 and above (~361 and above)	1 (1.4)	2 (2.6)	
**Type of accommodation ^a^**			
Temporary shelter (jhupri)	0 (0.0)	2 (2.6)	0.240
Mud (kutcha) house	55 (74.3)	61 (78.2)	
Semi-permanent (semi-pucca) house	13 (17.6)	13 (16.7)	
Permanent brick (pucca) house	6 (8.1)	2 (2.6)	
**Source of drinking water ^a^**			
Tap water	1 (1.4)	0 (0.0)	0.487
Tube well	73 (98.6)	78 (100.0)	
**Sanitation ^a^**			
No toilet facility	2 (2.7)	1 (1.3)	0.816
Non-sanitary latrine	23 (31.1)	24 (30.8)	
Sanitary latrine	49 (66.2)	53 (67.9)	
**Maternal education ^a^**			
Illiterate	20 (27.0)	40 (51.3)	**0.010**
Primary	26 (35.1)	24 (30.8)	
Secondary	22 (29.7)	11 (14.1)	
Higher secondary and above	6 (8.2)	3 (3.8)	
**Maternal occupation ^a^**			
Agriculture worker	1 (1.4)	2 (2.6)	0.720
Garment worker/weaver/tailor	9 (12.2)	12 (15.4)	
Unemployed	64 (86.5)	64 (82.1)	
**Paternal education ^a^**			
Illiterate	26 (35.1)	40 (51.3)	0.174
Primary	23 (31.1)	19 (24.4)	
Secondary	18 (24.3)	16 (20.5)	
Higher secondary and above	7 (9.5)	3 (3.8)	
**Paternal occupation ^a^**			
Agriculture worker	11 (15.1)	23 (30.3)	0.207
Daily wage earners	10 (13.7)	12 (15.8)	
Business	16 (21.9)	16 (21.1)	
Garment worker/weaver/tailor	24 (32.9)	19 (25.0)	
Others	11 (15.1)	5 (6.6)	
Unemployed	1 (1.4)	1 (1.4)	

^a^ Missing data, ^b^ Fisher’s exact test, ^c^ independent t test (2-tailed), ^d^ Mann-Whitney U test (2-tailed), ^e^ Chi-square test (2-tailed), ^f^ 1USD =81BDT. The statistically significant values are in bold.

**Table 2:** Descriptive findings of the study participants.

	Group A				Group B			
Timepoint	0 Month	6 Months	12 Months	*p*-Value ^a^	0 Month	6 Months	12 Months	*p*-Value ^a^
	*n =* 77	*n =* 77	*n =* 67		*n =* 79	*n =* 73	*n =* 67	
**GMFM Total score**								
Mean (SD)	32.5 (18.6)	42.2 (20.1)	42.3 (18.2)	-	39.3 (22.0)	46.4 (23.5)	41.2 (19.4)	-
Median (IQR)	34.8 [16.0, 46.5]	43.6 [23.6, 54.4]	44.6 [30.5, 52.3]	<0.001	46.9 [16.0, 59.3]	46.9 [26.3, 65.8]	42.2 [22.7, 54.6]	<0.001
**GMFCS, *n* [%]**	76	77	67		79	73	67	
Level I	4 (5.3)	11 (14.3)	5 (7.5)	<0.001	1 (1.3)	7 (9.6)	1 (1.5)	<0.001
Level II	10 (13.2)	14 (18.2)	6 (9.0)		22 (27.8)	20 (27.4)	12 (17.9)	
Level III	14 (18.4)	19 (24.7)	20 (29.9)		19 (24.1)	11 (15.1)	16 (23.9)	
Level IV	16 (21.1)	14 (18.2)	17 (25.4)		7 (8.9)	16 (21.9)	15 (22.4)	
Level V	32 (42.1)	19 (24.7)	19 (28.4)		30 (38.0)	19 (26.0)	23 (34.3)	
**CFCS, *n* [%]**					***n =* 77 ^b^**			
Level I	15 (19.5)	24 (31.2)	16 (23.9)	0.003	20 (26.0)	29 (39.7)	19 (28.4)	0.095
Level II	12 (15.6)	7 (9.1)	8 (11.9)		11 (14.3)	5 (6.8)	6 (9.0)	
Level III	11 (14.3)	13 (16.9)	19 (28.4)		13 (16.9)	9 (12.3)	18 (26.9)	
Level IV	11 (14.3)	12 (15.6)	8 (11.9)		17 (22.1)	12 (16.4)	3 (4.5)	
Level V	23 (29.9)	21 (27.3)	16 (23.9)		16 (20.8)	18 (24.7)	21 (31.3)	
NA [aged ≤ 2 years]	5 (6.5)	0 (0.0)	0 (0.0)		0 (0.0)	0 (0.0)	0 (0.0)	
**VSS, *n* [%]**								
Level I	4 (5.2)	8 (10.4)	13 (19.4)	<0.001	15 (19.0)	28 (38.4)	20 (29.9)	0.232
Level II	6 (7.8)	7 (9.1)	11 (16.4)		13 (16.5)	0 (0.0)	6 (9.0)	
Level III	5 (6.5)	5 (6.5)	5 (7.5)		6 (7.6)	5 (6.8)	7 (10.4)	
Level IV	13 (16.9)	13 (16.9)	38 (56.7)		41 (51.9)	39 (53.4)	34 (50.7)	
NA [aged ≤ 4 years]	49 (63.6)	44 (57.1)	0 (0.0)		4 (5.1)	1 (1.4)	0 (0.0)	
**DASS 21, *n* [%]**								
**Depression**		*n =* 76 ^b^						
Normal	48 (62.3)	61 (80.3)	33 (49.3)	<0.001	73 (92.4)	53 (72.6)	31 (46.3)	<0.001
Mild	7 (9.1)	8 (10.5)	13 (19.4)		6 (7.6)	7 (9.6)	22 (32.8)	
Moderate	14 (18.2)	6 (7.9)	20 (29.9)		0 (0.0)	11 (15.1)	14 (20.9)	
Severe	3 (3.9)	0 (0.0)	1 (1.5)		0 (0.0)	2 (2.7)	0 (0.0)	
Extremely severe	5 (6.5)	1 (1.3)	0 (0.0)		0 (0.0)	0 (0.0)	0 (0.0)	
**Anxiety**		*n =* 76 ^b^				*n =* 72 ^b^		
Normal	45 (58.4)	56 (73.7)	4 (6.0)	<0.001	79 (100.0)	28 (38.9)	3 (4.5)	<0.001
Mild	14 (18.2)	6 (7.9)	4 (6.0)		0 (0.0)	10 (13.9)	2 (3.0)	
Moderate	2 (2.6)	7 (9.2)	27 (40.3)		0 (0.0)	22 (30.6)	32 (47.8)	
Severe	9 (11.7)	3 (3.9)	26 (38.8)		0 (0.0)	10 (13.9)	20 (29.9)	
Extremely severe	7 (9.1)	4 (5.3)	6 (9.0)		0 (0.0)	2 (2.8)	10 (14.9)	
**Stress**		*n =* 76 ^b^						
Normal	35 (45.5)	54 (71.1)	10 (14.9)	<0.001	34 (43.0)	56 (76.7)	5 (7.5)	<0.001
Mild	19 (24.7)	10 (13.2)	34 (50.7)		32 (40.5)	11 (15.1)	37 (55.2)	
Moderate	10 (13.0)	9 (11.8)	23 (34.3)		13 (16.5)	5 (6.8)	18 (26.9)	
Severe	8 (10.4)	1 (1.3)	0 (0.0)		0 (0.0)	1 (1.4)	7 (10.4)	
Extremely severe	5 (6.5)	2 (2.6)	0 (0.0)		0 (0.0)	0 (0.0)	0 (0.0)	

^a^ Friedman test, ^b^ missing data.

**Table 3:** Descriptive findings among children in Group A (*n =* 77) and Group B (*n =* 79) according to their age at baseline.

	Group A Aged Less Than 5 Years			Group A Aged 5 or More Years			Group B Aged Less Than 5 Years			Group B Aged 5 or More Years		
Time Point	0 m (*n =* 56)	6 m (*n =* 56)	12 m (*n =* 49)	0 m (*n =* 21)	6 m (*n =* 21)	12 m (*n =* 18)	0 m (*n =* 7)	6 m (*n =* 6)	12 m (*n =* 6)	0 m (*n =* 71)	6 m (*n =* 67)	12 m (*n =* 61)
**GMFM 66**												
Mean (SD)	31.1 (18.3)	42.1 (20.1)	41.6 (17.8)	36.6 (19.3)	42.6 (20.6)	44.0 (19.7)	34.2 (4.4)	36.7 (30.0)	31.5 (21.3)	40.1 (21.9)	47.3 (22.9)	42.1 (19.1)
Median [IQR]	30.2 [14.8, 46.0]	43.2 [24.8, 54.4]	46.1 [27.2, 52.4]	43.6 [17.8, 51.0]	44.5 [22.9, 59.1]	43.6 [29.0, 52.6]	36.0 [10.4, 58.6]	33.7 [11.1, 64.1]	29.8 [10.4, 50.2]	48.5 [16.0, 59.9]	47.7 [30.0, 66.7]	44.4 [24.0, 55.1]
*p*-value	Across all three time points: **<0.001**, 0–6 m: **<0.001**, 6–12 m: 0.068, 0–12 m: **<0.001**			Across all three time points: 0.058, 0–6 m: **0.009**, 6–12 m:0.492, 0–12 m: 0.093			Across all three time points: 0.607, 0–6 m: 0.600, 6–12 m: 0.345, 0–12 m: 0.463			Across all three time points: **<0.001**, 0–6 m: **<0.001**, 6–12 m: **<0.001**, 0–12 m: 0.147		
**GMFCS level**												
Level I	4 (7.3)	11 (19.6)	4 (8.2)	0 (0.0)	0 (0.0)	1 (5.6)	1 (14.3)	1 (16.7)	0 (0.0)	0 (0.0)	6 (9.0)	1 (1.6)
Level II	5 (9.1)	9 (16.1)	6 (12.2)	5 (23.8)	5 (23.8)	0 (0.0)	1 (14.3)	1 (16.7)	1 (16.7)	21 (29.6)	19 (28.4)	11 (18.0)
Level III	14 (25.5)	14 (25.0)	13 (26.5)	0 (0.0)	5 (23.8)	7 (38.9)	1 (14.3)	1 (16.7)	1 (16.7)	18 (25.4)	10 (14.9)	15 (24.6)
Level IV	8 (14.5)	10 (17.9)	13 (26.5)	8 (38.1)	4 (19.0)	4 (22.2)	1 (14.3)	0 (0.0)	1 (16.7)	6 (8.5)	16 (23.9)	14 (23.0)
Level V	24 (43.6)	12 (21.4)	13 (26.5)	8 (38.1)	7 (33.3)	6 (33.3)	3 (42.9)	3 (50.0)	3 (50.0)	26 (36.6)	16 (23.9)	20 (32.8)
*p*-value	Across all three time points: **<0.001**, 0–6 m: **<0.001**, 6–12 m: **<0.001**, 0–12 m: 0.192			Across all three time points: 0.107, 0–6 m: 0.285, 6–12 m:0.792, 0–12 m: 0.776			Across all three time points: 0.097, 0–6 m: 0.317, 6–12 m: 0.180, 0–12 m: 0.102			Across all three time points: **<0.001**, 0–6 m: **0.001**, 6–12 m: **<0.001**, 0–12 m: 0.168		
**CFCS level**										*n =* 69		
Level I	13 (23.2)	16 (28.6)	13 (26.5)	2 (9.5)	8 (38.1)	3 (16.7)	1 (14.3)	1 (16.7)	0 (0.0)	19 (27.5)	28 (41.8)	19 (31.1)
Level II	4 (7.1)	4 (7.1)	3 (6.1)	8 (38.1)	3 (14.3)	5 (27.8)	0 (0.0)	0 (0.0)	1 (16.7)	11 (15.9)	5 (7.5)	5 (8.2)
Level III	7 (12.5)	10 (17.9)	14 (28.6)	4 (19.0)	3 (14.3)	5 (27.8)	1 (14.3)	2 (33.3)	2 (33.3)	12 (17.4)	7 (10.4)	16 (26.2)
Level IV	9 (16.1)	9 (16.1)	7 (14.3)	2 (9.5)	3 (14.3)	1 (5.6)	3 (42.9)	1 (16.7)	0 (0.0)	13 (18.8)	11 (16.4)	3 (4.9)
Level V	18 (32.1)	17 (30.4)	12 (24.5)	5 (23.8)	4 (19.0)	4 (22.2)	2 (28.6)	2 (33.3)	3 (50.0)	14 (20.3)	16 (23.9)	18 (29.5)
N/A (aged < 2 y)	5 (8.9)	0 (0.0)	0 (0.0)	0 (0.0)	0 (0.0)	0 (0.0)	0 (0.0)	0 (0.0)	0 (0.0)			
*p*-value	Across all three time points: **<0.029**, 0–6 m: **0.001**, 6–12 m: 1.00, 0–12 m: **0.045**			Across all three time points: **0.029**, 0–6 m: **0.011**, 6–12 m:0.083, 0–12 m: 0.705			Across all three time points: 0.807, 0–6 m: 0.564, 6–12 m: 0.414, 0–12 m: 0.705			Across all three time points: 0.110, 0–6 m: 0.177, 6–12 m: **0.015**, 0–12 m: 0.131		
**VSS**												
Level I	2 (3.6)	2 (3.6)	11 (22.4)	2 (9.6)	6 (28.6)	2 (11.1)	0 (0.0)	0 (0.0)	0 (0.0)	15 (21.1)	28 (41.8)	20 (32.8)
Level II	1 (1.8)	3 (5.4)	6 (12.2)	5 (23.8)	4 (19.0)	5 (27.8)	0 (0.0)	0 (0.0)	1 (16.7)	13 (18.3)	0 (0.0)	5 (8.2)
Level III	1 (1.8)	2 (3.6)	2 (4.1)	4 (19.0)	3 (14.3)	3 (16.7)	0 (0.0)	1 (16.7)	2 (33.3)	6 (8.5)	4 (6.0)	5 (8.2)
Level IV	3 (5.4)	5 (8.9)	30 (61.2)	10 (47.6)	8 (38.1)	8 (44.4)	4 (57.1)	4 (66.7)	3 (50.0)	37 (52.1)	35 (52.2)	31 (50.8)
Not applicable	49 (87.5)	44 (78.6)	0 (0.0)	0 (0.0)	0 (0.0)	0 (0.0)	3 (42.9)	1 (16.7)	0 (0.0)	0 (0.0)	0 (0.0)	0 (0.0)
*p*-value	Across all three time points: **<0.001**, 0–6 m: **0.041**, 6–12 m: **<0.001**, 0–12 m: **<0.001**			Across all three time points: **0.023**, 0–6 m: **0.014**, 6–12 m:0.070, 0–12 m: 0.414			Across all three time points: 0.368, 0–6 m: 0.180, 6–12 m: 1.000, 0–12 m: 0.144			Across all three time points: **0.045**, 0–6 m: **0.011**, 6–12 m: 0.197, 0–12 m: 0.236		
**DASS 21**												
**Depression**					(*n =* 20)							
Mean (SD)	4.9 (4.1)	2.3 (2.6)	5.3 (2.7)	5.9 (5.7)	2.3 (1.6)	4.7 (1.6)	2.6 (2.1)	0.8 (1.6)	5.7 (1.5)	2.4 (1.4)	2.8 (2.7)	4.8 (1.9)
Median [IQR]	4.00 [2.0, 7.0]	2.0 [1.0, 3.0]	5.0 [3.0, 8.0]	4.0 [2.0, 8.0]	2.0 [1.0, 3.7]	4.0 [3.7, 6.2]	3.0 [1.0, 4.0]	0.0 [0.0, 1.7]	5.0 [4.7, 7.2]	2.0 [2.0, 3.0]	2.0 [1.0, 4.0]	5.0 [3.0, 6.0]
*p*-value	Across all three time points: **<0.001,** 0–6 m: **<0.001**, 6–12 m: **<0.001**, 0–12 m: 0.189			Across all three time points: **0.016**, 0–6 m: **0.003**, 6–12 m: **0.003**, 0–12 m: 0.943			Across all three time points: 0.094, 0–6 m: 0.197, 6–12 m: **0.042**, 0–12 m: 0.072			Across all three time points: **<0.001**, 0–6 m: 0.725, 6–12 m: **<0.001**, 0–12 m: **<0.001**		
**Anxiety**			(*n =* 47)		(*n =* 20)						(*n =* 66)	
Mean (SD)	4.0 (3.9)	2.7 (3.1)	7.1 (2.2)	4.2 (4.8)	2.4 (3.6)	7.1 (2.5)	0.6 (0.5)	3.2 (3.8)	6.0 (2.2)	0.9 (0.9	4.9 (3.1)	7.2 (2.2)
Median [IQR]	2.5 [1.0, 5.0]	2.0 [0.0, 4.0]	7.0 [5.0, 9.0]	3.0 [1.0, 6.0]	1.0 [0.0, 2.7]	8.0 [5.5, 9.0]	1.0 [0.0, 1.0]	2.0 [0.0, 6.7]	7.0 [4.2, 7.2]	1.0 [0.0, 1.0]	5.0 [2.0, 7.0]	7.0 [5.0, 9.0]
*p*-value	Across all three time points: **<0.001**, 0–6 m: **0.046**, 6–12 m: **<0.001**, 0–12 m: **<0.001**			Across all three time points: **0.004**, 0–6 m: 0.102, 6–12 m: **0.006**, 0–12 m: 0.093			Across all three time points: **0.022**, 0–6 m: 0.144, 6–12 m: 0.173, 0–12 m: **0.027**			Across all three time points: **<0.001**, 0–6 m: **<0.001**, 6–12 m: **<0.001**, 0–12 m: **<0.001**		
**Stress**			(*n =* 48)		(*n =* 20)							
Mean (SD)	8.2 (3.9)	5.8 (3.7)	8.4 (2.6)	9.7 (4.1)	5.5 (3.6)	8.1 (3.1)	6.4 (3.0)	3.0 (2.8)	7.7 (1.4)	7.5 (2.5)	5.4 (2.9)	9.3 (3.0)
Median [IQR]	7.0 [5.0, 11.0]	5.0 [3.0, 8.0]	8.5 [7.0, 10.0]	9.0 [7.5, 11.5]	5.5 [3.0, 8.0]	8.5 [5.7, 11.0]	7.0 [3.0, 9.0]	2.0 [0.7, 6.2]	7.5 [6.7, 8.5]	8.0 [6.0, 9.0]	5.0 [4.0, 8.0]	9.0 [7.0, 12.0]
*p*-value	Across all three time points: **<0.001**, 0–6 m: **<0.001**, 6–12 m: **<0.001**, 0–12 m: 0.608			Across all three time points: **0.010**, 0–6 m: **0.002**, 6–12 m: 0.058, 0–12 m: 0.382			Across all three time points: 0.066, 0–6 m: 0.206, 6–12 m: 0.066, 0–12 m: 0.136			Across all three time points: **<0.001**, 0–6 m: **<0.001**, 6–12 m: **<0.001**, 0–12 m: **0.009**		

The statistically significant values are in bold.

## Data Availability

Data are available upon reasonable request from the corresponding author.

## Supplementary Materials

The following is available online at https://www.mdpi.com/article/10.3390/brainsci11091189/s1, Supplementary Material S1: “Shishu Shorgo” Transition Program Manual.
