# Supplementary material for: Outcome of Community-Based Early Intervention and Rehabilitation for Children with Cerebral Palsy in Rural Bangladesh: A Quasi-Experimental Study
Source: Brain Sci. 2021 Sep 10;11(9):1189. doi: 10.3390/brainsci11091189 (PMC8469407; doi:10.3390/brainsci11091189)
Supplement: Supplementary file 1 [file brainsci-11-01189-s001.zip › brainsci-1326133-SI.pdf]

SHISHU SHORGO

Children's Heaven

Transition Program Manual

2015

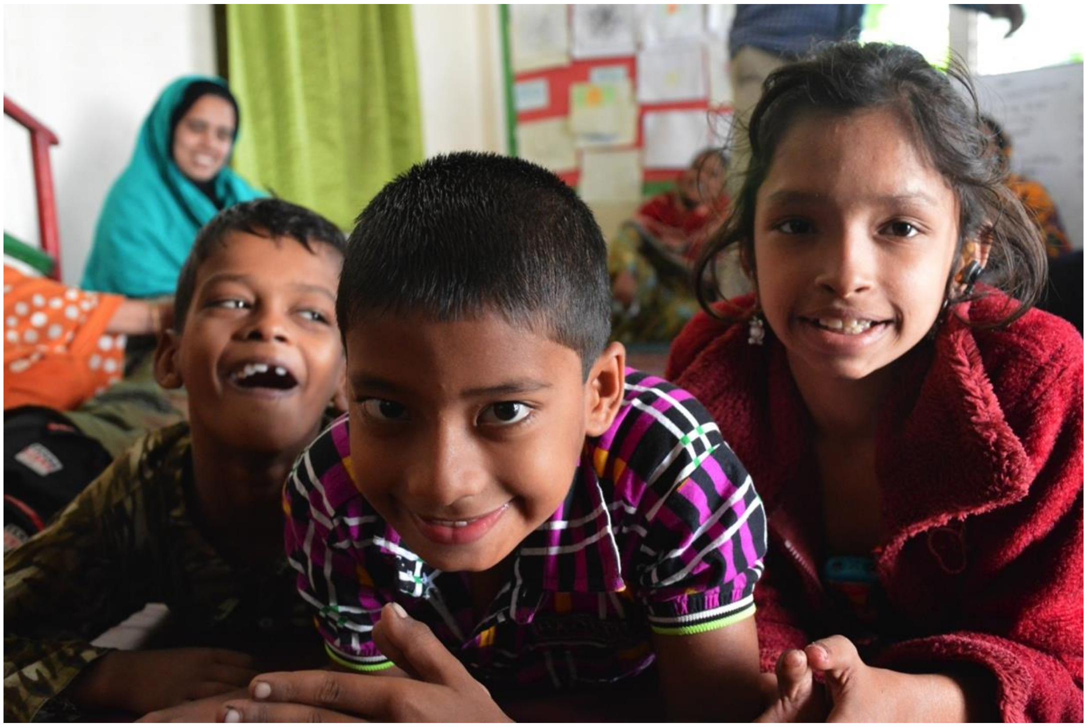

Authors

Cate Biesot

Occupational Therapist

Australian Youth Ambassador for Development 2013-2014

Child Sight Foundation (CSF)

Dr Namita Jacob

Regional Education Specialist, Asia Pacific

Perkins International

Acknowledgements

The transition program and the accompanying manual was a collaborative effort between:

- **Children with disabilities and their families** living in Shahjadpur, Bangladesh. Thank you for being patient with us while CSF has developed our programs, and your enthusiasm in participating in CSF's programs. Thank you to the parents for their ongoing dedication to their children with disabilities, striving to provide the best opportunities for their children.

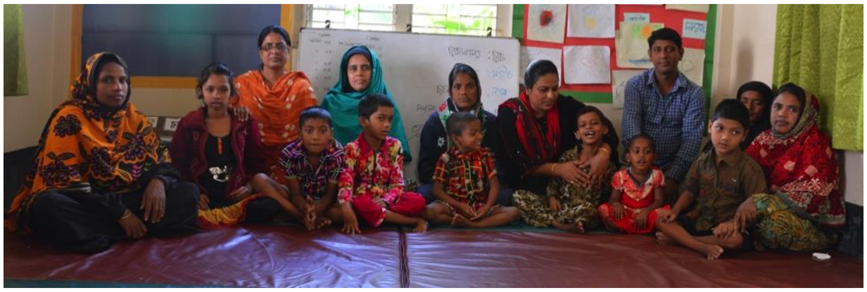

- **CSF** www.csf-global.org, a non-government, charity organisation that works to fulfil the rights of children with vision impairment and additional disabilities in Bangladesh. CSF's mission and vision is based on the United Nations Convention to the Rights of Persons with Disabilities (UNCRPD). CSF is reputed for their research into causes and the identification of vision impairment in children in Bangladesh. CSF is the pioneer of the Key Informant Method, which has also been introduced into other developing countries. CSF is also reputed for providing ophthalmology, optometry and cataract surgery to children and adults in both rural and city areas of Bangladesh. Through their research, CSF has identified a large number of children living with vision impairment as well as additional disabilities. CSF is expanding it's services to strive to achieve the rights of these children living with multiple disabilities, through providing parent education programs

- **Perkins International** http://www.perkinsinternational.org/, an international non-government organisation who work with children with vision impairment and additional disabilities. Dr. Namita Jacob, education specialist for Asia/Pacific provided editing support for this manual and the manual incorporates materials and ideas from Perkins International trainings and resources. A special thank you to Perkins International Asia Pacific team who continue to provide invaluable technical support to CSF's Shishu Shorgo.

- **Australian Volunteers for International Development**; an Australian Government, Australian Aid initiative. Under the program, Australian Youth Ambassador for Development, Cate Biesot, an Occupational Therapist, volunteered her time with CSF in Bangladesh from 2013 to 2014. Her volunteer assignment was to work with CSF

and their partners to start Shishu Shorgo and it's transition program. The manual is a product of her year's work and experience with this program.

Table of Contents

Acknowledgements 2

Introduction 6

Part 1: ASSESSMENT AND GOAL SETTING 12

- **Client Profile 13**
- **Individual Assessment 14**

Part 2: GROUP THERAPY 22

- **Program Goals 23**
- **Timetables 27**
  - **Month 1: Who Am I 27**
  - **Month 2: My Family and Friends 31**
  - **Month 3: The World Around Me 35**
  - **Month 4: School and Productivity 39**
- **National Anthem 43**
- **Welcome Circle 45**
- **Music Therapy & Rhymes 50**
- **Story Time 53**
- **Classes 58**
  - **Month 1: Who am I 58**
  - **Month 2: My Family and Friends 81**
  - **Month 3: The World Around Me 106**
  - **Month 4: School and Productivity 127**
- **Nasta & Toilet Break 149**
- **Movement 159**
  - **Stretching 160**
  - **Individual Movement 168**
  - **Group Movement 176**
- **Goodbye Circle and Homework 208**
- **Group Therapy Progress Report 210**

Part 3: COMMUNITY FOLLOW-UP 218
Community Follow-up Checklist 219
Home and Community Environment and Participation Action Record 236
School Environment and Participation Action Record 246
Vocation Environment and Participation Action Record 259

APPENDIX: Communication Resources 260
Augmentative and Alternative Communication (AAC) Fact Sheet 261
Picture cards for Group Therapy Activities 266
Orientation Communication Board 268
“I feel” Picture Cards 269
Feelings Symbols 273
Actions Symbols 275
ABC Alphabet and Number Board 276
Example Communication Boards 277
Visual Timetables 282

Introduction

Shishu Shorgo, or Children's Heaven, is CSF's Disability Support Centre based in Shahjadpur, a village 200km from Bangladesh's capital city Dhaka. In collaboration with their partners, CSF developed this manual to assist their field workers run a seven month transition program through Shishu Shorgo, for children with disabilities and their families living in Shahjadpur. CSF has worked extensively in remote areas of Bangladesh, where children with disabilities are often excluded from schools and access to medical, support and therapy services are extremely limited.

The goals and structure of the transition program were based upon a needs analysis conducted by CSF in April 2013 with children with disabilities and families living in Shahjadpur. Parents and their children with disabilities expressed the need for the Disability Support Centre to provide:

- Therapy to help their child communicate, mobilise, and care for themselves e.g. feed themself
- A place for their child to play with other children
- Daily programs (Sunday – Thursday) for 2 – 3 hours per day held at the centre rather their homes, which parents can also attend and assist as possible

The parents identified that therapy is required to give their child the opportunity to:

- Be able to attend school and receive an education
- Be independent, get married, find a job and earn money
- Have friends

In order to cater for the large number of children living with disabilities in Shahjadpur and other remote villages CSF works with, this intensive group therapy program was time limited to four months, with community follow-up provided up to seven months, to help transition to community, school and family support.

The goal of the transition program is to:

Increase the child's participation in their family, school and community.

What this Transition Program Involves

The transition program comprises:

- Parent support and education
- Group therapy
- Community follow-up

Parent Support and Education
Parent support and education is provided through involving the child's caregivers in all elements of the transition program. The teachers and fieldworkers must involve the parent in the group therapy and community follow-up by providing family-centred care, keeping the caregiver informed about the child's progress, and providing recommendations and empowering caregivers to advocate for and facilitate their child's participation in their home, community, school and vocation. Through this program, caregivers form support networks with other caregivers of children with disabilities.

Caregivers are also encouraged to participate in CSF's Caregiver training, which follows CSF's ***Getting to Know Cerebral Palsy*** **Caregiver Training Manual**. This is discussed during the child's initial assessment and goal setting.

Group therapy
The group therapy is provided as an intensive four month program that focuses on assisting the child build the following skills:

- Activities of daily living (toileting, dressing, eating)
- Functional mobility
- Play
- Communication
- School-readiness
- Productivity

The child attends the sessions with at least one primary caregiver, allowing both to develop skills.

Community follow-up
Throughout the seven month transition program, CSF staff provides the child and family follow-up in the community. The goal of this community follow-up is required to facilitate the transition process by:

- Providing strategies and assistive devices to assist the child at home with activities of daily living (washing, toileting, eating, dressing).
- Supporting the child's local school to enable their admission to school and participation in school activities.
- Increasing awareness about disability and the child's abilities and rights to facilitate the child's family and community develop support networks and increase opportunities for the child to participate in the community.
- Supporting the child to develop a meaningful vocation in their family and community, particularly for those children unable to attend school (e.g. sorting vegetables).

Important things to know before starting the Transition Program

- This manual was developed to assist adults who are not rehabilitation professionals provide intervention to support children with disabilities in their community. In order to provide safe and effective rehabilitation, technical support from rehabilitation professionals is required.

- While this manual was developed specifically for CSF's Disability Support Centre in Bangladesh, the manual is transferrable to other organisations working in developing countries.

- **This manual is to be used as a guide only. Activities should be changed or adapted to suit the unique abilities of the child and their economic, social and cultural context.**

- Community follow-up needs to be provided as soon as the child starts the transition program.

- Since the program is implemented over a very short period, it is recommended that children are grouped into similar age groups, skills and abilities, in order to more effectively target their goals, for example:

  - Group A – Older children aged ~10+
    Goal: To increase participation in their family and community
    Focus: Functional literacy and numeracy and other pre vocational skills

  - Group B – School aged children ~6-10 years old
    Goal: For admission to school, to increase participation in their family and school.
    Focus: Pre reading, pre math and pre writing, moving onto literacy and numeracy. Children need to be guided quickly and intensively through the pre skills. Many of them do not have pre skills mostly because they have not had any exposure or opportunity for learning.

  - Group C – Younger children aged 2-6 years old
    Goal: To achieve their developmental milestones and readiness for school.
    Focus: Developmental milestones (as shown in the developmental charts on the assessment form), school readiness skills. If the centre has the capacity, this group will remain with the program through the early intervention period.

- It is recommended that there is a maximum of 10 children per group, with two field staff who are referred to as teachers in this manual.

- There shall be at least two field staff that provide community follow-up. They are referred to as field workers in this manual.

Structure of the Transition Program

Before admission: Assessment and Goal Setting	1. Select children for potential admission to Shishu Shorgo's Transition Program<br>2. Conduct the **Initial Assessment and Goal Setting**. Identify whether the child's goals are for increased participation.<br>3. If the goal includes increased participation, admit to the Disability Support Centre.	
Admission		
Month 1 - 4	**Provide Group therapy**<br>1. Allocate the child to a particular group according to their age, goals and abilities, for example group A, B or C.<br>2. Ensure a maximum of 10 children are in each group.<br>3. Ensure two teachers are available for each group.<br>4. Provide intervention using the **Group Therapy Program Guideline**. Adapt activities according to the child's goals and abilities.	**Provide Community Follow-Up**<br>1. As soon as the child is admitted to the centre, start completing the **Community Follow-Up Checklist**
Month 4	**Child graduates from Group Therapy Program**	**Continue providing Community Follow-Up, as per the Community Follow-Up Checklist.**
Month 5 - 6		
Month 7		**Discharge from Shishu Shorgo Transition Program**

How to use this Manual

The manual comprises:

1. **An Initial Assessment and Goal Setting tool.** This holistic assessment and goal setting must be conducted prior to admission to the Disability Support Centre and readministered every three months.

2. **A guideline for the group therapy program**. This program is a four month intensive program that is designed to be run five days a week for approximately three hours.

   The program has a different theme each month. These themes are:
   - Month 1: Who am I
   - Month 2: My Family and Friends
   - Month 3: The World Around Me
   - Month 4: School and Productivity

   This group therapy guideline includes:
   - Goals for each theme
   - Timetable for each week
   - Activities
   - Augmentative and Alternative Communication (AAC) fact sheet and example templates
   - Monthly progress report

   Activities are grouped in different sessions, and include:
   - National Anthem
   - Welcome Circle
   - Music therapy and Rhymes
   - Story time
   - Movement
   - Nasta and toilet break
   - Classes
   - Goodbye Circle and Homework

   For every activity there is listed:
   - Skills the activity will help build
   - Resources required
   - Instructions for the teacher to run the activity

   The 'classes' are set for each day. For other sessions, the teacher can choose the activity each day from the range provided in the manual. Try and tie the activity chosen into the theme.

   Every child has a unique set of interests, abilities and goals. It is important to **tailor the activities to help the child achieve success and learn**. You can also change the activities so that the children can learn about and participate in festivals and other important events happening in your community.

3. **A guideline for providing community follow-up.** This involves a series of checklists:
   - **Community Follow-up Checklist:** This provides an overall structure for the Community Follow-up program, and when and how to conduct each checklist.
   - **Home Environment Checklist:** To be used for all children.
   - **Home and Community Participation Checklist:** To be used for all children.
   - **School Participation Checklists:** To be used for those children whose goal is to participate in school.
   - **Vocation Checklists:** To be used for those children whose goal is to participate in a vocation.
   - **Action Records:** To be used to record progress with implementing strategies, assistive devices and modifications that assist the child participate in home, community, school and vocational activities

4. **Communication resources.** These resources provide information and examples of Alternative and Augmentative Communication (AAC) and must be referred to and utilised throughout all aspects of the Transition Program. The communication resources include:
    - Augmentative and Alternative Communication (AAC) Fact Sheet
    - Picture cards for Group Therapy Activities
    - Orientation Communication Board
    - “I feel” Picture Cards
    - Feelings Symbols
    - Actions Symbols
    - ABC Alphabet and Number Board
    - Example Communication Boards
    - Visual Timetables

Part 1:

ASSESSMENT

AND

GOAL SETTING

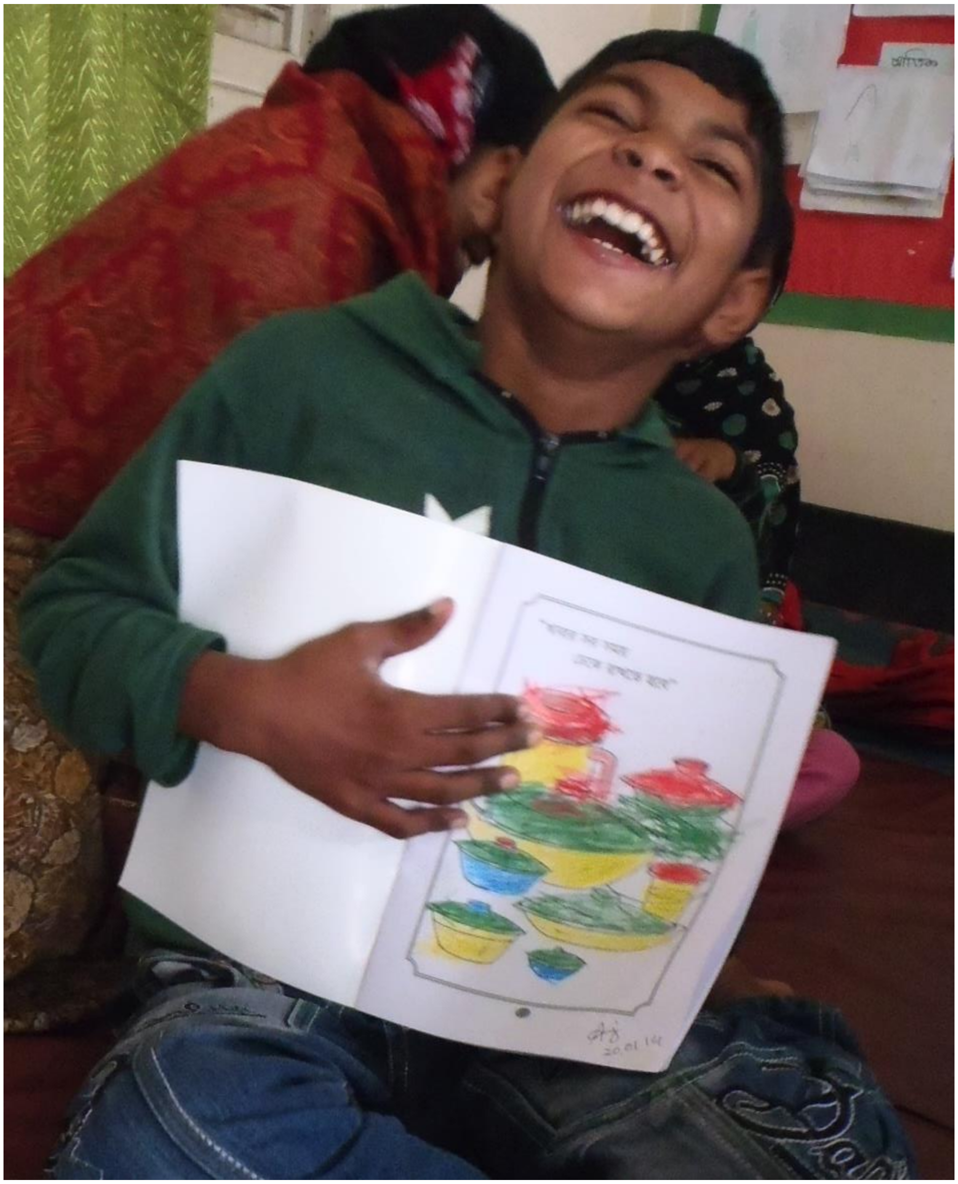

Client Profile

ID Code	
Child's name	
Child's date of birth, season of birth	
Child's address	
Parent's phone number	
Child's disability	
Other diagnoses	
Medication	
Has your child had fits? If yes, are they taking medication for this?	
Mother's name, and job	
Father's name, and job	
Who is your child's main caregiver?	
Sisters and brothers names and date of birth	
Is your child going to school? If yes, what is the name of the school? What is the teachers' name?	
Doctor's name	
Other supports (other members of the community/families, other organisations)	

Individual Assessment

Date/s of assessment:	Child's age:
Name of Assessor:	Assessor's position title:

1. **Daily Routine.** Say: "Therapy is all about trying to help your child do every-day activities such as feeding themself, sitting themselves, playing with other children and going to school, as independently and safely as possible. So first I need to know what activities your child is currently doing every day. I will ask you some questions about what your child does during the day".

What does your child's morning look like? What do they do? Who are they with?

What does your child's midday look like? What do they do? Who are they with?

What does your child's afternoon look like? What do they do? Who are they with?

What does your child's night look like? What do they do? Who are they with?

2. Current level of independence. Say: "Now that I know your child's current routine, I want to find out how much your child can do independently and what he/she needs help with during particular activities. I will ask you about a particular activity, what your child can do by themself and what they can't do by themself.

Type of Activity	*Activity*		✓ **Tick if can do independently**	✗ **Cross if cannot do independently**	**Comments**
Self-Care	**Washing**	Hands			
		Hair, face			
		Body			
		Brushing teeth			
		Brushing hair			
	**Dressing**	Pulling up/down pants			
		Putting on/off pants			
		Putting on/off top or dress			
		Doing up/undoing buttons			
		Putting on/off shoes			
	**Toileting**	Signalling when need to use toilet			
		Sitting on toilet seat			
		Squatting			
	**Sitting**	On floor			
		On lap			
		On chair			
	**Moving**	Reaching with one hand			
		Grasping with one hand			
		Reaching with both hands			
		Grasping with both hands			
		Rolling			
		Crawling			
		Standing			
		Walking on flat/ uneven ground			
		Walking up and down steps			

Type of Activity	Activity		✓ **Tick if <u>can</u> do independently**	✗ **Cross if <u>cannot</u> do independently**	**Comments**
Self-Care	**Communicating**	Making sounds			
		Making choices by sound/pointing/words/ other			
		Making facial expressions			
		Saying words			
		Saying sentences			
		Understand 1 step instructions that is not part of a usual routine			
		Understand 2-3 step instructions that are not part of a usual routine			
	**Eating and drinking**	Feeding self			
		Drinking from cup			
Productivity	**Play**	Playing alone			
		Paying with other children			
		Does your child build/make things?			
		Does your child use objects for other purposes e.g. a small brick for a phone?			
	**School**	Going to/from school			
		Sitting in class			
		Participating in class			
		Doing homework			
	**Helping around the house**	Getting snack			
		Making snack/meal			
		Cleaning			
		Helping brothers/sisters			

3. Goal Setting. Say: Which five activities are of most importance to you? (list in the table below). Over the next six months, our goals will be to help your child improve their independence in these five activities. I am going to ask you how well you think your child does this activity now, and how satisfied you are with your child's current ability to do this activity. I will ask you to rate your level of satisfaction on a scale of 1 to 5. 1 is not satisfied at all, and 5 is completely satisfied. After three months of therapy, I will ask you the same questions. This will be how we measure your child's improvement. After three months we will re-write the goals. Sometimes three months is long enough to achieve a goal, but sometimes if it's a big goal, a child needs more time. So when we re-write the goals in three months, some goals will be new, and some may stay the same".

	**Initial goals**<br>Date:		**3 month review of goals**<br>Date:	
**Top 5 important activities**	**How well does your child perform this task?**	**How satisfied are you with your child's current performance?**	**How well does your child perform this task?**	**How satisfied are you with your child's current performance?**
1.				
2.				
3.				
4.				
5.				

Say: "Working towards these goals involves your child participating in therapy at the centre and you continuing therapy at home. If you agree with the above goals and agree to continue therapy at home, sign below":

Parent signature: Staff signature: Date:

Sign and date when **6 month review** completed by parent and staff member.

Parent signature: Staff signature: Date:

4. Performance components. <u>Say</u>: "Next I need your child to show me exactly what he/she can do – in other words, what skills your child has and has not developed. This will help me work out why your child is having trouble doing these activities. Once we work out what skills are missing, we can help develop these skills so that he/she can be more independent in the activity. I am going to see what physical skills and social and thinking skills your child has developed. First I will start with physical skills".

Assessment of Child's Physical Skill Development

~ usually developed at 0 – 5 years ~

Date:______________

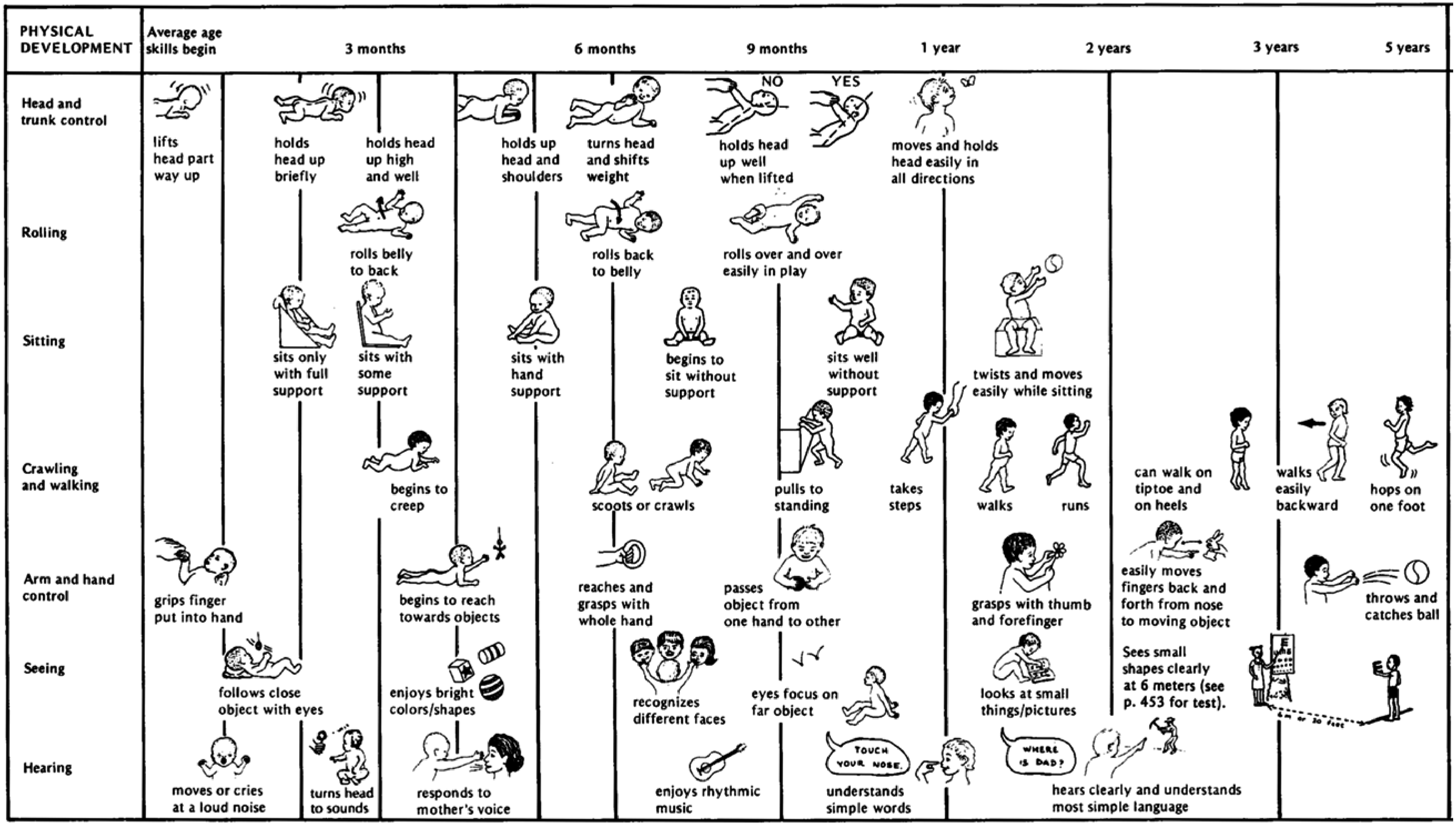

Say: “I will now find out what social and thinking skills your child has developed”.

Date:______________

Assessment of Child’s Social and Thinking Skill Development

~ usually developed at 0 – 5 years ~

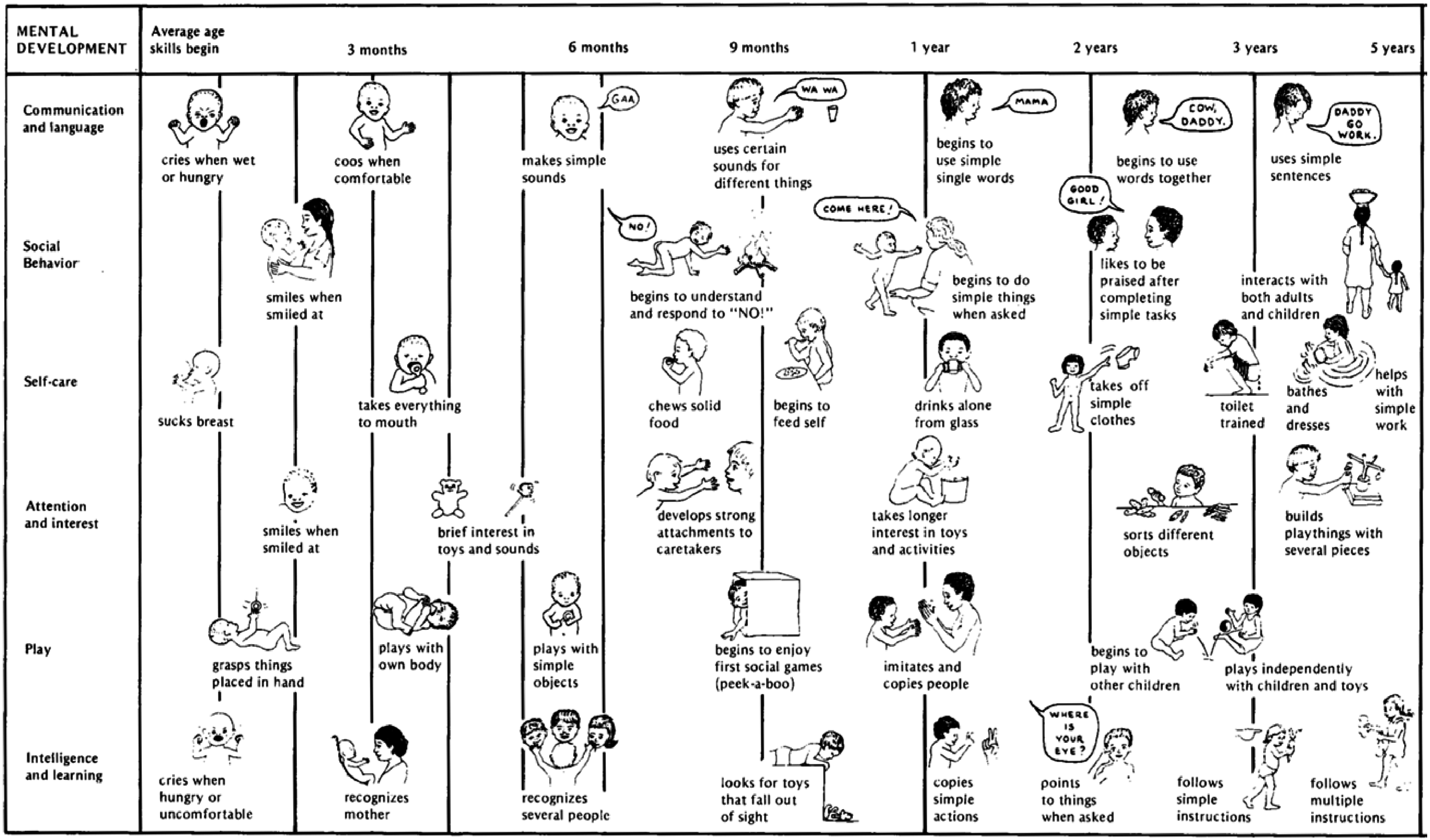

Assessment of Child's Physical, Social, Thinking and Communication Development

~ usually developed at 6 – 18 years ~

Date:______________

Skill Development	6 – 8 years	8 – 13 years	13 – 18 years
**Physical**	• Descends stairs alternating feet<br>• Balances on one foot to count of 5 to 10<br>• Two footed skip<br>• Walks on a balance beam<br>• Uses a bat to hit a ball<br>• Jumps about 30cms vertically, broad jumps about 1 metre<br>• Adult-like skill in throwing and catching<br>• Grasps pencil maturely but writes or draws with stiffness and concentration<br>• Draws recognisable person with head, body and limbs<br>• Threads needle<br>• Uses scissors<br>• Shuffles and sorts playing cards<br>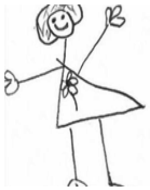	• In the previous age chunk children can use a bat to hit a ball, but this age period is when they can coordinate enough to use one object accurately to propel another one in a desired direction – caroms, hockey, table tennis and so on<br>• This is also the age when the child is better able to judge space, speed, and able to time their own movement and force to respond accurately<br>• Fine motor is also similarly well-developed so that the child is able to adjust grasp, force and movement to achieve very fine and precise actions from painting to needle work	• Adolescent growth spurt (girls before boys)<br>• Body changes associated with puberty<br>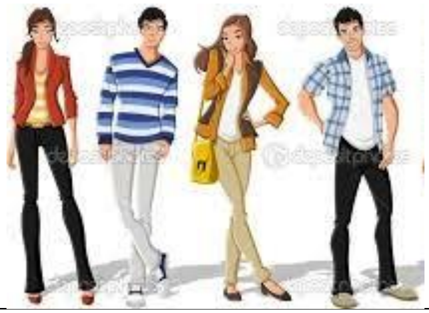
**Social**	• Associative and cooperative play<br>• Takes turns in conversation<br>• Understands and begins to use sarcasm<br>• Still very attached to and dependent on parents, but less need to be in direct physical contact with them<br>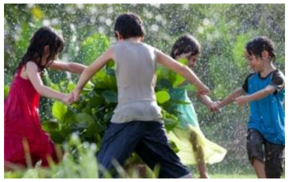	• Adjusts to personal space and the personal space of others<br>• Friends are usually of the same gender<br>• Forms close friendships in pairs or small groups<br>• More aware of status within group<br>• Feels greater peer pressure to conform<br>• Can recognise the 'moral' of a story<br>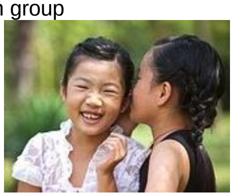	• Teenage 'hangouts' become important centres of activity<br>• Desire to be independent of parents<br>• Preoccupied with own behaviour, personality, physical appearance<br>• Friends with both genders<br>• Romantic relationships begin<br>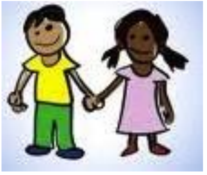
**Thinking**	• Learns to read and write<br>• Enjoys learning simple rhythms and movement routines<br>• Interested in riddles and jokes<br>• Describes self according to skills being acquired ('I can hop!')<br>• Assembles models and puzzles<br>• Unlocks door with key<br>• Folds paper along straight lines<br>• Ties shoes<br>• Knows right from left<br>• Begins to understand another person's perspective of the same situation<br>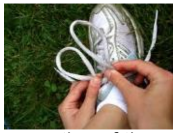	• Defines self in comparison with others ('I'm better at reading than she is')<br>• Aware of time, can plan events for the day<br>• Develops spatial and directional awareness for effective participation in games<br>• Develops understanding of metaphor, puns and jokes<br>• Maintains attention and concentration<br>• Likes to memorise and identify facts<br>• Can mask or 'fake' emotions<br>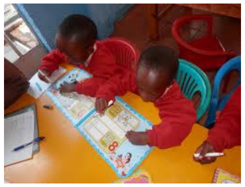	• Able to classify, arrange ideas, generalise<br>• Developing definite ideas and attitudes, for example, towards religion, politics, race etc.<br>• Understands more complex (abstract) concepts and hypothetical's<br>• Likes to discuss and debate<br>• Systematic problem-solving<br>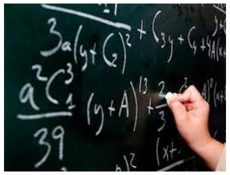
**Communication and Language**	• Shift from egocentric to social speech<br>• Speaks complete sentences<br>• Vocabulary expands to approx. 50 000 words by age 12<br>• Understands and recognizes irony, jokes and sarcasm	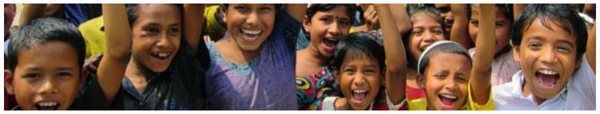	• Adopts personal speech patterns<br>• Communication becomes focus of relationships<br>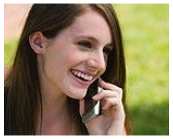

5. Need for Parent Training and Support: Say: "I am also worried about you, and how you are managing with caring for your child with a disability. Caring for any child, but even more a child with a disability, can be emotionally and physically exhausting. Often parents are unsure of the meaning of their child's disability and disability is not accepted by the community, leaving parents and their child feeling isolated and unsupported".

Say: "We are running a parent training group which discusses what disability means, the rights of people with disabilities, and different strategies to care for your child to enable their development and participation, and also help prevent you from injuring yourself while lifting and carrying your child".

Are you already involved in parent training?	Yes	No	If no, would you like to participate in the parent training?	Yes	No	If yes, provide parent with contact details for parent training.<br>Date provided:<br><br>Staff signature:

Say: "We also have a parent support group that meets here regularly. In this parent support group you will meet other parents of children with disabilities, get to share experiences, developed supportive friendships and socialise".

Are you already involved in the parent support group?	Yes	No	If no, would you like to participate in the parent support group?	Yes	No	If yes, provide parent with contact details for support group.<br>Date provided:<br><br>Staff signature:

Part 2:

GROUP THERAPY

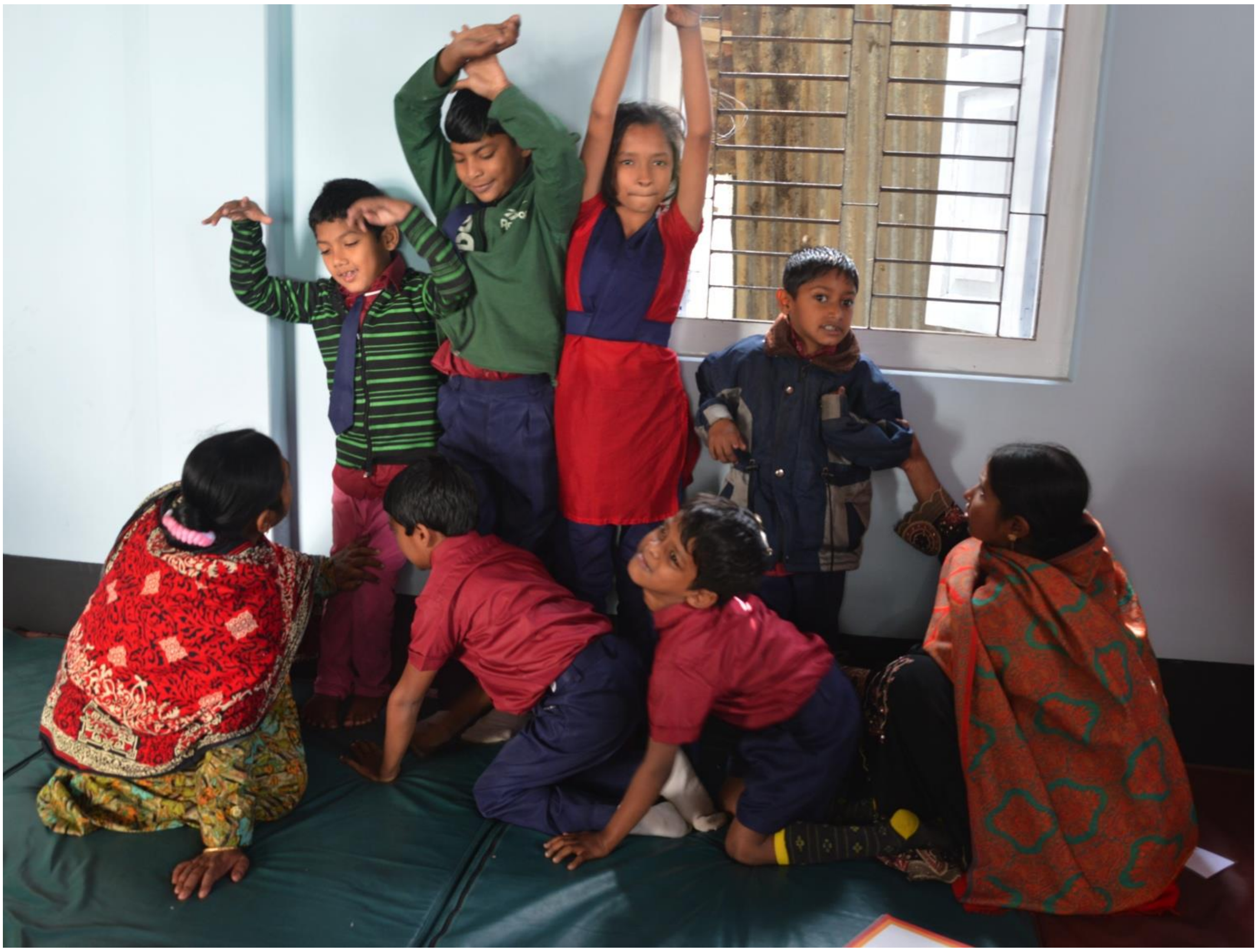

Group Therapy Program Goals

Please note: These group therapy program goals are generalised for the group of children. Children develop skills at different rates and thus activities need to be graded for different skill levels to facilitate development.

Month 1: Who am I

Goals

By the end of the month children will:

	Type of Skills
• Be independent in at least one step of each ADL task (e.g. identifying shoes, reaching under tap when appropriate) using required assistive devices<br>• Practice independence in a second step of each ADL task (practice to occur during group therapy and at home)	Activities of Daily Living
• Respond to their name, recognise the letters and say the sounds that make up their name<br>• Copy or write all or part of their name (this may involve rearranging letter cards to make their name for those children who have difficulty controlling a pencil/crayon)<br>• Increase volume and clarity of vocal sounds<br>• Be introduced to multiple forms of AAC and understand the outcome of communicating yes, no, more and finished using AAC<br>• Identify and communicate their feelings and personal needs<br>• Identify and communicate their likes and dislikes<br>• Listen and follow simple instructions	Language and Communication
• Hold a pencil/crayon (using required modifications/assistive devices) and draw straight and curved lines and shapes<br>• Maintain head position and sitting balance with required supports (special chair, cushions) on floor<br>• Maintain head position and sitting balance with required supports in chair at a table<br>• Increase control of isolated movement including reaching with hand and pointing with finger<br>• Practice using required assistive devices for increased independence with whole body movement (parallel bars for practicing standing and walking, walking frame, wheelchair)	Movement
• Identify object use<br>• Recall and identify different parts of their body<br>• Sequence two step activities<br>• Recall and follow the group therapy routine<br>• Recall the order of activities<br>• Count to 5 forwards and backwards, arrange numbers in sequence<br>• Add, subtract and divide up to 5 objects	Cognition
• Take turns<br>• Identify and express their own likes and dislikes<br>• Ask and understand others feelings, personal needs, likes and dislikes	Social

Month 2: My Family and Friends

Goals:

By the end of the month children will:

	Type of Skills
• Be independent in at least two steps of each ADL task<br>• Practice independence in a third step of each ADL task (practice to occur during natural routines at the center and home)	Activities of Daily Living
• Read and write their name or organise letter cards to make their name<br>• Pronounce, read and copy or write at least 10 letters<br>• Recognise letters they have learnt and the sounds they make in words<br>• Read and write at least 2 words<br>• Increase volume and clarity of sounds when pronouncing letters and words<br>• Communicate yes, no, more and finished using AAC when prompted<br>• Understand pictures and simple symbols<br>• Identify friends and family by picture<br>• Expand use of AAC to communicate with family and friends other needs and choices	Language and Communication
• Increase sitting balance (endurance) with reduced level of support for sitting and head control on floor and in chair at a table<br>• Control a pencil/crayon to write letters and words<br>• Increase control of isolated movement including reaching with hand and pointing with finger<br>• Increase arm and hand control for cutting using scissors, gluing, grasp and release, opening and closing containers<br>• Practice using required assistive devices for increased independence with mobility (parallel bars for practicing standing and walking, walking frame, wheelchair)	Movement
• Count to 10 forwards and backwards, arrange numbers in sequence<br>• Add, subtract and divide up to 10 objects (e.g. children to divide 9 tokens between three children so that they each have the same)<br>• Sequence three steps of a task<br>• Identify more and less, biggest and smallest	Cognition
• Identify the roles of members in their family (mother, father, sister, brother etc.)<br>• Recognise their role in their family (daughter/son, sister/brother etc.)<br>• Engage in activities to fulfil their role in their family<br>• Recognise their role as a friend<br>• Engage in activities to fulfil their role as a friend	Social

Month 3: The World around Me

Goals:

By the end of the month children will:

	Type of Skills
• Be independent in at least three steps of each ADL task<br>• Practice independence in a forth step of each ADL task (practice to occur during group therapy and at home)	Activities of Daily Living
• Identify vegetables and fruit<br>• Make choices e.g. vegetables and fruit to purchase/eat<br>• Identify, pronounce and copy or write at least 15 letters<br>• Recognise letters they have learnt and the sounds they make in words<br>• Read and write at least 5 words<br>• Pronounce letters and words in their speech more clearly<br>• Use individualised AAC at school and home to ask and answer questions and communicate choices	Language and Communication
• Increase dynamic sitting balance (leaning to reach) on floor<br>• Increase sitting balance and endurance in a chair at a table<br>• Control a pencil/crayon to write words and sentences<br>• Increase control of isolated movement including reaching with hand and pointing with finger<br>• Increase arm and hand control and endurance for cutting using scissors, gluing, grasp and release, opening and closing containers<br>• Increase independence and endurance with mobility (using required assistive devices)	Movement
• Identify money<br>• Match picture and object, find odd one out in a group of pictures or objects<br>• Count to 15 forwards and backwards, arrange numbers in sequence<br>• Add, subtract and divide up to 15 objects<br>• Sequence 4 steps of a task	Cognition
• Identify roles of different people in the community<br>• Greet, make choices, give money and receive change at the market	Social

Month 4: School and Productivity

Goals:

By the end of the month children will:

	Type of Skills
• Be independent in at least four steps of each ADL task<br>• Practice independence in a fifth step of the task (practice is to occur during group therapy and at home)	Activities of Daily Living
• Identify, pronounce and copy or write at least 20 letters<br>• Recognise letters they have learnt and the sounds they make in words<br>• Read and write at least 10 words<br>• Use AAC spontaneously at school and home<br>• Be understood by others when speaking and using AAC in conversation	Language and Communication
• Increase dynamic sitting balance and endurance.<br>• Increase handwriting endurance and legibility<br>• Increase control of isolated movement including reaching with hand and pointing with finger<br>• Increase arm and hand control and endurance for cutting using scissors, gluing, grasp and release, opening and closing containers<br>• Increase independence and endurance with mobility (using required assistive devices)	Movement
• Recall stories and relate stories to self/others<br>• Problem solve – provide multiple solutions<br>• Identify and count money to provide correct money to purchase item and change<br>• Count to 20 forwards and backwards, arrange numbers in sequence<br>• Add, subtract and divide up to 20 objects<br>• Sequence 5 steps of a task	Cognition
• Understand their role as a student<br>• Understand different working roles<br>• Develop goals – their dreams and aspirations	Social

Who Am I
Week 1 Timetable

<table>
<tr><th colspan="2">Time</th><th>Sunday</th><th>Monday</th><th>Tuesday</th><th>Wednesday</th><th>Thursday</th></tr>
<tr><th>Morning</th><th>Afternoon</th><th colspan="5">Activity</th></tr>
<tr><td>9:00am</td><td>2:00pm</td><td colspan="5">National Anthem
Take off shoes</td></tr>
<tr><td>9:15am</td><td>2:15pm</td><td>Welcome circle: Greetings</td><td>Welcome circle: Greetings</td><td colspan="3">Welcome circle: Greetings and Orientation</td></tr>
<tr><td>9:30am</td><td>2:30pm</td><td>Music Therapy: Welcome Song</td><td>Music Therapy: Welcome Song</td><td>Story Time</td><td>Sing song: When you’re happy and you know it</td><td>Music Therapy: Welcome Song</td></tr>
<tr><td>9:45am</td><td>2:45pm</td><td>Yoga</td><td>Individual Movement</td><td>Yoga</td><td>Individual Movement</td><td>Yoga</td></tr>
<tr><td>10:00am</td><td>3:00pm</td><td colspan="5" rowspan="2">Group Movement Activities</td></tr>
<tr><td>10:15am</td><td>3:15pm</td></tr>
<tr><td>10:30am</td><td>3:30pm</td><td colspan="5" rowspan="2">Morning/Afternoon tea and toilet break</td></tr>
<tr><td>10:45am</td><td>3:45pm</td></tr>
<tr><td>11:00am</td><td>4:00pm</td><td rowspan="3">Class 1: My name</td><td rowspan="2">Class 2: Orientation</td><td rowspan="2">Class 3: Feelings</td><td rowspan="3">Class 4: My shadow</td><td rowspan="2">Class 5: My Story, my portrait</td></tr>
<tr><td>11:15am</td><td>4:15pm</td></tr>
<tr><td>11:30am</td><td>4:30pm</td><td>Story time</td><td>Sing song: When you’re happy and you know it</td><td>Story Time</td></tr>
<tr><td>11:45am</td><td>4:45pm</td><td colspan="5">Goodbye circle and homework</td></tr>
<tr><td>12:00pm</td><td>5:00pm</td><td colspan="5">Finish</td></tr>
</table>

Who Am I
Week 2 Timetable

Time		Sunday	Monday	Tuesday	Wednesday	Thursday
**Morning**	**Afternoon**	**Activity**				
9:00am	2:00pm	National Anthem<br>Take off shoes				
9:15am	2:15pm	Welcome circle: Greetings and Orientation				
9:30am	2:30pm	Music Therapy: Welcome Song	Story Time	Music Therapy: Heads and Shoulders	Story Time	Music Therapy: Heads and Shoulders
9:45am	2:45pm	Individual Movement	Yoga	Individual Movement	Yoga	Individual Movement
10:00am	3:00pm	Group Movement Activities				
10:15am	3:15pm					
10:30am	3:30pm	**Morning/Afternoon tea and toilet break**				
10:45am	3:45pm					
11:00am	4:00pm	Class 6: Rulers and squares, preparation for classroom quilt	Class 7: Classroom quilt	Class 8: Body drawing	Sing Song: When you're happy and you know it	Class 10: My Story, personal information
11:15am	4:15pm				Class 9: Feelings faces	
11:30am	4:30pm					
11:45am	4:45pm	Goodbye circle and homework				
12:00pm	5:00pm	**Finish**				

Who am I
Week 3 Timetable

Time		Sunday	Monday	Tuesday	Wednesday	Thursday
**Morning**	**Afternoon**	**Activity**				
9:00am	2:00pm	National Anthem<br>Take off shoes				
9:15am	2:15pm	Welcome circle: Greetings and Orientation				
9:30am	2:30pm	Music Therapy: Welcome Song	Story Time	Music Therapy: Shake shake shake	Story Time	Music Therapy: When you're happy and you know it
9:45am	2:45pm	Yoga	Individual Movement	Yoga	Individual Movement	Yoga
10:00am	3:00pm	Group Movement Activities				
10:15am	3:15pm					
10:30am	3:30pm	**Morning/Afternoon tea and toilet break**				
10:45am	3:45pm					
11:00am	4:00pm	Class 11: Who am I?	Class 12: My daily routine	Class 13: Fingerprints	Class 14: 'Me' bag	Class 15: My Story, my favourite things
11:15am	4:15pm					
11:30am	4:30pm					
11:45am	4:45pm	Goodbye circle and homework				
12:00pm	5:00pm	**Finish**				

Who am I
Week 4 Timetable

Time		Sunday	Monday	Tuesday	Wednesday	Thursday
**Morning**	**Afternoon**	**Activity**				
9:00am	2:00pm	National Anthem<br>Take off shoes				
9:15am	2:15pm	Welcome circle: Greetings and Orientation				
9:30am	2:30pm	Music Therapy or Rhyme	Story Time	Music Therapy or Rhyme	Story Time	Music Therapy or Rhyme
9:45am	2:45pm	Individual Movement	Yoga	Individual Movement	Yoga	Individual Movement
10:00am	3:00pm	Group Movement Activities				
10:15am	3:15pm					
10:30am	3:30pm	**Morning/Afternoon tea and toilet break**				
10:45am	3:45pm					
11:00am	4:00pm	Class 16: What will I wear today?	Class 17: Getting ready to start my day	Class 18: Who am I? Feelings bingo	Class 19: 'Me 'shirt	Class 20: My Story, my favourite activities
11:15am	4:15pm					
11:30am	4:30pm					
11:45am	4:45pm	Goodbye circle and homework				
12:00pm	5:00pm	**Finish**				

My Family and Friends
Week 1 Timetable

Time		Sunday	Monday	Tuesday	Wednesday	Thursday
**Morning**	**Afternoon**	**Activity**				
9:00am	2:00pm	National Anthem<br>Take off shoes				
9:15am	2:15pm	Welcome circle: Greetings and Orientation				
9:30am	2:30pm	Music Therapy or Rhyme	Story Time	Music Therapy or Rhyme	Story Time	Music Therapy or Rhyme
9:45am	2:45pm	Yoga	Individual Movement	Yoga	Individual Movement	Yoga
10:00am	3:00pm	Group Movement Activities				
10:15am	3:15pm					
10:30am	3:30pm	**Morning/Afternoon tea and toilet break**				
10:45am	3:45pm					
11:00am	4:00pm	Class 21: My Story, who is in my family	Class 22: My Story, who am I in my family	Class 23: Rulers and squares, preparation for class quilt	Class 24: Classroom quilt, my family	Class 25: My Story, my family tree
11:15am	4:15pm					
11:30am	4:30pm					
11:45am	4:45pm	Goodbye circle and homework				
12:00pm	5:00pm	**Finish**				

My Family and Friends
Week 2 Timetable

Time		Sunday	Monday	Tuesday	Wednesday	Thursday
**Morning**	**Afternoon**	**Activity**				
9:00am	2:00pm	National Anthem<br>Take off shoes				
9:15am	2:15pm	Welcome circle: Greetings and Orientation				
9:30am	2:30pm	Story Time	Music Therapy or Rhyme	Story Time	Music Therapy or Rhyme	Story Time
9:45am	2:45pm	Individual Movement	Yoga	Individual Movement	Yoga	Individual Movement
10:00am	3:00pm	Group Movement Activities				
10:15am	3:15pm					
10:30am	3:30pm	**Morning/Afternoon tea and toilet break**				
10:45am	3:45pm					
11:00am	4:00pm	Class 26: Family flower	Class 27: My Story, how do I spend time with my family	Class 28: What will I wear today	Class 29: Getting ready to start my day	Class 30: Feelings and needs
11:15am	4:15pm					
11:30am	4:30pm					
11:45am	4:45pm	Goodbye circle and homework				
12:00pm	5:00pm	**Finish**				

My Family and Friends
Week 3 Timetable

Time		Sunday	Monday	Tuesday	Wednesday	Thursday
**Morning**	**Afternoon**	**Activity**				
9:00am	2:00pm	National Anthem<br>Take off shoes				
9:15am	2:15pm	Welcome circle: Greetings and Orientation				
9:30am	2:30pm	Music Therapy or Rhyme	Story Time	Music Therapy or Rhyme	Story Time	Music Therapy or Rhyme
9:45am	2:45pm	Yoga	Individual Movement	Yoga	Individual Movement	Yoga
10:00am	3:00pm	Group Movement Activities				
10:15am	3:15pm					
10:30am	3:30pm	**Morning/Afternoon tea and toilet break**				
10:45am	3:45pm					
11:00am	4:00pm	Class 31: Helping in the kitchen	Class 32: Role play, families	Class 33: Making nasta, Jhal Muri	Class 34: My Story, how do I spend time with my friends	Class 35: Friendship necklaces
11:15am	4:15pm					
11:30am	4:30pm					
11:45am	4:45pm	Goodbye circle and homework				
12:00pm	5:00pm	**Finish**				

My Family and Friends
Week 4 Timetable

Time		Sunday	Monday	Tuesday	Wednesday	Thursday
**Morning**	**Afternoon**	**Activity**				
9:00am	2:00pm	National Anthem<br>Take off shoes				
9:15am	2:15pm	Welcome circle: Greetings and Orientation				
9:30am	2:30pm	Story Time	Music Therapy or Rhyme	Story Time	Music Therapy or Rhyme	Story Time
9:45am	2:45pm	Individual Movement	Yoga	Individual Movement	Yoga	Individual Movement
10:00am	3:00pm	Group Movement Activities				
10:15am	3:15pm					
10:30am	3:30pm	**Morning/Afternoon tea and toilet break**				
10:45am	3:45pm					
11:00am	4:00pm	Class 36: Making a pig family and friends	Class 37: People in my life puzzle	Class 38: Write an invitation card	Class 39: My Story, my hero	Class 40: Family day
11:15am	4:15pm					
11:30am	4:30pm					
11:45am	4:45pm	Goodbye circle and homework				
12:00pm	5:00pm	**Finish**				

The World Around Me
Week 1 Timetable

Time		Sunday	Monday	Tuesday	Wednesday	Thursday
**Morning**	**Afternoon**	**Activity**				
9:00am	2:00pm	National Anthem<br>Take off shoes				
9:15am	2:15pm	Welcome circle: Greetings and Orientation				
9:30am	2:30pm	Music Therapy or Rhyme	Story Time	Music Therapy or Rhyme	Story Time	Music Therapy or Rhyme
9:45am	2:45pm	Yoga	Individual Movement	Yoga	Individual Movement	Yoga
10:00am	3:00pm	Group Movement Activities				
10:15am	3:15pm					
10:30am	3:30pm	**Morning/Afternoon tea and toilet break**				
10:45am	3:45pm					
11:00am	4:00pm	Class 41: Introduce theme, the world around me	Class 42: Alphabet and numbers	Class 43: What do I see at the fruit market	Class 44: Excursion to the fruit market	Class 55: My Story, my neighbourhood
11:15am	4:15pm					
11:30am	4:30pm					
11:45am	4:45pm	Goodbye circle and homework				
12:00pm	5:00pm	**Finish**				

The World Around Me
Week 2 Timetable

Time		Sunday	Monday	Tuesday	Wednesday	Thursday
**Morning**	**Afternoon**	**Activity**				
9:00am	2:00pm	National Anthem<br>Take off shoes				
9:15am	2:15pm	Welcome circle: Greetings and Orientation				
9:30am	2:30pm	Story Time	Music Therapy or Rhyme	Story Time	Music Therapy or Rhyme	Story Time
9:45am	2:45pm	Individual Movement	Yoga	Individual Movement	Yoga	Individual Movement
10:00am	3:00pm	Group Movement Activities				
10:15am	3:15pm					
10:30am	3:30pm	**Morning/Afternoon tea and toilet break**				
10:45am	3:45pm					
11:00am	4:00pm	Class 46: Alphabet and numbers	Class 47: Money	Class 48: What do I see at the vegetable market	Class 49: Excursion to the vegetable market	Class 50: Role play, market place
11:15am	4:15pm					
11:30am	4:30pm					
11:45am	4:45pm	Goodbye circle and homework				
12:00pm	5:00pm	**Finish**				

The World Around Me
Week 3 Timetable

Time		Sunday	Monday	Tuesday	Wednesday	Thursday
**Morning**	**Afternoon**	**Activity**				
9:00am	2:00pm	National Anthem<br>Take off shoes				
9:15am	2:15pm	Welcome circle: Greetings and Orientation				
9:30am	2:30pm	Music Therapy or Rhyme	Story Time	Music Therapy or Rhyme	Story Time	Music Therapy or Rhyme
9:45am	2:45pm	Yoga	Individual Movement	Yoga	Individual Movement	Yoga
10:00am	3:00pm	Group Movement Activities				
10:15am	3:15pm					
10:30am	3:30pm	**Morning/Afternoon tea and toilet break**				
10:45am	3:45pm					
11:00am	4:00pm	Class 51: Shopping	Class 52: Fruit salad	Class 53: What do I see at the clothes market	Class 54: Excursion to the clothes market	Class 55: Making fabric dolls
11:15am	4:15pm					
11:30am	4:30pm					
11:45am	4:45pm	Goodbye circle and homework				
12:00pm	5:00pm	**Finish**				

The World Around Me
Week 4 Timetable

Time		Sunday	Monday	Tuesday	Wednesday	Thursday
**Morning**	**Afternoon**	**Activity**				
9:00am	2:00pm	National Anthem<br>Take off shoes				
9:15am	2:15pm	Welcome circle: Greetings and Orientation				
9:30am	2:30pm	Story Time	Music Therapy or Rhyme	Story Time	Music Therapy or Rhyme	Story Time
9:45am	2:45pm	Individual Movement	Yoga	Individual Movement	Yoga	Individual Movement
10:00am	3:00pm	Group Movement Activities				
10:15am	3:15pm					
10:30am	3:30pm	**Morning/Afternoon tea and toilet break**				
10:45am	3:45pm					
11:00am	4:00pm	Class 56:<br>Role play, market place	Class 57: Shopping	Class 58: Vegetable salad	Class 59: Excursion to the field, I spy game	Class 60: My Story, my role in my community
11:15am	4:15pm					
11:30am	4:30pm					
11:45am	4:45pm	Goodbye circle and homework				
12:00pm	5:00pm	**Finish**				

School and Productivity
Week 1 Timetable

Time		Sunday	Monday	Tuesday	Wednesday	Thursday
**Morning**	**Afternoon**	**Activity**				
9:00am	2:00pm	National Anthem<br>Take off shoes				
9:15am	2:15pm	Welcome circle: Greetings and Orientation				
9:30am	2:30pm	Music Therapy or Rhyme	Story Time	Music Therapy or Rhyme	Story Time	Music Therapy or Rhyme
9:45am	2:45pm	Yoga	Individual Movement	Yoga	Individual Movement	**Morning/Afternoon tea and toilet break**
10:00am	3:00pm	Group Movement Activities				Class 65: Excursion to school
10:15am	3:15pm					
10:30am	3:30pm	**Morning/Afternoon tea and toilet break**				
10:45am	3:45pm					
11:00am	4:00pm	Class 61: My Story, school, helping others and work	Class 62: Alphabet and numbers	Class 63: Helping at home, getting dressed and ready for my day	Class 64: Science, make your own quicksand	
11:15am	4:15pm					
11:30am	4:30pm					
11:45am	4:45pm	Goodbye circle and homework				
12:00pm	5:00pm	**Finish**				

School and Productivity
Week 2 Timetable

<table>
<tr><th colspan="2">Time</th><th>Sunday</th><th>Monday</th><th>Tuesday</th><th>Wednesday</th><th>Thursday</th></tr>
<tr><th>Morning</th><th>Afternoon</th><th colspan="5">Activity</th></tr>
<tr><td>9:00am</td><td>2:00pm</td><td colspan="5">National Anthem
Take off shoes</td></tr>
<tr><td>9:15am</td><td>2:15pm</td><td colspan="5">Welcome circle: Greetings and Orientation</td></tr>
<tr><td>9:30am</td><td>2:30pm</td><td>Story Time</td><td>Music Therapy or Rhyme</td><td>Story Time</td><td>Music Therapy or Rhyme</td><td>Story Time</td></tr>
<tr><td>9:45am</td><td>2:45pm</td><td>Individual Movement</td><td>Morning/Afternoon tea and toilet break</td><td>Individual Movement</td><td>Yoga</td><td>Morning/Afternoon tea and toilet break</td></tr>
<tr><td>10:00am</td><td>3:00pm</td><td rowspan="2">Group Movement Activities</td><td rowspan="7">Class 67: Excursion to farm</td><td colspan="2" rowspan="2">Group Movement Activities</td><td rowspan="7">Class 70: Excursion to hand loom</td></tr>
<tr><td>10:15am</td><td>3:15pm</td></tr>
<tr><td>10:30am</td><td>3:30pm</td><td rowspan="2">Morning/Afternoon tea and toilet break</td><td colspan="2" rowspan="2">Morning/Afternoon tea and toilet break</td></tr>
<tr><td>10:45am</td><td>3:45pm</td></tr>
<tr><td>11:00am</td><td>4:00pm</td><td rowspan="3">Class 66: Words, sentences and maths</td><td rowspan="3">Class 68: Teacher says</td><td rowspan="3">Class 69: Science, make lemonade</td></tr>
<tr><td>11:15am</td><td>4:15pm</td></tr>
<tr><td>11:30am</td><td>4:30pm</td></tr>
<tr><td>11:45am</td><td>4:45pm</td><td colspan="5">Goodbye circle and homework</td></tr>
<tr><td>12:00pm</td><td>5:00pm</td><td colspan="5">Finish</td></tr>
</table>

School and Productivity
Week 3 Timetable

<table>
<tr><th colspan="2">Time</th><th>Sunday</th><th>Monday</th><th>Tuesday</th><th>Wednesday</th><th>Thursday</th></tr>
<tr><th>Morning</th><th>Afternoon</th><th colspan="5">Activity</th></tr>
<tr><td>9:00am</td><td>2:00pm</td><td colspan="5">National Anthem
Take off shoes</td></tr>
<tr><td>9:15am</td><td>2:15pm</td><td colspan="5">Welcome circle: Greetings and Orientation</td></tr>
<tr><td>9:30am</td><td>2:30pm</td><td>Music Therapy or Rhyme</td><td>Story Time</td><td>Music Therapy or Rhyme</td><td>Story Time</td><td>Music Therapy or Rhyme</td></tr>
<tr><td>9:45am</td><td>2:45pm</td><td>Yoga</td><td>Individual Movement</td><td>Yoga</td><td>Individual Movement</td><td>Morning/Afternoon tea and toilet break</td></tr>
<tr><td>10:00am</td><td>3:00pm</td><td colspan="4" rowspan="2">Group Movement Activities</td><td rowspan="7">Class 75: Excursion to a tailor</td></tr>
<tr><td>10:15am</td><td>3:15pm</td></tr>
<tr><td>10:30am</td><td>3:30pm</td><td colspan="4" rowspan="2">Morning/Afternoon tea and toilet break</td></tr>
<tr><td>10:45am</td><td>3:45pm</td></tr>
<tr><td>11:00am</td><td>4:00pm</td><td rowspan="3">Class 71: Words, sentences and maths</td><td rowspan="3">Class 72: Helping at home, washing dishes and wiping</td><td rowspan="3">Class 73: Science, make a volcano</td><td rowspan="3">Class 74: Teacher says</td></tr>
<tr><td>11:15am</td><td>4:15pm</td></tr>
<tr><td>11:30am</td><td>4:30pm</td></tr>
<tr><td>11:45am</td><td>4:45pm</td><td colspan="5">Goodbye circle and homework</td></tr>
<tr><td>12:00pm</td><td>5:00pm</td><td colspan="5">Finish</td></tr>
</table>

School and Productivity
Week 4 Timetable

Time		Sunday	Monday	Tuesday	Wednesday	Thursday
**Morning**	**Afternoon**	**Activity**				
9:00am	2:00pm	National Anthem<br>Take off shoes				
9:15am	2:15pm	Welcome circle: Greetings and Orientation				
9:30am	2:30pm	Story Time	Music Therapy or Rhyme	Story Time	Music Therapy or Rhyme	Story Time
9:45am	2:45pm	Individual Movement	Yoga	Individual Movement	Yoga	Individual Movement
10:00am	3:00pm	Group Movement Activities				
10:15am	3:15pm					
10:30am	3:30pm	**Morning/Afternoon tea and toilet break**				
10:45am	3:45pm					
11:00am	4:00pm	Class 76: Invitation to you graduation ceremony	Class 77: Making graduation nasta, Jhal Muri	Class 78: Dress up day, what do I want to be when I grow up	Class 79: Making a graduation hat	Class 80: Graduation ceremony
11:15am	4:15pm					
11:30am	4:30pm					
11:45am	4:45pm	Goodbye circle and homework				
12:00pm	5:00pm	**Finish**				

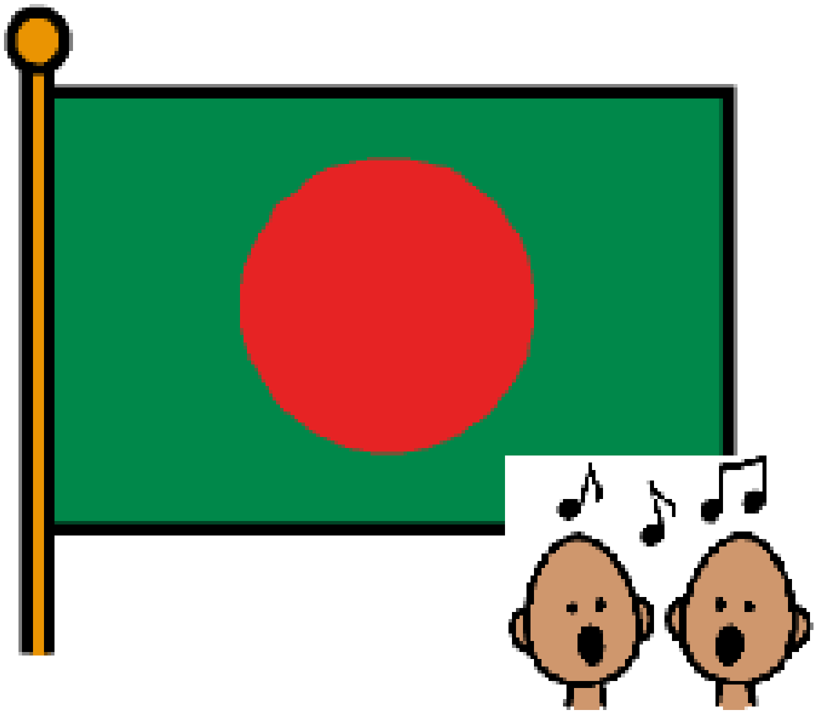

জাতীয় সঙ্গীত

National Anthem

Helps develop these Mental Skills:

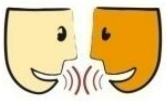
Language and communication

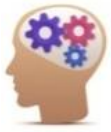
Cognition

Helps develop these Functional Skills:

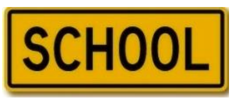

School readiness

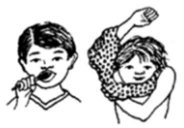
Activities of daily living

Bangladesh National Anthem

To develop:

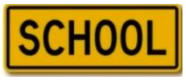

School readiness

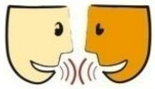
Language and communication

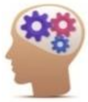
Cognition: Memory

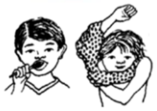
Activities of daily living: Removing shoes

Need:

- Bangladesh National Flag
- Outdoor handrail
- Special seat for those children unable to stand with handrail
- Drum and/or Tamborine

Instructions:

1. Move outside the classroom, next to the flag and beside the handrail.
2. Children who are comfortably able, stand holding onto the handrail.
3. Children unable to stand, sit in special seat.
4. Sing the national anthem.
5. For children who are unable to verbally communicate and sing, give them a musical instrument such as a drum or tamborine to play in time with the beat of the anthem. This can also be used to help develop upper limb skills.
6. After the anthem is finished, instruct children to take off their shoes and move back inside the classroom. Encourage each child to take off their shoes as independently as possible. Remember:

The key points to helping a child gain independence in activities of daily living are:

- Be patient, give the child TIME to think through and attempt/complete steps of the task themself.
- Recognise and acknowledge a child's attempts and ability to complete steps of the task independently.
- Do NOT take over and do the task for them. If the task is too difficult for them, think why is it too difficult? Try and make it a little less difficult for the child. FACILITATE ability.
- Provide prompts only if the child is not initiating or skips a step of the task, i.e. only provide a prompt if it is needed, and do not continually prompt the child through a task. Provide a prompt and then give the child time to think and do.
- Only provide assistance to facilitate ability, and make the step of the task achievable for the child. Make sure that you don't provide so much assistance that the child is not actively participating. Ensure that the step of the task is still a challenge, but provide assistance so that it is achievable for the child. The child needs to do AS MUCH as they can themselves, and ALWAYS actively participate.

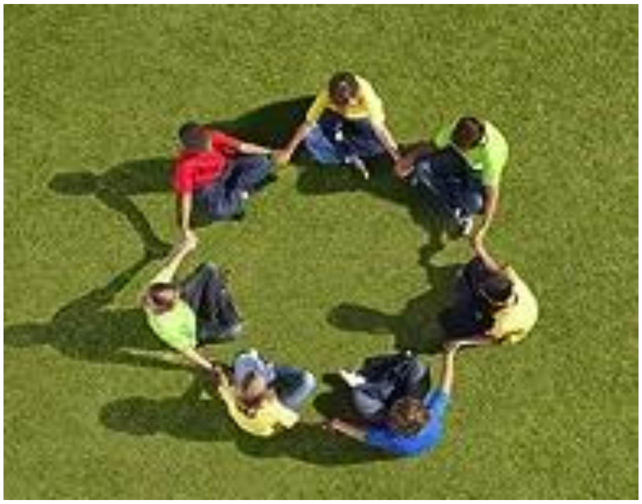

Welcome Circle

Helps develop these skills:

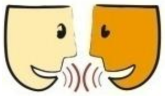 Language and communication

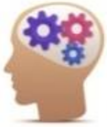 Cognition

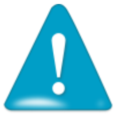 Attention

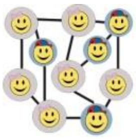 Social behaviour

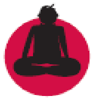 Sitting

Activities:

1. Vocal warm-Up
2. Greeting
3. Orientation

Tools Required:

- Foam floor mats
- Special floor chair or cushions to help children sit independently

Instructions:

1. Children to sit in a circle. Use the special seats or cushions to help children sit independently.

Welcome circle: Vocal Warm-Up

To develop:

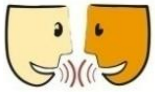

Language and Communication: Vocal control

Need:

- Tissues
- Bangla alphabet poster/books for all children to see

Instructions:

1. Each day, choose three vowel sounds and three consonant sounds to practice. Show and explain to the children how to make the sound to help them learn, and so that children who are deaf get very visible differences in sounds, and children with cerebral palsy have a chance to exercise their tongues and breath. For example, labial sound "B", nasal "MMM", with your teeth on the lip "V", with your tongue behind the teeth "L".
2. Choose a sound game to play with the children:
   a. Choose a sound and ask children to make the sound as long as possible, the child who can make the sound the longest is the winner.
   b. Choose a sound and ask the children to make the sound as loud as possible, the child who can make the sound the loudest is the winner.
   c. Put a tissue in front of each child. Instruct the children to blow the tissue as far away as they can, without moving their bottom. The child who can blow the tissue the furthest is the winner.
   d. Get children to say as many different words as they can that start with or end with that sound.

Welcome circle: Greeting

To develop:

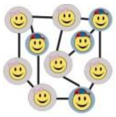

Social behaviour: introductions and greetings

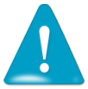

Attention: awareness of activities for the day and length of time

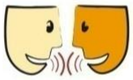

Language and communication

Need:

- Good Morning Song Sheet
  OR
  Shishu Shorgo Gan CD, CD player and Music Therapy Manual
- Attendance record
- Activity cards
- Blue-tack
- Whiteboard
- Augmentative and Alternative Communication tools. Refer to Appendix: Communication Resources for details.

Instructions:

1. Welcome the children to the group for today.
2. Choose 1 song from the Good Morning Song Sheet or play track 1 “Aslamalykum” from the Shishu Shorgo Gan CD.
3. Sing the song together. Include sign language and actions e.g. waving hello to each other, particularly so that children unable to verbally communicate are included and all children learn sign language to support their verbal communication.
4. Ask the children to welcome each other and ask how they are today. Use communication boards and simple signs to support verbal communication.
5. Teacher to complete the attendance record. Children to respond when their name is called to indicate they are here. Get the children to count how many children are at Shishu Shorgo that day.
6. Introduce the activity timetable for the day. Say and stick the activities in order on the board. Get children to assist with putting the activity cards on the board.
7. Stick the picture card for ‘now’ next to the first activity.
8. Refer back to the board after finishing each activity to introduce the next activity. Ask the children, “What does the timetable say we will do next?” Move the picture card for ‘now’ as you move to the next activity.

Good Morning Song Sheet

Song 1:
(to the tune of "Happy Birthday")
Good Morning to you
Good Morning to you
Good Morning Dear class
Good Morning to you!

Song 2:
School is waiting
Can't be late.
Hurry! Hurry!
Half-past eight.
Out the door
And down the street,
Then softly, quietly,
Take your seat.

Song 3:
(to the tune of Brownie Smile)
I awoke this morning, ate my breakfast early.
I dressed by myself and to school I hurry.
School, school, here I am today.
Now I am ready to read and sing and play.

Song 4:
Hello Neighbor, what do you say?
(Children bow to each other)
It's going to be a happy day.
(Children clap hands together)
So greet your neighbor,
(Children give each other high fives)
And boogie on down.
(Children wiggle their hips)
Give 'em a bump,
(Children bump their hips together)
and turn around.
(Children turn around)

Song 5:
(tune of If You're Happy and You Know It)
Good morning, (first name and last name).
How are you?
Good morning, (first name and last name).
How are you?
How are you this special day?
We're glad you came to play.
Good morning, (first name and last name).
How are you?

Song 6:
Oh, the duck says "Quack",
and the cow says "Moo",
the old red rooster says
"Cock-a-doodle-doo".
The sheep says "Baa",
and the cat says "Meow",
but I say "Good Morning
when I see you!

Song 7:
Good morning, good morning!
School has begun.
Good morning, good morning!
Isn't it fun!

Pencils and crayons
Scissors and glue
Erasers and paper
Reading books, too.

Good morning, good morning!
School has begun.
Good morning, good morning!
Isn't it fun!

Class 2: Orientation

To develop:

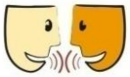

Language and communication: Verbal, visual and written communication to describe the time and weather

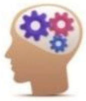

Cognition: Memory and awareness of time and weather

Need:

- Board with the following questions:
    - *What is the day today?*
    - *What month is it?*
    - *What is the date today?*
    - *What year is it?*
    - *What is the season?*
    - *What is the weather like today?*
- Laminated word cards with the days of the week, and different months
- Three sets of laminated number cards with the numbers 1-9
- Laminated picture cards showing different weather conditions and seasons with the words written below the picture
- **Orientation Communication Board.** Refer to Appendix: Communication Resources for details.
- Blue-tack

Instructions:

1. Bring the children's attention to the orientation board.
2. Ask one question at a time*
   "What is the day today?"
   "What is the date today?"
   "What month is it?"
   "What year is it?"
   "What is the weather like today?"

3. After each question, select each child to respond one at a time. Select one child for each question to pick and put the corresponding card on the board

Difficulty Level	Child asked to pick...		Child to pick the card from...
Easy	Picture cards (Easiest)	And given...	Two options
Moderate	Number cards		Three options
Hard	Word cards		Whole pile (Hardest)

* After asking each question, ask each child to respond one at a time. Revise Class 2: Orientation, as required. For those children unable to communicate verbally, give them a communication board to help them answer. After asking and answering each question, select one child to pick and put the corresponding picture card on the board. Match the level of difficulty to the abilities of each child. Ensure the child is being challenged, but able to succeed with time, prompting or minimal assistance. Use this table below to help decide what type of card and how many options to give each child.

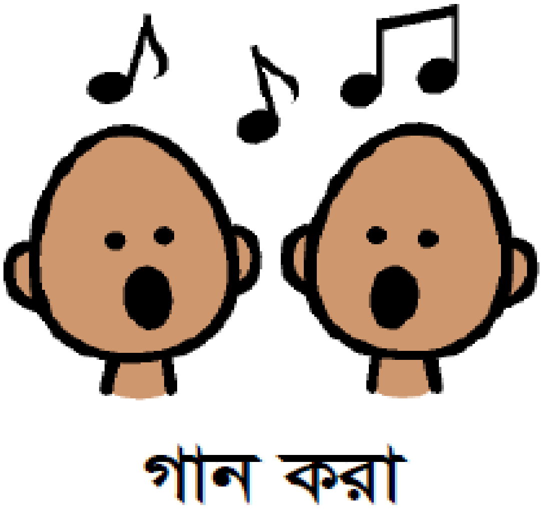

Music Therapy & Rhymes

Helps to develop these Mental skills:

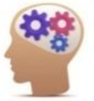 Cognition

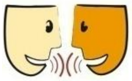 Language and communication

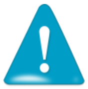 Attention

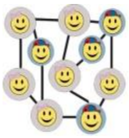 Social behaviour

Helps to develop these Physical skills:

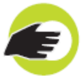 Arm and hand control

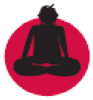 Sitting

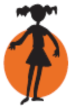 Standing

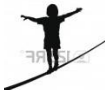 Balance

Tools Required:

- Foam floor mats
- Special floor chair or cushions for supported sitting
- Music Therapy Manual
- Music Therapy, “Shishu Sorgo Gan” CD
- CD player
- Rhyme Sheet

Instructions:

1. Children to sit in a circle.
2. Use the special seats or cushions to support children in sitting.

Music Therapy & Rhymes

To develop:

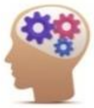
Cognition: Memory, following commands

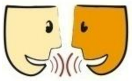
Language and communication: Vocal control, vocabulary

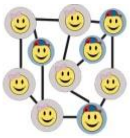
Social behaviour: Greetings, turn taking, social awareness

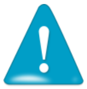
Attention: Focus and participation

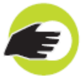
Arm and hand control and coordination

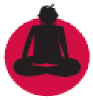 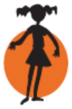
Sitting, Standing

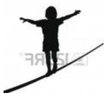
Balance

Need:

- **Music Therapy Manual**
- Music Therapy, 'Shishu Sorgo Gan' CD
- CD player
- Other items as listed in the Music Therapy Manual for each song
  OR
- **Rhyme Sheet**

Instructions:

1. Select one song from the **Music Therapy Manual** for the session. Follow the instructions in the music therapy manual.
   OR
   Select one rhyme from the **Rhyme Sheet**.
2. Repeat the song or rhyme three times each session.

You can repeat a song or rhyme on other days, but make sure you regularly introduce a new song or rhyme, e.g. sing two different songs each week.

Rhyme Sheet

To find song/rhyme type into google search 'Bangla children's song' or 'Bangla children's rhyme'. A selection of songs/rhymes will come up. If you are looking for a song that matches a particular theme e.g. brushing your teeth, type 'Children's song brushing teeth' into Google search. It may come up with English songs or rhymes which you could translate into Bangla. Choose those songs/rhymes that you think the children can learn from and would enjoy.

Here are some songs/rhymes that you could use:

Rhyme 1: Nature
Chaand utheche phool phooteche
Kodom tolaay key ?
Haathi naachchhe ghoda nachchhe
Sona monir be' (biye)

Rhyme 2: Don't cry
Aatul baatul shyamla shatul
Shyamla geche haate
Shymlader dui chhele
Pothe bosey kaande
Aar kendo na aar kendo na
Chhola bhaja debo
Ebar jodi kaando tobey
Tooley aachhaar debo.

Rhyme 3: Fantasy creature
Haattimatim tim
Taader khaada duto sing
Taara maathey paarey dim
Taara haattimatim tim.

Video Links

Selection of Bangla children's songs:
http://www.youtube.com/watch?v=mRU_rM8zhjQ

Bangla alphabet song:
http://www.dailymotion.com/video/xjdkdj_bengali-nursery-rhyme-alphabet_school

Brushing teeth song (in English):
http://www.bbc.co.uk/schoolradio/subjects/earlylearning/nurserysongs/A-E/brush_your_teeth

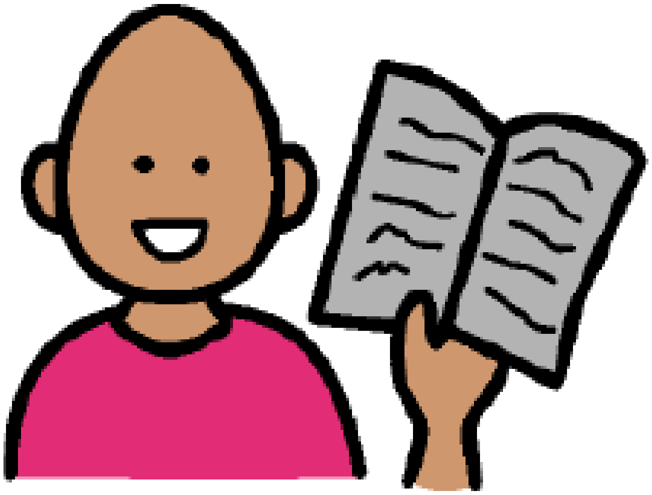

গল্প

Story Time

Helps develop these Mental Skills:

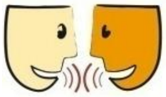 Language and communication

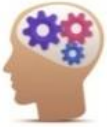 Cognition

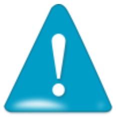 Attention

Helps develop these Physical Skills:

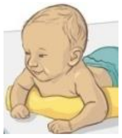 Head and trunk control

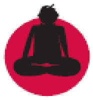 Sitting

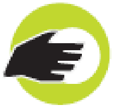 Arm and hand control

Tools Required:

- Foam floor mats
- Special floor chair or cushions for supported sitting

Instructions:

1. Children to sit in a circle.
2. Use the special seats or cushions to support children in sitting.

Story and Questions

To develop:

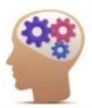

Cognition: Recalling and sequencing a story, letters, words

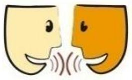

Language and communication: Understanding a story, making sentences

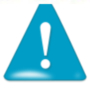

Attention: Listening quietly

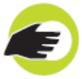

Arm and hand control: Pointing, picking up and putting down, drawing, writing letters, words and sentences

Need:

- Children's story
- Foam floor mats
- Supported chairs or cushions to help children sit independently
- Paper and pencils
- Augmentative and Alternative Communication tools. Refer to Appendix: Communication Resources for details.

Instructions:

1. Select a child to get a story book from the bookcase. This is an opportunity for the child to practice moving, reaching and grasping.
2. Select a child to choose a story from the story book.
3. Show the children the front of the book and introduce the name and author of the story.
4. Read the story. As you read the story:
    a. Point to and talk about the pictures in the story. Ask the children to name/describe different characters on the front page e.g. a small girl, a tall boy, a yellow lion, the tiger is smaller than the lion.
    b. Include actions for children to copy e.g. waving arms to show how a bird flies.
    c. Use sign language and communication boards to help explain the story. This will help children with hearing impairment understand. This also provides children with difficulty talking an opportunity to practice using a communication board.

After the story is finished:

5. Ask the children a few questions about the story. Try and ask questions relating to the theme of the month. To grade the questions for each child, use the table below as a guide:

All children	Use pictures in the story to ask questions that require a one word answer or pointing. E.g. which is the biggest/smallest, what colour is the person's top, what is this person's name?<br><br>If the child does not initiate an answer, prompt by giving two choices for the answer.<br><br>E.g. Put a picture from the story in front of the child and ask them to point to the bird. If no response, ask, is this a bird (and point to a bird) or is this a bird (and point to another subject in the picture).
If the child finds one word answer questions easy….	Ask simple questions (with one or two word answers) about the story. E.g. Who did Priya play with in the paddy fields? What animals lived on the farm?<br><br>For children who have difficulty talking, use communication boards. Provide children a set of picture cards for a matching activity. E.g. if there are animals in the story, provide children with picture cards and match the animal with that in the story.
If the child finds simple questions easy…	Ask children to retell the story (as much of the story as they can remember). Provide prompts if the child gets stuck, e.g. what happened after Priya milked the cow?<br><br>Ask thinking questions. E.g. Do you think Priya was happy? Why?<br><br>Ask questions that connect the stories to their experiences and feelings. E.g. Who is your best friend? What do you love to do together? Would you like to play in a paddy field? Why?

6. Photocopy pictures from the story and ask the children to put pictures in sequence. Use sign language and communication boards.

7. Ask children to retell the story by:
    a. Drawing the story
    b. Get children to assume a different character of the story and act it out.

8. Photocopy a picture in the story. Get the children to practice ripping/using scissors to make a puzzle. Grade the task according to the child's abilities:

Easy	Ask the child to rip the picture in half. Then put the pieces together.
Medium	Draw three clear parallel lines on the picture. Ask the child to use scissors to cut the picture in three straight pieces, following the lines. Then ask the child to put the pieces together.
Hard	Draw clear curvy lines on the picture. Ask the child to use scissors to cut the picture following the lines. Then ask the child to put the pieces together.

9. Develop the children's literacy. To grade the activity for each child, use the table below as a guide:

All children	Learn letters. Each week choose 10 letters for the children to learn. Write the letter on the board. Get children to identify different names/words that start with, contain or end with that letter. Get the children to find that letter in the story or in their alphabet books. Get the children to practice writing and saying the letter. Get the children to say words that start with or end with that letter.<br><br>Teach the children simple sign language and how to use a simple communication board for basic needs and instructions e.g. go, stop, finish, more, toilet, drink, food.
If the child learns letters easily…	From the word list, choose 10 words each week for the children to learn. Get the children to practice finding the word in a sentence, writing the word, saying the word alone and in a sentence, and if applicable putting an action to that word.<br><br>Teach the children more complex sign language and communication boards for them to respond in a timely manner and ask questions.
If the child learns words easily…	Learn sentences from the story. Practice reading the sentence and writing the sentence. Use alphabet and word cards to allow children who find it hard to write to form words and sentences.<br><br>Teach the children more complex sign language and communication boards to have a simple conversation.

Word List

Month 1: Who am I?

Mother	Toilet	Like	Hello
Father	Eat	Hate	Goodbye
Brother	Drink	Yes	Home
Sister	Want	No	Market
Play	Need	More	School

Month 2: My Family and Friends

Grandma	Go	Rickshaw	Big
Grandpa	Come	Cricket	Small
Auntie	Walk	Ball	Some
Uncle	Shop	Badminton	Many
Friend	Talk	Help	Old
Neighbour	Stop	Sick	Young

Month 3: The World Around Me

Restaurant	Rice	Eggplant	Red
Market	Egg	Fruit	Yellow
Shopkeeper	Chicken	Mango	Purple
Rickshaw walla	Beef	Lychee	Orange
Doctor	Mutton	Banana	Blue
Water	Vegetables	Ripe	Black
Tea	Potato	Rotten	Pink
Coconut	Beans	Colour	Green

Month 4: School and Productivity

Learn	Teacher	Desk	Science
Write	Students	Chair	Maths
Read	Pencil	Sit	English
Listen	Paper	Stand	Bangla
Look	Notebook	Uniform	Together

Classes

Month 1: Who am I

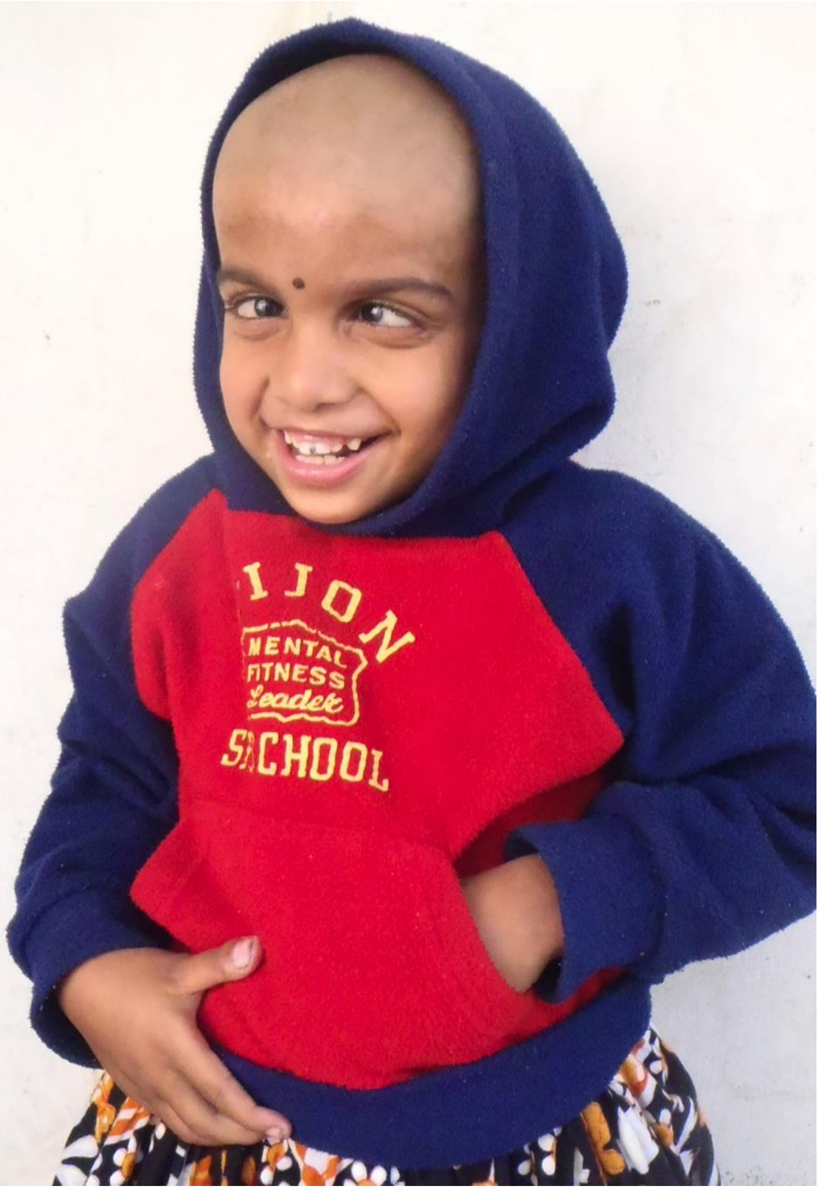

Class 1: My Name

To develop:

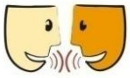

Language and communication: Reading and writing own name

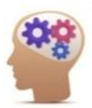

Cognition: Name and letter recognition, letter construction

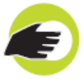

Arm and hand control: Pinching and rolling, ripping, gluing

Need:

- Laminated name cards
- Play-dough (ball for each child)
- Bangla alphabet card. Refer to Appendix: Communication Resources for details.
- A4 pieces of plain white paper, (1 piece for each student)
- Marker
- Scrap pieces of material, coloured paper/magazines that can be used to make a collage
- Glue

Instructions:

1. Ask every person in the room to say their name one at a time.
2. Ask the children, who gives us our name?
3. Explain to the children, most names have meanings. Ask the children one at a time, what does your name mean? The child's mother can help explain it. Draw pictures and use actions to help explain the meaning of their name if the child has difficulty understanding speech.
4. Put each child's name card in front of them. Ask them, what does this say? Say it's their name. Point to the different letters that make their name, and make the different sound for each letter. Practice with the child.
5. Give each child a ball of play-dough. Write your name on the whiteboard. Point to the first letter and demonstrate how to make the first letter of your name. Ask the children to point to the first letter of their name on the name card in front of them. Instruct the children to make the first letter of their name using play-dough.
6. Show the children a Bangla alphabet board. Ask each child to find and point to the first letter of their name. They can look at their name card to help.
7. Draw a large outline of the first letter of their name on a piece of A4 paper, and write the following letters of their name normally beside it. Instruct the children to fill in the first letter outline by ripping and gluing pieces of coloured paper or pieces of material in the space. This is called a collage.
8. Once finished, get the children to show their collage to the rest of the class. Get all children to say that child's name and the sound the first letter of their name makes. Put the finished product on the wall.

Class 2: Orientation

To develop:

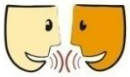

Language and communication: Verbal, visual and written communication to describe the time and weather

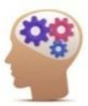

Cognition: Memory and awareness of time and weather

Need:

- Board with the following questions:
    - *What is the day today?*
    - *What month is it?*
    - *What is the date today?*
    - *What year is it?*
    - *What is the season?*
    - *What is the weather like today?*
- Laminated word cards with the days of the week, and different months
- Three sets of laminated number cards with the numbers 1-9
- Laminated picture cards showing different weather conditions and seasons with the words written below the picture
- **Orientation Communication Board.** Refer to Appendix: Communication Resources for details.
- Blue-tack

Instructions:

1. Ask children how many days there are in a week. Name the days in the week. Ask the children what days they come to the centre and what days they stay home. Ask the first question on the orientation board: *"What is the day today?"* *
2. Ask children how many months in a year. Name the months. Ask the second question on the orientation board: *"What month is it?"* *
3. Explain to the children that within every month a number is given to each day. This is called the date. Tell the children, the date yesterday was_____. Ask the third question on the orientation board: *"What is the date today?"* *
4. Explain to the children that after 12 months, a new year starts. The year is given a number. Ask the fourth question on the orientation board: *"What year is it?"* *
5. Ask children what different seasons there are in a year. Talk about each season and what happens during each season. Then ask the fifth question on the orientation board: *"What is the season?"* *
6. Talk about the weather. Take all the children outside. Ask the children do you feel hot or cold? What does the sky look like? Give children a piece of chalk each and get them to draw on the concrete what they see in the sky. Ask each child to describe what they are drawing. Go back inside and show them the picture cards of different weather conditions. Ask them the sixth question: *"What is the weather like today?"* *

* After asking each question, ask each child to respond one at a time. For those children unable to communicate verbally, give them a communication board to help them answer. After asking and answering each question, select one child to pick and put the corresponding picture card on the board. Match the level of difficulty to the abilities of each child. Ensure the child is being challenged, but able to succeed with time, prompting or minimal assistance. Use this table below to help decide what type of card and how many options to give each child.

Difficulty Level	Child asked to pick...		Child to pick the card from...
Easy	Picture cards (Easiest)	And given...	Two options
Moderate	Number cards		Three options
Hard	Word cards		Whole pile (Hardest)

Class 3: Feelings

To develop:

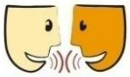

Language and communication: Express feelings verbally and visually

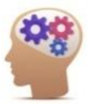

Cognition: Self awareness, matching

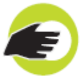

Arm and hand control: Pencil grip and control, drawing

Need:

- Pictures of different people's faces cut out from a magazine or newspaper
- 2 sets of **"I feel" Picture Cards** or **Feelings Symbols.** Refer to Appendix: Communication Resources for details.
- Paper plates
- Coloured pencils, crayons or markers
- Approx 20cm long sticks
- Sticky tape

Instructions:

1. Ask the children, how do you show when you are happy? How do you show you are sad? How do you show you are angry? How do you show you are hurting? How do you show you are tired? Encourage children to make different facial expressions and movements.
2. Show pictures of different people's faces one at a time. Ask the children, are they happy, sad, angry or hurting. Talk about what makes them look sad, happy, etc.
3. Show the expression picture cards one at a time. Ask the children what does this face show?
4. Ask each child to point to point the picture that shows how they are feeling today. Encourage the children to say "today I am feeling........"
5. Play a matching game. Put four pictures in front of each child: Two of the same cards and two different cards. Ask the children to select the cards that are the same. You can make this harder by putting 6 pictures in front of the child: four of the same cards and two different.
6. Give each child a paper plate and ask them to draw a face showing a feeling. They can look closely at the pictures showing that feeling e.g. happiness, to help them.
7. Once the drawing is finished, attach long sticks to the back of the plate with sticky tape.
8. Using the plates like puppets, get the children to ask each other, how are you feeling today?

Class 4: My Shadow

To develop:

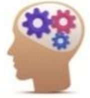

Cognition: Awareness of body, parts and functions

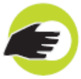

Arm and hand control: Pincer grip and drawing

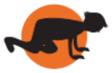

Crawling

Need:

- Chalk
- Name cards
- Body parts label cards

Instructions:

1. Explain what a shadow is. Ask the children, where have you seen your shadow?
2. Go outside and try and find your shadow. Get the children to find their shadow. Tell them to move their hand and watch how their shadow moves. Tell them to move their leg and watch how the shadow moves etc.
3. Instruct the children to trace your shadow. If you find your shadow on the concrete: give the children chalk to draw around the shadow on the concrete. If you find your shadow on soft dirt ground: give the children sticks to draw the outline in the dirt. If you find your shadow on a grass field or hard dirt ground: give the children sticks or twigs to place on the ground around your shadow.
4. Return to Shishu Shorgo and instruct the children to get in groups of two. One child to lie on the concrete and the second child to draw around their body with chalk. Then the children swap, so that all children have a traced outline of their body on the concrete.
5. Instruct children to identify and draw their own features on their traced body – eyes, eyebrows, nose, ears, hair, fingers, and clothing.
6. Give each child a set of body parts cards. Instruct the children to put the card with a picture and word on their body trace to label the parts of their body.
7. Get the children their name card and instruct the child to copy their name onto the concrete below the picture using the chalk. If this is too difficult, tell them to draw the first letter of their name using chalk, or make it with leaves/sticks etc.
8. Once finished, instruct the children to look at each other's body trace and identify the different body parts.

Class 5: My Story, My Portrait

To develop:

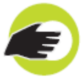

Arm and hand control: Ripping and gluing, cutting with scissors, pencil grip and control, drawing

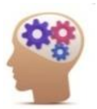

Cognition: Recognition of facial features

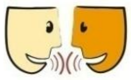

Language and communication: Read and write own name, describe characteristics about themselves and others

Need:

- Blank drawing books (1 book for each child)
- Colouring-in pencils, and/or crayons
- Scissors
- Mirror
- Portraits of famous people printed from the internet or cut out from magazines or newspapers

Instructions:

1. Explain to the children that each child will make their own story in their book. Each week we will add something new to their story.
2. Show children a variety of famous self-portraits printed from the internet or cut out from magazines or newspapers. Talk about the characteristics of the pictures. E.g. that a portrait is a picture of a person's face. On their face they have two eyes, one nose, one mouth, two ears, two eyebrows, and hair.
3. Draw a large oval on the first page of each child's drawing book. This will be the template for their portrait.
4. Give children a mirror to look into. Ask them, what is the colour of your hair? Get them to draw their hair in that colour on the template. Ask them, what colour are your eyes? Get the children to draw two eyes in that colour. Etc.
5. Instruct children to use the scissors and cut single straight lines at the top of the page to create a fringe and make their hair come out of the page.
6. Give each child their name card. Instruct them to copy their name onto their portrait. If that is too difficult, help them write the first letter of their name.
7. Once finished, instruct children to show their portrait to the rest of the class and describe the features. Prompt them by asking questions: Who is this portrait of? How many eyes? What colour eyes? Where are the lips? Where is the hair? What colour is the hair? Etc.

Class 6: Rulers and Squares, Preparation for Classroom Quilt

To develop:

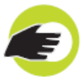

Arm and hand control: Pencil grip and control, drawing and cutting straight lines

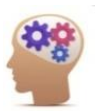

Cognition: Recognition of shapes, understanding ruler use

Need:

- A4 pieces of white paper (1 piece for each child)
- A4 pieces of coloured paper (1 piece for each child)
- Rulers (1 for each child)
- Scissors (1 for each child)
- Pencils (1 for each child)
- A rectangle block and a square block

Instructions:

1. Give each child a ruler. Pick up a ruler and lead a group discussion about the ruler: Ask the children what is this? What shape is it? Does it have straight edges or curved? Is the surface flat or bumpy? What is written on the ruler? Are the numbers Bangla or English?
2. Explain to the children there are numbers written on the ruler, and the numbers are from 1 to 30. We can use this to measure the length of things. Because it has a flat surface, you can easily measure something on a flat surface, e.g. a piece of paper.
3. Give each child a piece of white paper and a piece of coloured paper.
4. Instruct each child to trace around their hand on one side of their piece of white paper. Provide assistance as needed.
5. Show children how they can measure the length of each finger. Encourage the child to do as much as they can themselves – placing the ruler next to the trace of each finger, and prompt the child to position the ruler with 0 at the base of the finger, and read the number beside the tip of each finer.
6. Explain to the children that because a ruler has straight edges, you can also use it to draw straight lines. If you want to draw a straight line a certain length, you can do this using a ruler. This is what we will do next to make squares from these pieces of paper. Show the children two blocks – one rectangle and one square. Talk about each shape. Which sides of the rectangle are the same lengths? Which are different? Which sides of the square are the same lengths? Which are different? Select a few children to check by using the ruler.
7. Instruct the children to turn their white piece of paper over. Tell them that now we are going to make a white square with four sides that are all 15 cm long. Demonstrate how you draw and then cut out a square. Encourage each child to do as much as possible themselves. Provide prompts and assistance as required.
8. Instruct the children to make a square out of their coloured piece of paper with four sides 18 cm long.
9. Children to show each other their finished squares.
10. Keep the squares to use tomorrow for the classroom quilt.

Class 7: Classroom Quilt

To develop:

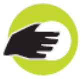

Arm and hand control: Pencil grip and control, drawing, writing, gluing, taping

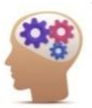

Cognition: Recalling special events

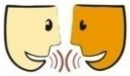

Language and communication: Describing special events

Need:

- Squares of white paper (one piece for each child)
- Squares of coloured paper (one piece for each child)
- Coloured pencils/crayons
- Glue
- Sticky tape
- Laminated name cards

Instructions:

1. Brainstorm with children a list of special events. E.g. birthday, a holiday, a sports competition, a festival, a wedding. Write these on the whiteboard for the children to see.
2. Provide each child with a piece of white square paper.
3. Instruct each child to draw and/or write about a special event on their white square.
4. Instruct each child to centre and paste the completed white square on a coloured square and write their name on the bottom of the square. Use the laminated name cards for the child to copy if needed.
5. When all the children have completed their square, have the children tape the squares into rows to form a quilt pattern.
6. To finish the quilt, have a few children cut out and tape a white border around the quilt. Children can decorate the border any way they wish.
7. Display the quilt and have each child talk about their own special event to the rest of the group.

Class 8: Body drawing

To develop:

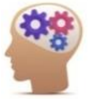

Cognition: Awareness of body parts and functions

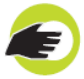

Arm and hand control: Pencil grasp and drawing

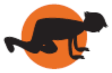

Crawling

Need:

- Long lengths of paper, one for each person
- Marker
- Scraps of material, coloured paper
- Crayons
- Name cards
- Body parts label cards

Instructions:

1. Put children in pairs.
2. First child to lie on a long length of paper. Second child to draw with a pencil or marker around the first child's body.
3. Swap, so that each child has an outline of their body on paper.
4. Each child to then identify and draw their own features on their traced body – eyes, eyebrows, nose, ears, hair, fingers, clothing. The child can stick real pieces of fabric, different coloured paper on their body. The child can also label different parts of their body.
5. Assist the child to write their name below their body. If unable to write their name independently, get the child to identify their laminated name card and instruct the child to copy their name onto the picture.
6. Hang the finished drawings around the room.
7. As a group, move around the room to look at each child's body drawing. Ask the group, who is this drawing of? What can you see? Where are the eyes, how many? Where are the hands? Where are the feet? What do the feet do? Etc.

Class 9: Feelings Faces

To develop:

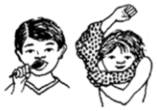

Activities of daily living: Basic food preparation and eating

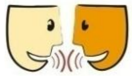

Language and communication: Expressing feelings

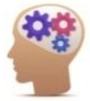

Cognition: Recognition of facial features, construction

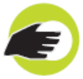

Arm and hand control: Grasp and release

Need:

- **"I feel" Picture Cards** or **Feelings Symbols**. Refer to Appendix: Communication Resources for details.
- Plate or serviette or piece of newspaper (to use as a plate) for each child
- Hat ruti (1 for each child)
- Mayonnaise
- Egg cut in rings
- Apple cut in wedges
- Tomato cut in wedges
- Cucumber cut in sticks
- Blunt butter knife (for spreading mayonnaise)

Instructions:

1. Ask the children, how do you show when you are happy? How do you show you are sad? How do you show you are angry? How do you show you are hurting? How do you show you are tired? Encourage children to make different facial expressions and movements.
2. Instruct children in the class to ask each other "how are you feeling today?"
3. Put the ingredients on plates in the middle of the circle.
4. Give each child a picture card with a facial expression showing a feeling. Get each child to show their picture card to the rest of the group and say or use actions to show what that feeling is.
5. Explain to the children that today you will be making faces expressing the feeling on your picture card using these ingredients in front of you. At the end you will get to eat your face! But not until you have finished making your face and showing the group.
6. Write the steps on the board and explain each step to the group. Step 1: take one ruti. Step 2: spread mayonnaise over the ruti to make the skin. Step 3: Make the eyes with eyebrows to show your feeling. Step 4: Make the mouth to show your feeling. Step 5: Add a nose. Step 6: Add ears. Step 7: Add hair.
7. Guide the children through each step. Encourage them to use different ingredients to make the different facial features.
8. Once finished, get each child to show the group and explain the feeling shown on the ruti face.

9. Eat the face for nasta! Encourage the child to eat independently.

Class 10: My Story, Personal Information

To develop:

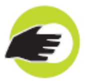

Arm and hand control: Ripping and gluing, cutting with scissors, pencil grip and control, drawing

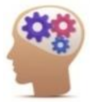

Cognition: Recognition of facial features

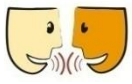

Language and communication: Read and write own name, describe characteristics about themselves and others

Need:

- Laminated name cards
- Bangla and English number cards
- Tape measure
- Ball of string
- Scissors
- My Story book
- Pencils (1 for each person)
- Piece of paper (1 for each person)
- Blue-tack

Instructions:

1. Write these headings on the whiteboard: Name:, age:, birthday:, height:, address:
2. Instruct the children able to copy words to write these headings in their book. For those children unable to write sentences, ask the adults in the room to copy these headings into the child's book.
3. Children to identify their laminated name card and copy their name into their book next to the heading 'name:'
4. Children to say or sign their age, identify the number from the number cards in Bangla and in English, and write the number next to the heading 'age:'
5. Using a tape measure, measure the height of the child. Get another child to read the number on the tape measure. Children to write their height in Bangla and English beside the heading 'height'.
6. Roll out the ball of string beside the child and cut to the height of the child. Get children to compare their pieces of string with each other. Who is the shortest? Who is the tallest? Put in order of shortest to tallest and then stick on the wall with blue-tack. Get the children to write (or copy) their name onto a piece of paper and stick it under their string on the wall.
7. Instruct the children to stand in front of their piece of string, so that they are standing in order from shortest to tallest.

Class 11: Who am I?

To develop:

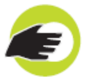

Arm and hand control: Pencil grip and control, writing, drawing, cutting and pasting.

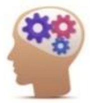

Cognition: Recalling personal information about self and others.

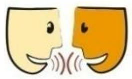

Language and communication: Verbal, visual and written communication of own name and other characteristics about self.

Need:

- A4 pieces of paper (1 for each child)
- Pencil (1 for each child)
- Magazines
- Scissors
- Glue
- Number cards

Instructions:

1. Write the following headings on the white board:
   'How many sisters do I have?'
   'How many brothers do I have?'
   'What is my favourite food?'
   'What food do I hate?'
   'What is my favourite toy?'
   'What is my favourite place to go?'
   'What is my favourite activity?'
   'Where do I live?'
   'When is my birthday?'
   'My name is:'
2. Instruct the children able to write sentences to copy these headings onto their piece of paper. For those children unable to write sentences, ask the adults in the room to copy these headings on strips of paper, and instruct the children to glue them in order on their piece of paper.
3. Ask each question to the class and assist the children to communicate their answer verbally and/or visually, and then write, draw or cut and paste a picture of their answer next to the heading on their piece of paper. Provide prompts, actions, visuals etc. to help the child answer the question.
4. Collect the completed papers. Introduce the 'Who am I' game. Tell the children that I am going to pretend to be one of you children, and you are detectives! I will give you clues, and you need to work out who I am. The first person to say who I am and get it correct is the winner.
5. Start the game, pause for 3 to 5 seconds between each question to give children time to answer. Say: "Who am I?" "I have .... sisters". Pause. "I have.... brothers". Pause. "My favourite food is....." Pause. "I hate to eat....." Pause. "My favourite toy is...." Pause. "My favourite place to go is...." Pause. My favourite activity is...." Pause. "I live in ...." Pause. My birthday is...." Pause. My name is?"
6. Continue until all the children's papers have been read.

Class 12: My Daily Routine

To develop:

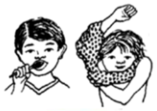

Activities of daily living: Putting on and taking off shoes, washing face and hands

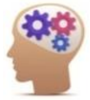

Cognition: Recall, sequencing activities

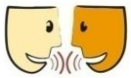

Language and communication: verbally and/or visually communicating daily activities

Need:

- White-board and marker
- Activities of daily living picture cards
- Blue-tack
- Bucket
- Washcloth x 2
- Towel x 2
- Hand soap x 2

Instructions:

1. Explain to the group that routine means the activities that we must do every day or regularly, and what order to we do these activities in.
2. Ask the group, “What are some activities that you do every day?” Write a list on the board. Use communication boards and picture cards to help include children who have difficulty verbally communicating.
3. Ask the group, “What are some activities that you do every week?” Write a list on the boards. Use communication boards and picture cards as required.
4. Using the number cards and picture cards, show the children your morning routine. E.g. using blue-tack, stick the number 1 card on the board. Then stick the picture card showing getting up out of bed, next to number 1. Then stick the number 2 card below number 1. Stick the picture card showing eating, next to number 2 etc.
5. Give children a set of number cards 1,2,3, and three picture cards each. Ask the children to put the numbers in order. Then ask the children to pick the picture card showing what they did first this morning, what they did second, and what they did third, and put it next to the corresponding number.
6. Get the children to say/show the class their sequence.
7. Introduce the song, “This is the way we.... wash our face, brush our teeth, yawn and stretch, eat our nasta”, etc. Show the children actions and instruct the children to copy these actions.
8. Tell the children that now we’ll practice some of the things you do in the morning. Tell the children to show your mum’s how grown up and helpful they are by doing it yourself! Today

we're going to put on our shoes and then wash our face and hands. Remember how we wash our face? Show the action and sing "this is the way we wash our face" with the children. Remember how we wash our hands? Show the action and sing "this is the way we wash our hands".

9. Instruct the children to find their shoes and put their shoes on independently or with minimal help (set up the task so that the child is being challenged, but is able to complete some of the steps independently, e.g. identifying their shoe, slipping their foot into the shoe, opening and closing the Velcro shoe. Instruct the children to them move to the bathroom tap and wait in a straight line.
10. Have a bucket with water and plastic chair set-up to use for those children who cannot reach the sink or stand independently. Have a wash cloth in the bucket and beside the sink, and hand soap and dry towel beside the sink and bucket.
11. One by one, ask the children to and wash their face and then their hands.
    a. For children able to stand and reach the sink: Get them to stand in front of the sink, turn on the tap, splash the water on their face and use the wash cloth (if they usually use this at home), dry their face, put soap on their hands, wash their hands, turn off the tap and dry their hands.
    b. For children unable to reach the sink, but able to stand: Get them to stand beside the bucket, turn on the tap to put water in the bucket, turn off the tap, splash the water on their face and use the wash cloth (if they usually use this at home), dry their face, put soap on their hands, wash their hands and then dry their hands.
    c. For children unable to stand: Get them to sit on a plastic chair beside the bucket, turn on the tap to put water in the bucket, turn off the tap, splash the water on their face, and use the wash cloth (if they usually use this at home), dry their face, put soap on their hands, wash their hands and then dry their hands.
12. Once finished washing their face and hands, instruct the child to return to the classroom, take off their shoes, and wait for nasta.

The key points to helping a child gain independence in activities of daily living are:

- Be patient, give the child TIME to think through and attempt/complete steps of the task themself.
- Recognise and acknowledge a child's attempts and ability to complete steps of the task independently. E.g. a child may not be able to turn the tap on enough for a steady stream of water to come out, but they may be able to reach out, loosely grasp the tap, and give one turn of the tap. With practice and over time, the child will be able to grasp the tap a little firmer, and turn further.
- Do NOT take over and do the task for them. If the task is too difficult for them, think why is it too difficult? Is the child trying to move a tight tap? Then, just loosen the tap a little and get the child to try again. FACILITATE ability.
- Provide prompts only if the child is not initiating or skips a step of the task, i.e. only provide a prompt if it is needed, and do not continually prompt the child through a task. Provide a prompt and then give the child time to think and do.
- Only provide assistance to facilitate ability, and make the step of the task achievable for the child. Make sure that you don't provide so much assistance that the child is not actively participating. Ensure that the step of the task is still a challenge, but provide assistance so that it is achievable for the child. The child needs to do AS MUCH as they can themselves, and ALWAYS actively participate

Class 13: Fingerprints

To develop:

Cognition: Identify similarities and differences

Arm and hand control: Pencil grasp and control, handwriting

Language and communication: Describing characteristics of self and others

Need:

- Stamp pad
- Paper
- Pencils
- Laminated name cards

Instructions:

1. Lead a group discussion about similarities and difference in appearance. Ask children about their hair colour, eye colour, height, number of fingers, number of toes, left or right handedness. What is similar and what is different? Write a list on the board of similarities and differences and discuss.
2. Tell children to look at their hand, do you see all the lines on your hand? Have a look at the person hand beside you. Are your lines the same or different?
3. Now look at your fingertips. Do you see all the little lines? We can see these lines better by putting coloured ink on our finger and using our thumb/finger like a stamp.
4. Tell children to press their thumb on the stamp pad and then press onto their piece of paper to make a thumbprint. Can you see all the little lines?
5. Tell the children we're going to investigate whether the little lines on our fingers and thumb are the same or different as each other. Allocate a particular finger print for each child to collect. E.g. one child to collect thumb print, one child to collect index finger print etc. Ask children to collect that thumb/finger print of all other students in the class. Once you make a print, make sure you write your name underneath it. Use the laminated name cards if required.
6. After all prints are finished, ask children if the thumbprints are the same or different. Ask children to describe and show the group how they are different.
7. To finish this class, instruct the children to turn their thumbprints into animals by drawing extra lines and circles etc. They can turn the prints into chickens, cows etc.

Homework: Children to put together a 'me' bag that contains their favourite toys, book, fruit, pictures etc. Make sure you explain to the parents that the children need to choose the items that go in the bag, not the parents. The parents can provide prompts by asking questions to the child – what is your favourite toy, fruit etc. But make sure the child makes the choice, not the parent.

Class 14: ‘Me’ Bag

To develop:

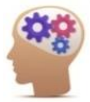

Cognition: Identify characteristics and use of their favourite items

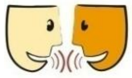

Language and communication: Describing their favourite items, importance and use

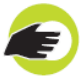

Arm and hand control: Grasping and maintaining hold on items

Need:

- Children to have completed their homework from the previous day: putting together a ‘me’ bag that contains their favourite toys, book, fruit, pictures etc. Make sure you explain to the parents that the children need to choose the items that go in the bag, not the parents. The parents can provide prompts by asking questions to the child – what is your favourite toy, fruit etc. But make sure the child makes the choice, not the parent.

Instructions:

1. Explain to the children that will now have a ‘me’ presentation in which children show their favourite items to the rest of the class and discuss the importance of the items in their bag.

2. One child at a time to present to the rest of the group. Show one item at a time and describe that item.

3. Prompt children as required by asking about their item – what colour is it? Who do you play with it with? How often do you use this? What do you use it for? Etc.

4. Ask the child to pass around an item around the group so that other children can look. Then ask the group questions about the item.

Class 15: My Story, My Favourite Things

To develop:

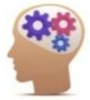

Cognition: Identify their favourite things

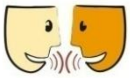

Language and communication: verbal, visual, written communication of favourite things

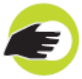

Arm and hand control: Pencil grasp, control and handwriting

Need:

- My Story book
- Pencil
- Colour pencils
- Glue

Instructions:

1. Write these headings on the whiteboard:
   My favourite colour is:
   My favourite food is:
   My favourite toy is:
   My favourite animal is:
   My favourite holiday is:
   My favourite book is:
   My favourite song is:
2. Instruct the children able to write sentences to copy these headings onto their piece of paper. For those children unable to write sentences, ask the adults in the room to copy these headings onto strips of paper so that the child can put them in order and glue them into their book.
3. Ask children to answer each question by drawing and writing a word.
4. Ask adults to write the words on a separate piece of paper for children to copy. Show them the letters that makes up the word and the sounds each letter makes.
5. Once finished, one at a time, children to share their answers with the rest of the class.

Class 16: What Will I Wear Today?

To develop:

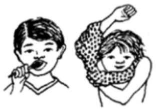

Activities of daily living: Dressing

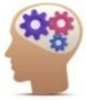

Cognition: Awareness of weather and personal needs

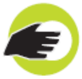

Arm and hand control: Doing up and undoing buttons and zips

Need:

3 of each:

- Jumpers
- Jackets
- Socks
- Shawl
- Umbrellas
- Raincoat
- T-shirt
- Shorts
- Salwar Kameez
- Hat
- Weather picture cards
- Whiteboard marker and whiteboard

Instructions:

1. Talk to the group about the different weather conditions. Ask the children, what are the different seasons? Write these on the board. Then ask the children, during the winter season is it hot or cold? What do you wear to try and stay warm? Write these on the board and show the different items of clothing to the group. Ask, during the summer season is it hot or cold? What do you wear to try and stay cool? Again write these on the board and ask the class to pick up the items of clothing they would wear from the pile and show the rest of the class. Discuss the other seasons, temperatures and what you would wear.
2. Talk about the different items of clothing. Hold up a hat. Ask the class, what is this for? How do you wear this? What body part do you put this on? Do boys, girls or both boys and girls wear this? Do this for each item of clothing.
3. Introduce the game to the group: We are now going to play a game. I will say what the weather is like today, and I will show you a picture of the weather today. Then you have to choose one item of clothing that you would need to wear to keep warm, cool, or dry, and put it on. You need to put the item on yourself! The first person to put the item on is the winner.
4. Hold up one picture card, say 'The weather today is.....' and put the card on the board. Allow enough time for all children to finish the activity. Only provide prompts and assistance if/when the child doesn't initiate the task, or the task is too difficult. Only provide assistance to make the task easier, do not do the task for the child. Make sure the child is challenged and participating!
5. Continue this for all weather cards. Repeat weather cards more than once.
6. Write on the board the winner of each round of the game.

Class 17: Getting Ready to Start My Day

To develop:

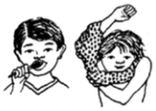

Activities of daily living: Washing face, brushing teeth, brushing hair

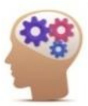

Cognition: Sequencing

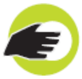

Arm and hand control: Doing up and undoing buttons and zips

Need:

- Children to complete homework – bring their toothbrush and hairbrush
- White-board and marker
- Activities of daily living picture cards
- Tubes of toothpaste
- Blue-tack
- Bucket
- Washcloth x 2
- Towel x 2

Instructions:

1. Discuss with the group that routine means the activities that we must do every day or regularly, and what order to we do these activities in. Ask the group, "What do you do every day in the morning?" Write a list on the board. Use communication boards and picture cards to help include children who have difficulty verbally communicating.
2. Using the number cards 1 to 6, and picture cards of getting up out of bed, washing face, brushing teeth, brushing hair, eating and leaving home, show the children your morning routine before starting your day. Using blue-tack, stick the number 1 card on the board. Then stick the picture card showing getting up out of bed, next to number 1. Then stick the number 2 card below number 1. Stick the picture card showing washing face, next to number 2, etc.
3. Give children a set of number cards 1 to 6, and a set of picture cards each. Ask the children to put the numbers in order. Then ask the children to pick the picture card showing what they did first this morning, what they did second, and what they did third etc. and put it next to the corresponding number.
4. Get the children to say/show the class their sequence.
5. Introduce the song, "This is the way we.... wash our face, brush our teeth, yawn and stretch, eat our nasta", etc. Show the children actions and instruct the children to copy these actions.
6. Tell the children that now we'll practice some of the things you do in the morning. Tell the children to show your mum's how grown up and helpful they are by doing it yourself! Today we're going to put on our shoes, wash our face, brush our teeth and brush our hair. Remember how we wash our face? Show the action and sing "this is the way we wash our face" with the children. Remember how we brush our teeth? Show the action and sing "this is the way we brush our teeth".
7. Instruct the children to find their shoes and put their shoes on independently or with minimal help (set up the task so that the child is being challenged, but is able to complete some of the steps independently, e.g.

identifying their shoe, slipping their foot into the shoe, opening and closing the Velcro shoe. Instruct the children to them move to the bathroom tap and wait in a straight line.

8. Have a bucket with water and plastic chair set-up to use for those children who cannot reach the sink or stand independently. Have a wash cloth in the bucket and beside the sink, and a tube of toothpaste and dry towel beside the sink and bucket.
9. One by one, ask the children to and wash their face and brush their teeth.
    a. For children able to stand and reach the sink: This is the sequence for the children to complete as independently as possible: Stand in front of the sink, turn on the tap, splash the water on their face and use the wash cloth (if they usually use this at home), dry their face, put toothpaste on their toothbrush, brush their teeth, spit, wash their mouth, turn off the tap and dry their mouth.
    b. For children unable to reach the sink, but able to stand: This is the sequence for children to complete as independently as possible: Stand beside the bucket, turn on the tap to put water in the bucket, turn off the tap, splash the water on their face and use the wash cloth (if they usually use this at home), dry their face, put toothpaste on their toothbrush, brush their teeth, spit, wash their mouth, and then dry their mouth.
    c. For children unable to stand: Get them to sit on a plastic chair beside the bucket, turn on the tap to put water in the bucket, turn off the tap, splash the water on their face, and use the wash cloth (if they usually use this at home), dry their face, put toothpaste on their toothbrush, brush their teeth, spit, wash their mouth, and then dry their mouth.
10. Once finished washing their face and brushing their teeth, instruct the child to return to the classroom, take off their shoes.
11. Ask children to pick up/sit beside a mirror, hold their hairbrush and brush their hair.
12. Once all children are finished, say now we are ready to start the day. Talk about how they can do this every morning, as much as they can by themselves.

The key points to helping a child gain independence in activities of daily living are:

- Be patient, give the child TIME to think through and attempt/complete steps of the task themself.
- Recognise and acknowledge a child's attempts and ability to complete steps of the task independently. E.g. a child may not be able to turn the tap on enough for a steady stream of water to come out, but they may be able to reach out, loosely grasp the tap, and give one turn of the tap. With practice and over time, the child will be able to grasp the tap a little firmer, and turn further.
- Do NOT take over and do the task for them. If the task is too difficult for them, think why is it too difficult? Is the child trying to move a tight tap? Then, just loosen the tap a little and get the child to try again. FACILITATE ability.
- Provide prompts only if the child is not initiating or skips a step of the task, i.e. only provide a prompt if it is needed, and do not continually prompt the child through a task. Provide a prompt and then give the child time to think and do.
- Only provide assistance to facilitate ability, and make the step of the task achievable for the child. Make sure that you don't provide so much assistance that the child is not actively participating. Ensure that the step of the task is still a challenge, but provide assistance so that it is achievable for the child. The child needs to do AS MUCH as they can themselves, and ALWAYS actively participate.

Class 18: Who am I? Feelings Bingo

To develop:

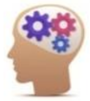

Cognition: Recall, awareness of self and others, similarities and differences, matching

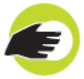

Arm and hand control: Grasp and release

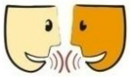

Language and communication: Expressing feelings and needs

Need:

- Completed 'Who am I' sheets from class 11

Instructions:

1. Show the class the Who am I sheets from class 11 and discuss with the class. Remind the class of the Who am I game from class 11: Tell the children "I am going to pretend to be one of you, and you are detectives! I will give you clues, and you need to work out who I am. The first person to say who I am and get it correct is the winner".
2. Start the game, pause for 3 to 5 seconds between each question to give children time to answer. Say: "Who am I?" "I have .... sisters". Pause. "I have.... brothers". Pause. "My favourite food is....." Pause. "I hate to eat....." Pause. "My favourite toy is...." Pause. "My favourite place to go is...." Pause. My favourite activity is...." Pause. "I live in ...." Pause. My birthday is...." Pause. My name is?"
3. Continue until all the children's Who am I sheets have been read.
4. Show the children the feelings picture cards. Instruct children to ask each other, how are you feeling today, and children to answer verbally and by selecting the corresponding picture card.
5. Show the children the needs cards. Show the children each picture and ask what the picture is. Then ask the children to select the card to represent my need. Say, I need to go to the toilet. Wait/prompt the children to select the card that says toilet. Then say, I need to drink water. Wait/prompt the children to select the card that has a glass of water. Etc.
6. Tell the class "We are now going to play a game using these feelings and needs cards. The game is called bingo". Give each child a feelings and needs bingo card, and 9 bottle lids each. Tell the children that you will read out and show a feeling/need card and the children have to look at their bingo card, if they have the same picture on their card, and put a bottle lid on top of that picture. As soon as you have 3 bottle lids in a row, you have to put up your hand and call "BINGO". The first person to put their hand up and call bingo is the winner.
7. Play at least three rounds of bingo.

Class 19: 'Me' Shirt

To develop:

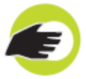

Arm and hand control: Painting, drawing

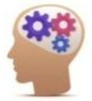

Cognition: Identify characteristics and use of their favourite items

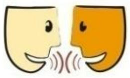

Communication: Describing their favourite items, importance and use

Need:

- Plain, light-coloured T-shirt from home for each child
- Paints or markers designed for use on clothing
- Smocks (one for each child)

Instructions:

1. Explain that today we are going to create a Me-shirt, a T-shirt that expresses an important personal trait.
2. Discuss with the children that many T-shirts have art or writing on them. Some children may be wearing such T-shirts. Encourage these children to stand up and show their T-shirts to the class. Ask other children if they have any similar shirts at home. On the board, list the reasons children like to wear these kinds of T-shirts. If children have difficulty thinking of reasons, you might offer some, for e.g. they can show what team you're on, they can show places you've been, they're pretty, they're funny.
3. Tell children that they are each going to design a T-shirt. Explain that the design should tell about something personal. It could include the child's name, and pictures of favourite things to do, of family members, or even of their favourite animal.
4. When children are ready, have them draw or paint their designs of their Me-shirts.
5. Hang them up to dry.
6. Once they are dry, instruct the children to put them on, as independently as possible. As the children to show and describe what is on their Me-shirt to the class.
7. Tell the children they can keep their Me-shirt and take it home.

Class 20: My Story, My Favourite Activities

To develop:

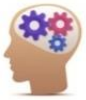

Cognition: Identify their favourite activities

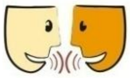

Language and communication: Express their favourite things

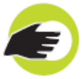

Arm and hand control: Pencil grasp, control and handwriting

Need:

- My Story book
- Pencil
- Colour pencils
- Magazines
- Glue
- Scissors

Instructions:

1. Write these headings on the whiteboard:
   I like to play with:
   My favourite place to go is:
   My favourite game is:
   I like to draw:
   I like to make:
   I like to collect:
   My favourite sports is:
   Other activities I like to do are:
2. Instruct the children able to write sentences to copy these headings onto their piece of paper. For those children unable to write sentences, ask the adults in the room to copy these headings onto strips of paper so that the child can put them in order and glue them in their book.
3. Ask children to answer each question by drawing a picture, ripping/cutting a picture out of a magazine and gluing it in their book, and by writing a word.
4. Ask adults to write the words on a separate piece of paper for children to copy. Show them the letters that makes up the word and the sounds each letter makes.
5. Once finished, one at a time, children to share their answers with the rest of the class.
6. Then play Who am I using the completed favourite activities.

Classes

Month 2: My Family and Friends

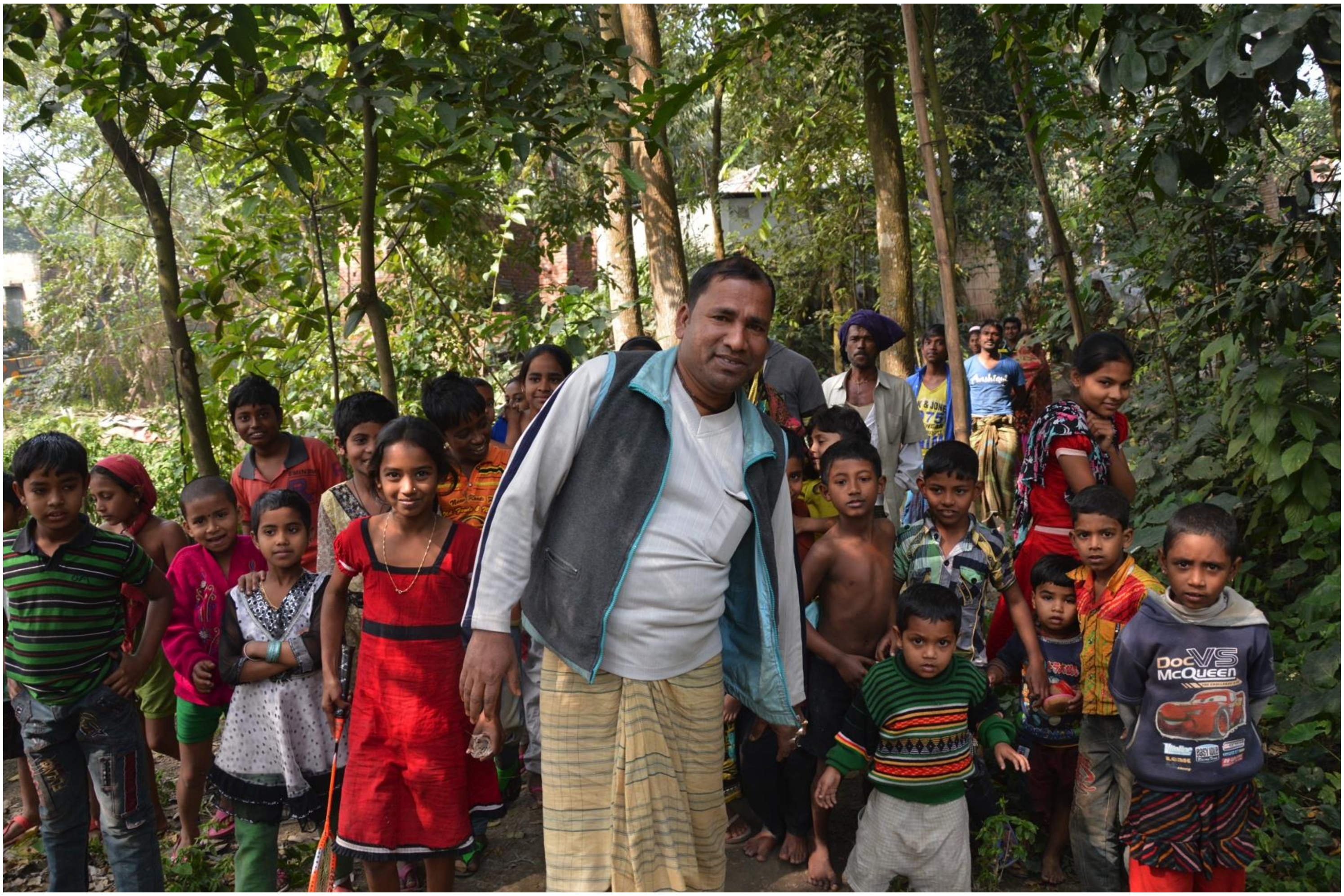

Class 21: My Story, Who is in My Family?

To develop:

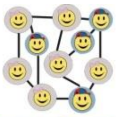

Social behaviour: Awareness of roles of different family members

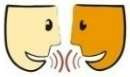

Language and communication: Naming different family members

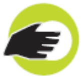

Arm and hand control: Gluing

Need:

- Family picture cards
- My Story book
- Glue

Instructions:

1. Introduce the new topic for the month – my family and friends.
2. Ask the class, "What does family mean?" Discuss with the children that family are people you are related to genetically, people who raise you, or grow up with you, live with you, and are very close to you.
3. Ask the children, who are members of your family? Write on the board their answers – mother, father, sister, brother, auntie, uncle, cousin, grandmother, grandfather.
4. Show a picture of a family – it could be a photo, or a picture from a magazine or newspaper. Name the different members of the family.
5. Give each child a set of family picture cards. Ask the children to select the cards that show who are in their family and glue the pictures into their My Story book.
6. Ask children to raise their hand if they have this person in their family: Call out "Mother". Children raise their hands. Discuss all children have raised their hand, we all have mothers. Call out "sister". Discuss not all children have sisters. Etc.

Homework: Children ask the different members of their family to write their name beside the picture that describes them. E.g. Children need to ask their mother to write their name beside the picture of 'Mother'.

Class 22: My Story, Who am I in My Family?

To develop:

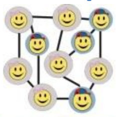

Social behaviour: Awareness of role in the family

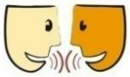

Language and communication: Naming relationships to other family members, reading words

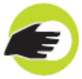

Arm and hand control: Gluing

Need:

- My Story book
- Paper
- Marker
- Glue

Instructions:

1. Prepare a set of 'Who am I in my Family' word cards by writing these words on one piece of paper:
    a. Daughter
    b. Son
    c. Brother
    d. Sister
    e. Grandson
    f. Granddaughter
    g. Cousin
    h. Nephew
    i. Niece

    Photocopy the piece of paper so that there is one for each child.
    Cut out each word to make individual cards.
2. Ask children one by one, to present their homework to the class. Instruct children to point to the picture representing their family member, and point and say their role (e.g. mother) and their name (e.g. Farhana).
3. Discuss with the children that they also fill many different roles in their families. Explain that a girl may be a daughter, a sister, a niece, and a granddaughter, while a boy may be a son, a brother, a nephew, and a grandson.
4. Ask children to raise their hand if they are this person in their family: Call out "Brother" "Sister" "Cousin", daughter, son, granddaughter, grandson, auntie, uncle etc. Explain all sisters are girls and all brothers are boys.
5. In each child's book, write the heading "In my family, I am a..."
6. Give children a set of word cards. Ask children to select the word card that says who they are in their family and then glue it in their book. Provide prompts and assistance by sounding out the word, writing the word so that the child can find the card that matches etc.
7. Share with the rest of the class and discuss.

Class 23: Rulers and Squares, Preparation for Classroom Quilt

To develop:

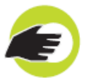

Arm and hand control: Pencil grip and control, drawing and cutting straight lines

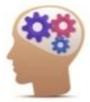

Cognition: Recognition of shapes, understanding ruler use

Need:

- **My Family Home Handout**
- Coloured paper
- Rulers
- Scissors
- Writing pencils
- A rectangle block and a square block

Instructions:

1. Give each child a piece of coloured paper and the house handout.
2. Instruct children to cut out the house.
3. Instruct the children to cut out a square from the coloured paper that is 2cm longer than the house. Demonstrate how you draw and then cut out a square. Encourage each child to do as much as possible themselves. Provide prompts and assistance as required.
4. Children to show each other their finished house cut-outs and squares.
5. Keep the house cut-outs and squares to use tomorrow for the classroom quilt.

My Family Home

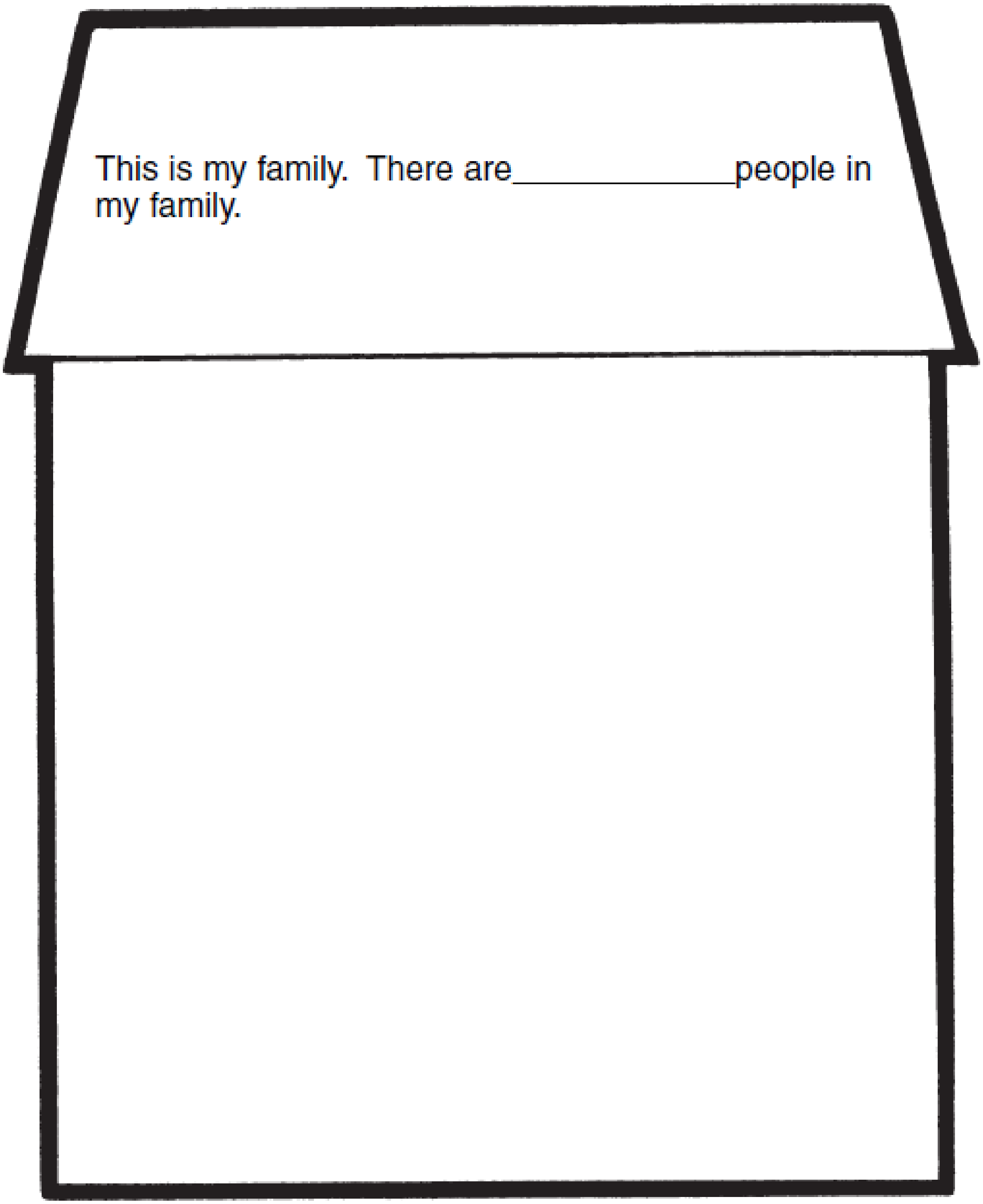

Class 24: Classroom Quilt, My Family

To develop:

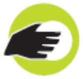

Arm and hand control: Drawing, writing, gluing, sewing

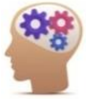

Cognition: Recalling family members, understanding different sizes and heights with age

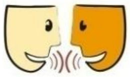

Language and communication: Describing family and roles of family members

Need:

- House cut-outs
- Squares of coloured paper
- Coloured pencils/crayons
- Glue
- Sticky tape
- String
- Hole-punch

Instructions:

1. Tell the children that now you we will make a quilt showing the people in each of our families.
2. Provide each child with a cut-out house.
3. Instruct children to draw their family inside their house, and write how many people are in their family.
4. Children can trace around their bigger fingers e.g. index finger to help them draw their mother and father, and trace around their smaller fingers e.g. little finger to help them draw themselves, and their brothers and sisters on their white square. Write who they are below the finger drawing e.g. sister, brother, mum and dad. Demonstrate this on the whiteboard.
5. Instruct each child to centre and paste the completed house on a coloured square and write their name on the bottom of the square. Children can copy their name if unable to recall letters.
6. When all the children have completed their square, hole-punch each side of the square.
7. Give children a piece of string. Tie one end of string to one hole on their square. Ask the children to thread the squares together to form a quilt pattern.
8. Display the quilt and have each child talk about their own family drawing to the rest of the group.

Class 25: My Story, My Family Tree

To develop:

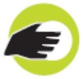 Arm and hand control: Drawing, writing, gluing.

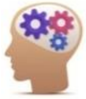 Cognition: Recalling family members, relationships and names.

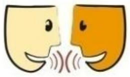 Language and communication: Describing family members.

Need:

- **My Family Tree Handout**
- My Story books
- Pencils
- Scissors
- Glue

Instructions:

1. Explain to the children what a family tree is. Draw an example of a family tree on the board.
2. Give each child a family tree handout.
3. Tell children to cut out the tree and glue it in their My Story book.
4. Tell children to fill out the spaces by writing the name of their family member and drawing a picture. Children can refer back to the names written on 'Who is in my Family' page of My Story.
5. Share with the class and discuss.

Homework: Children collect the handprints of themselves, their mother, father, and brother/sisters. Bring these handprints to class tomorrow, for making a family flower.

My Family Tree

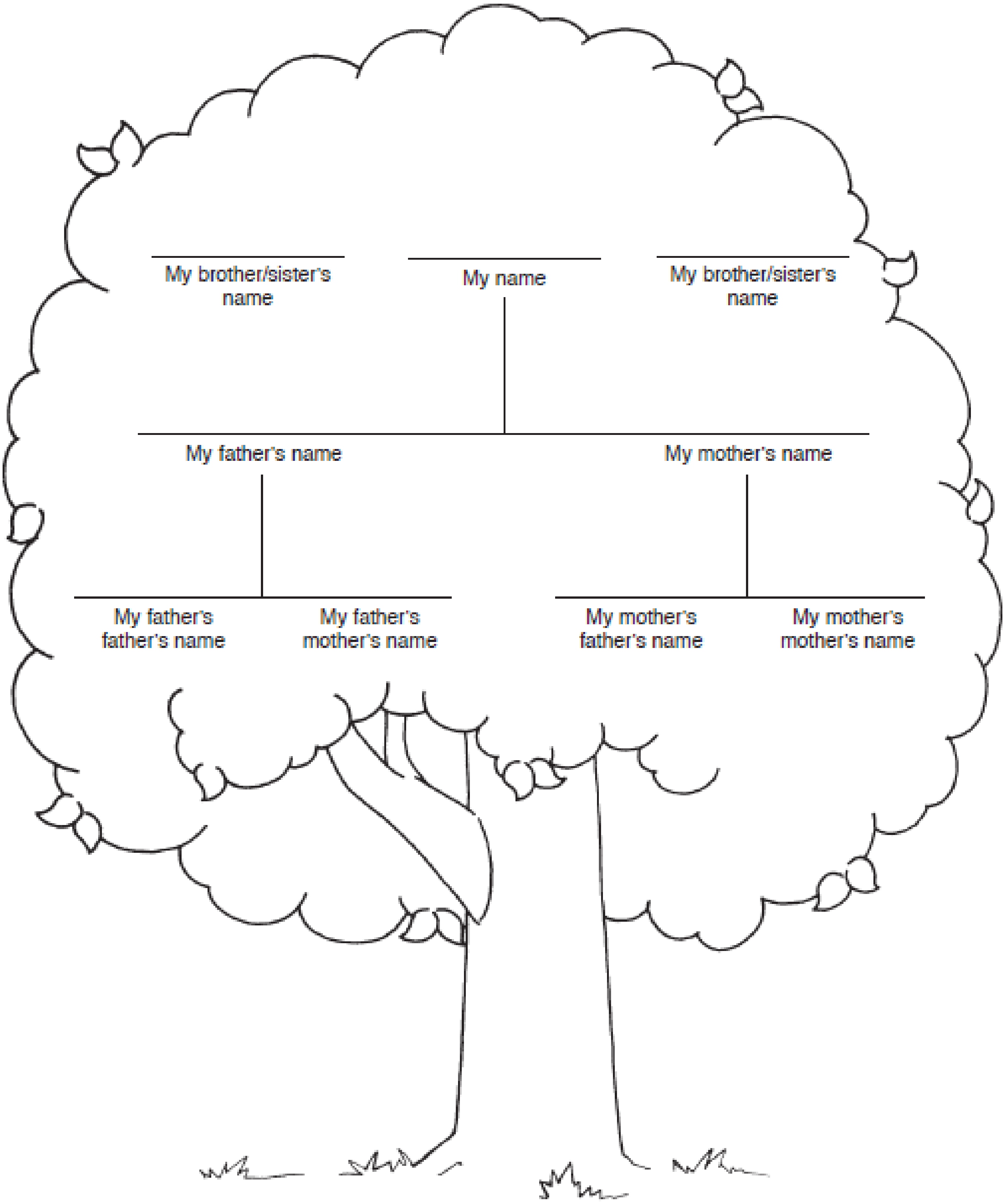

Class 26: Family Flower

To develop:

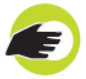

Arm and hand control: Drawing, writing, cutting, gluing

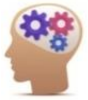

Cognition: Sequencing steps of task to create flower

Need:

- Children to bring their homework – collect the handprints of their mother, father, brothers, sisters
- Sticky-tape
- A4 pieces of paper
- Glue
- Scissors
- Glitter and other craft materials
- Colour pencils

Instructions:

1. Tell children to cut out the handprints.
2. Tell children to go outside and collect two leaves and a stick.
3. Give each child a piece of plain piece of paper.
4. Children to use sticky-tape to stick the stick on the piece of paper. This will be the stem of the flower.
5. Children to use glue to stick the leaves along the stem.
6. Position the handprints with largest hand first (to the back) and smallest to the front. Use glue to stick it on the paper, above the stem.
7. Children can decorate the flower and write a message to their family.

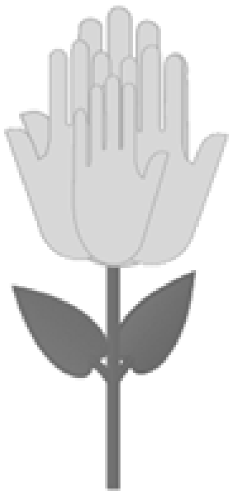

Homework: Children give the flower to their family.

Class 27: My Story, How do I Spend Time with My Family?

To develop:

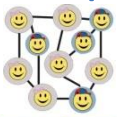

Social behaviour: Awareness of role in the family

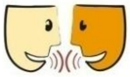

Cognition: Recalling activities with different members of the family

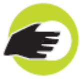

Arm and hand control: Writing, drawing, cutting, gluing

Need:

- My Story book
- Magazines/ newspapers
- Writing pencils
- Colouring pencils
- Glue
- Scissors

Instructions:

1. Discuss with the children how you spend time with different family members. How you spend time with your mum might be different to how you spend time with your dad, and how you spend time with your sisters and brothers.
2. Write the heading on the board: How do I spend time with...
3. Write the subheadings below: Mother, Father, Sisters, Brothers
4. Ask the children: How do you spend time with your mother? What do you do with your mother? Write the answers next to the subheading 'mother'.
5. Ask the children: How do you spend time with your father? What do you do with your father? Write the answers next to the subheading father.
6. Continue for sisters and brothers.
7. Tell children they will now make a collage showing how they spend time with their family, in their My Story books.
8. Ask children to copy the headings into their books, leaving space for them to glue/draw pictures. If a child is unable to copy the heading, write the headings on strips of paper and ask the child to glue the headings in order in their My Story book.
9. Instruct the children to cut out, or rip out pictures they find in magazines showing how they spend time with different members of their family, and glue the pictures next to the person they do that activity with. The children can also draw pictures if they can't find anything in the magazines.
10. Children show and discuss their collage with the rest of the class.

Class 28: What Will I Wear Today?

To develop:

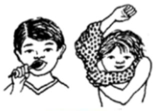

Activities of daily living: Dressing.

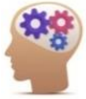

Cognition: Dressing for the weather.

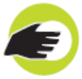

Arm and hand control: Doing up and undoing buttons and zips.

Need:

3 of each:

- Jumpers
- Jackets
- Socks
- Shawl
- Umbrellas
- Raincoat
- T-shirt
- Shorts
- Salwar Kameez
- Hat
- Weather picture cards
- Whiteboard marker and whiteboard

Instructions:

1. Talk about how you spend time with your family getting ready for your day. You can be helpful and more grown up by trying to do as much as you can yourself to get ready. For instance, choosing what you will wear for the day and getting dressed.
2. Talk to the group about the different weather conditions. Ask the children, what are the different seasons? Write these on the board. Then ask the children, during the winter season is it hot or cold? What do you wear to try and stay warm? Write these on the board and show the different items of clothing to the group. Ask; during the summer season is it hot or cold? What do you wear to try and stay cool? Again write these on the board and ask the class to pick up the items of clothing they would wear from the pile and show the rest of the class. Discuss the other seasons, temperatures and what you would wear.
3. Talk about the different items of clothing. Hold up a hat. Ask the class, what is this for? How do you wear this? What body part do you put this on? Do boys, girls or both boys and girls wear this? Do this for each item of clothing.
4. Introduce the game to the group: We are now going to play a game. I will say what the weather is like today, and I will show you a picture of the weather today. Then you have to choose one item of clothing that you would need to wear to keep warm, cool, or dry, and put it on. You need to put the item on yourself!
5. Hold up one picture card, say 'The weather today is.....' and put the card on the board. Allow enough time for all children to finish the activity. Only provide prompts and assistance if/when the child doesn't initiate the task, or the task is too difficult. Only provide assistance to make the task easier, do not do the task for the child. Make sure the child is challenged and participating!
6. Continue this for all weather cards. Repeat weather cards more than once.

Homework: Children to dress as independently as possible at home. Bring a toothbrush and hairbrush tomorrow for class.

Class 29: Getting Ready to Start My Day

To develop:

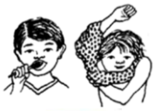

Activities of daily living: Washing face, brushing teeth, brushing hair

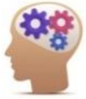

Cognition: Sequencing

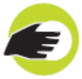

Arm and hand control: Doing up and undoing buttons and zips

Need:

- Children to complete homework – bring their toothbrush and hairbrush
- White-board and marker
- Activities of daily living picture cards
- Tubes of toothpaste
- Blue-tack
- Bucket
- Washcloth x 2
- Towel x 2

Instructions:

1. Talk about how you spend time with your family getting ready for your day. You can be helpful and more grown up by trying to do as much as you can yourself to get ready. For instance, choosing what you will wear for the day and getting dressed.
2. Discuss with the group that routine means the activities that we must do every day or regularly, and what order to we do these activities in. Ask the group, "What do you do every day in the morning?" Write a list on the board. Use communication boards and picture cards to help include children who have difficulty verbally communicating.
3. Using the activities of daily living picture cards: getting up out of bed, washing face, brushing teeth, brushing hair, eating and leaving home, ask the children to put the cards in order of what they did this morning. Provide prompts as required.
4. Get the children to say/show the class their sequence.
5. Sing the song, "This is the way we.... wash our face, brush our teeth, yawn and stretch, eat our nasta", etc. Show the children actions and instruct the children to copy these actions.
6. Tell the children that now we'll practice some of the things you do in the morning. Tell the children to show your mum's how grown up and helpful they are by doing it yourself! Today we're going to put on our shoes, wash our face, brush our teeth and brush our hair.
7. Instruct the children to find their shoes and put their shoes on independently or with minimal help (set up the task so that the child is being challenged, but is able to complete some of the steps independently, e.g. identifying their shoe, slipping their foot into the shoe, opening and closing the Velcro shoe. Instruct the children to them move to the bathroom tap and wait in a straight line.
8. Have a bucket with water and plastic chair set-up to use for those children who cannot reach the sink or stand independently. Have a wash cloth in the bucket and beside the sink, and a tube of toothpaste and dry towel beside the sink and bucket.
9. One by one, ask the children to wash their face and brush their teeth.

a. For children able to stand and reach the sink: This is the sequence for the children to complete as independently as possible: Stand in front of the sink, turn on the tap, splash the water on their face and use the wash cloth (if they usually use this at home), dry their face, put toothpaste on their toothbrush, brush their teeth, spit, wash their mouth, turn off the tap and dry their mouth.
b. For children unable to reach the sink, but able to stand: This is the sequence for children to complete as independently as possible: Stand beside the bucket, turn on the tap to put water in the bucket, turn off the tap, splash the water on their face and use the wash cloth (if they usually use this at home), dry their face, put toothpaste on their toothbrush, brush their teeth, spit, wash their mouth, and then dry their mouth.
c. For children unable to stand: Get them to sit on a plastic chair beside the bucket, turn on the tap to put water in the bucket, turn off the tap, splash the water on their face, and use the wash cloth (if they usually use this at home), dry their face, put toothpaste on their toothbrush, brush their teeth, spit, wash their mouth, and then dry their mouth.

10. Once finished washing their face and brushing their teeth, instruct the child to return to the classroom, take off their shoes.
11. Ask children to pick up/sit beside a mirror, hold their hairbrush and brush their hair.
12. Once all children are finished, say now we are ready to start the day. Talk about how they can do this every morning, as much as they can by themselves.

The key points to helping a child gain independence in activities of daily living are:

- Be patient, give the child TIME to think through and attempt/complete steps of the task themself.
- Recognise and acknowledge a child's attempts and ability to complete steps of the task independently. E.g. a child may not be able to turn the tap on enough for a steady stream of water to come out, but they may be able to reach out, loosely grasp the tap, and give one turn of the tap. With practice and over time, the child will be able to grasp the tap a little firmer, and turn further.
- Do NOT take over and do the task for them. If the task is too difficult for them, think why is it too difficult? Is the child trying to move a tight tap? Then, just loosen the tap a little and get the child to try again. FACILITATE ability.
- Provide prompts only if the child is not initiating or skips a step of the task, i.e. only provide a prompt if it is needed, and do not continually prompt the child through a task. Provide a prompt and then give the child time to think and do.
- Only provide assistance to facilitate ability, and make the step of the task achievable for the child. Make sure that you don't provide so much assistance that the child is not actively participating. Ensure that the step of the task is still a challenge, but provide assistance so that it is achievable for the child. The child needs to do AS MUCH as they can themselves, and ALWAYS actively participate

Homework: Children get ready as independently as possible at home.

Class 30: Feelings & Needs

To develop:

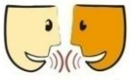

Language and communication: Express feelings and needs verbally and visually

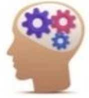

Cognition: Self awareness, matching

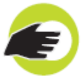

Arm and hand control: Pencil grip and control, drawing

Need:

- Pictures of different people's faces cut out from a magazine or newspaper
- Magazines/newspapers
- **"I feel" Picture Cards** or **Feelings Symbols**. Refer to Appendix: Communication Resources for details.
- Communication board. Refer to Appendix: Communication Resources for details.
- Paper
- Coloured pencils, crayons or markers
- **Cube template**
- Scissors
- Glue

Instructions:

1. Discuss with the children how it is important in a family to communicate your feelings and needs.
2. Ask the children, how do you show when you are happy? How do you show you are sad? How do you show you are angry? How do you show you are hurting? How do you show you are tired? Encourage children to make different facial expressions and movements.
3. Show pictures of different people's faces one at a time. Ask the children, are they happy, sad, angry or hurting. Talk about what makes them look sad, happy, etc.
4. Show the feelings picture cards one at a time. Ask the children what does this face show?
5. Ask each child to point to point the picture that shows how they are feeling today. Encourage the children to say "today I am feeling........"
6. Play a matching game. Put four pictures in front of each child: Two pairs of picture cards. Ask the children to select the cards that are the same. You can make this harder by putting 3 or 4 pairs of pictures in front of the child.
7. Show the children the communication board. Explain how they can use the communication board to help express how they feel and what they need. Ask children to point to different pictures on the communication board.
8. Introduce signs for feelings and needs: water/drink, food, toilet etc.
9. Practice using communication board and signs to answer questions.
10. Give each child a copy of the cube template.
11. Ask the children to draw different expressions or needs on the squares. The children can also cut out the picture cards or pictures from magazines/newspapers and glue them on the squares.
12. Demonstrate how to cut out, fold and glue the template to make a cube.
13. Take turns rolling the 'feelings and needs' dices and reading the picture on top.

Cube Template

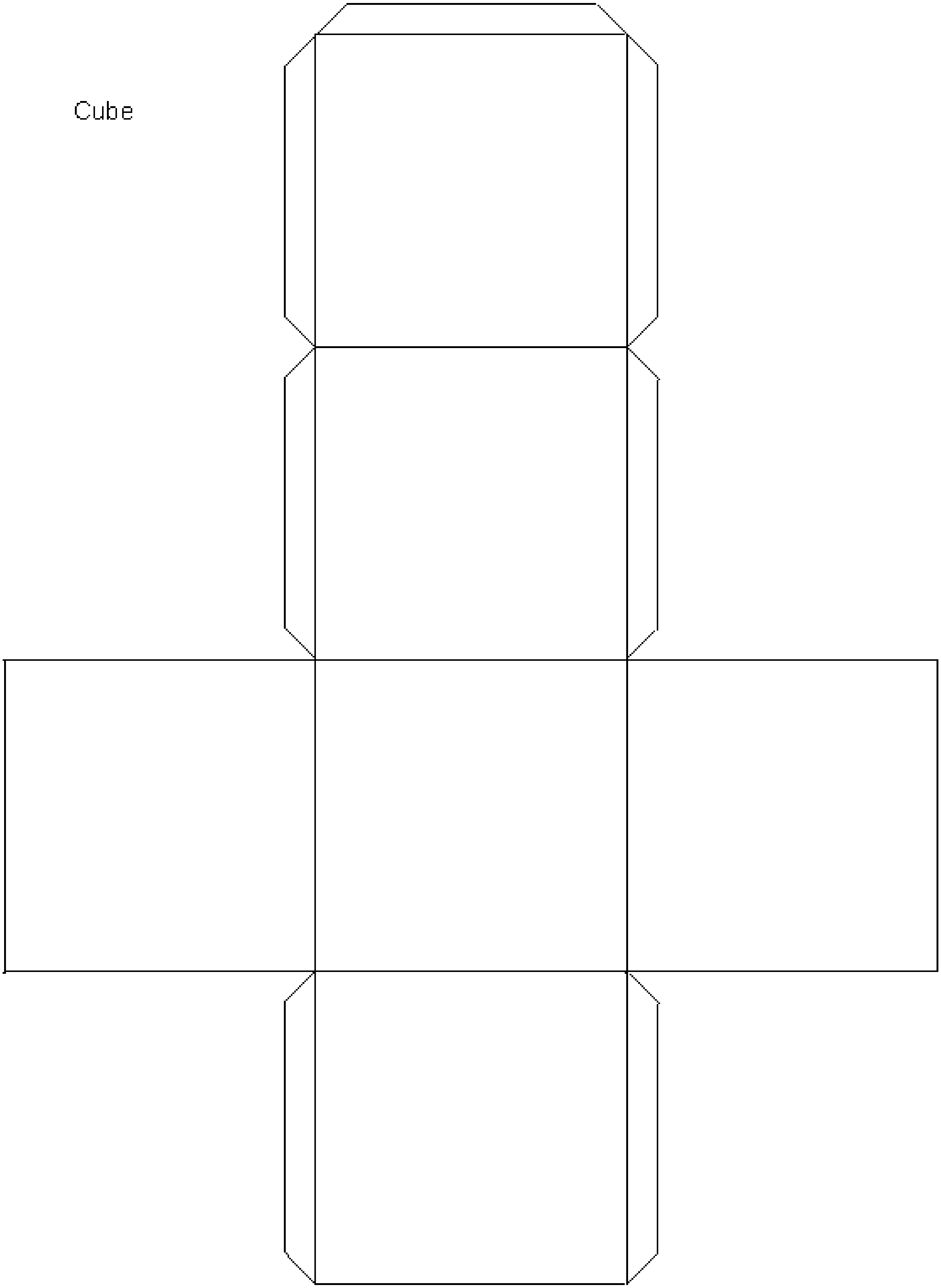

Class 31: Helping in the Kitchen

To develop:

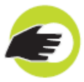

Arm and hand control: Grip strength, coordination

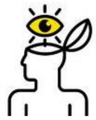

Perception: Determining differences and similarities in object shape, size etc, and understanding spatial relationship between objects

Need:

- Jars with different lids
- Bottles with different lids
- Bowl
- Mixing spoon
- Dry rice
- Jug
- Cup
- Bucket with water
- Plates and cups

Instructions:

1. Children rotate activities and practice the following:
   a. Opening and closing jar lids
   b. Opening and closing bottle lids
   c. Stirring a bowl with dry rice
   d. Shaking a bottle with dry rice
   e. Pouring water from a jug into a cup, or if that is too difficult, into a bowl.
   f. Lifting and carrying items of different sizes and shapes from one place to another.
   g. Washing and drying plates and cups used during nasta

Homework: Children practice these activities at home.

Class 32: Role Play, Families

To develop:

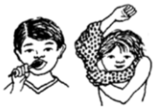

Activities of daily living: Dressing a doll, pretending to feed themselves/doll

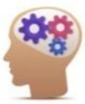

Cognition: Imaginary play, following instructions

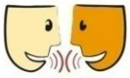

Language and communication

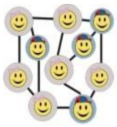

Social behaviour: Greetings, turn taking

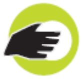

Arm and hand control

Need:

- Dolls and dolls clothes
- Sheets
- Towels
- Spoons and bowls
- Scooter boards
- Floor mats

Instructions:

1. Tell the children that today we are going to play pretend families. Get children to gather in groups of two, three or four and decide what roles each child will be in their family. Get the children to count how many members are in their family.
2. Give each group of children a dress-able doll that will be a child in the family.
3. Children can use materials around the room e.g. mats and sheets to make their home.
4. Using picture cards, tell children what is happening during their day e.g.:
    a. It's night-time, time to say goodnight to other people in your family and go to bed.
    b. It's morning, time to say good morning and time to wake up the child.
    c. It's time to take the doll child to the toilet, give it a wash, dress it, cook, feed the child etc.
    d. It's time for the father to go to work. The child can then move to another area and using blocks pretend to be building, or pretend to be working at the market etc.
    e. It's time for the mother to go shopping at the market. The child can then move to another area and collect items around the room, pretending they are food items to bring back to their home.
    f. Time for the children to go to school, go and play with their friends.
    g. It's time to make the bed, clean the house etc.
    h. The child can sit on scooter boards (and push themselves using their hands) to pretend to be taking a rickshaw/bus to their workplace/market.
    i. Finish with, it's been a busy day, it's now night-time and time for you all to go to sleep. Children lie down in their pretend homes.

Class 33: Making Nasta, Jhal Muri

To develop:

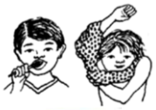

Activities of Daily Living: Basic food preparation and eating

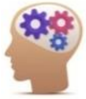

Cognition: Sequencing, construction

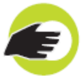

Arm and hand control: Grasp and release, cutting, peeling, scooping, pouring

Need:

- Puffed rice (Muri)
- Chickpea flour noodles (Sev)
- Fried lentils (Channa Dal)
- Crushed peanuts
- Mung bean sprouts
- Cilantro
- Coriander leaves
- Coconut meat
- Chopped ginger root
- Tomato
- Cucumber
- Chopped onion
- Tamarind sauce
- Lime
- Mustard oil
- Chaat masala
- Garam masala
- Chopping board/plate
- Large bowls
- Knife (ensure child supervised while using the knife)
- Spoon
- Small bowls or newspaper

Instructions:

1. All children wash their hands with soap
2. Write instructions on the board, or give the children visual instruction sheets and explain the instructions to the children:
   a. Ingredients:
      i. 1 cup puffed rice (Muri)
      ii. ¼ cup chickpea flour noodles (Sev)
      iii. ¼ cup fried lentils (Channa Dal)
      iv. ¼ cup crushed peanuts
      v. ½ cup mung bean sprouts
      vi. ¼ cup cilantro
      vii. ¼ cup coriander leaves
      viii. 1 tablespoon coconut meat
      ix. 1 tablespoon chopped ginger root
      x. 1 tomato
      xi. ½ cucumber
      xii. ¼ chopped onion
      xiii. 1 tablespoon tamarind sauce
      xiv. Lime juice of ¼ lime
      xv. 1 teaspoon mustard oil
   b. Chop cilantro, coriander leaves, coconut, tomato and cucumber and put into a large bowl.**
   c. Measure and add to the bowl: puffed rice, chickpea flour noodles, fried lentils, peanuts, mung bean sprouts, ginger, onions, tamarind sauce, mustard oil, chaat masala and garam masala
   d. Squeeze the juice of ¼ of a lime into the bowl
   e. Mix everything together using a spoon

   ** Child to measure and crush using hands and/or chop using a knife: cilantro, coriander leaves, and coconut. Ensure each child is closely supervised while using a knife. If child manages well using a knife, child can chop tomato and cucumber (with close supervision). Otherwise, adult should chop tomato and cucumber and then the child puts this into the bowl.
3. Children to spoon a little into a small bowl or a cone made from newspaper and eat for nasta!
4. Children to ask their parent, would you like some Jhal Muri? If they say yes, then the child spoons a little of the mixture into a small bowl or a cone from newspaper and hands it to their parent.
5. Children then serve themselves a little of the mixture and eat for nasta!

Homework: Children take home the remainder of the mixture to share with their family and friends. Children must ask their family and friends, "Would you like some Jhal Muri" and serve.

Class 34: My Story, How do I Spend Time with My Friends?

To develop:

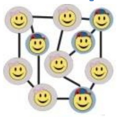

Social behaviour: Awareness of role as a friend

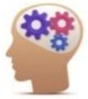

Cognition: Recalling activities with different friends

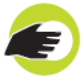

Arm and hand control: Writing, drawing, cutting, gluing

Need:

- My Story book
- Magazines/ newspapers
- Writing pencils
- Colouring pencils
- Glue
- Scissors

Instructions:

1. Write the heading on the board: How do I spend time with my friends?
2. Discuss with the children how you spend time with different friends, and write the answers on the board.
3. Tell children they will now make a collage showing how they spend time with their friends, in their My Story books.
4. Ask children to copy the heading into their books. If a child is unable to copy the heading, write the heading on a strip of paper and ask the child to glue the heading in their My Story book.
5. Instruct the children to cut out, or rip out pictures they find in magazines showing how they spend time with different friends, and glue the pictures in their book. The children can also draw pictures if they can't find anything in the magazines.
6. Children show and discuss their collage with the rest of the class.

Class 35: Friendship Necklaces

To develop:

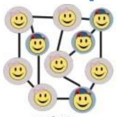

Social behaviour: Engagement in friendship role

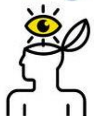

Perception: Understanding spatial relationship between objects

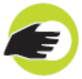

Arm and hand control: Threading, painting, gluing

Need:

- String
- Beads and/or dry pasta
- Paints
- Glitter and glue
- Painting smocks

Instructions:

1. Today we will make friendship necklaces. You will make two necklaces – one for you and one to give to your special friend.
2. Give each child a container with beads/dry pasta and a piece of string.
3. Children cut the string in half and thread beads/dry pasta to make two necklaces.
4. Once finished, children then paint the necklaces and decorate with glitter.

Homework: Children give a necklace to their special friend.

Class 36: Making a Pig Family and Friends

To develop:

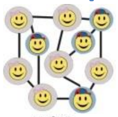

Social behaviour: Understanding of family and friends

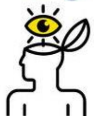

Perception: Understanding spatial relationship between objects

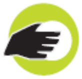

Arm and hand control: Threading, painting, gluing

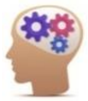

Cognition: Counting

Need:

- Selection of 2 Litre, 1 Litre, 500mL and 250mL plastic or cardboard bottles
- Toilet paper roles
- Cardboard
- Scissors
- Nuts or pebbles
- Markers

Instructions:

1. Give each child one bottle. The child will make a pig!
2. Children put a mark on the bottle where the four legs, one tail and two ears will go.
3. Adults help with cutting out four large holes for the legs, one small hole for the tail and two slits for the ears.
4. Children make the pig:
   a. Insert toilet paper rolls into the leg holes
   b. Make and insert a tail into the tail hole
   c. Make cardboard ears and insert them into the slits
   d. Draw on the eyes, ears, nose and mouth
   e. Put in nuts or pebbles through the nose
5. Put the pigs together. As a class discuss what roles each pig is: the mother, father, daughter, son, friend etc.
6. Use the pigs for counting – count the total number of pigs, count the number of pigs in the family, the number of friends, if one pig leaves to go to the market how many pigs are left, etc.

Class 37: People in My Life Puzzle

To develop:

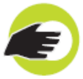

Arm and hand control: Drawing, cutting, gluing

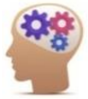

Cognition: Puzzles

Need:

- Paper
- Coloured pencils
- Craft materials – coloured paper, scrap material, glitter, newspaper/magazines etc.
- Glue
- Scissors

Instructions:

1. Ask children to draw a picture showing all the important people in their life: their family, and their friends. Tell them to make it as colourful as they can! They can use coloured pieces of paper and other craft materials.
2. Once finished, children show the rest of the class and tell them who is in their picture.
3. Ask the children to turn the picture into a puzzle, by cutting the picture into smaller squares:
    a. Start by cutting the picture in half. Then the children have to put the picture together.
    b. If that is easy, cut the picture into four pieces. Then put it together.
    c. If that is easy, cut the picture into eight pieces. Then put it together.
    d. Etc.

Homework: Children take home the puzzle and practice at [illegible]

Class 38: Write an Invitation Card

To develop:

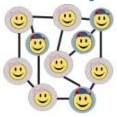

Social behaviour: Engagement in family role

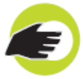

Arm and hand control: Threading, painting, gluing

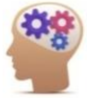

Cognition: Counting

Need:

- Paper
- Coloured pencils
- Craft materials – coloured paper, scrap material, glitter, newspaper/magazines etc.
- Glue
- Envelopes

Instructions:

1. Tell the children on Friday we will have a Family Day. You need to invite your family to come to Family Day. One way of doing this is making them an invitation card.
2. Show children an example of an invitation, and talk about what is included on the invitation. Make a list of what to include on the board e.g. name of person you are inviting, event, time and place, who the invitation is from etc.
3. Give each child a piece of paper. Ask them to copy headings from the board and fill in the spaces.
4. Children then decorate the invitation.
5. Children to put the invitation into an envelope, write the person's name on the front of the envelope, and decorate the envelope.

Homework: Children give the invitation to their

Class 39: My Story, My Hero

To develop:

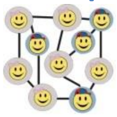

Social behaviour: Recognition of important people in our lives.

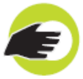

Arm and hand control: Drawing, writing.

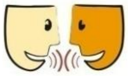

Language and communication: Verbal and visual communication, increase vocabulary.

Need:

- My Story books
- Pictures of famous Bangladeshi 'heroes'
- Writing pencils
- Colouring pencils
- Magazines/newspapers
- Glue
- Scissors

Instructions:

1. Discuss hero – what is a hero? Who are Bangladeshi heroes? Show pictures to the class and discuss. Discuss that family members and friends are those closest to you and are heroes.
2. Each child to write in their book the name of their hero. Children to draw a picture of their hero and show through pictures, and/or words why they are their hero.
3. Children to copy the heading 'My hero's autograph' into their books.

Homework: Children collect their hero's

Class 40: Family Day

To develop:

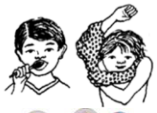

Activities of daily living: Basic food preparation.

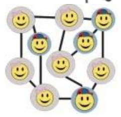

Social behaviour: Greetings and polite interactions.

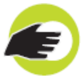

Arm and hand control: Chopping food, grasp and release, lifting and carrying.

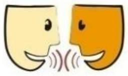

Language and communication: Verbal and visual communication, increase vocabulary.

Need:

- Nasta
- Plates
- Glasses
- Water
- Serving tray
- Serving trolley

Instructions:

1. Children to introduce members of their family to the rest of the class – their name, who they are in their family, what they do together, what they like etc.
2. Children to greet others' family members, introduce self, and ask 'how are you today'.
3. Children to ask 'would you like some nasta?' and 'would you like some water?'
4. Children to wash their hands with soap.
5. Children to serve nasta by putting food on individual plates, cutting up food, bringing glasses of water for the families etc.

Classes

Month 3: The World Around Me

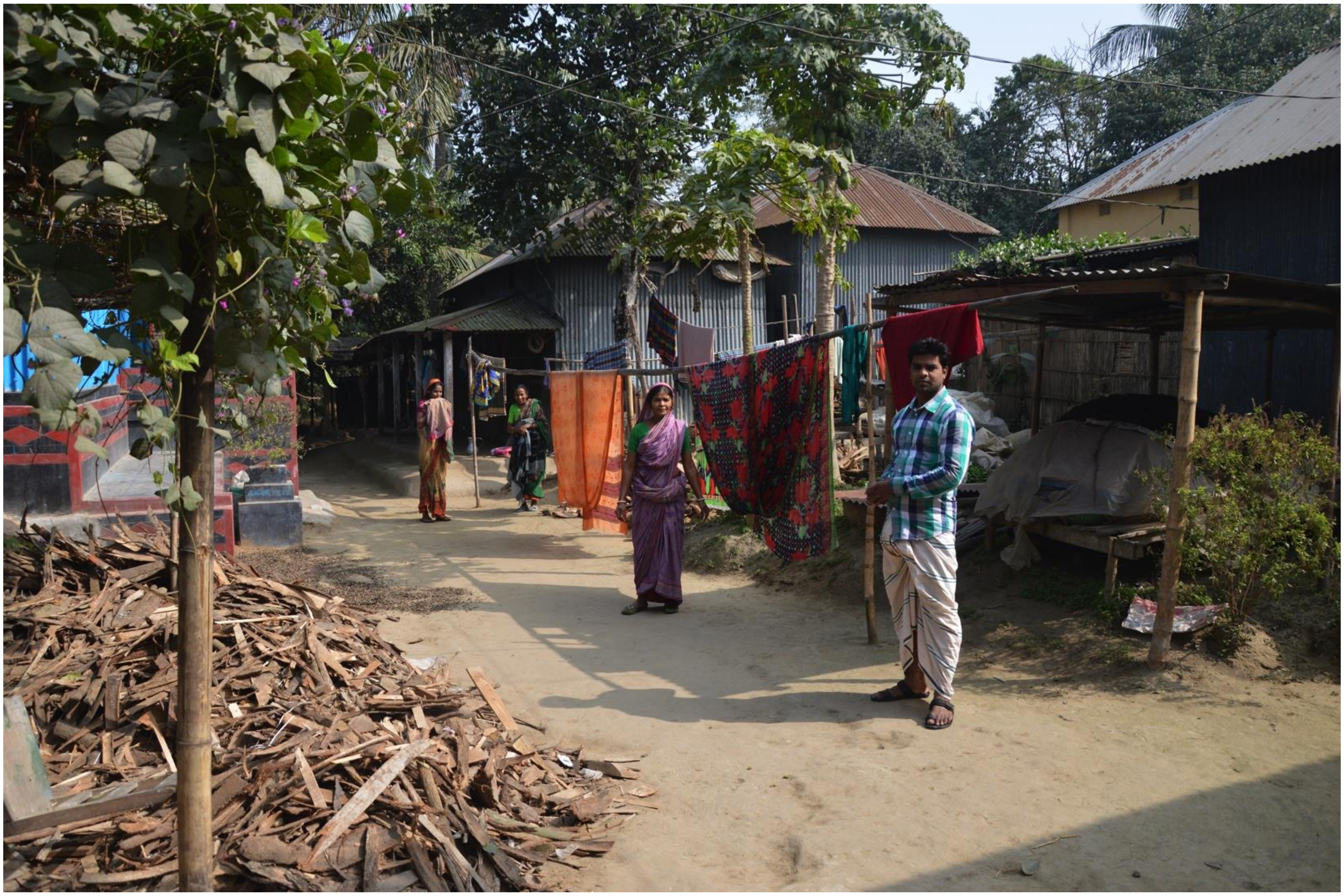

Class 41: Introduce theme, The World around Me

To develop:

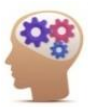

Cognition: Awareness and understanding of place and surroundings, counting

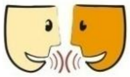

Language and communication: Describing their surroundings, vocabulary

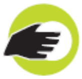

Arm and hand control: Drawing and writing

Need:

- Paper
- Colouring and writing pencils
- A4 stiff cardboard paper
- Glue

Instructions:

1. Introduce theme, the world around me.
2. Explain my community is where I am and what is around me. Discuss with the class where we all are – our country, district, town, our house, our school, etc. Discuss with the class what is around us – what buildings, trees, animals, etc. Write/draw answers on the board.
3. Give each child a piece of paper. Ask children to draw a map showing where they are. Where are the buildings, where is the road, where are the trees? Where is the office? Where is the gate? Where is the kitchen? Where is the corner shop? Draw on the piece of paper. They can move around outside the classroom to have a look.
4. Mark out an area in front of each child using chalk on pavement or sticks. Ask the children to build a miniature community using little boxes, figurines/other objects available to represent houses, people, animals, and plants.
5. Give each child pieces of A4 stiff cardboard paper. Ask the children to search for numbers of different things in the environment as you call them out. The children are then to paste these on their piece of cardboard. E.g. Ask the children to find a paste on their paper: one paper clip, 2 sweet papers, 3 leaves, 4 stickers, 5 small stones. Ask the children to add the number of objects and write the number beside the objects.

Class 42: Alphabet and Numbers

To develop:

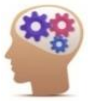

Cognition: Alphabet, numbers and counting

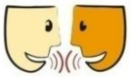

Language and communication: Pre-reading skills, vocabulary

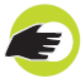

Arm and hand control: Drawing and writing

Need:

- Alphabet poster, or write the alphabet on the board
- Handwriting sheets (photocopies of handwriting books)

Instructions:

1. Use a poster of the alphabet, or write the alphabet on the board.
2. As a class, recite the alphabet. Point to each letter as you say the letter.
3. Point to a letter and ask children to make the sound.
4. Play a game where you select a letter and ask a child to say a word that has that sound.
5. Practice writing letters using handwriting sheets.
6. Count 1 to 10. (Or more if this is easy).
7. Give each child three to ten items e.g. blocks, basket of balls. Ask children counting questions, for example:
   a. How many items do they have in total
   b. How many blue items are there
   c. Pass me five balls, four yellow balls
8. Practice writing numbers using handwriting sheets.
9. Using number flash cards, get the children to put them in order, fill in the missing number and build it backwards.

Class 43: What do I see at the Fruit Market?

To develop:

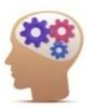

Cognition: Alphabet, recognition of the characteristics of different fruits, recall

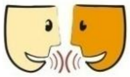

Language and communication: Pre-reading skills, vocabulary

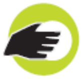

Arm and hand control: Writing

Need:

- Variety of different fruits
- Handwriting worksheets
- Writing pencils
- Scales to measure the weight of fruits

Instructions:

1. Tell children tomorrow we will go on an excursion to the fruit market.
2. Ask children what do you think you will see at the fruit market? Ask them to say as many different names of fruit as they can. What else will you see at the fruit market? (e.g. shopkeeper)
3. Show them different fruits. As you show each fruit, write the name of the fruit on the board. Say the sounds the letters make. Ask children to write the name of this fruit in their books. Use handwriting worksheets to practice writing the letters.
4. Put two different fruits in front of each child. Ask the children to count how many fruits are in front of them. Ask each child to select the fruit which is the biggest. Then ask them to select which fruit is the smallest. Get the children to weigh the fruits and get the children to read out the number on the scales. Ask them which fruit is the heaviest? Which fruit is the lightest? What colours are the fruit? Do the fruits feel hard or soft? Etc.

Class 44: Excursion to the Fruit Market

To develop:

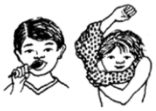

Activities of daily living: Purchasing items from the market

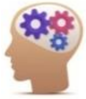

Cognition: Characteristics of items and awareness of surroundings, counting

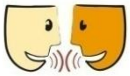

Language and communication: Asking and understanding need and cost

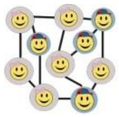

Social behaviour: Greetings, turn taking

Need:

- Assistive devices for mobility
- Rickshaw transport
- Communication cards, chat books
- Bag to carry fruits in

Instructions:

1. Tell children today we are going on an excursion to the fruit market, and you need to tell me what you see.
2. Ask children to greet the shopkeeper.
3. Ask children, what fruits do you see? Ask children to count, and describe different fruits.
4. Ask children, what else do you see? Who do you see here? If you want to buy something what would you do?
5. Get children to decide what fruit they want to buy for nasta tomorrow, or provide them a shopping list using words and pictures, e.g. two bananas, one apple, 100 gram of grapes and one lime. Get the children to count how many pieces of fruit they will need. The children will count the number of fruits as the shopkeeper puts it on the scales/in a bag. Watch the shopkeeper weigh the fruit and have the children read the weight on the scales. Negotiate who is in charge of paying, receiving the change. Have all the children involved in counting the money.   Negotiate who will carry the fruits safely back.
6. During the travel to and from the market place, discuss with the children different life skills. E.g. what to do if you were lost, how to ask for help, what to say, knowing your name, parents name, village name, school name etc. A chat book is useful for this for those children who have difficulty speaking.

Class 45: My Story, My Neighbourhood

To develop:

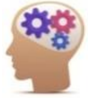

Cognition: Awareness and understanding of place and surroundings, recall

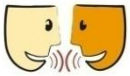

Language and communication: Vocabulary

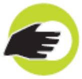

Arm and hand control: Drawing and writing

Need:

- Paper
- Coloured pencils or crayons
- Old magazines or newspapers
- Scissors
- Glue

Instructions:

1. Discuss with the children the characteristics of their neighbourhood asking questions such as:
   a. Are there houses, buildings, or both in your neighbourhood? What do they look like?
   b. Are there farms, open spaces, or markets near your house?
   c. What kinds of animals live in your neighbourhood?
2. Have each child draw a map of their neighbourhood. They can search, cut-out and paste on their piece of paper pictures from magazines. The map should show:
   a. The house or the building he or she lives in
   b. Neighbouring houses
   c. Roads
   d. Shop
   e. Market
   f. Farm
   g. Trees
   h. Flowers
   i. People
   j. Rickshaws
   k. Etc.
3. Have children share their finished pictures with the rest of the class. What are the similarities and differences?

Class 46: Alphabet and Numbers

To develop:

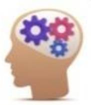

Cognition: Alphabet, numbers and counting

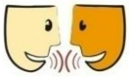

Language and communication: Pre-reading skills, vocabulary

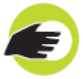

Arm and hand control: Drawing and writing

Need:

- Alphabet poster, or write the alphabet on the board
- Handwriting sheets (photocopies of handwriting books)

Instructions:

1. Use a poster of the alphabet, or write the alphabet on the board.
2. As a class, recite the alphabet. Point to each letter as you say the letter.
3. Point to a letter and ask children to make the sound.
4. Play a game where you select a letter and ask a child to say a word that has that sound.
5. Practice writing letters using handwriting sheets.
6. Count 1 to 20. (Or more if this is easy).
7. Give each child three to ten items e.g. blocks, basket of balls. Ask children counting questions, for example:
    a. How many items do they have in total
    b. How many blue items are there
    c. Pass me five balls, four yellow balls
8. Practice writing numbers using handwriting sheets.
9. Using number flash cards, get the children to put them in order, fill in the missing number and build it backwards.

Class 47: Money

To develop:

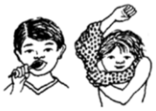

Activities of daily living: Handling money

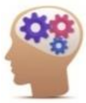

Cognition: Money, counting

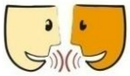

Language and communication: Asking and understanding cost

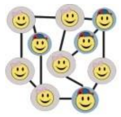

Social behaviour: Turn taking

Need:

- Paper money

Instructions:

1. Show children a Takka note at a time, and ask children to say or show using sign how much the note is worth.
2. Put a variety of Takka notes in front of each child. Ask the child to select the 5 Takka note etc.
3. Put three notes in front of each child – two that are the same and one that is different. Ask the child which notes are the same? Which note is different?
4. Instruct children to work in pairs. Give each pair an item. Tell the children to take turns pretending one person is a shopkeeper and the other is the customer. The shopkeeper reads the number off a card how much the toy is and tells the customer. The customer has to give the correct money.
5. To increase the difficulty, the shopkeeper has to give change.

Class 48: What do I see at the Vegetable Market?

To develop:

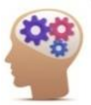

Cognition: Alphabet, recall, recognition of the characteristics of different fruits

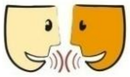

Language and communication: Pre-reading skills, vocabulary

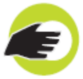

Arm and hand control: Writing

Need:

- Variety of different vegetables
- Handwriting worksheets
- Writing pencils
- Scales

Instructions:

1. Tell children tomorrow we will go on an excursion to the vegetable market.
2. Ask children what do you think you will see at the vegetable market? Ask them to say as many different names of vegetables as they can. What else will you see at the vegetable market? (e.g. shopkeeper)
3. Show them different vegetables. As you show each vegetable, write the name of the vegetables on the board. Say the sounds the letters make. Ask children to write the name of this vegetable in their books. Use handwriting worksheets to practice writing the letters.
4. Put two different vegetables in front of each child. Ask the children to count how many vegetables are in front of them. Ask each child to select the vegetable which is the biggest. Then ask them to select which vegetable is the smallest. Get the children to weigh their vegetables on the scales. Ask them which vegetable is the heaviest? Which vegetable is the lightest? What colours are the vegetables? Do the vegetables feel hard or soft? Does the outside feel smooth or rough? What shape is the vegetable? Etc.
5. Ask other questions to practice counting e.g. how many vegetables are there in total. Pass five vegetables. Etc.
6. Ask children to work out how many of the same vegetable they would need to make 100 grams, 200 grams etc.

Class 49: Excursion to the Vegetable Market

To develop:

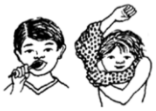

Activities of daily living: Purchasing items from the market

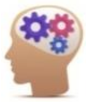

Cognition: Characteristics of items and awareness of surroundings, counting

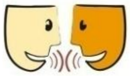

Language and communication: Asking and understanding need and cost

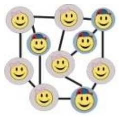

Social behaviour: Greetings, turn taking

Need:

- Assistive devices for mobility
- Rickshaw transport

Instructions:

1. Tell children today we are going on an excursion to the vegetable market, and you need to tell me what you see.
2. Ask children to greet the shopkeeper.
3. Ask children, what vegetables do you see? Ask children to count, describe different vegetables.
4. Ask children, what else do you see? Who do you see here? If you want to buy something what would you do?
5. Get children to decide what vegetables they want to buy for nasta tomorrow, or provide them a shopping list using words and pictures, e.g. two cucumbers, one lettuce, 100 gram of tomatoes and one lime. Get the children to count how many pieces of fruit they will need. The children will count the number of vegetables as the shopkeeper puts it on the scales/in a bag. Watch the shopkeeper weigh the vegetables and have the children read the weight on the scales. Negotiate who is in charge of paying, receiving the change. Have all the children involved in counting the money.   Negotiate who will carry the vegetables safely back.
6. During the travel to and from the market place, discuss with the children different life skills. E.g. what to do if you were lost, how to ask for help, what to say, knowing your name, parents name, village name, school name etc. A chat book is useful for this for those children who have difficulty speaking.

Class 50: Role play, Market Place

To develop:

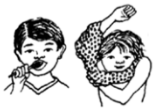

Activities of daily living: Buying items from the market, handling money

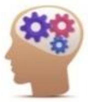

Cognition: Providing correct money and change

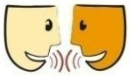

Language and communication: Asking and understanding need and cost

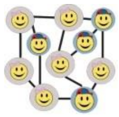

Social behaviour: Greetings, turn taking

Need:

- Variety of items – toys, clothing, etc.
- Scales
- Pretend/real money

Instructions:

1. Instruct the children to collect items around the room that they can then sell at their shop. Children then place the items they collected in front of them – either on a table, or on a mat. The children can set up their shop inside or outside.
2. Children sit behind their shop front as shopkeepers and say what type of shop they have e.g. selling plants or toys or fruit.
3. Tell one child they need to go to another shop to buy something e.g. grapes.
4. Becoming the customer, that child must move from their shop, and go around to the other shops asking if they have grapes available. When they reach the shop that has grapes, they need to say how many grapes they need to buy, ask the price, and then pay the money.
5. The shopkeeper must say the price, count/weigh the items as they put it in the bag, receive money and give the required change.
6. Continue so that all children have turns at being the shopkeeper and customer.

Class 51: Shopping

To develop:

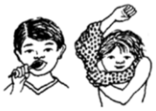

Activities of daily living: Buying items from the market, handling money

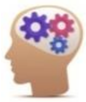

Cognition: Providing correct money and change

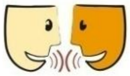

Language and communication: Asking and understanding need and cost

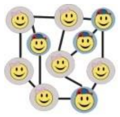

Social behaviour: Greetings, turn taking

Need:

- Paper
- Writing pencils
- Assistive devices for mobility
- Rickshaw transport

Instructions:

1. Tell the class that tomorrow they are going to make a fruit salad.
2. The children must choose the ingredients for the fruit salad and go shopping.
3. As a class, make a shopping list by writing the list on the board.
4. Instruct children to say and write down the fruit they would like to buy and how much they need.
5. Go to the fruit market and buy the fruit. Children take turns in asking for different fruit, and how much it is.
6. Children handle the money and carry it back to the centre ready for the next day.

Class 52: Fruit Salad

To develop:

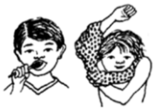

Activities of daily living: Purchasing items from the market

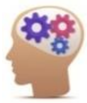

Cognition: Characteristics of items and awareness of surroundings, counting

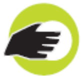

Arm and hand control: Peeling and chopping

Need:

- Fruits purchased the previous day
- Plates/chopping board/table
- Knife (provide close supervision)
- Peeler (if needed)
- Large bowl
- Picture cards of instructions

Instructions:

1. Write the instructions on the board. Give visual instruction sheets (using pictures) to children who have trouble understanding and remembering verbal instructions. Explain the instructions to the class and ask the children to follow the instructions and make their fruit salad as a class. Allocate different fruits to different children to peel and chop up. Provide close supervision while children are using a knife to chop up the fruit.
    a. Children to peel and chop up fruit.
    b. Children to put all fruit into a large bowl.
    c. Use a spoon to add yoghurt to the fruit.
    d. Mix.
    e. Serve fruit salad into smaller bowls/plates
    f. Eat
    g. Squeeze orange into a glass
    h. Drink
2. Clean up! Instruct children to do different activities to help with the clean up.

Class 53: What do I see at the Clothes Market?

To develop:

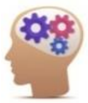

Cognition: Alphabet, recognition of the characteristics of different clothing items and materials, recall

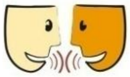

Language and communication: Pre-reading skills, vocabulary

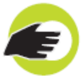

Arm and hand control: Writing

Need:

- Range of different clothing and materials
- Handwriting worksheets
- Writing pencils

Instructions:

1. Ask children, what do you think you will see at the clothes market? Ask them to name as many different types of clothing, and materials as they can. What else will you see at the clothes market?
2. Show them different materials and items of clothing. Ask children to hold, feel the different fabrics, and name and describe the fabrics and clothing. Write names of the clothing items on the board. Say the sounds the letters make. Ask children to write the clothing names in their books. Use handwriting worksheets to practice writing the letters.
3. Practice putting on different items of clothing. Play the 'What will I wear today?' game (Class 16).

Class 54: Excursion to the Clothes Market

To develop:

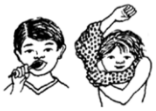

Activities of daily living: Purchasing items from the market

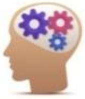

Cognition: Characteristics of items and awareness of surroundings, counting

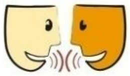

Language and communication: Asking and understanding need and cost

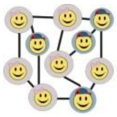

Social behaviour: Greetings, turn taking

Need:

- Assistive devices for mobility
- Rickshaw transport
- Notebook
- Writing pencils

Instructions:

1. Tell children today we are going on an excursion to the vegetable market, and you need to tell me what you see.
2. Ask children to greet the shopkeeper.
3. Ask children, what clothing and materials do you see? Ask children to describe different materials and clothing items.
4. Ask children, what else do you see? Who do you see here?
5. Each child to ask the shopkeeper how much one item of clothing is. The children have to write down the price in their book.
6. Back in the classroom, compare prices. What item was the most expensive? What item was the cheapest?

Class 55: Making Fabric Dolls

To develop:

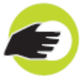

Arm and hand control: Drawing, needlework

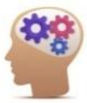

Cognition: Sequencing the task

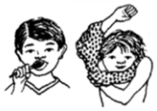

Activities of daily living: Sewing

Need:

- Paper
- Material pieces
- Needle and thread
- Marker

Instructions:

1. Draw an outline of a doll on a piece of paper and cut it out
2. Trace around the paper outline on two pieces of material
3. Cut out the material outline
4. Sew the two dolls together, leaving a small opening
5. Turn the doll inside out
6. Stuff the doll with material scraps or newspaper/tissues
7. Sew together the opening
8. Draw on a face
9. Using other pieces of fabric, dress the doll in a Sari or lungi and shirt

Class 56: Role play, Market Place

To develop:

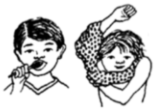

Activities of daily living: Purchasing items from the market

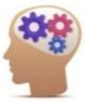

Cognition: Counting, providing correct money and change

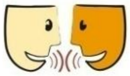

Language and communication: Greetings, requesting and selling items

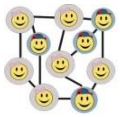

Social behaviour: Greetings, turn taking

Need:

- Variety of items – toys, clothing, etc.
- Scales
- Pretend/real money

Instructions:

1. Instruct the class to set up their shops. Instruct the children to collect items around the room that they can then sell at their shop. Children then place the items they collected in front of them – either on a table, or on a mat. The children can set up their shop inside or outside.
2. Children sit behind their shop front as shopkeepers and say what type of shop they have e.g. selling plants or toys or fruit.
3. Tell one child they need to go to another shop to buy something e.g. grapes.
4. Becoming the customer, that child must move from their shop, and go around to the other shops asking if they have grapes available. When they reach the shop that has grapes, they need to say how many grapes they need to buy, ask the price, and then pay the money.
5. The shopkeeper must say the price, count/weigh the items as they put it in the bag, receive money and give the required change.
6. Continue so that all children have turns at being the shopkeeper and customer.

Class 57: Shopping

To develop:

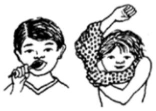

Activities of daily living: Buying items from the market, handling money

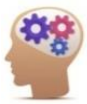

Cognition: Providing correct money and change

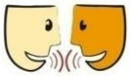

Language and communication: Asking and understanding need and cost

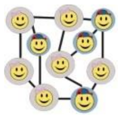

Social behaviour: Greetings, turn taking

Need:

- Paper
- Writing pencils
- Assistive devices for mobility
- Rickshaw transport

Instructions:

1. Tell the class that tomorrow they are going to make a vegetable salad.
2. The children must choose the ingredients for the vegetable salad and go shopping.
3. As a class, make a shopping list by writing the list on the board. Make sure you only choose vegetables that don't need cooking.
4. Instruct children to say and write down the vegetables they would like to buy and how much they need.
5. Go to the vegetable market and buy the vegetables. Children take turns in asking for different vegetables, and how much it is.
6. Children handle the money and carry the vegetables back to the centre ready for the next day.

Class 58: Vegetable Salad

To develop:

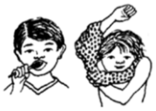

Activities of daily living: Purchasing items from the market

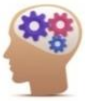

Cognition: Characteristics of items and awareness of surroundings, counting

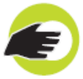

Arm and hand control: Peeling and chopping

Need:

- Vegetables purchased the previous day
- Plates/chopping board/table
- Knife (provide close supervision)
- Peeler
- Large bowl

Instructions:

1. Write the instructions on the board. Explain the instructions to the class and ask the children to follow the instructions and make their vegetable salad as a class. Give children who have difficulty understanding visual instruction sheets (using pictures). Allocate different vegetables to different children to peel and chop up. Provide close supervision while children are using a knife to chop up the vegetables.
    a. Children to peel (if suitable) and chop up vegetables.
    b. Children to put all vegetables into a large bowl.
    c. Squeeze in lime over the vegetables.
    d. Mix.
    e. Serve vegetable salad into smaller bowls/plates
    f. Eat
2. Clean up! Instruct children to do different activities to help with the clean up.

Class 59: Excursion to the Field and River, I Spy Game

To develop:

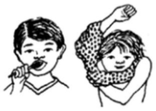

Activities of daily living: Purchasing nasta

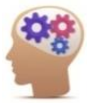

Cognition: Awareness of surroundings, recognition of colours, giving correct money, counting

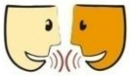

Language and communication: Communicating need, naming surroundings

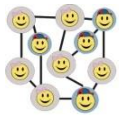

Social behaviour: Greetings, turn taking

Need:

- Money to purchase nasta
- Money for a boat ride on the river

Instructions:

1. If there is a children's park/playground in the village, go to the park for the session. Otherwise, go on an excursion to an open field.
2. While at the field, play the 'I Spy' game. To play 'I Spy', one persons starts by looking around a choosing something for the other children to guess, e.g. a tree. Then you say: "I spy with my little eye something.... green". The other children then have to look around and take turns guessing what it could be e.g. "Is it grass?" Make sure all children have a turn guessing. Those children who have difficulty communicating verbally can point or use sign language. The child who guesses correctly then has to choose something for the others to guess.
3. Children must order some street food for nasta. They must visit the stall, greet the walla, ask for their nasta and pay the money.
4. Take the children for a boat ride on the river. Play the 'I Spy' game. Name what you see on the river. Count the number of boats/cows in a field beside the river etc.

Class 60: My Story, My Role in My Community

To develop:

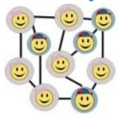

Social behaviour: Awareness of role as a member of the community

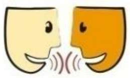

Language and communication: Communicating role in the community through written and spoken language and pictures

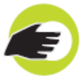

Arm and hand control: Writing, drawing, cutting and pasting

Need:

- My Story books
- Writing pencils
- Colour pencils
- Magazines/newspapers
- Scissors
- Glue

Instructions:

1. Talk about how we interact with our community. What do we do in our community? What have we done in this last month in our community? Discuss with the children and write points on the board.
2. Ask children to write a heading in their books, 'My Role in My Community'. They can then write words, draw pictures, and cut and paste pictures from magazines/newspapers in their book.
3. Once finished, show to the rest of the class and discuss.

Homework: Children to be active in their community. Parents to let their child help with shopping, take them to play in a field with other children, etc. Parents to ENCOURAGE and FACILITATE their child's participation in their homes and community.

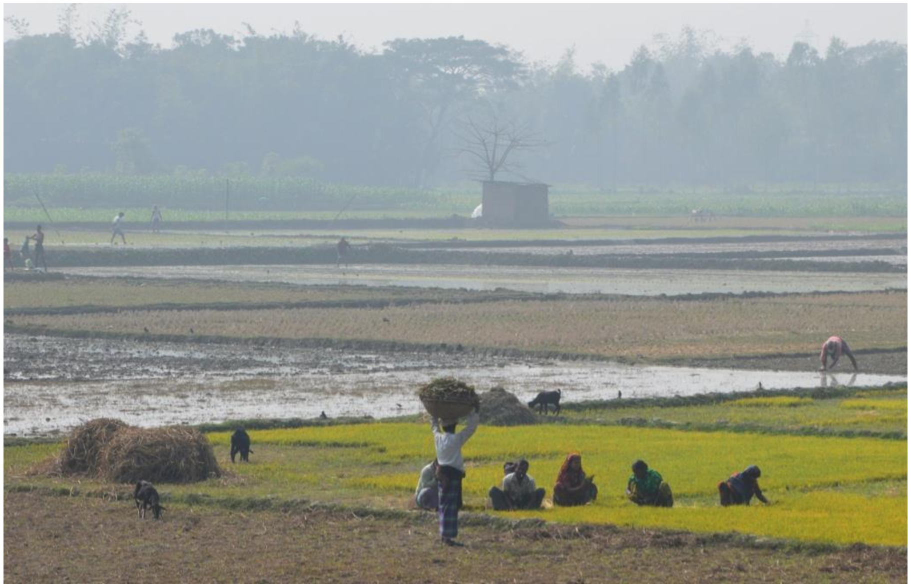

Classes

Month 4: School and Productivity

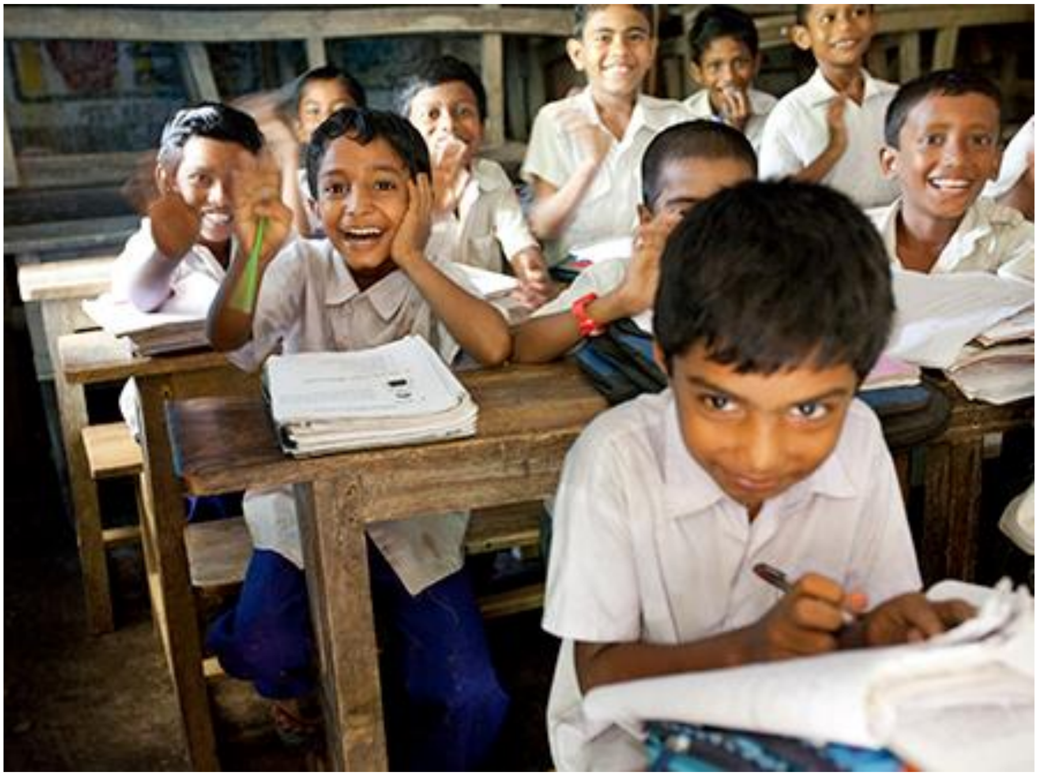

Class 61: My Story, School and Productivity

To develop:

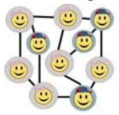

Social behaviour: Awareness and understanding of roles as student, helper and worker

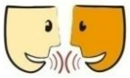

Language and communication: Describing their surroundings, vocabulary

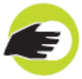

Arm and hand control: Drawing and writing

Need:

- My Story
- Colouring and writing pencils
- Magazines/newspapers
- Glue

Instructions:

1. Introduce theme, School and Productivity.
2. Brainstorm with the class, what do you do at school? What do you need to go to school? Write on the board.
3. Brainstorm with the class, how can you help others? Who can you help? Write on the board.
4. Brainstorm with the class, what jobs do people do? Write on the board.
5. Write the headings on the board:
    a. At school I would like to learn about:
    b. At home I will help others by:
    c. What I would like to be when I grow up:
6. Ask children to copy the headings in their My Story book. For those children unable to write sentences, ask the adults in the room to copy these headings on strips of paper, for the children to glue in order in their books
7. Ask children to write words, draw pictures, cut and paste pictures from magazines and newspapers in response to each heading.

Class 62: Alphabet and Numbers

To develop:

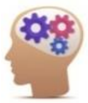

Cognition: Alphabet, numbers and counting

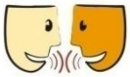

Language and communication: Pre-reading skills, vocabulary

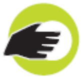

Arm and hand control: Drawing and writing

Need:

- Alphabet poster, or write the alphabet on the board
- Handwriting sheets (photocopies of handwriting books)

Instructions:

1. Use a poster of the alphabet, or write the alphabet on the board.
2. As a class, recite the alphabet. Point to each letter as you say the letter.
3. Point to a letter and ask children to make the sound.
4. Play a game where you select a letter and ask a child to say a word that has that sound.
5. Practice writing letters using handwriting sheets.
6. Count 1 to 30. (Or more if this is easy).
7. Give each child three to ten items e.g. blocks, basket of balls. Ask children counting questions. Eg:
    a. How many items do they have in total
    b. How many blue items are there
    c. Pass me five balls, four yellow balls
8. Practice writing numbers using handwriting sheets.
9. Using number flash cards, get the children to put them in order, fill in the missing number and build it backwards.

Homework: Bring a toothbrush and hairbrush tomorrow for [illegible]

Class 63: Helping at Home, Getting Dressed and Ready for My Day

To develop:

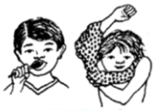

Activities of daily living: Dressing, washing face, brushing teeth, brushing hair

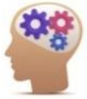

Cognition: Dressing for the weather, sequencing

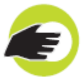

Arm and hand control: Doing up and undoing buttons and zips

Need:

3 of each:

- Jumpers
- Jackets
- Socks
- Shawl
- Umbrellas
- Raincoat
- T-shirt
- Shorts
- Salwar Kameez
- Hat

- Weather picture cards
- Whiteboard marker and whiteboard
- Children to complete homework – bring their toothbrush and hairbrush
- White-board and marker
- Activities of daily living picture cards
- Tubes of toothpaste
- Blue-tack
- Bucket
- Washcloth x 2
- Towel x 2

Instructions:

1. Talk about how you spend time with your family getting ready for your day. You can be helpful and more grown up by trying to do as much as you can yourself to get ready. This includes getting dressed, washing your face, brushing your teeth and brushing your hair.

Getting Dressed for the Weather:

2. Talk to the group about the different weather conditions. Ask the children, what are the different seasons? Write these on the board. Then ask the children, during the winter season is it hot or cold? What do you wear to try and stay warm? Write these on the board and show the different items of clothing to the group. Ask; during the summer season is it hot or cold? What do you wear to try and stay cool? Again write these on the board and ask the class to pick up the items of clothing they would wear from the pile and show the rest of the class. Discuss the other seasons, temperatures and what you would wear.
3. Talk about the different items of clothing. Hold up a hat. Ask the class, what is this for? How do you wear this? What body part do you put this on? Do boys, girls or both boys and girls wear this? Do this for each item of clothing.
4. Introduce the game to the group: We are now going to play a game. I will say what the weather is like today, and I will show you a picture of the weather today. Then you have to choose one item of clothing that you would need to wear to keep warm, cool, or dry, and put it on. You need to put the item on yourself!
5. Hold up one picture card, say 'The weather today is.....' and put the card on the board. Allow enough time for all children to finish the activity. Only provide prompts and assistance if/when the child doesn't initiate the task, or the task is too difficult. Only provide assistance to make the task easier, do not do the task for the child. Make sure the child is challenged and participating!
6. Continue this for all weather cards. Repeat weather cards more than once.

Washing Face, Brushing Teeth and Brushing Hair:

7. Talk about how you spend time with your family getting ready for your day. You can be helpful and more grown up by trying to do as much as you can yourself to get ready. For instance, choosing what you will wear for the day and getting dressed.
8. Discuss with the group that routine means the activities that we must do every day or regularly, and what order to we do these activities in. Ask the group, "What do you do every day in the morning?" Write a list on the board. Use communication boards and picture cards to help include children who have difficulty verbally communicating.
9. Using the activities of daily living picture cards: getting up out of bed, washing face, brushing teeth, brushing hair, eating and leaving home, ask the children to put the cards in order of what they did this morning. Provide prompts as required.
10. Get the children to say/show the class their sequence.
11. Sing the song, "This is the way we.... wash our face, brush our teeth, yawn and stretch, eat our nasta", etc. Show the children actions and instruct the children to copy these actions.
12. Tell the children that now we'll practice some of the things you do in the morning. Tell the children to show your mum's how grown up and helpful they are by doing it yourself! Today we're going to put on our shoes, wash our face, brush our teeth and brush our hair.
13. Instruct the children to find their shoes and put their shoes on independently or with minimal help (set up the task so that the child is being challenged, but is able to complete some of the steps independently, e.g. identifying their shoe, slipping their foot into the shoe, opening and closing the Velcro shoe. Instruct the children to them move to the bathroom tap and wait in a straight line.
14. Have a bucket with water and plastic chair set-up to use for those children who cannot reach the sink or stand independently. Have a wash cloth in the bucket and beside the sink, and a tube of toothpaste and dry towel beside the sink and bucket.
15. One by one, ask the children to wash their face and brush their teeth.
    a. For children able to stand and reach the sink: This is the sequence for the children to complete as independently as possible: Stand in front of the sink, turn on the tap, splash the water on their face and use the wash cloth (if they usually use this at home), dry their face, put toothpaste on their toothbrush, brush their teeth, spit, wash their mouth, turn off the tap and dry their mouth.
    b. For children unable to reach the sink, but able to stand: This is the sequence for children to complete as independently as possible: Stand beside the bucket, turn on the tap to put water in the bucket, turn off the tap, splash the water on their face and use the wash cloth (if they usually use this at home), dry their face, put toothpaste on their toothbrush, brush their teeth, spit, wash their mouth, and then dry their mouth.
    c. For children unable to stand: Get them to sit on a plastic chair beside the bucket, turn on the tap to put water in the bucket, turn off the tap, splash the water on their face, and use the wash cloth (if they usually use this at home), dry their face, put toothpaste on their toothbrush, brush their teeth, spit, wash their mouth, and then dry their mouth.
16. Once finished washing their face and brushing their teeth, instruct the child to return to the classroom, take off their shoes.
17. Ask children to pick up/sit beside a mirror, hold their hairbrush and brush their hair.
18. Once all children are finished, say now we are ready to start the day. Talk about how they can do this every morning, as much as they can by themselves.

The key points to helping a child gain independence in activities of daily living are:

- Be patient, give the child TIME to think through and attempt/complete steps of the task themself.
- Recognise and acknowledge a child's attempts and ability to complete steps of the task independently. E.g. a child may not be able to turn the tap on enough for a steady stream of water to come out, but they may be able to reach out, loosely grasp the tap, and give one turn of the tap. With practice and over time, the child will be able to grasp the tap a little firmer, and turn further.
- Do NOT take over and do the task for them. If the task is too difficult for them, think why is it too difficult? Is the child trying to move a tight tap? Then, just loosen the tap a little and get the child to try again. FACILITATE ability.
- Provide prompts only if the child is not initiating or skips a step of the task, i.e. only provide a prompt if it is needed, and do not continually prompt the child through a task. Provide a prompt and then give the child time to think and do.
- Only provide assistance to facilitate ability, and make the step of the task achievable for the child. Make sure that you don't provide so much assistance that the child is not actively participating. Ensure that the step of the task is still a challenge, but provide assistance so that it is achievable for the child. The child needs to do AS MUCH as they can themselves, and ALWAYS actively participate

Homework: Children to get ready for their day as independently as possible at

Class 64: Science, Make your own Quick Sand

To develop:

School skills: Science

Cognition: Sequencing, cause and effect, measuring

Arm and hand control: Pouring, stirring

Need:

For each child:

- 1 cup of maize corn flour
- Half a cup of water
- Large plastic container
- Spoon

Instructions:

1. Explain that at school you learn about science. Explain what science means: Science is the study of the world through experiment. The world is composed of animal, water, soil and plants among others all of which are being studied in science.
2. Today we will do an experiment – we will use corn flour and water to make quicksand! Explain and discuss what quicksand is: Quicksand is a thick, liquid mixture of sand and water. It is kind of like watery soil that can't support any weight. But don't worry -- if you ever happen to fall into quicksand, you won't get buried alive!
3. Set-up for the experiment outside. This experiment is messy.
4. Instruct the children to fill one cup with corn flour and put the flour in the container.
5. Instruct the children to fill half the cup with water and pour it into the container.
6. Children then stir and watch what happens.
7. Continue stirring until the mixture is hard.
8. Put different objects, with different weights and watch to see if they sink in the quicksand!

Teacher's Homework: Organise school visit. Speak with school about visiting a school and interviewing one school student.

Class 65: Excursion to School

To develop:

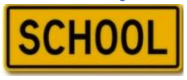

School skills: Familiarity with school grounds and structure

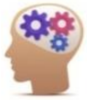

Cognition: Awareness of surroundings, recognition of colours, giving correct money

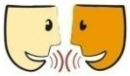

Language and communication: Communicating need

Need:

- Assistive devices for mobility
- Rickshaw transport
- Notebooks
- Writing pencils

Instructions:

1. Explain to the children that today we will visit one of the local schools. We will see what the school looks like, and interview one of the students about what you do at school.
2. As a class, prepare interview questions:
   a. What time do you have to wake up?
   b. What time do you go to school?
   c. What classes do you do at school? What do you do during class?
   d. What is your favourite class?
   e. What time is break? What do you do during your break?
   f. What time do you finish school?
3. Ask children to write the interview questions in their notebook. For those children unable to write sentences, write the questions on strips of paper for children to put in order and glue into their notebook.
4. At the school, observe the surroundings. What do you see? Name what you see – buildings, stairs, grass, playground, colours etc. Ask children to write down part or all of the words in their books.
5. Interview a student. Children take turn asking the questions. Children write the answers in their notebook.

Class 66: Words, Sentences and Maths

To develop:

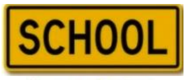

School skills: Handwriting

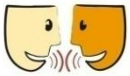

Language and communication: Reading and writing

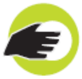

Arm and hand control: Pencil grasp and control

Need:

- Handwriting worksheets
- Writing pencils

Instructions:

1. Tell children today we will practice our handwriting. This will help us with going to school. You all must sit quietly at your desk and write, like you will need to do at school.
2. Give children different handwriting worksheets according to their skill:
    a. Single letter
    b. If single letters are easy for the child, then give them combined letters
    c. If combined letters are easy, give them words.
    d. If words are easy, give them sentences.
3. Sound out the letters and read out words. Give examples of words that the single/combined letters are used in. Use pictures to help children understand.
4. Ask children to present their completed worksheet to the class. Children to say the sound the letter makes, and say a word that contains that letter, or read out their sentences to the class.
5. Count 1 to 40. (Or more if this is easy).
6. Give each child three to ten items e.g. blocks, basket of balls. Ask children counting questions, for example:
    a. How many items do they have in total
    b. How many blue items are there
    c. Pass me five balls, four yellow balls
7. Do simple maths problems as a class – start with addition. Use items to demonstrate.
8. Practice writing numbers using handwriting sheets.
9. Using number flash cards, get the children to put them in order, fill in the missing number and build it backwards.

Class 67: Excursion to Farm

To develop:

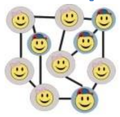

Social behaviour: Different working roles

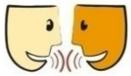

Language and communication: Asking questions, vocabulary

Need:

- Rickshaw transport
- Assistive devices for mobility
- Notebooks
- Writing pencils

Instructions:

1. Tell children that today we are going to visit a farm and see what a farmer does. We want to find out about his working schedule. To do this, we will interview him by asking a few questions.
2. Prepare some interview questions:
    a. What time do you get up in the morning?
    b. What time do you start work?
    c. What do you do at work?
    d. What time do you finish work?
    e. What time do you go to sleep?
3. Ask children to write the interview questions in their notebook. For those children unable to write sentences, write the questions on strips of paper for children to put in order and glue into their notebook.
4. At the farm, observe the surroundings. What do you see? Name different animals, crops, colours etc. Ask children to write down part or all of the words in their books.
5. Interview a farmer. Children take turn asking the questions. Children write the answers in their notebook.

Class 68: Teacher Says

To develop:

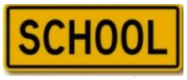

School skills: Following directions

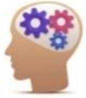

Cognition: Following one and two step commands

Need:

- Sign language and/or communication board

Instructions:

1. Children gather around Teacher.
2. Tell children "I am your teacher. At school you must do what your teacher says. Now we are going to play a game called 'Teacher Says'. We will all take turns being the teacher".
3. Give instructions that a teacher might make at school, e.g.:
   a. Sit down
   b. Stand up
   c. No talking
   d. Sit at your desk and write in your notebooks (sit at table and pretend to write)
   e. Put your hand up
   f. Pay attention
   g. Look at me
   h. Etc.
4. Use sign language, communication boards and other visual cues for those children with hearing impairment.
5. Nominate other children to be the teacher and give the rest of the class directions.

Class 69: Science, Make Lemonade

To develop:

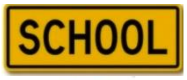

School skills: Science

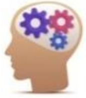

Cognition: Sequencing, cause and effect, measuring

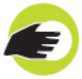

Arm and hand control: Pouring, stirring

Need:

For each child:

- Lemon
- Drinking glass
- Water
- 1 teaspoon baking soda
- Sugar

Instructions:

1. Revision: What is science? Explain science is the study of the world through experiment. The world is composed of animal, water, soil and plants among others all of which are being studied in science.
2. Today we will do an experiment – we will make a fizzy lemon drink! Explain and discuss what lemonade is (7-up and sprite). How do we make lemon juice go bubbly and fizzy?
3. Set-up for the experiment inside or outside.
4. Instruct children to squeeze as much of the juice from the lemon as you can into the glass.
5. Tell children to then pour in an equal amount of water as lemon juice.
6. Children add a teaspoon of baking soda and stir
7. Taste the mixture and add some sugar if it needs to be sweeter.
8. Drink and enjoy!

Class 70: Excursion to Hand Loom

To develop:

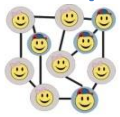

Social behaviour: Different working roles

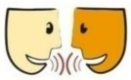

Language and communication: Asking questions, vocabulary

Need:

- Rickshaw transport
- Assistive devices for mobility
- Notebooks
- Writing pencils

Instructions:

1. Tell children that today we are going to visit a farm and see what a person working with a hand loom does. We want to find out about his working schedule. To do this, we will interview him by asking a few questions.
2. Revise the interview questions:
   a. What time do you get up in the morning?
   b. What time do you start work?
   c. What do you do at work?
   d. What time do you finish work?
   e. What time do you go to sleep?
3. Ask children to write the interview questions in their notebook. For those children unable to write sentences, write the questions on strips of paper for children to put in order and glue into their notebook.
4. At the hand loom, observe the surroundings. What do you see? Name different colours, fabrics etc. Ask children to write down part or all of the words in their books.
5. Interview a worker in the handloom factory. Children take turn asking the questions. Children write the answers in their notebook.

Class 71: Words, Sentences and Maths

To develop:

School skills: Handwriting

Language and communication: Reading and writing

Arm and hand control: Pencil grasp and control

Need:

- Handwriting worksheets
- Writing pencils

Instructions:

1. Tell children today we will practice our handwriting. This will help us with going to school. You all must sit quietly at your desk and write, like you will need to do at school.
2. Give children different handwriting worksheets according to their skill:
    a. Single letter
    b. If single letters are easy for the child, then give them combined letters
    c. If combined letters are easy, give them words.
    d. If words are easy, give them sentences.
3. Sound out the letters and read out words. Give examples of words that the single/combined letters are used in. Use pictures to help children understand.
4. Ask children to present their completed worksheet to the class. Children to say the sound the letter makes, and say a word that contains that letter, or read out their sentences to the class.
5. Count 1 to 40. (Or more if this is easy).
6. Give each child three to ten items e.g. blocks, basket of balls. Ask children counting questions. Eg:
    a. How many items do they have in total
    b. How many blue items are there
    c. Pass me five balls, four yellow balls
7. Do simple maths problems as a class – start with addition, and then introduce subtraction. Use items to demonstrate.
8. Practice writing numbers using handwriting sheets.
9. Using number flash cards, get the children to put them in order, fill in the missing number and build it backwards.

Class 72: Helping at Home, Washing Dishes and Wiping

To develop:

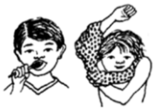

Activities of daily living: Cleaning

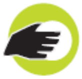

Arm and hand control: Grip strength, coordination

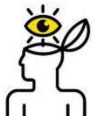

Perception: Determining differences and similarities in object shape, size etc, and understanding spatial relationship between objects

Need:

- Buckets of water
- Dishwashing soap
- Sponges
- Cloths

Instructions:

1. Tell children they can help at home by washing the dishes, and wiping down benches.
2. Instruct children to:
    a. Children able to walk; fill up buckets of water and carry them into the class.
    b. Wash their dishes in buckets of water using a sponge.
    c. Dry the dishes with a cloth.
    d. Stack dishes in a specified area.
    e. Children able to walk; carry the dishes to the kitchen.
    f. Wipe down benches, and floor mat using a damp cloth.
3. Tell children they can be more independent and helpful in the kitchen by practicing opening and closing lids, stirring, shaking, pouring and lifting.
4. Set-up the different stations and instruct children to rotate from one station to another every 2-3 minutes.
    h. Opening and closing jar lids
    i. Opening and closing bottle lids
    j. Stirring a bowl with dry rice
    k. Shaking a bottle with dry rice
    l. Pouring water from a jug into a cup, or if that is too difficult, into a bowl.
    m. Lifting and carrying items of different sizes and shapes from one place to another.

Class 73: Science, make a Volcano

To develop:

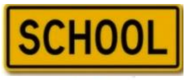

School skills: Science

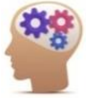

Cognition: Sequencing, cause and effect, measuring

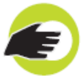

Arm and hand control: Pouring, stirring

Need:

- Baking soda (make sure it's not baking powder)
- Vinegar
- A container to hold everything and avoid a big mess
- Cloth

Instructions:

1. Revision: What is science? Explain science is the study of the world through experiment. The world is composed of animal, water, soil and plants among others all of which are being studied in science.
2. Today we will do an experiment – we will make a volcano! Explain and discuss what a volcano is.
3. Set-up activity outside. This will be messy!
4. Instruct children to place some of the baking soda into your container.
5. Tell children to pour in some of the vinegar.
6. Watch as the reaction takes place!

Class 74: Teacher Says

To develop:

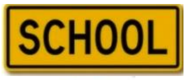

School skills: Following directions

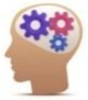

Cognition: Following one and two step commands

Need:

- Sign language, picture cards/symbols or communication boards. Refer to Appendix: Community Resources for details.

Instructions:

1. Children gather around the teacher.
2. Tell children "I am your teacher. At school you must do what your teacher says. Now we are going to play a game called 'Teacher Says'. We will all take turns being the teacher".
3. Give instructions that a teacher might make at school, e.g.:
    a. Sit down
    b. Stand up
    c. No talking
    d. Sit at your desk and write in your notebooks (sit at table and pretend to write)
    e. Put your hand up
    f. Pay attention
    g. Look at me
    h. Etc.
4. Use sign language, communication boards and other visual cues for those children with hearing impairment.
5. Nominate other children to be the teacher and give the rest of the class directions.

Class 75: Excursion to a Tailor

To develop:

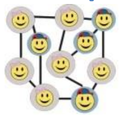

Social behaviour: Different working roles

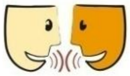

Language and communication: Asking questions, vocabulary

Need:

- Rickshaw transport
- Assistive devices for mobility
- Notebooks
- Writing pencils

Instructions:

1. Tell children that today we are going to visit a farm and see what a tailor does. We want to find out about his working schedule. To do this, we will interview him by asking a few questions.
2. Revise the interview questions:
   a. What time do you get up in the morning?
   b. What time do you start work?
   c. What do you do at work?
   d. What time do you finish work?
   e. What time do you go to sleep?
3. Ask children to write the interview questions in their notebook. For those children unable to write sentences, write the questions on strips of paper for children to put in order and glue into their notebook.
4. At the tailors, observe the surroundings. What do you see? Name different colours, fabrics etc. Ask children to write down part or all of the words in their books.
5. Interview a tailor. Children take turn asking the questions. Children write the answers in their notebook.

Class 76: Invitation to Your Graduation Ceremony

To develop:

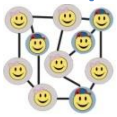

Social behaviour: Engagement in family role

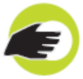

Arm and hand control: Threading, painting, gluing

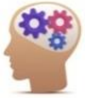

Cognition: Counting

Need:

- Paper
- Coloured pencils
- Craft materials – coloured paper, scrap material, glitter, newspaper/magazines etc.
- Glue
- Envelopes

Instructions:

1. Tell the children that on Friday you will be graduating from Shishu Shorgo. Friday will be your last day of class at Shishu Shorgo. So on Friday we will have a Graduation Ceremony. You need to invite your family to come to your Graduation Ceremony. One way of doing this is making them an invitation card.
2. Revision: show children an example of an invitation, and talk about what is included on the invitation. Make a list of what to include on the board e.g. name of person you are inviting, event, time and place, who the invitation is from etc.
3. Give each child a piece of paper. Ask them to copy headings from the board and fill in the spaces.
4. Children then decorate the invitation.
5. Children to put the invitation into an envelope, address the envelope to their family, and decorate the envelope.

Homework: Children give the invitation to their family.

Class 77: Making Graduation Nasta, Jhal Muri

To develop:

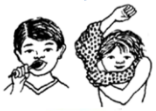

Activities of daily living: Basic food preparation and eating

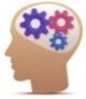

Cognition: Sequencing, construction

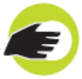

Arm and hand control: Grasp and release, cutting, peeling, scooping, pouring

Need:

- Puffed Rice (Muri)
- Chickpea flour noodles (Sev)
- Fried lentils (Channa Dal)
- Crushed peanuts
- Mung bean sprouts
- Cilantro
- Coriander leaves
- Coconut meat
- Chopped ginger root
- Tomato
- Cucumber
- Chopped onion
- Tamarind sauce
- Lime
- Mustard oil
- Chaat masala
- Garam masala
- Chopping board/plate
- Large bowls
- Knife (ensure child supervised while using the knife)
- Spoon
- Small bowls or newspaper

Instructions:

1. Tell children today you will be making Nasta for your graduation ceremony on Friday.
2. All children wash their hands with soap
3. Write instructions on the board or provide children with visual instruction sheets and explain the instructions to the children:
   a. Ingredients:
      xvi. 1 cup puffed rice (Muri)
      xvii. ¼ cup chickpea flour noodles (Sev)
      xviii. ¼ cup fried lentils (Channa Dal)
      xix. ¼ cup crushed peanuts
      xx. ½ cup mung bean sprouts
      xxi. ¼ cup cilantro
      xxii. ¼ cup coriander leaves
      xxiii. 1 tablespoon coconut meat
      xxiv. 1 tablespoon chopped ginger root
      xxv. 1 tomato
      xxvi. ½ cucumber
      xxvii. ¼ chopped onion
      xxviii. 1 tablespoon tamarind sauce
      xxix. Lime juice of ¼ lime
      xxx. 1 teaspoon mustard oil
   b. Chop cilantro, coriander leaves, coconut, tomato and cucumber and put into a large bowl.**
   c. Measure and add to the bowl: puffed rice, chickpea flour noodles, fried lentils, peanuts, mung bean sprouts, ginger, onions, tamarind sauce, mustard oil, chaat masala and garam masala
   d. Squeeze the juice of ¼ of a lime into the bowl
   e. Mix everything together using a spoon

   ** Child to measure and crush using hands and/or chop using a knife: cilantro, coriander leaves, and coconut. Ensure each child is closely supervised while using a knife. If child manages well using a knife, child can chop tomato and cucumber (with close supervision). Otherwise, adult should chop tomato and cucumber and then the child puts this into the bowl.
4. Children pour into a container to be stored for their Graduation Ceremony on Friday.

Homework: Children to dress-up as what they want to be when they grow up e.g. a nurse, a tailor etc.

Class 78: Dress-Up Day, What do I want to be when I grow up?

To develop:

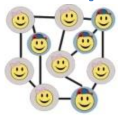

Social behaviour: Explore different working roles

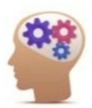

Cognition: Recall, setting goals

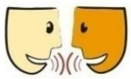

Language and communication: Vocabulary

Need:

- My Story books
- Writing pencils
- Colour pencils
- Magazines/Newspapers
- Glue
- Scissors

Instructions:

1. Children to introduce themselves and what they have dressed up as.
2. Discuss as a class what each job is and what you would do.
3. Role play scenarios: e.g. if one child is dressed up as a nurse, have another child pretend to be their patient.
4. Take a photo of each child and print it out to give to them on Graduation Day.
5. Instruct children to review their My Story books.
6. Write the headings on the board:
    a. At school I would like to learn about:
    b. At home I will help others by:
    c. What I would like to be when I grow up:
7. Ask children to copy the headings into the My Story book.
8. Children to then write, draw, or create cut and paste pictures from magazines/newspapers to answer each heading.

Class 79: Making a Graduation Hat

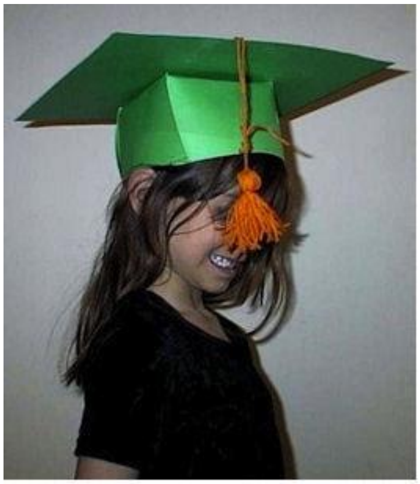

To develop:

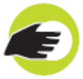

Arm and hand control: Cutting, gluing, folding, stapling

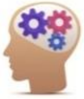

Cognition: Sequencing, construction

Need:

- Cardboard paper
- Stapler
- Glue
- Ribbon
- Scissors

Instructions:

1. Cut 2 squares out of the poster board – make them about 12-inches across.
2. Cut large 'V' shapes in all 4 corners of the cardboard paper as shown in the diagram.
3. Fold in the edge of each 'V' and staple them together approximately where the red dots are in the diagram.
4. Match the numbers of each side together; for example, staple 1 and 1 together, staple 2 and 2 together etc. This will be the part of the cap that will go on a head.
5. Put a dab of glue in the centre of the square piece of cardboard paper that you didn't cut. Glue the top of the hat to this board.
6. Make a tassel out of ribbon and glue it to the centre top of the cardboard paper.
7. Store it in the classroom for the children to wear at their Graduation Ceremony on Friday.

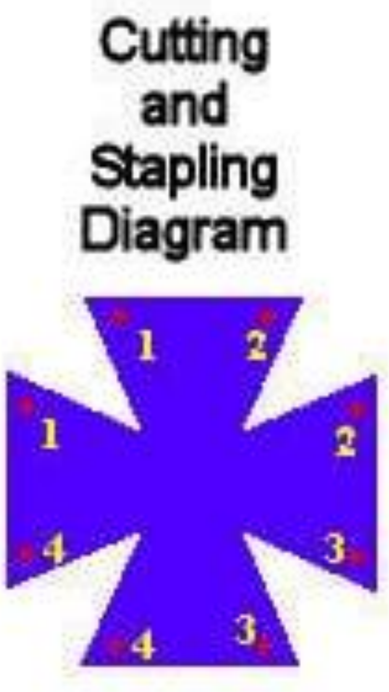

**Teacher's Homework:** Prepare a speech and graduation certificates for tomorrow's class.

Class 80: Graduation Ceremony

To develop:

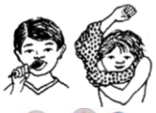

Activities of daily living: Serving food

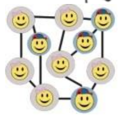

Social behaviour: Greetings and polite interactions

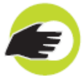

Arm and hand control: Scooping, pouring, folding, grasp and release, lifting and carrying

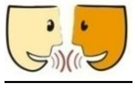

Language and communication: Verbal and visual communication

Need:

- Nasta (Jhal Muri prepared during Class 77)
- Plates/newspaper
- Glasses
- Water
- Serving tray
- Serving trolley

Instructions:

1. Children to introduce members of their family to the rest of the class – their name, who they are in their family, what they do together, what they like etc.
2. Children to greet others' family members, introduce self, and ask 'how are you today'.
3. Teacher to make a speech, thanking the children and families for participating, a summary of what was achieved over the past 4 month program.
4. Teacher to present Graduation Certificates and individual awards to every child in the class.
5. When the child's name is called, the child must move to the teacher and take their certificate. The other children clap.
6. Children to ask families 'would you like some nasta?' and 'would you like some water?'
7. Children to wash their hands with soap.
8. Children to serve nasta by putting food on individual plates or in wrapped up newspaper, and bringing glasses of water for the families etc.

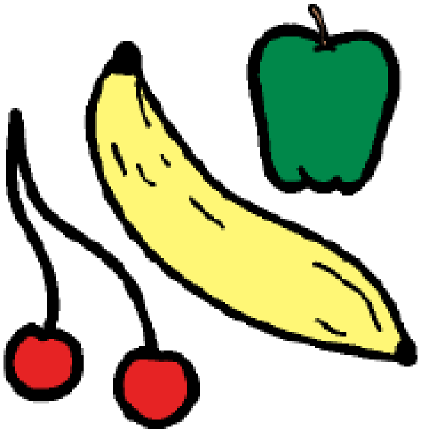

নাস্তা

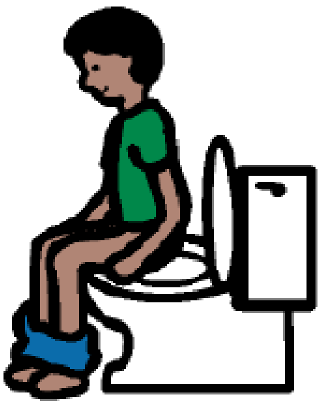

টয়লেট

Nasta & Toilet Break

Helps develop these Functional Skills:

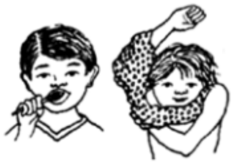

Activities of Daily Living

The nasta and toilet break is a fantastic opportunity for children to practice functional skills, particularly self-care skills. Remember, the main reason why we provide therapy for children with disabilities is to help them be more independent in functional tasks. So, even though this is considered a break for the children, it is actually one of the most important sessions of the day!

Seek any opportunities for children to practice functional skills throughout the day, but particularly use this break to focus on developing independence in functional skills, specifically, Activities of Daily Living. Functional skills you can get the children to practice during this break include:

1. Cleaning-up the classroom
2. Putting shoes on and taking shoes off
3. Washing hands
4. Eating nasta
5. Toileting
6. Dressing

Acknowledgement:

This section includes ideas from Chapter 36: Feeding, Chapter 37: Dressing, and Chapter 38: Toilet Training, of the book Disabled Village Children by David Werner:
Werner, D. (2009). Disabled Village Children: A guide for community health workers, teachers, and families. California: The Hesperian Foundation.
Please refer to the English and/or Bangla version of Disabled Village Children for more details.

Activities of Daily Living: Cleaning-up the classroom

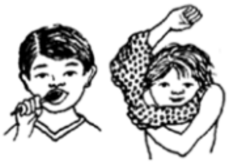

Involves these skills:

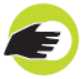

Arm and hand control: Gasp and release, lifting, carrying, coordination

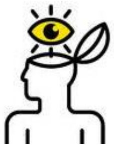

Perception: Locating objects in a room

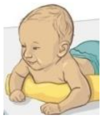

Head control and sitting while picking up objects

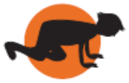 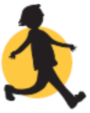

Crawling, walking towards objects

Need:

- Baskets to put toys in
- Plastic rubbish bins
- Shelves to put toys in

Instructions:

For children who have significant difficulty moving:

1. Tell the child to sit. Support the child with cushions/special chair if necessary.
2. Give the child a basket to put toys in and ask the child to pick up toys around them and put them in the basket.
3. Give the child a damp cloth. And ask the child to wipe down the area around them with the cloth.

For children who can move, tell children to:

1. Pick up items around the room and put them away – in baskets, on shelves, and put rubbish in the bin. For children unable to walk independently, get them to push a cart filled with toys to then put away on a shelf. Get children to name and count how many items they are carrying, how many are left to put away etc.
2. Carry buckets of water to clean up with or to water plants/trees.
3. Sweep the floor with a broom.

Activities of Daily Living: Putting Shoes on & Taking Shoes off

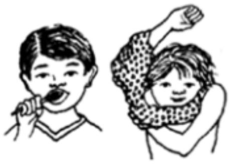

Involves these skills:

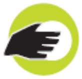

Arm and hand control: Reaching, fine motor to do up and undo shoelaces, buckles or velcro

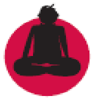

Sitting

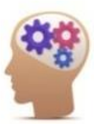

Cognition: Problem solving

Need:

- No equipment

Instructions:

1. Tell children to put their shoes on before going outside, and to take their shoes off before coming back inside. Get children to count how many feet they have and how many shoes they wear.
2. Children to complete as many of the steps of the task as independently as possible:
    a. Select own shoes
    b. Pick up shoes with hands
    c. Sit on the ground
    d. Choose left shoe for left foot
    e. Open fastening (loosen shoe laces or open Velcro)
    f. Put shoe in front of foot
    g. Push toes into shoe, while holding onto shoe
    h. Push heel into shoe, while holding onto shoe
    i. Close fastening (shoe laces or Velcro)
3. Use these strategies:

The key points to helping a child gain independence in activities of daily living are:

- Be patient, give the child TIME to think through and attempt/complete steps of the task themself.
- Recognise and acknowledge a child's attempts and ability to complete steps of the task independently.
- Do NOT take over and do the task for them. If the task is too difficult for them, think why is it too difficult? FACILITATE ability.
- Provide prompts only if the child is not initiating or skips a step of the task, i.e. only provide a prompt if it is needed, and do not continually prompt the child through a task. Provide a prompt and then give the child time to think and do.
- Only provide assistance to facilitate ability, and make the step of the task achievable for the child. Make sure that you don't provide so much assistance that the child is not actively participating. Ensure that the step of the task is still a challenge, but provide assistance so that it is achievable for the child. The child needs to do AS MUCH as they can themselves, and ALWAYS actively participate.

Activities of Daily Living: Washing Hands

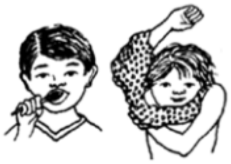

Involves these skills:

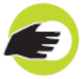

Arm and hand control: Reaching, turning tap

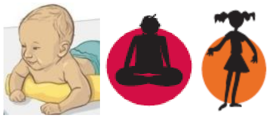

Head control and sitting or standing while washing hands

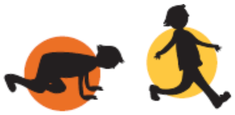

Crawling, walking towards objects

Need:

- Bucket
- Washcloth x 2
- Towel x 2
- Hand soap x 2

Instructions:

1. Tell children to put their shoes on.
2. Have a bucket with water and plastic chair set-up to use for those children who cannot reach the sink or stand independently. Have a wash cloth in the bucket and beside the sink, and hand soap and dry towel beside the sink and bucket.
3. One by one, ask the children to wash their hands. Get the children to count to 5 or 10 as they wash their hands. Enable the child to do as much of the task as they can themselves, this may be just reaching to touch the water with their hands.
    a. For children able to stand and reach the sink:
    Get them to stand in front of the sink, turn on the tap, put soap on their hands, wash their hands, turn off the tap and dry their hands.
    b. For children unable to reach the sink, but able to stand:
    Get them to stand beside the bucket, turn on the tap to put water in the bucket, turn off the tap, put soap on their hands, wash their hands and then dry their hands.
    c. For children unable to stand:
    Get them to sit on a plastic chair beside the bucket, turn on the tap to put water in the bucket, turn off the tap, put soap on their hands, wash their hands and then dry their hands.
4. Once finished washing their face and hands, instruct the child to return to the classroom, take off their shoes, and wait for nasta.

Activities of Daily Living: Eating Nasta

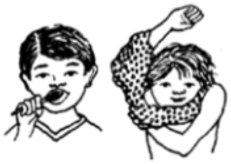

Involves these skills:

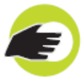

Arm and hand control: Hand to mouth coordination, grasp and release

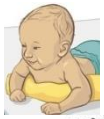

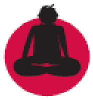

Head control and sitting while eating

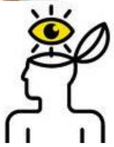

Perception: Position in space

Need:

- Nasta. Provide a variety of textured food from day to day.
- Supportive seating/cushions, as required.
- Assistive devices for eating and drinking, as required.

Instructions:

1. Tell children it is time to eat our morning-tea.
2. Tell children to sit on the floor or at the table. Use supportive seats/cushions for children who require support to sit.
3. Get the children to count how many people are eating, and how many plates/spoons/napkins they need. Select children to collect the right number of plates/spoons/napkins and get all children to count as they are handed out.
4. Encourage the child to do as many steps of the task as independently as possible.

Steps of Eating Food:

a. Reach and bring hand to plate (using spoon/fork if required)
b. Break up food into smaller pieces with their hand (if required)
c. Grasp piece of food with their hand or using a spoon/fork, and bring it to their mouth
d. Open mouth
e. Put food inside mouth (release grasp)
f. Close mouth
g. Chew and swallow
h. Bring hand back to plate
i. Repeat until all food finished

Assistive devices for eating:

If requiring spoon/fork to eat food, but child has difficulty maintaining grasp on handle:

a) Make the handle thicker

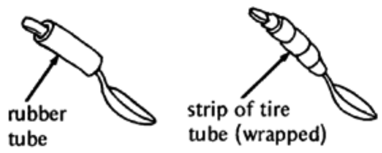

b) Add a strap to go around the hand

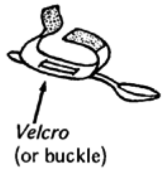

If the child has difficulty raising their arm to bring their hand to their mouth:

a) Use an arm rocker

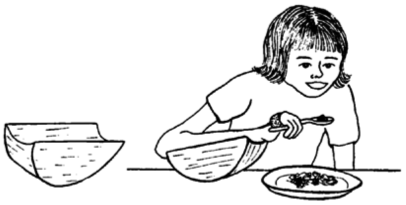

Steps of Drinking from a Cup:

a. Reach out for cup
b. Grasp cup
c. Lift cup and bring to mouth
d. Open mouth, tilt head back, tilt cup so that some water enters mouth
e. Close mouth, drop chin/ bring head back to straight position and swallow
f. Bring cup back to ground/table
g. Repeat

Assistive devices for drinking:

If tilting head back causes uncontrolled backward stiffening or coughing (which indicates possible choking):

a) Use a plastic cup with a piece cut out, so that the child can drink without bending their head back.

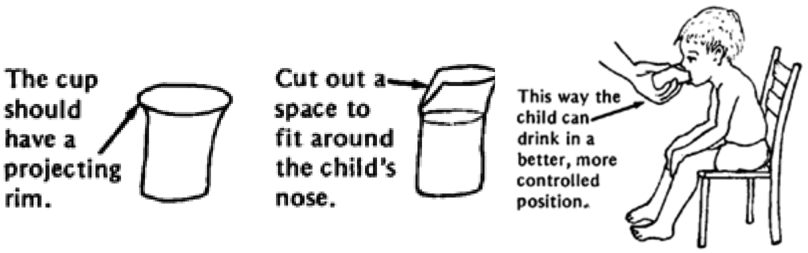

b) For children who cannot hold a cup, use a straw and a non-spill cup. You can make a non-spill cup by gluing a plate or tin to the bottom of the cup.

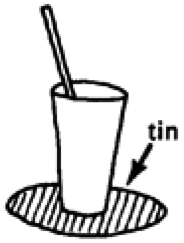

Activities of Daily Living: Toileting

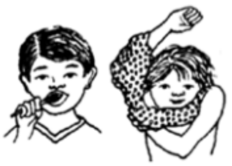

Requires these skills:

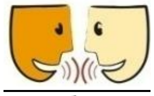
Language and communication: Communicating need to use the toilet

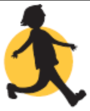
Walking to and from the toilet

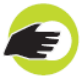
Arm and hand control: Pulling up and down pants, adjusting clothing, opening and closing fastenings, washing hands

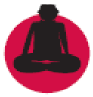
Squatting or sitting while toileting

Need:

- Communication board
- Sign language sheet
- Mobility devices as required
- Toileting devices as required

Instructions:

1. Ensure that there is a way for every child to easily signal the need to use the toilet. It could be verbally, using a communication board, and having a toilet card up on a wall so that any child can look/point/ take the card easily to show the teacher, even during an activity.

2. If the child is not able to sense the need to toilet, put the child on a toilet schedule. This could look like:

Atik's toilet Schedule

<table>
<tr><td rowspan="5">I need to sit on the toilet for 5 minutes:</td><td>When I wake up in the morning</td></tr>
<tr><td>Twenty minutes after I finish morning tea</td></tr>
<tr><td>Twenty minutes after I finish lunch</td></tr>
<tr><td>Twenty minutes after I finish dinner</td></tr>
<tr><td>Before I go to sleep</td></tr>
</table>

3. Encourage each child to do as many steps of the task as independently as possible.

Steps of toileting:

a) Identifying need.
b) Signalling need.
c) Moving to the toilet.
d) Backing onto the toilet
e) Pulling pants down
f) Squatting/sitting
g) Bladder/bowel movement
h) Cleaning self
i) Standing
j) Pulling pants up
k) Moving from toilet to sink
l) Washing hands

Adaptive strategies

If child has difficulty communicating need to use the toilet verbally:

a) Teach the child sign and/or use a communication board to support verbal communication.

If the child is unable to walk to the toilet independently:

a) And is learning to walk, provide support at the child's hips (refer to individual movement activities, walking), and/or use walking frame.
b) And has contractures/deformity such that they cannot stand/walk, use a wheelchair.

To teach the child how to pull their pants up and down:

If the child is not able to use their fingers to pull pants on and off, use pants with an elastic waistband. Teach the child to use the heel of their palm to push their pants down. Attach loops to the waistband so that the child can slide their hands into them and raise their arms to pull up their pants.

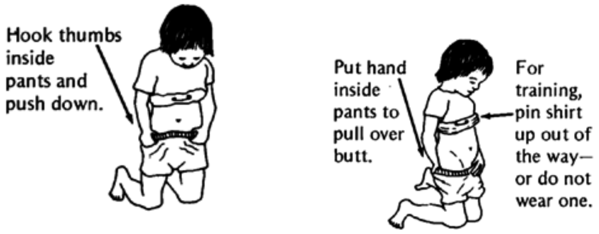

If the child is unable to maintain standing or kneeling position to pull pants up and down, they can do this while lying on a mat:

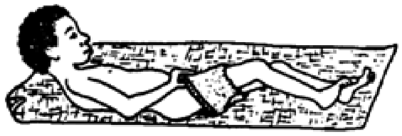

If the child has difficulty maintaining balance in the squat position: Provide the child with hand support:

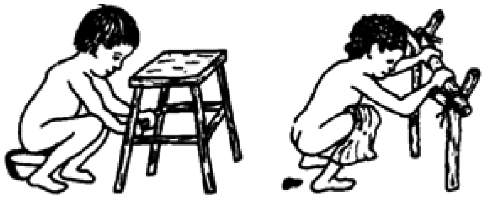

If the child is unable to squat:

a) Use a tire potty seat. Put a tire over the squat toilet. The child can sit on this tyre while toileting

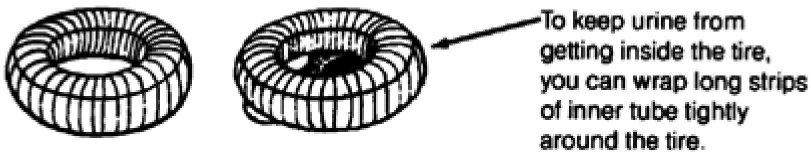

b) Put a tire on a wood or metal framed chair

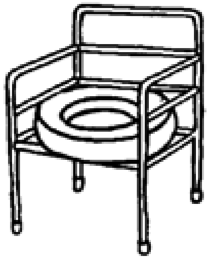

c) Make a wooden or metal-framed chair with circular hole in the seat.

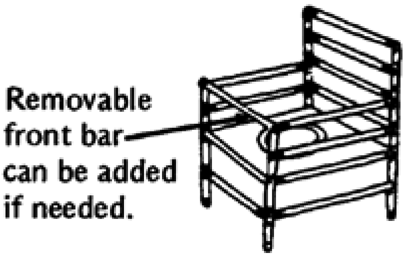

Activities of Daily Living: Dressing

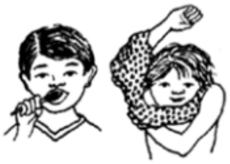

Requires these skills:

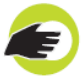

Arm and hand control: Coordination and fine motor to do up fastenings

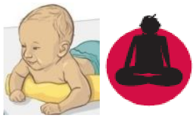

Head control and sitting while dressing

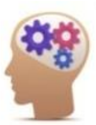

Cognition: Correct orientation of clothing, problem solving

Need:

- No equipment

Instructions:

1. Make the most of opportunities to practice dressing with the children e.g. if a child is wanting to take off/put on a jumper, and when they need to pull up/down their pants to go to the toilet.
2. Get the child to do as many steps of the task as they can for themself. Steps of the task include:
    a. Choose item of clothing
    b. Position item of clothing in front of them ready to put on
    c. Thread limbs through clothing (i.e. legs through leg holes, or arms through arm holes)
        i. If jumper, kameez or t-shirt: Pull jumper over head
        ii. If pants: Pull pants up/down legs
        iii. Pull up/down pants over bottom or jumper/t-shirt/kameez over torso
        iv. Do up fastenings e.g. zip and buttons
    d. Adjust clothing so that it sits comfortably
3. Only provide prompts and assistance if needed, and only as much as needed for the child to complete the task.
4. Use these strategies:

- Be patient! Give the child time to problem solve through the task of dressing. Praise the child when he/she does well or tries hard.
- Always let the child finish the task. Little by little have the child do more of the steps.
- Use loose-fitting, easy to put on clothing.
- If one arm or leg is more affected than the other, tell the child to thread the affected limb through the pants/arm-sleeve first.
- Let the child try to do as much as they can themself. Be ready to help if it gets too difficult, but only as much as is needed.
- When providing assistance, talk to the child. Help the child learn body parts, the names of clothes and the way these relate. E.g. “The arm goes into the sleeve”, “the foot goes into the pants”. Encourage the child to help in any way possible.

Helps to develop these Physical Skills:

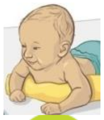 Head and trunk control

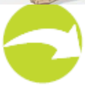 Rolling

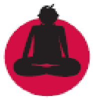 Sitting

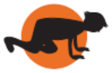 Crawling

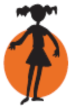 Standing

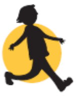 Walking

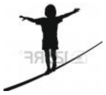 Balance

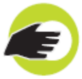 Arm and hand control

Also helps develop these Mental Skills:

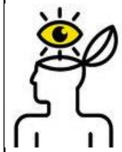 Perception

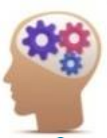 Cognition

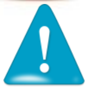 Attention

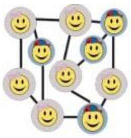 Social behaviour

Movement

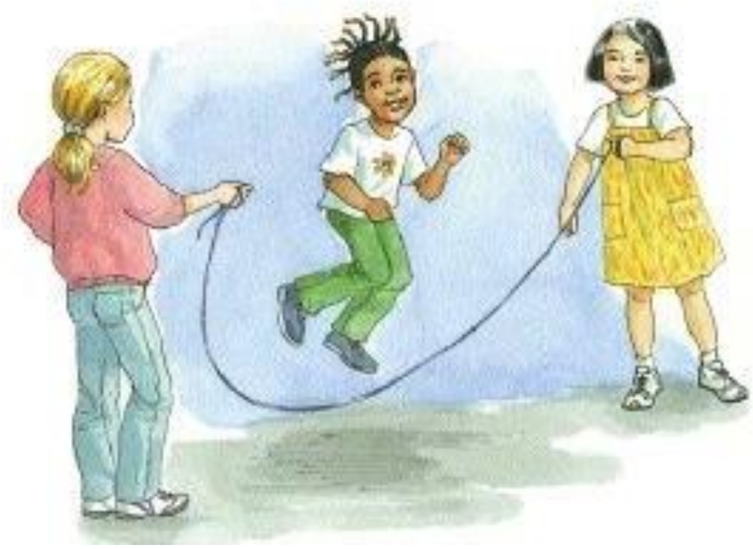

Activities:

1. Stretching: Yoga
2. Individual movement
3. Group movement

Stretching: Yoga

Goal:

- Maintain and increase joint range of motion
- Prevent joint stiffness and muscle shortening
- Warm up body for the group movement activities

Need:

- Foam floor mats
- **Animal Yoga Instruction sheet** (Basic Yoga for all Children)
- Additional Yoga Instruction sheets: **Additional Yoga Poses, Yoga Poses that Help Balance – Moderate and Advanced, Yoga Routine**

Instructions:

1. Follow the Animal Yoga Instruction Sheet and Additional Yoga Poses – Easy
2. Demonstrate the pose, one at a time, and instruct the children to copy you.
3. Once demonstrated, assist those children having difficulty obtaining the pose. Gently guide the child's limbs to achieve a pose as close to the one demonstrated as possible.
4. In all of these activities encourage the children to stay within their "own space", this helps teach the concept of personal space.

To make harder:

- Moderate: Include Additional Yoga Poses - Moderate, Yoga poses that help balance - Moderate
- Advanced: Include Yoga poses that help balance - Advanced, or Yoga Routine, or Yoga Song Sheet

To make easier:

- Follow Animal Yoga and Additional Yoga Poses – Easy.

1. Place your hands around the child's waist, providing light support to enable the child to maintain their balance.
2. Slowly and gently guide the child's limbs to achieve a pose as close to the one demonstrated as possible.

Animal Yoga Instruction Sheet

(Basic Yoga for All Children)

Starting Position

1. **Tell the children to stand or sit in a circle**

Snake

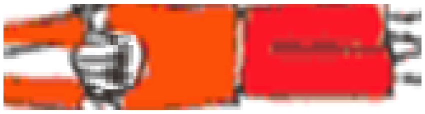

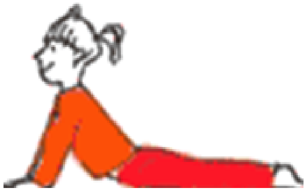

1. Drop to the floor and lie on your stomach
2. Stretch from head to toe, telling the children, "Pretend you are a snake in the grass and stretch in the sun."
3. Hold this pose for approximately 30 seconds
4. Bring hands back by your shoulders, palms on the floor
5. Straighten your arms and lift your head and torso up as high as you can, keeping your elbows close to your body and leaning on your hands.

Crocodile

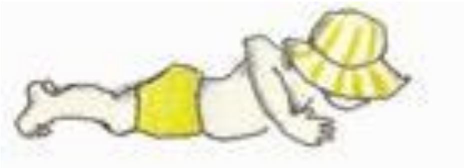

1. Move hands back beside your shoulders with your elbows bent
2. Raise your head and bend upward from the waist, arching your head back.
3. Tell the children, "We're crocodiles in the swamp or the river. What can we find to eat today? Look, get that frog or bug" and snap your jaws open and shut like a crocodile.

Cat

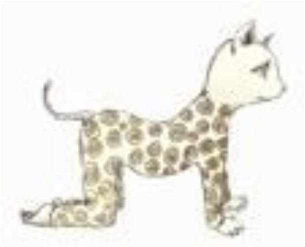

1. Raise your body so that you are kneeling on all fours, keeping arms straight, with relaxed shoulders and raised head
2. Tell the children, "Now let's be a cat". Encourage the children to make "meow" sounds.
3. Arch your back like a cat that is mad.

4. Alternate between the raised head meowing position to the arched back silent position several times, breathing deeply when their back is arched.

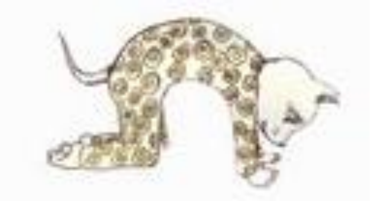

Dog

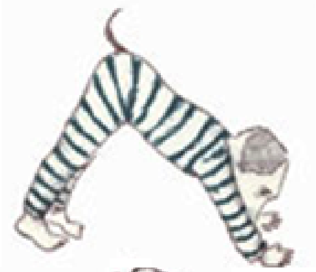

1. Lift up your hips, keeping arms straight. Try and keep your legs straight and feet flat on the floor. This may be a harder pose for them to hold for very long to begin with.
2. Bring your hips back down to the floor, keeping your legs and arms straight, and raising your head like a dog.
3. Encourage the children to "Woof".
4. Vary between hips up and hips down position.
5. Finish with hips up.

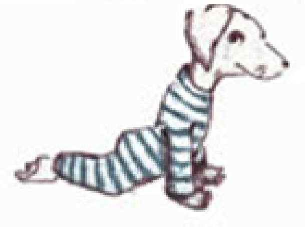

Lion

1. Lean back on your knees and lift arms completely off the floor, hanging down beside your body as a lion.
2. Lift your chest, open your mouth wide and from the abdomen, roar like a lion, breathing in and out and stretching their tongue out as they roar. This also can be rather difficult, but the tongue being stretched out relaxes the neck and facial muscles, so encourage them to be silly with "sticking their tongues" out.

Butterfly

1. Sit on your bottom, bend your knees in front of you to make in a triangle shape with the soles of your feet touching each other and hands on your ankles.
2. Move your legs up and down, in a "flapping butterfly" motion. Encourage the children to float and flap slowly and deliberately, being silent like a butterfly.

Monkey

1. Stand on your feet, bend your knees and squat down close to the floor with your feet body width apart.
2. Touch your hands to your chest and then raise and lower your elbows while making a monkey sound “oo oo oo ah ah”

Frog

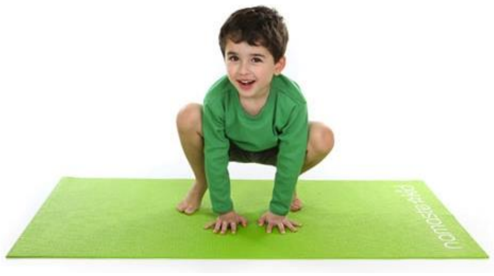

1. Squat on the floor, balancing on your toes, knees spread wide, hands on the floor between your legs.
2. Straighten your legs and lower your head toward your knees
3. Return to squatting
4. Make “ri-bit” noise of a frog
5. Repeat

Turtle

As we begin to finish up our animal yoga session, turtle is a good way to begin to quiet down.

1. Kneel and sit on your heels
2. Lower your upper bodies so that your forehead touches the floor.
3. Slide your arms forward above your head while keeping your bottom curled up on your heels in a small hunched turtle pose.
4. Tell the children to hold inside their shell so no one can see them.

Mouse

We finish our kid yoga game with mouse for quietness and stillness.

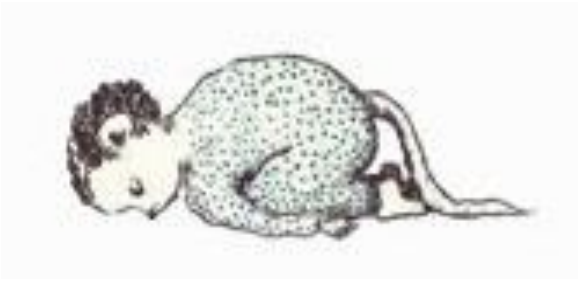

1. Slide your arms back alongside your body toward your feet.
2. Keep your bottoms on your heels and place your arms by your sides so that your hands are along your entire body ending at the feet.
3. Encourage the children to remain "quiet as a mouse" for as long as they can, usually at least 30 seconds.

Additional Yoga Poses Instruction Sheet

Collated from website: http://www.namastekid.com/learn/kids-yoga-poses/

Easy – for all children

Salutation Seal

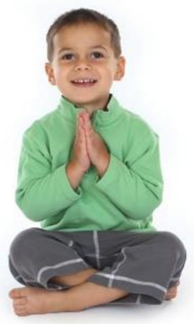

1. Sit with your legs crossed
2. Bring your palms together. Gently press hands against each other, straighten your back and allow your eyes to close

Bridge

1. Lay down on your back
2. Place your arms on the floor, alongside your body
3. Bend your knees, setting your feet flat on the floor
4. Exhale, and lift your hips towards the ceiling. Hold this pose for a few breaths
5. Exhale, and lower your hips back to the floor

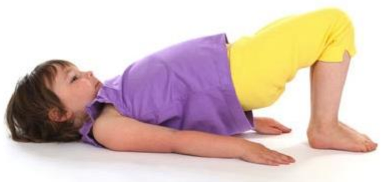

Moderate – for children that can stand with minimal support or independently

Star

1. Stand with your feet wide apart
2. Stretch your arms out to either side
3. Sing "Twinkle Twinkle Little Star"

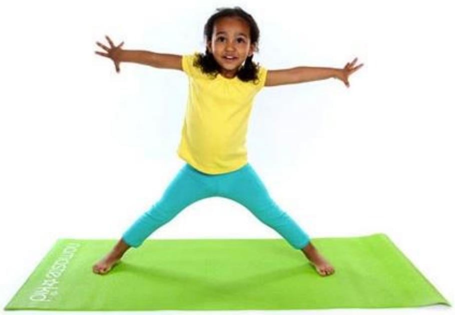

Warrior

1. Stand with your feet wide apart
2. Turn your left foot in and your right foot out 90 degrees
3. Lift your arms to shoulder height
4. Bend your right knee. Be careful not to flex your knee past the 90 degree point with your ankle
5. Keep your torso tall, turn your head, and look out over your right finger tips
6. Warrior call "huh" or "I am strong"
7. Straighten your legs and lower your arms
8. Repeat on opposite side

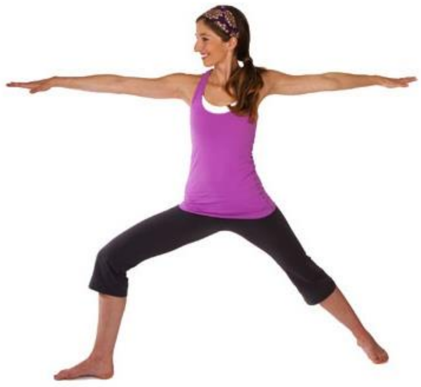

Yoga Poses that help Balance
Moderate – for children who can sit independently

Rainbow

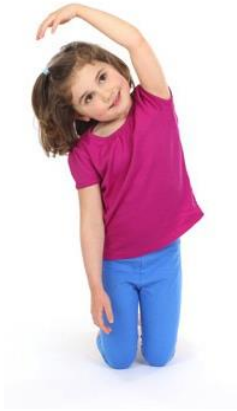

1. Kneeling (can also do standing if the child can stand independently)
2. Raise both hands over your head
3. Drop one hand by your side, exhale, and arch the other over your body
4. Recite the colours of the rainbow
5. As you bring your dropped arm back above your head, straighten your body
6. Repeat on the other side

Three-legged Dog

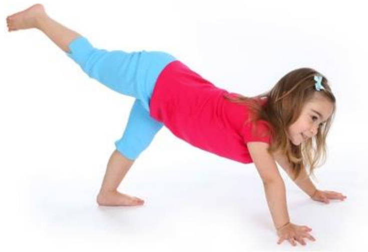

1. Begin as dog with hips up and arms and legs straight
2. Lift one leg
3. Shake leg in the air, like a dog
4. Lower leg
5. Repeat with the opposite leg

Flower

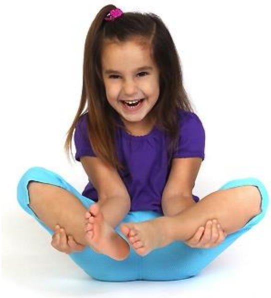

1. Sit on the floor with your back straight and the bottoms of your feet together
2. Place your hands underneath your ankles
3. Lift your feet off the floor, keeping your knees wide and back straight

Partner Boat

1. Sit facing your partner and bend your knees, with feet flat on the floor
2. Touch your big toes to your partner's big toes
3. Lean forward and hold hands with your partner, with arms on the outside of your legs
4. Press the soles of your feet to your partner's on one side. Do the same on the other side.
5. Gently rock back and forth and sing "Row Your Boat".
6. If both partners are balanced and would like a challenge, press your feet together and lift them towards the ceiling to straighten your legs.
7. Slowly return your feet to the floor and stop holding hands to release the pose

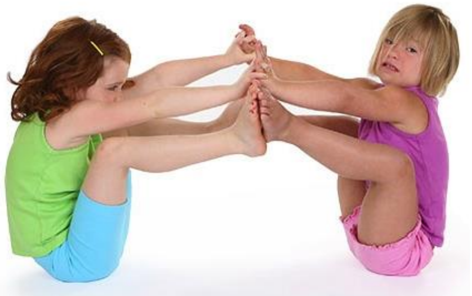

Yoga Poses that help Balance
Advanced – for children who can walk

Tree

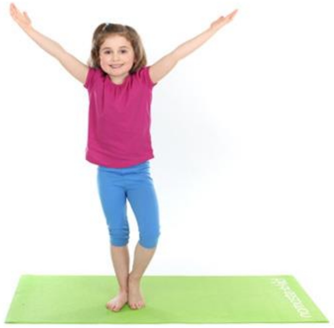

1. Begin standing up straight
2. To help with balance, extend arms out to either side
3. Lift your right foot, turning your knee out, place your foot above or below your left knee, depending on your comfort level
4. Press your hands together in front of you
5. Raise arms overhead, and look up to your hands
6. Return hands to your chest and lower your right leg
7. Repeat with left leg

Airplane

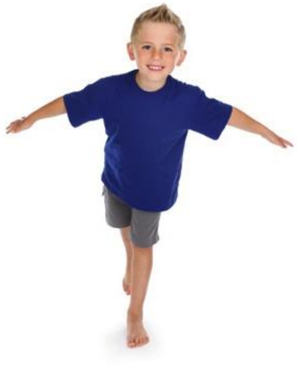

1. Begin standing up straight
2. Extend arms out to either side
3. When you feel balanced, lean forward, lifting one leg straight behind you
4. Hold this pose, make airplane noises and pretend you are flying
5. Return your leg to the ground and your arms to your sides
6. Repeat with opposite leg

Kite

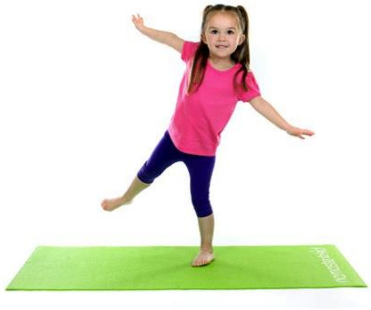

1. Stand tall, with your legs spread wide
2. Reach your arms out to either side
3. Keeping your neck and spine straight, tip your star to one side, balancing on one leg for as long as you can
4. Return to star pose, with both feet on the floor
5. Tip your star to the other side, and hold it with your leg firmly on the floor

Yoga Routine
Advanced – for children who can walk

Start Here

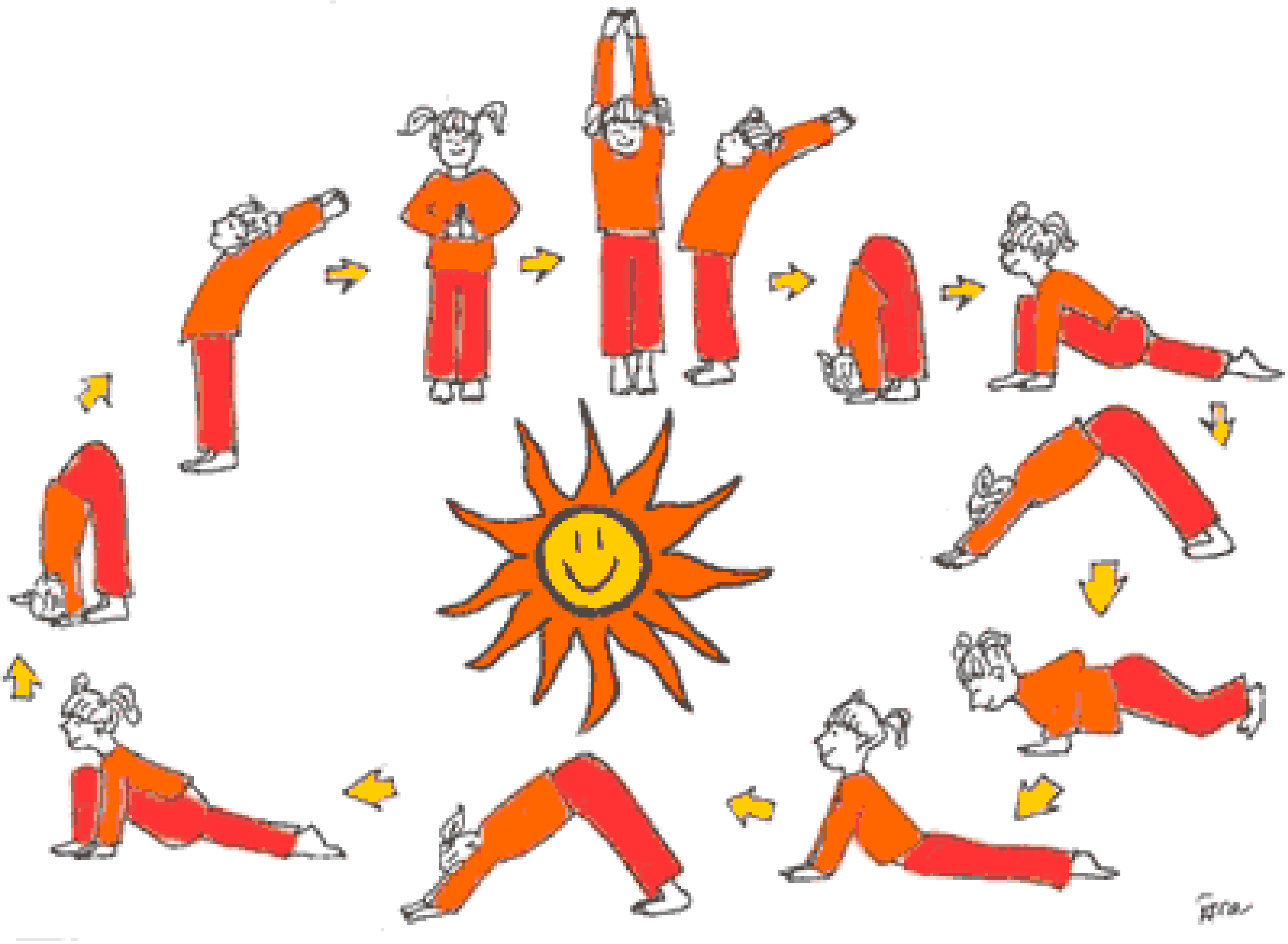

Individual Movement

Goal:

- Work towards the child's individual movement goals

Need:

- Foam floor mats
- Movement skill instruction sheets

Instructions:

1. Select one movement skill for each child to practice for that session:
    a. **Head Control**
    DO THIS IF: The child often has their head down during activities that involve looking straight ahead/around, and raises their head for short periods of time.
    DO NOT DO THIS IF: The child is able to raise their head and look straight and around for as long as needed.
    b. **Rolling and Twisting**
    Suitable for all children.
    c. **Body Control, Balance and Sitting**
    Suitable for all children.
    d. **Creeping and Crawling**
    Suitable for all children.
    e. **Standing, Walking and Balance**
    DO THIS IF: The child can stand with support at their hips, or independently.
    DO NOT DO THIS IF: The child has significant contractures/deformity where the child cannot weight bear through their lower limbs and is not able to stand.

2. Help the child's parent do the activity with their child.

Acknowledgement:

This section is an abbreviated version of Chapter 35, Development Activities of the book Disabled Village Children by David Werner:
Werner, D. (2009). Disabled Village Children: A guide for community health workers, teachers, and families. California: The Hesperian Foundation.
Please refer to the English and/or Bangla version of Disabled Village Children for more details.

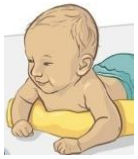

Movement Skill: Head Control

NOT SUITABLE FOR ALL CHILDREN

DO THIS IF:
- The child frequently has their head down during activities that involve looking straight ahead/around, and raises their head for short periods of time.

Goal:
- Child to be able to lift and control her head (and use her eyes and ears).

Need:
- Floor mat
- If lying: pillow, foam, or blanket to use as a soft wedge
- If sitting: supported chair, and/or pillows
- If standing: standing frame
- Something to attract the child's attention (change every day) e.g. brightly coloured objects that make strange or pretty sounds, bubbles, balloon, mirror, lights in cardboard box, hairbrush, puzzle

Instructions:
1. Help position the child in lying, sitting or standing:
   a. In Lying: Lie the child face down on a floor mat.

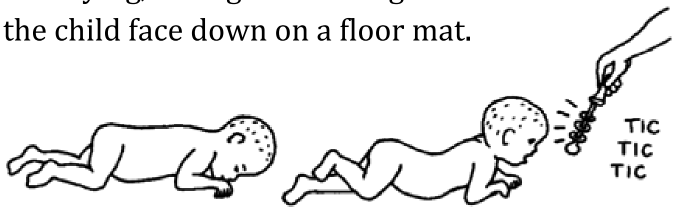

   Or, face down on a floor mat with a soft wedge underneath the child's belly.

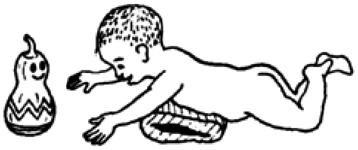

   b. In Sitting: Position the child in a supported chair, or on a foam floor mat cross-legged with pillows to help support the child in sitting.

   c. In Standing: Position the child in a standing frame.

2. Encourage the child to look at something in-line or above eye level to reach for and play with. Attract their attention with:
   a. Brightly coloured objects that make strange or pretty sounds.
   b. Blow bubbles
   c. Balloon above head
   d. Mirror
   e. Lights in cardboard box
   f. Peek-a-boo
   g. Put on items on head
   h. Call name
   i. Lifting head to look in the mirror while parent brushes hair
   j. Puzzle

3. Encourage the child to look up for the toys and maintain this for as long as possible.
4. Measure and record the length of time the child can lift their head for each day to record progress.

Movement Skill: Rolling and Twisting

SUITABLE FOR ALL CHILDREN

Goal:

- Child to be able to roll and twist both sides (left and right) while using their hands

Need:

- Foam floor mats
- Object to hold in the child's hand and pass e.g. ball
- Bucket

Instructions:

1. Lie the child face down on a floor mat.
2. Get child to perch on elbows to lift head.
3. Attract the child's attention by holding a toy/object in front of them.
4. Then move the toy to one side. Encourage the child to turn their head and shoulders to follow the toy, and instruct the child to reach out sideways and take the toy from you, touch it to their chest, and then pass it back to you. Repeat this on the other side.
5. Then move the toy upward, so that the child twists onto their side and back.
6. Play a game where the child rolls and twists to take the toy from you, and then rolls and twists to the other side to pass it to someone else/put in a bucket.

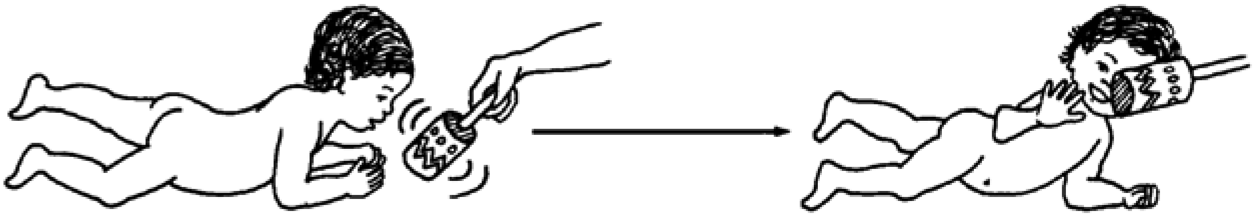

If the child does not roll over after various tries, help them by lifting their leg.

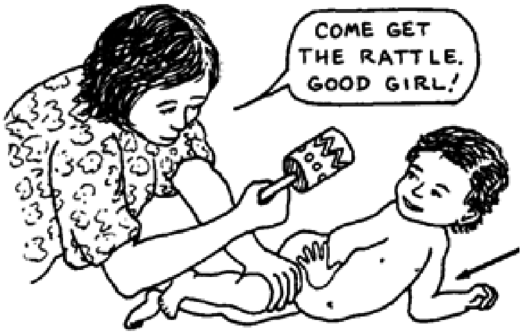

7. Repeat the same above lying the child on their back (face up)

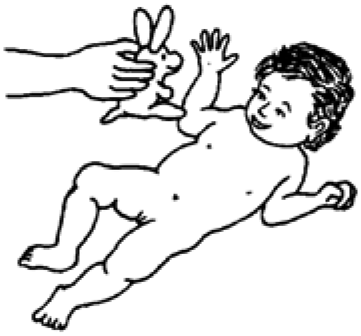

Movement Skill: Body control, balance and sitting

SUITABLE FOR ALL CHILDREN

Goal:

- Child to be able to sit up straight and balance with their body to turn and reach.

Need:

- Floor mat
- Log
- Exercise ball and/or tilt board
- Toy to pass e.g. ball

Instructions:

For children who fall over when you sit them up:

1. Help the child develop a protective reaction with their arms. Put them on a log, hold their hips and slowly roll them sideways. Encourage the child to 'catch' themself with a hand.

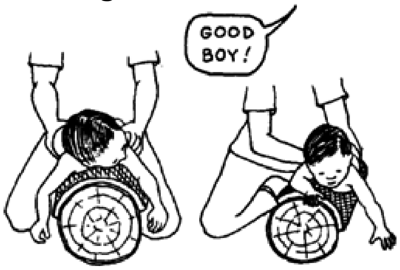

For children who can 'catch' themself lying, but has difficulty sitting independently for short periods of time:

1. Sit her up, hold her above the hips and gently push her from side to side, and forward and backward so that she learns to catch and support herself with her arms.

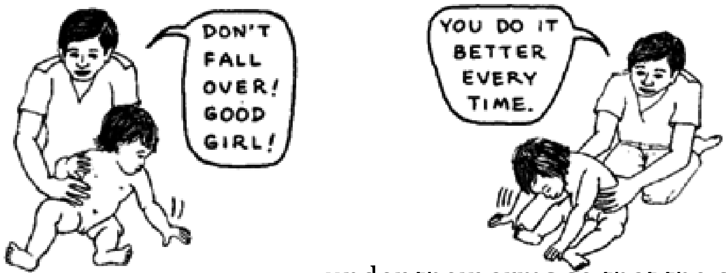

2. Sit the child on a log. Hold the child loosely under their arms so that the child's body can adapt to leaning. As the child gets better balance, move your hands down to his hips and then thighs, so that he depends less on your support. Give the child something to hold so that he learns to use his body and not his arms to keep his balance. You can do the same thing on a 'tilt board' or a large ball.

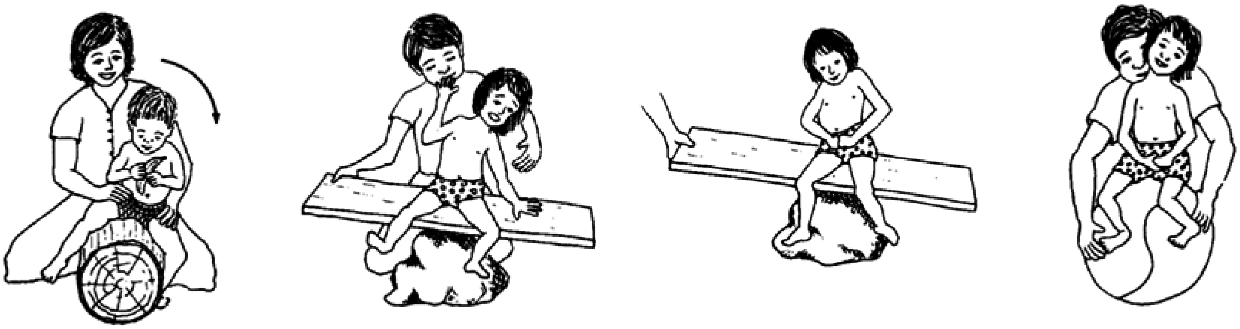

For children able to sit independently for longer periods of time:

1. Sit the child on the 'tilt board' or a large ball and provide no support (just supervision). Ask them to shift their weight from side to side and maintain their balance.
2. Help the child learn to keep their balance while using their hands and twisting their body:
    a. Sitting on the ground: Pass a ball or other toy from side to side.

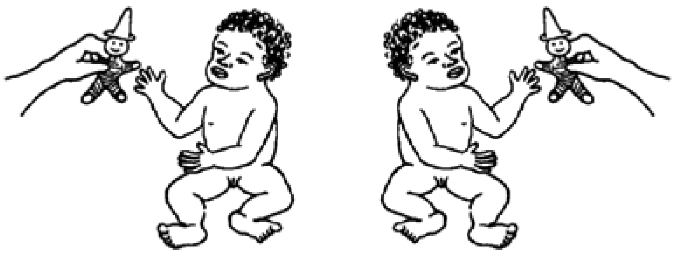

    b. Sitting on a log or seat: Lean and reach for a toy from side to side.

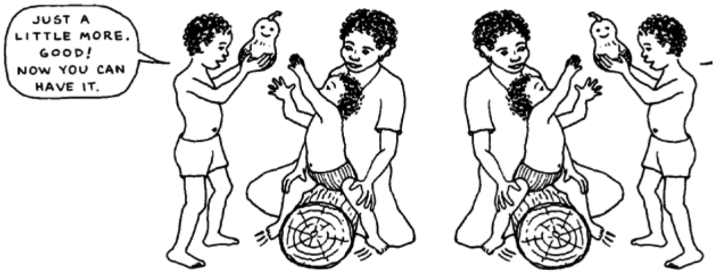

For children unable to sit up by themself:

1. Help them learn to sit up:
    a. From lying on their back. As the child starts to rise, push on the higher hip.

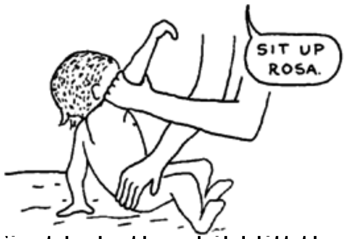

    b. From lying on their belly. First help the child lift their shoulders. Then help the child roll to one side, rise onto one elbow, and s [illegible]

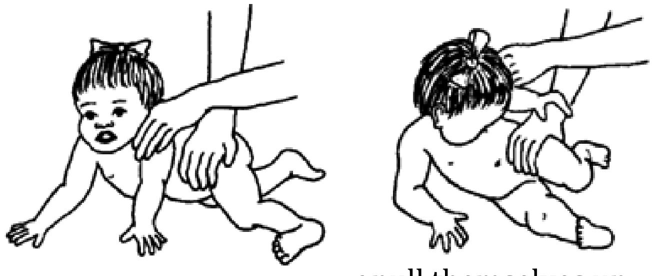

    c. **NOTE:** For some children, they will need to use a rail to push or pull themselves up independently. For an older child, who after practice cannot bring themself from lying to sitting just using their hands, give them a rail to hold onto and practice sitting up.

Movement skill: Creeping and Crawling

SUITABLE FOR ALL CHILDREN

Goal:

- To move independently by creeping and crawling to reach and grasp an object.

Need:

- Toy to grasp
- Sheet
- Tunnel – tire, large drain pipe or sheet over chairs

Instructions:

<u>For children who are unable or have some difficulty creeping and crawling:</u>

1. Put a toy just out of reach, and ask the child to go get the toy by creeping or crawling. To make more difficult, move the toy further away.

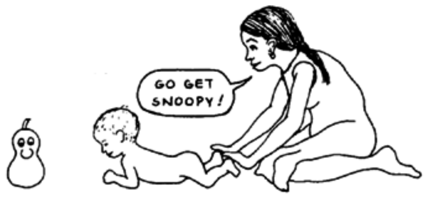

   a. If the child cannot bring their leg forward to creep, help them by lifting

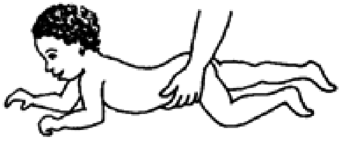

   b. If the child has trouble crawling, hold them up with a towel or sheet.

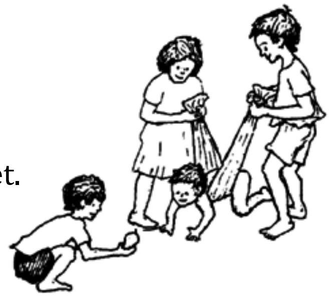

<u>For children who can crawl fairly well:</u>

1. Play crawling games. The child can crawl up and down a small hill or pile of straw. This will help improve their strength and balance.

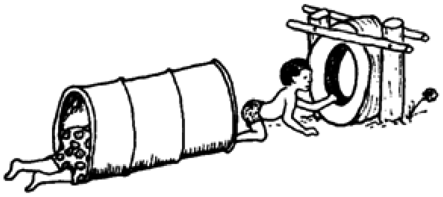

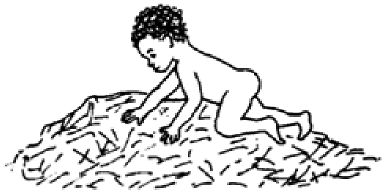

2. Encourage the child to crawl sideways and backward.

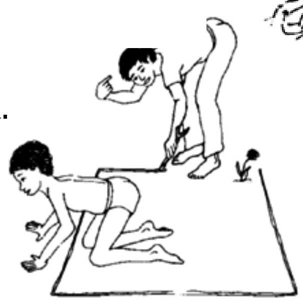

3. Have the child reaching for objects while standing on their knees and shifting their weight. Do not do this in a child with spasticity whose knees bend a lot when they stand.

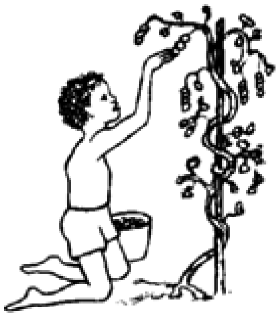

Movement Skill: Standing, Walking and Balance

NOT SUITABLE FOR ALL CHILDREN

DO THIS IF:
- The child can stand with support at their hips, or independently.

DO NOT DO THIS IF:
- The child has significant contractures/deformity where the child cannot weight bear through their lower limbs and is not able to stand.

Goal:
- Child to be able to stand independently, and walk independently, while using their hands.

Need:
- Thick rope
- Parallel bars
- Walker
- Balance board

Instructions:

For children beginning to stand:

1. Support the child's hips with your hands. Spread the child's feet apart to form a wide base. First do this from in front, later from behind. Move the child gently from side to side, so that they learn to shift their weight from one leg to the other.

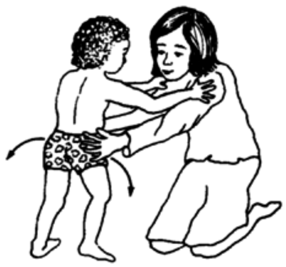

2. As the child gains better balance, you can provide a light support at the shoulde[illegible]

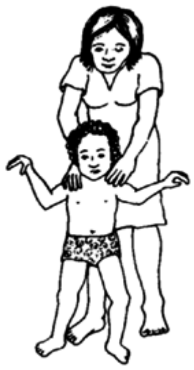

   Or, have the child hold a hose or rope. Because it is flexible, the child needs to balance more.

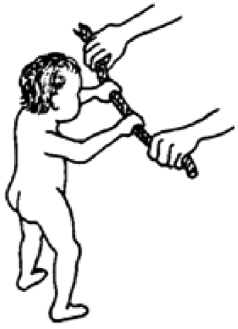

For children learning to walk:

1. Use parallel bars to help a child with weak legs or difficulty balancing on their feet, start walking.

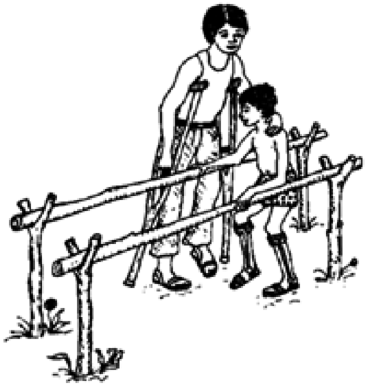

2. Support your child only as much as they need, until they can walk by themself

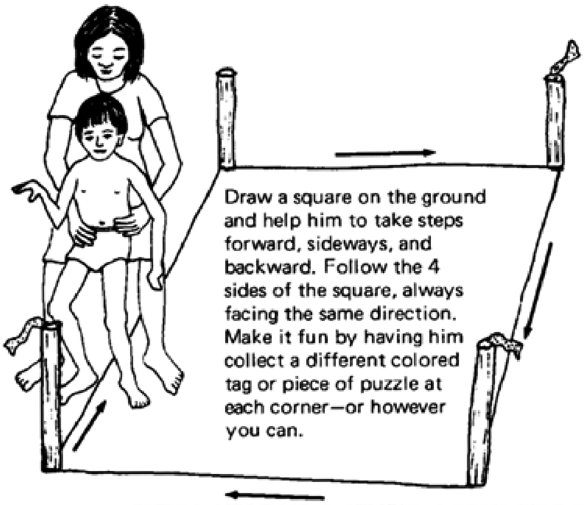

3. Use a walker to provide both support and independence for the child who is learning to walk or who has balance problems.

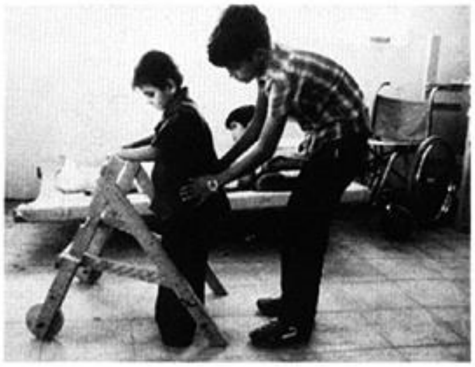

For children who tend to walk tiptoe:

1. Practice walking sideways and backward. This encourages children to bring their heels down.

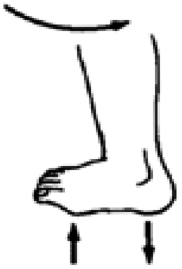

For children with poor balance:

1. Use a balance board. Move slowly at first.

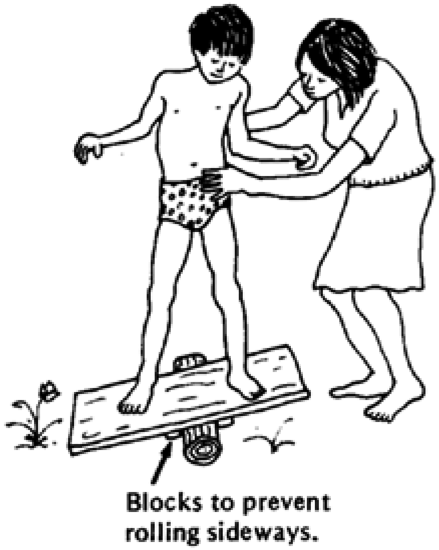

Group Movement

Goal:

- To develop gross-motor skills through social, play-based activities

While this is not listed on every instruction sheet, all group movement activities help children develop:

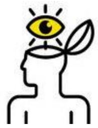

Perception: Position in space

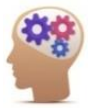

Cognition: Motor planning

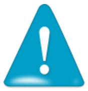

Attention: Focus on activity

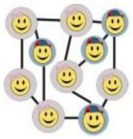

Social behaviour: Turn taking

Need:

- Movement activity instructions

Instructions:

1. Select one or two group movement activities for that session.
2. You will need to repeat group movement activities for future sessions, but make sure you are not repeating the same activity every day.
3. Make sure that you choose activities that focus on different skills throughout the week. E.g.
    a. On Sunday you choose an activity that develops balance
    b. On Monday you choose an activity that develops crawling/walking
    c. On Tuesday you choose an activity that develops upper limb strength
    d. Etc.
4. Make sure you use different environments to do the group movement activity. Do the activity outside on the concrete, outside on a soft grassy area, or inside on foam floor mats. Do not do all the activities inside.

Movement 1: Balance Beam

To develop:

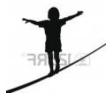

Balance

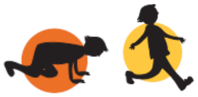

Crawling, Walking

Need:

Items children can walk on/through for balance, e.g.

- Balance board
- Tires
- Zig zag board
- Tunnels

Instructions:

1. Arrange a variety of equipment designed for the children to walk on for balance, one after the other in a series with gaps in between.
2. Instruct the children to move over, under, and through the equipment and reach the finishing point.

To make harder:

- Call out a movement like walk, crawl or scoot and the children move through the equipment using this movement
- Reduce the width of the boards
- Increase the distance between equipment

Adaptations:

- Provide hand support on one or both sides for children who are not confident while walking

Movement 2: Body Bowling

To develop:

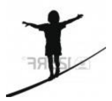

Balance

Moving isolated body parts, coordination

Need:

- Foam floor mats
- Six bowling pins (made out of 1 L soft drink bottles half filled with sand)

Instructions:

1. Tell children to start by lying down or sitting on the floor mat.
2. Arrange six bowling pins around the mat.
3. Tell the children to move to and hit each bowling pin with a named body part such as the left knee, the right heel, the head, the left elbow and so on.

Movement 3: Pendulum Bowling

To develop:

Balance

Arm and hand control and coordination

Need:

- Balloon, ball or large stuffed toy with rope attached

Instructions:

1. Suspend a balloon, ball or large stuffed toy from an overhead horizontal pipe or hook to make a pendulum.
2. Divide children into two groups, to stand in a row on either side of the pendulum.
3. Children take turns hitting the balloon/ball/toy only using their hands – one child hits the suspended toy to the opposite side and any child on the opposite side can hit it back.
4. Continue so that each child has a turn of hitting the toy first.

To make harder:

- Suspend 2 or 3 balls of different colours and each child is allowed to hit only one colour ball.
- Suspend the ball above arms reach or at a lower level that children must jump or bend a little to hit.
- Give children a plastic bat or racket to hit instead of their hands
- Reduce the participants to 2 or 4 and make them stand farther from each other

Adaptations:

- Tell children who have difficulty maintaining balance to sit or stand with support and hit
- Allow children with difficulty moving to use any body part for hitting the ball

Movement 4: Hop Scotch

To develop:

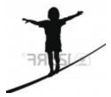

Balance

Need:

- Chalk
- Flat stone or plastic piece

Instructions:

1. Using chalk, draw a rectangular box on the ground and divide into a minimum of 2 rows and 2 columns.
2. Name each row and column with alphabets or numbers or colours.
3. Get the children to line up behind the box.
4. One by one, tell each child to throw the flat stone or plastic piece to furthest box, and then hop on single leg from one column to other without touching the border lines to the stone, pick up the stone and then come back to the starting point in the same way as they went.

To make harder:

- Draw the rectangles with different sizes and arranged irregularly
- Tell the child to balance the stone on open palm and hop
- Tell the child to put the stone on the top of their head and walk from one end to other without touching the lines

Adaptations:

- Call out the cell numbers to identify the box the child needs to move to next
- Jump with both legs or walk or crawl instead of hop

Movement 5: In the Pond – On the Bank

To develop:

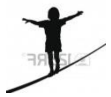

Balance

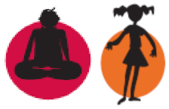

Sitting, Standing

Need:

- Chalk

Instructions:

1. Using chalk, draw a big circle on the ground and tell the children to stand around it.
2. When teacher calls “in the pond” all the children must jump inside the circle. When teacher calls “on the bank” all children must jump out of the circle. Those that don’t follow the instruction correctly, leave the game.

To make harder:

- Call the instructions “in the pond” and “on the bank” faster
- Add more actions instead of jumping, like hop or frog jump
- Make the edge of the circle six inches off the ground

Adaptations:

- Give the child one or two hand support

Movement 6: Clean Your Room

To develop:

Arm and hand control: Throwing, coordination

Sitting, Standing

Need:

- Sheet or net (to create barrier)
- Two buckets full of soft balls

Instructions:

1. Create a barrier by hanging a net, or sheets over a rope to divide the area.
2. Tell an equal number of children to stand on each side of the divider.
3. Give each side a bucket of soft balls.
4. Tell the children to tip each ball bucket over.
5. Children need to clean their room by picking up the balls and throwing them over to the other side. The game ends when all the balls are in one room, i.e. on one side of the curtain (or once you decide they have played long enough).
6. Tell children to help you pick up the balls, divide them equally into the two buckets and begin the game again.
7. Vary to throwing overhand, underhand, bowling, or throwing with the opposite hand.

To make harder:

- Use a large room so there is a larger space to search and move for the balls
- At step 6, tell the children to find as many balls as they can within one minute
- Hang the dividing curtain high so that the children have to throw higher

Adaptations:

- Give non-mobile children standers or a chair and some balls within reach
- Tell children to pass the balls to children in supported seating for throwing
- Lower the level of the dividing curtain so that children can throw the ball over it in sitting

Movement 7: Gutter Ball

To develop:

Arm and hand control: Grasp, release, catching

Crawling, Walking

Need:

- PVC pipes
- Plastic bucket
- Tennis balls

Instructions:

1. Slice PVC pipes in half along the length. Connect and support various lengths of plastic pipe to create a slightly sloping chute through which tennis balls roll.
2. Place a large plastic bucket at the end of the chute.
3. Instruct each child to place a tennis ball at the top of the pipe and let it roll, and then try to stop and grab it before it reaches the bucket at the bottom.
4. To make it a group game, instruct one child to release several balls, one after the other, and the other children have to try and grab a ball before it reaches the bucket.

To make harder:

- Use PVC pipe end pieces to change direction of the rolling ball
- Add more pipes so the distance to move is greater
- Increase the slope so the ball rolls faster
- Challenge children to use left and then right hand alternately

Adaptations:

- Use weighted balls or reduced slope to reduce the speed for children who have difficulty moving or reaching
- Use wheelchairs or prone standers for children who have difficulty moving or standing

Movement 8: Mat Maze

To develop:

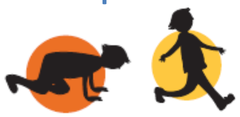

Movement: Body control, moving

Need:

- Foam floor mats

Instructions:

1. Create a maze with mats. Make some turns in the path which lead to a dead end. Only one way should take you through the maze.
2. One by one, tell the children to find their way through the maze to the end.
3. Tell children to repeat the maze.

To make harder:

- Create tunnels, steps and slides and other changes in levels
- Have children find ‘treasures’ like stuffed animals along the way

Adaptations:

- Use wider pathways for children who use assistive devices for mobility inside the maze
- Use auditory clues to help children orient themselves and find their way through the maze

Movement 9: Hand over Hand Pull

To develop:

Arm and hand control: Strength, grasp, coordination

Need:

- Plastic bottle or bag
- Rope
- Paper plate

Instructions:

1. Attach a large plastic bottle or bag to a rope and pulley
2. Attach a plate on top of the pulley
3. One by one, instruct each child to lift the bottle up until it bumps the plate by pulling the rope hand over hand.
4. After the bottle touches the plate, tell the child to release the rope and lets the plastic bottle fall to the ground.
5. Repeat.
6. Increase or decrease the weight of the bottle by filling it with different things.

To make harder:

- Increase the weight of the bottle to make it harder to lift
- Challenge children to pull up and drop the bottle as many times as they can within a minute

Adaptations:

- Tie a knot every few inches of the rope so that it is easy for the child to pull
- Use adapted/supported chairs or prone standers for children who have difficulty moving or standing
- Place bottle higher to reduce the height that must be pulled

Movement 10: Mountains and Valleys

To develop:

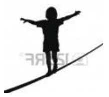

Balance

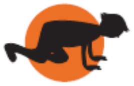 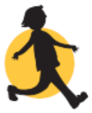

Lower body strength, crawling, walking

Need:

- Foam floor mats
- Tires

Instructions:

1. Arrange mats and tires to create different levels around a room.
2. Instruct children to move around the room climbing, jumping, rolling, balancing, crawling and sliding over the mats.
3. Alternatively, go outside to a grassy hill and climb, jump, roll, crawl and slide up/down the hill.

To make harder:

- Add tires and mats to increase the height of the mountains
- Place stuffed toys on top of the mountains and have the children collect them and carry them back in a sling bag or back pack

Adaptations:

- Children with limited mobility can roll or crawl over the mats
- Allow children who hesitate to climb to crawl up and down the mats to become familiar with the terrain

Movement 11: Active Alphabet

To develop:

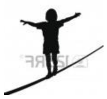

Balance

Body strength and control

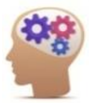

Cognition: Letter recognition

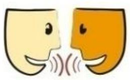

Communication: Vocabulary

Need:

- No equipment

Instructions:

1. Have children stand in their own personal space.
2. As you say the alphabet together, say a word for each letter and have students act out that word e.g. A for airplane, fly like an airplane around the room

Movement 12: I Woke Up in the Morning

To develop:

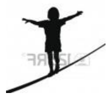

Balance

Body control

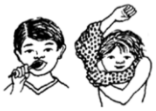

Activities of Daily Living: Morning routine

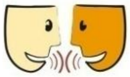

Communication: Vocabulary

Need:

- Foam floor mats

Instructions:

1. Tell the children to lie on their backs quietly on the floor.
2. Say the following rhyme as students do the corresponding movements:
   I woke up in the morning (students yawn and raise arms)
   And I jumped out of bed (jump up)
   Put some clothes on my body (pretend to get dressed)
   And a hat on my head (pretend to put a hat on)
   Took a leap to the kitchen (large step)
   And I ate my toast (pretend to eat)
   Listened to the news (hand on ear)
   From coast to coast (arm from one side to the other)
   Looked out the window (hand shielding eyes)
   To check the sun
   Ran out the door (run on the spot)
   To have some fun. (do a fun jumping jack)

Movement 13: The Mr. Man Game

To develop:

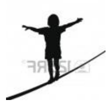

Balance

Body control

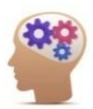

Cognition: Following commands

Need:

- No equipment

Instructions:

1. Tell children to stand in their personal space.
2. Call out the following commands, and demonstrate the actions for the children to copy. After the children have performed this activity numerous times and had the opportunity to learn the actions, do not demonstrate, make the children try and remember and show you.
    a. Mr. Slow - move slowly around the room.
    b. Mr. Rush - move fast on the spot.
    c. Mr. Jelly - shake your whole body.
    d. Mr. Muddle - take three steps backward slowly.
    e. Mr. Bounce - jump up and down on the spot until the next command is called.
    f. Mr. Small - crouch low and waddle around.
    g. Mr. Strong - move flexing your muscles.
    h. Mr. Tall - stretch up as high as you can.

Movement 14: Walk to School

To develop:

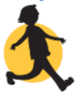

Walking, running

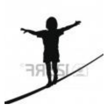

Balance

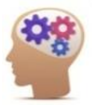

Cognition: Following commands

Need:

- Handrail

Instructions:

1. Tell the children to stand in a circle.
2. Read the following rhyme and demonstrate the actions for the children to say and copy. After the children have performed this activity numerous times and had the opportunity to learn the actions, do not demonstrate, make the children try and remember and show you.

Walk, walk, walk to school (walk around in a circle)
Walk to school together.
Walk, walk, walk to school
Walk to school and stop. (children freeze on the word stop)
Run, run, run to school (children run on the spot)
Run to school together.
Run, run, run to school
Run to school and stop. (children freeze on the word stop)
Hop, hop, hop to school (children hop in a circle)
Hop to school together.
Hop, hop, hop to school
Hop to school and stop. (children freeze on the word stop)
Skip, skip, skip to school (children skip in a circle)
Skip to school together.
Skip, skip, skip to school
Skip to school and stop. (children freeze on the word stop)
Jog, jog, jog to school (children jog on the spot)
Jog to school together.
Jog, jog, jog to school
Jog to school and stop. (children freeze on the word stop)
Tiptoe, tiptoe, tiptoe to school (students tiptoe on the spot)
Tiptoe to school together.
Tiptoe, tiptoe, tiptoe to school
Tiptoe to school and stop. (children freeze on the word stop)

Movement 15: Shape Up

To develop:

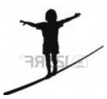

Balance

Body control

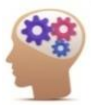

Cognition: Following commands

Need:

- No equipment

Instructions:

1. Tell children to stand in their own personal space.
2. Say the following rhyme and demonstrate the actions for children to also say and copy. After the children have performed this activity numerous times and had the opportunity to learn the actions, do not demonstrate, make the children try and remember and show you.

We're jumping up and down
We're jumping up and down
We're getting lots of exercise
We're jumping up and down.

We bend and touch our toes
We bend and touch our toes
We're getting lots of exercise
We bend and touch our toes.

We kick our legs up high
We kick our legs up high
We're getting lots of exercise
We kick our legs up high.

We wiggle our whole body
We wiggle our whole body
We're getting lots of exercise
We wiggle our whole body.

We stretch up to the sky
We stretch up to the sky
We're getting lots of exercise
We stretch up to the sky.

Movement 16: I Can

To develop:

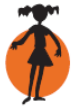

Standing

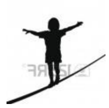

Balance

Body control

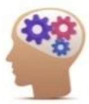

Cognition: Following commands

Need:

- Chairs

Instructions:

1. Tell children to stand in their own space or in a circle.
2. Say the following poem, having children perform the appropriate actions:
   I nod my head and clap my hands
   And then I turn around
   I stretch up high, I bend down low
   Then I sit upon the ground
   I touch my toes while sitting down
   Then jump up in the air
   I walk around in a circle
   Then sit quietly on a chair.
3. Do the actions faster and faster as the poem is recited more quickly each time.

Movement 17: Wiggles

To develop:

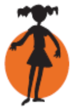

Standing

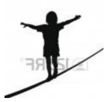

Balance

Body control

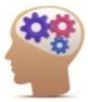

Cognition: Identifying body parts

Need:

- No equipment

Instructions:

1. Tell the children to lie or stand, while doing the following activities:
    a. Tell the children to wiggle their fingers.
    b. Then their fingers and wrists.
    c. Then their fingers, wrists, and forearms.
    d. Then their fingers, wrists, forearms, and elbows.
    e. Then their fingers, wrists, forearms, elbows, and shoulders.
    f. Then their fingers, wrists, forearms, elbows, shoulders, and rib cage.
    g. Then their fingers, wrists, forearms, elbows, shoulders, rib cage, and hips.
    h. Then their fingers, wrists, forearms, elbows, shoulders, rib cage, hips, and knees.
    i. Then their fingers, wrists, forearms, elbows, shoulders, rib cage, hips, knees and head.

Movement 18: I’m Tall, I’m Small

To develop:

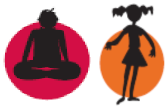

Sitting, Standing

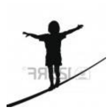

Balance

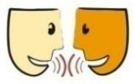

Language and communication: Vocabulary

Need:

- No equipment

Instructions:

1. Select one child to stand in front of the group as the leader.
2. Select another child to be the guesser, and turn around and close their eyes tight.
3. All children repeat the following verses:
   “I’m tall, very tall – all children stand or sit tall.
   I’m small, very small – all children squat or lie very low.
   Sometimes I am tall – all children stand or sit.
   Sometimes I am small – all children squat or lie low.
   Guess what I am now” – the leader either stands tall or goes small and all children follow their lead.
4. The guesser must now guess whether the class is tall or small.
5. If he/she guesses correctly they become the new leader, if not then pick a new guesser and the leader stays the same.

Movement 19: Up, Down, Stop Go

To develop:

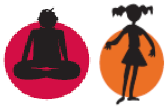

Sitting, Standing

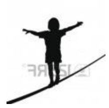

Balance

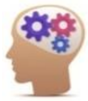

Cognition: Following commands, opposites

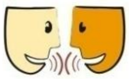

Language and communication: Vocabulary

Need:

- No equipment required

Instructions:

1. Tell children to stand in their personal space.
2. Explain to the children that they must listen very carefully and use their opposites.
3. Call out the following commands while the children perform the corresponding actions:
   Go – Children must STOP.
   Stop - Children must MOVE.
   Up - Children LIE down.
   Down – Children STAND, JUMP up to the ceiling or SIT tall.

Movement 21: The Number March

To develop:

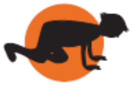 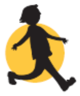

Crawling, Walking

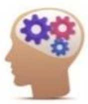

Cognition: Counting

Need:

- No equipment

Instructions

1. Tell children to sit or stand in a circle.
2. Say the following rhyme, children to march on the spot or sit and clap a beat, until you select them to march or crawl around the circle.

One person march around the circle; (select one person)
March, march, march. (everyone is marching on the spot)
One person march around the circle
Now, let's have two (select another person)

Two people march around the circle;
March, march, march (make sure everyone is marching on the spot)
Two people march around the circle,
Now let's have three. (choose a third person)

Three people march around the circle;
March, march, march (make sure everyone is marching on the spot)
Three people march around the circle,
Now let's have four. (choose a fourth person)

Four people march around the circle;
March, march, march (make sure everyone is marching on the spot)
Four people march around the circle,
Now let's have five. (choose a fifth person)

There are five people marching,
Round and around,
1, 2, 3, 4, 5 people march
There are five people marching,
Round and around,
1, 2, 3, 4, 5 people march.

Five people march around the circle;
March, march, march.
Five people march around the circle,
Now let's have four. (take one person away)

Four people march around the circle;
March, march, march (make sure everyone is marching on the spot)
Four people march around the circle,
Now let's have three. (take another person away)

Three people march around the circle;
March, march, march (make sure everyone is marching on the spot)
Three people march around the circle,
Now let's have two. (take another person away)

Two people march around the circle;
March, march, march (make sure everyone is marching on the spot)
Two people march around the circle,
Now let's have one. (take another person away)

One person march around the circle;
March, march, march
One person march around the circle
Now there is none.

Movement 22: Balloon Football

To develop:

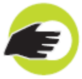

Arm and hand control: Strength and coordination

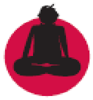

Sitting

Need:

- Balloon

Instructions:

1. Divide the class into two teams.
2. Children sit on the floor or in their chairs to form two lines facing each other 4 to 6 feet apart.
3. The balloon or beach ball is tossed between the two teams. Both teams try to bat it over the heads of their opponents so that the ball touches the floor behind the opposing team. Each touchdown scores a point. One child should be placed behind each team to serve as scorekeeper and ball retriever. The balloon should be put into play at different places along the two lines to prevent action from being concentrated among a few players.

Movement 23: Balloon Volleyball

To develop:

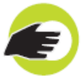

Arm and hand control: Strength and coordination

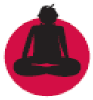
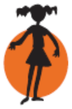

Sitting, Standing

Need:

- Two balloons
- Rope

Instructions:

1. Divide the class into two teams
2. Children stand or sit on the floor or in their chairs on both sides of a rope stretched just above their reach.
3. The children try to bat a balloon back and forth across the rope.

Movement 24: Classroom Mousetrap

To develop:

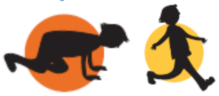

Crawling, Walking

Need:

- No equipment

Instructions:

1. Select two children to join hands and hold them up high to form an arch. This is the mousetrap.
2. Other children are the mice, and form a line and move through the arch. Children can crawl, walk or run.
3. The arch remains up until the teacher says, “Snap”; then they are brought down. Anyone caught forms additional arches.
4. Play continues until there is only one child left.

Movement 25: Flag Chase

To develop:

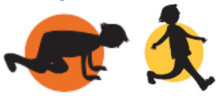

Crawling, Walking, Running

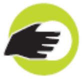

Arm and hand control: Grasp and release

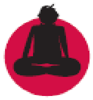

Sitting

Need:

- Four flags
- Foam mat
- Chairs
- Four markers

Instructions:

1. Divide the class into two even teams
2. Each team to sit (on the floor or on chairs) in a straight line, with a marker 3 feet in front of each team
3. In each team, the child at the left end has a flag.
4. When the teacher says “Go”, the player with the flag moves (crawls, walks, runs) as fast as possible to the marker, goes around the marker and moves to sit on the right end of the team. The flag is them passed down the line to the left, and the child now at the left end moves with the flag around the marker and to the right end.
5. The race ends when all children in each team have taken the flag around the marker.

Movement 26: Overhead Relay

To develop:

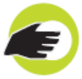

Arm and hand control: Grasp and release, strength and coordination

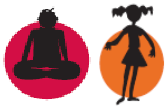

Sitting, Standing

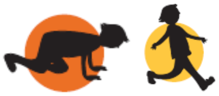

Crawling, Walking, Running

Need:

- Object to pass
- Foam floor mats

Instructions:

1. Children form a straight line, sitting on the floor, all facing one direction.
2. The first child in the line has the object to be passed on the floor in front of them.
3. When the teacher says "Go", the first child claps hands, picks up the object and passes it overhead to the child behind. The next child places the object on the floor, claps hands, and passes the object overhead to the child behind. The last child in the row receives the object and moves as quickly as possible (crawling, walking, running) to the front of the line.
4. The child who has come to the front then sits down, places the object on the floor in front of them, claps hands and passes the object overhead to the child behind.
5. This continues until the children are back in their original order.

Movement 27: Human Obstacle Course

To develop:

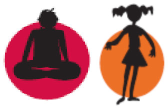

Sitting, Standing

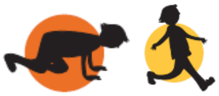

Crawling, Walking

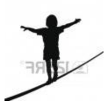

Balance

Need:

- Bicycle tire
- Foam floor mats

Instructions:

1. Divide the class in two groups. Half the class will be an obstacle and half will be moving around them, and then they will switch. Allocate one of the following positions to each child in the obstacle group:
    a. Lies down on stomach or back
    b. Makes a tunnel by standing with legs spread out wide
    c. Holds a bicycle tire up on the floor
    d. Holds body in a cat position (as per yoga)
    e. Holds body in a mouse position (as per yoga)
2. The other half of the class performs the activity by working through these human obstacles by maneuvering around, over, or under the obstacles. Give children time to figure out how to move themselves. If they are not able to figure out a way, you can give them suggestions:
    a. Leap or jump over the person laying down
    b. Crawl through the tunnel
    c. Jump through the tire
    d. Crawl under the cat, or put hands on back and bring legs over the cat
    e. Leap over the mouse
3. Swap

Movement 28: Hokey Pokey Aerobics

To develop:

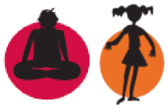

Sitting, Standing

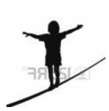

Balance

Body control

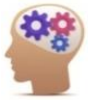

Cognition: Identifying body parts, following commands

Need:

- Foam floor mats

Instructions:

1. Form a circle
2. Say the following rhyme together and do the actions (the actions in red are more difficult and suitable for children able to walk):
    a. Put your right foot in (lunge forward on the right leg)
    Put your right foot out (return to standing position)
    Put your right foot in and shake it all about (lift right leg and shake it, or lunge forward on the right leg and lightly bounce up & down in lunge position)
    Do the Hokey Pokey and turn yourself about (move around in a circle, or do jumping jacks while turning in a circle)
    b. Repeat above but do the opposite for the left leg
    c. Put your right arm in (extend arm at the elbow)
    Put your right arm out (flex arm at the elbow)
    Put your right arm in and shake it all about (lift arm and shake)
    Do the Hokey Pokey and turn yourself about (move around in a circle, or do vertical jumps while turning in a circle)
    d. Do the opposite for the left arm
    e. Put your right elbow in (elbow flexed, forearm parallel to the ground)
    Put your right elbow out (elbow flexed, forearm parallel to the ground)
    Put your right elbow in and shake it all about (flexed elbow making circles)
    Do the Hokey Pokey and turn yourself about (move around in a circle, or hop on right foot while turning in a circle)
    f. Do the opposite for the left elbow
    g. Put your head in (slowly touch chin to chest)
    Put your head out (slowly move it back to starting position)
    Put your head in and shake it all about (slowly move head side to side)
    Do the Hokey Pokey and turn yourself around (move around in a circle, or jog in place while turning in a circle)
    h. Put your right hip in (tilt to raise right hip, or side jump in)
    Put your right hip out (lower right hip to normal position, or side jump out)
    Put your right hip in and shake it all about (move hips from side to side, or jump side to side)
    Do the Hokey Pokey and turn yourself around (move around in a circle)
    i. Do the opposite for the left hip
    j. Put your whole self in (move body forward – crawl, step or jump in)
    Put your whole self out (move body backward)
    Put your whole self in and shake it all about (move body in and out)
    Do the Hokey Pokey and turn yourself around (move around in a circle)
    k. Put your backside in backwards (move backside in – crawl, step or jump backward)
    Put your backside out (move backside out)
    Put your backside in and shake it all about (move in and out)
    Do the Hokey Pokey and turn yourself around (move around in a circle)

Movement 29: Bottle ball

To develop:

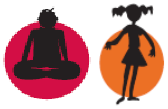

Sitting, Standing

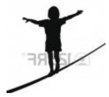

Balance

Body control

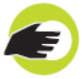

Arm and hand control: Throwing and catching

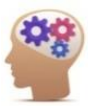

Cognition: Identifying body parts, following commands

Need:

- Empty plastic bottles
- Foam floor mats

Instructions:

1. Give each child an empty plastic bottle
2. Say and instruction and children follow:
    a. Dribble the bottle using their right foot
    b. Dribble the bottle using their left foot
    c. Dribble the bottle using the inside of their foot
    d. Dribble the bottle using the outside of their foot
    e. Move forward over the bottle
    f. Move backward over the bottle
    g. Move side-to-side over the bottle
    h. Try and steal other children's bottles! Make sure you protect your own.
    i. Roll, toss and catch the bottle to each other

Movement 30: The Run-Around

To develop:

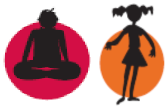

Sitting, Standing

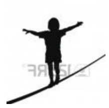

Balance

Body control

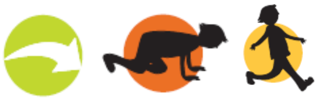

Rolling, Crawling, Walking

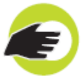

Arm and hand control: Grasp and release

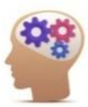

Cognition: Following commands

Need:

- No equipment

Instructions:

1. Give directions for children to follow:
    a. Roll three times
    b. Crawl to the door and stand up
    c. Walk to the tree, touch the bark and come back
    d. Jump three times
    e. Go to the bookshelf and pick up two books
    f. Etc.

Movement 31: Spoon Races

To develop:

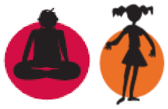

Sitting, Standing

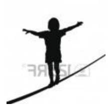

Balance

Body control

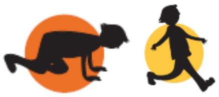

Crawling, Walking

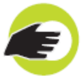

Arm and hand control: Grasp and release

Need:

- Spoons
- Small object to balance on spoon
- Foam floor mats, or soft grassy area

Instructions:

1. Split the class into two groups
2. Have each group sit in a line at opposite ends of the room
3. Give one child at the front of each line a spoon and an object to balance on the spoon in a bucket. The child holds the spoon, picks up the object with the spoon, and then moves to the group at the other end of the room while balancing the object on the spoon. Then puts the object in the bucket and hands the spoon to the next child.
4. The next child does the same.
5. Continue so that all children have a turn.

Alternatives:

1. Using small balls, children push the balls using only their noses, on their hands and knees, while moving to the other side of the room.
2. Using small bags filled with flour or rice, children balance these bags on their head while moving to the other side of the room.

Movement 32: We All Play Ball

To develop:

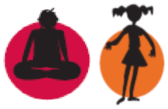

Sitting, Standing

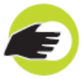

Arm and hand control: Throwing and catching

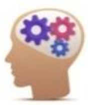

Cognition: Reciting alphabet, numbers etc.

Need:

- Ball

Instructions:

1. Roll or pass a ball back and forth while saying the alphabet, counting forward or backward, saying different colours, shapes, foods etc.
2. Roll, throw over-hand, underhand, with one hand, with two hands, bouncing, the ball to each other.

Movement 33: Toe Pick-Up

To develop:

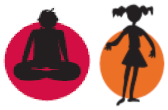

Sitting, Standing

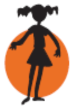

Leg and feet movement and control

Need:

- Five small objects for each child
- Container for each child

Instructions:

1. Children to pick up small objects with their toes and put them in a container.
2. To make more difficult cognitively, instruct the child to pick up the pink object, then the largest object etc.

Movement 34: Red Light, Green Light

To develop:

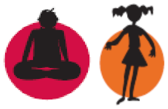

Sitting, Standing

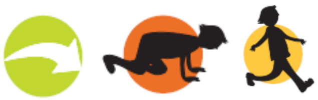

Rolling, Crawling, Walking

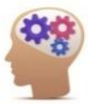

Cognition: Following commands

Need:

- Foam floor mats

Instructions:

1. Select one child to be the traffic light. They sit/stand at one side of the room.
2. The rest of the children sit/stand in a line at the other side of the room.
3. The stop light calls “green light”, and the children move toward the stop light. The stop light calls “red light” and the children must stop.
4. Continue so that all children have turns at being the stop light.

Variations:

- “Yellow light” can mean walk slowly, “blue light” can mean hop, “purple light” can mean walk backwards etc.

Movement 35: Basketball

To develop:

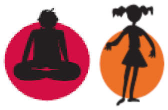

Sitting, Standing

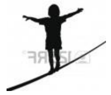

Balance

Body control

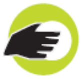

Arm and hand control: Strength, throwing, coordination

Need:

- Different sized balls
- Bin/bucket

Instructions:

1. Children take turns shooting the ball toward the bucket using different sized balls and at different distances from the bucket.

Movement 36: What time is it Mr. Fox?

To develop:

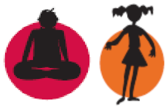

Sitting, Standing

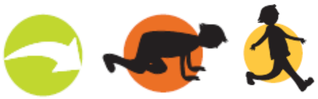

Rolling, Crawling, Walking

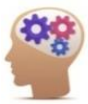

Cognition: Time, counting

Need:

- Foam floor mats or soft grassy area

Instructions:

1. Select one child to be Mr Fox. They sit/stand at one side of the room with the back to the rest of the group.
2. The rest of the children sit/stand in a line at the other side of the room.
3. The children ask "what time is it, Mr. Fox?"
4. Mr. Fox answers with a time.
5. If he says two o'clock, the other players must roll/crawl or take two steps toward Mr. Fox. Etc.
6. Once a child gets close enough to touch Mr. Fox, the fox turns around and tries to catch one of the players.
7. The players move back as fast as they can to the starting line.

Movement 37: Four Square

To develop:

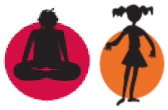

Sitting, Standing

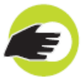

Arm and hand control: Strength, throwing, coordination

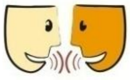

Language and communication: Reading numbers, words, recognising shapes

Need:

- Chalk
- Ball

Instructions:

1. Using chalk draw a large square made up of four squares.
2. In each of the four squares, draw a picture, or write a letter or number.
3. Take turns trying to bounce the ball into each square, saying the picture/letter/number inside the square.

Movement 38: Parachute

To develop:

Sitting, Standing

Arm and hand control and coordination

Need:

- Old bed-sheet
- Small balls or balloons

Instructions:

1. Put balls or balloons on top of the bed-sheet
2. Children hold onto the edges of the bed-sheet and wave their arms up and down so that the balls/balloons bounce on top.
3. Can take turns to crawl under the bed-sheet.
4. Can also take turns sitting on the bed-sheet and other children pull them around the room.

Movement 39: Scoops/Catchers

To develop:

Sitting, Standing

Arm and hand control

Need:

- Empty laundry detergent containers (that has one handle), with the bottom cut out and edges covered with tape/sanded
- Small balls

Instructions:

1. Children use scoops to toss and catch small balls to each other.

Movement 40: Goalie Target

To develop:

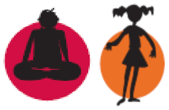

Sitting, Standing

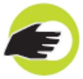

Arm and hand control: Strength, throwing, coordination

Need:

- Old sheet
- Soft balls/balloons

Instructions:

1. Hang up the sheet. Make sure there is nothing fragile behind it.
2. Select one child to be the goalie.
3. Other children try and throw/roll their balls/balloons into the goal and the goalie has to try and stop the ball/balloon from hitting the sheet.

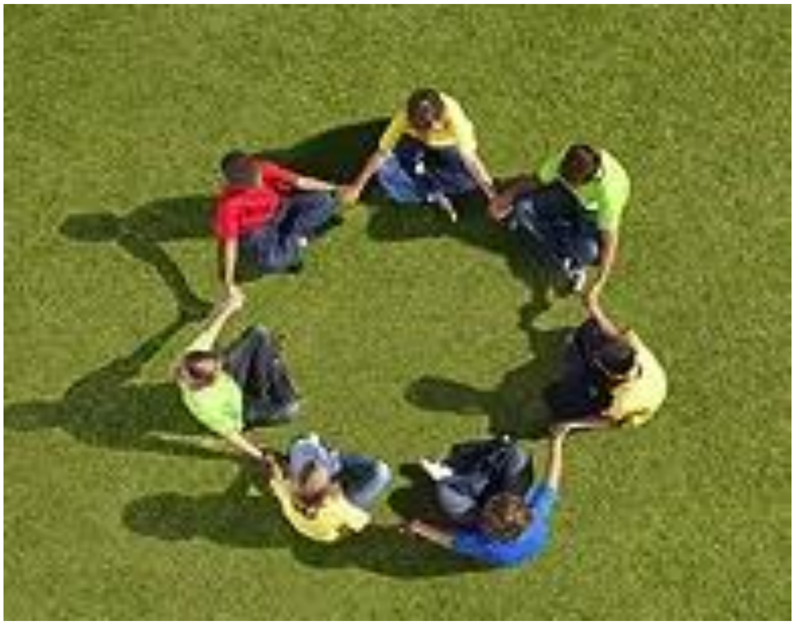

Goodbye Circle and Homework

Helps develop these Mental Skills:

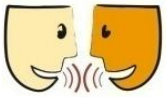
Language and communication

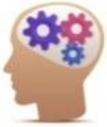
Cognition

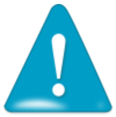
Attention

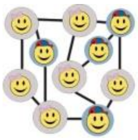
Social behaviour

Helps develop these Physical Skills:

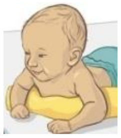
Head and trunk control

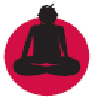
Sitting

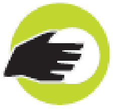
Arm and hand control

Goodbye Circle and Homework

To develop:

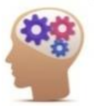 Cognition: Recalling and sequencing the activities and things learnt that day

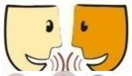 Language and communication

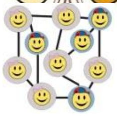 Social behaviour: Turn taking

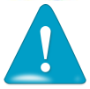 Attention: Active listening, focus and participation

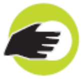 Arm and hand control and coordination

Need:

- Activity and picture cards
- Foam floor mats
- Supported chairs or cushions to help children sit independently
- Goodbye Song (e.g. Onek Dhonnobad from Music Therapy, 'Shishu Shorgo Gan ' CD) or rhyme

Instructions:

1. Children to sit in a circle.
2. Use the special seats or cushions to support children in sitting.
3. Practice reading the clock. Tell the children it's 11:45am or 4:45pm, and therefore it's time to pack up and get ready to go home.
4. Instruct children to pack up their class activity. Encourage and facilitate children to be able to participate as independently as possible (refer to Nasta break, 'Cleaning-up the Classroom' for more ideas)
5. Instruct children to sit in a circle.
6. Ask children to recall the activities they did that day. Use activity and picture cards to help them communicate and sequence the activities.
7. Ask children to recall the things they learnt that day. Revise the letters, words and numbers they learnt that day.
8. Remind children of their homework for the following day. Homework includes practicing the words they learnt, practicing activities of daily living, and movement. Remind parents to facilitate their child's learning at home. Refer to the class activity regarding additional homework required in preparation for the class activity the following day.
9. Sing a Goodbye song (e.g. Onek Dhonnobad) or rhyme. Get children to wave goodbye to each other.

Child's Name: ________________________ Date progress report completed: ______________ Completed by who: __________________

Group Therapy Progress Report

Month 1: Who Am I

Skills		What the child is able to do	What the child is learning to do
Activities of Daily Living	Eating		
	Dressing		
	Toileting		
Language and Communication (Understanding and expressing words and numbers)	Reading		
	Writing		
	Verbal vocabulary		
	Listening		
	AAC		

Skills		What the child is able to do	What the child is learning to do
Movement	Head control, Rolling, Sitting, Crawling, Walking		
	Arm and hand control (reaching, grasping, handwriting)		
Cognition	Memory		
	Counting		
	Problem solving		
	Identifying objects and understanding their use		
Social			

Other comments:

____

____

____

Child's Name: ______________________ Date progress report completed: ____________ Completed by who: _______________

Group Therapy Progress Report<br>Month 2: My Family and Friends			
Skills		What the child is able to do	What the child is learning to do
Activities of Daily Living	Eating		
	Dressing		
	Toileting		
Language and Communication (Understanding and expressing words and numbers)	Reading		
	Writing		
	Verbal vocabulary		
	Listening		
	AAC		

Skills		What the child is able to do	What the child is learning to do
Movement	Head control, Rolling, Sitting, Crawling, Walking		
	Arm and hand control (reaching, grasping, handwriting)		
Cognition	Memory		
	Counting		
	Problem solving		
	Identifying objects and understanding their use		
Social			

Other comments:

____

____

____

Child’s Name: ________________________ Date progress report completed: _____________ Completed by who: ________________

Group Therapy Progress Report<br>Month 3: The World Around Me			
Skills		What the child is able to do	What the child is learning to do
Activities of Daily Living	Eating		
	Dressing		
	Toileting		
Language and Communication (Understanding and expressing words and numbers)	Reading		
	Writing		
	Verbal vocabulary		
	Listening		
	AAC		

Skills		What the child is able to do	What the child is learning to do
Movement	Head control, Rolling, Sitting, Crawling, Walking		
	Arm and hand control (reaching, grasping, handwriting)		
Cognition	Memory		
	Counting		
	Problem solving		
	Identifying objects and understanding their use		
Social			

Other comments:

____

____

____

Child's Name: ______________________ Date progress report completed: ____________ Completed by who: _______________

Group Therapy Progress Report<br>Month 4: School and Productivity			
Skills		What the child is able to do	What the child is learning to do
Activities of Daily Living	Eating		
	Dressing		
	Toileting		
Language and Communication (Understanding and expressing words and numbers)	Reading		
	Writing		
	Verbal vocabulary		
	Listening		
	AAC		

Skills		What the child is able to do	What the child is learning to do
Movement	Head control, Rolling, Sitting, Crawling, Walking		
	Arm and hand control (reaching, grasping, handwriting)		
Cognition	Memory		
	Counting		
	Problem solving		
	Identifying objects and understanding their use		
Social			

Other comments:

____

____

____

Part 3:

COMMUNITY FOLLOW-UP

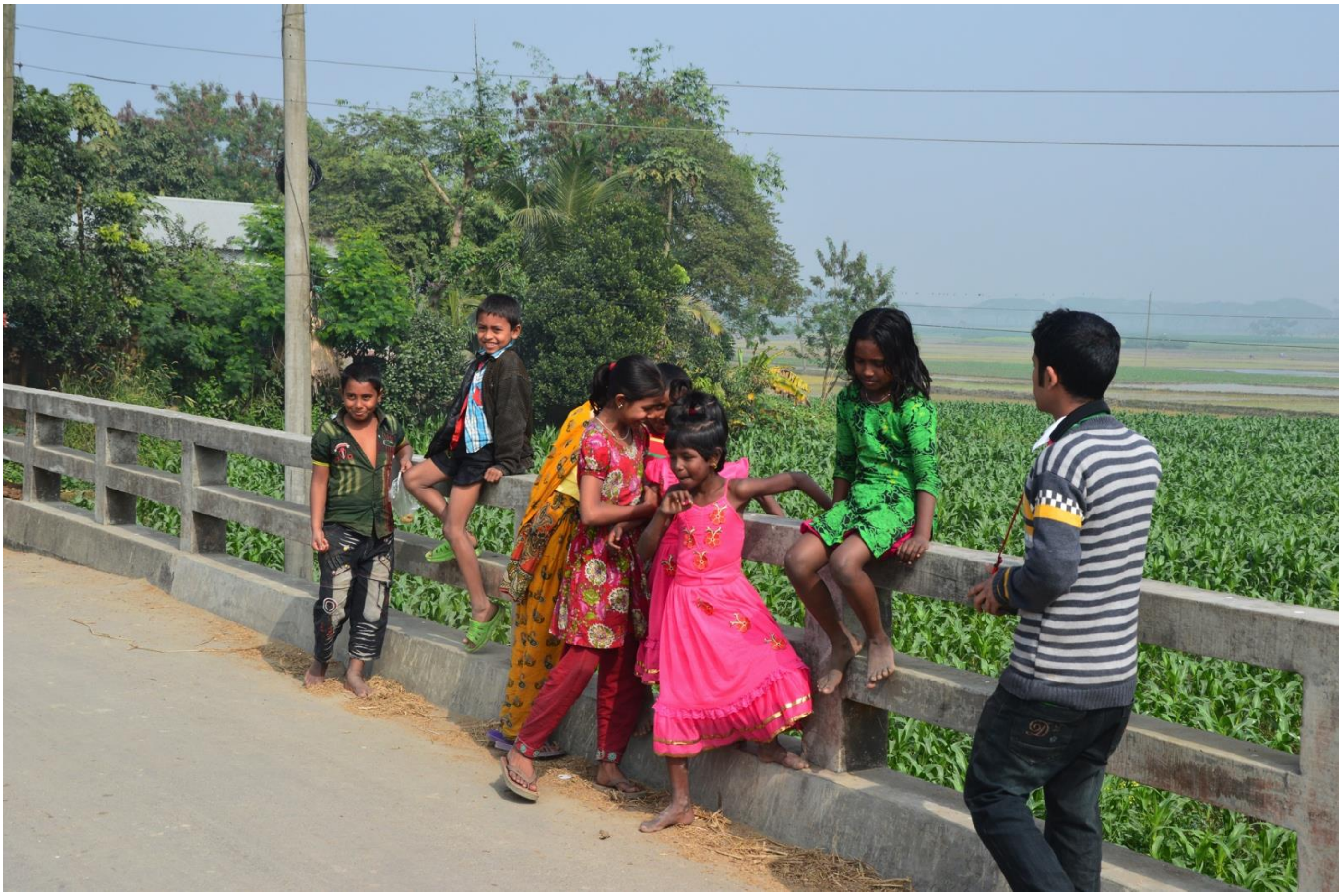

Community Follow-up Checklist

MONTH 1

1. Home Visit by Fieldworker

Purpose: To understand the environmental restrictions to the child's participation in their home and village.

Person responsible: Fieldworker

- ☐ Complete the **Home Visit Checklist**
- ☐ Identify the child's transition goal using the flowchart below:

Is the child under 14 years old?

Yes

No, the child over 14 years old

Is the child and family's goal for admission to school?

Is the child capable of attending a class appropriate for their age group?

Yes

No

Goal = Participation in School

Goal = Vocational Skills Training

2a. School Visit by Fieldworker and Teacher

2b. Continue to Month 2

Purpose: Introductions and goal setting.

Person responsible: Teacher and Fieldworker

☐ Complete **School Visit 1 Checklist**

3. Follow-up Home and Community Visits (for all children)

☐ Conduct follow-up home visits to commence home modifications and assistive devices. Modifications and assistive devices need to be completed over the 6-month transition program. Use the **Home and Community Environment and Participation Action Record** to record progress.

MONTH 2:

1. Home Visit by Fieldworker

Purpose:

1. To understand the opportunities provided for the child to participate in their home and community.
2. To continue home modifications and assistive devices.

Person responsible: Fieldworker

☐ Complete **Home and Community Participation Checklist**

☐ Continue home modifications and assistive devices. Modifications and assistive devices need to be completed over the 6 months. Use the **Home and Community Environment and Participation Action Record** to record progress.

☐ Commence home participation training. Strategies need to be implemented over the 6-month transition program. Use the **Home and Community Environment and Participation Action Record** to record progress.

2. School Visit or Vocation Visit by Fieldworker

Goal = Admission to School

↓

School Visit

☐ Complete **School Visit 2 Checklist**

Purpose: Prepare school environment for child's admission

Person responsible: Fieldworker

Use the **School Environment and Participation Action Record** to record progress.

Goal = Vocational Skills Training

↓

Vocation Planning

☐ Complete **Vocation Planning 2 Checklist**

Purpose: To identify a suitable vocation for the child

Person responsible: Fieldworker

Use the **Vocation Environment and Participation Action Record** to record progress.

MONTH 3:

1. Home Visit by Fieldworker

☐ Continue home modifications and assistive devices. Modifications and assistive devices need to be completed over the 6 months. Use the **Home and Community Environment and Participation Action Record** to record progress.

☐ Continue home participation training. Strategies need to be implemented over the 6-month transition program. Use the **Home and Community Environment and Participation Action Record** to record progress.

2. School Visit or Vocation Visit by Fieldworker

Goal = Admission to School

↓

School Visit

☐ Complete **School Visit 3 Checklist**

Purpose: Introduce child to school

Person responsible: Fieldworker

Use the **School Environment and Participation Action Record** to record progress.

Goal = Vocational Skills Training

↓

Vocation Planning

☐ Complete **Vocation Planning 2 Checklist**

Purpose: Plan for how the child will participate in their vocation

Person responsible: Fieldworker

Use the **Vocation Environment and Participation Action Record** to record progress.

MONTH 4:

1. Home Visit by Fieldworker

☐ Continue home modifications and assistive devices. Modifications and assistive devices need to be completed over the 6 months. Use the **Home and Community Environment and Participation Action Record** to record progress.

☐ Continue home participation training. Strategies need to be implemented over the 6-month transition program. Use the **Home and Community Environment and Participation Action Record** to record progress.

2. School Visit or Vocation Visit by Fieldworker

Goal = Admission to School

↓

School Visit

☐ Complete **School Visit 4 Checklist**

Purpose: Provide schoolteacher recommendations to facilitate the child's learning and set admittance date

Person responsible: Teacher and Fieldworker

Use the **School Environment and Participation Action Record** to record progress.

Goal = Vocational Skills Training

↓

Vocation Visit

☐ Complete **Vocation Visit 1 Checklist**

Purpose: Child to commence their vocation

Person responsible: Fieldworker

Use the **Vocation Environment and Participation Action Record** to record progress.

MONTH 5:

1. Home Visit by Fieldworker

☐ Continue home modifications and assistive devices. Modifications and assistive devices need to be completed over the 6 months. Use the **Home and Community Environment and Participation Action Record** to record progress.

☐ Continue home participation training. Strategies need to be implemented over the 6-month transition program. Use the **Home and Community Environment and Participation Action Record** to record progress.

2. School Visit or Vocation Visit by Fieldworker

Goal = Admission to School

↓

School Visit

☐ Complete **School Visit 5 Checklist**

Purpose: Check-in following child's admission to school

Person responsible: Fieldworker

Use the **School Environment and Participation Action Record** to record progress.

Goal = Vocational Skills Training

↓

Vocation Visit

☐ Complete **Vocation Visit 2 Checklist**

Purpose: Maximise the child's participation in their vocational activity

Person responsible: Fieldworker

Use the **Vocation Environment and Participation Action Record** to record progress.

MONTH 6:

1. Home Visit by Fieldworker

☐ Continue home modifications and assistive devices. Modifications and assistive devices need to be completed over the 6 months. Use the **Home Environment and Participation Action Record** to record progress.

☐ Continue home participation training. Strategies need to be implemented over the 6-month transition program. Use the **Home and Community Environment and Participation Action Record** to record progress.

2. School Visit or Vocation Visit by Fieldworker

Goal = Admission to School

↓

School Visit

☐ Complete **School Visit 6 Checklist**

Purpose: Maximise child's participation in school activities

Person responsible: Fieldworker

Use the **School Environment and Participation Action Record** to record progress.

Goal = Vocational Skills Training

↓

Vocation Visit

☐ Complete **Vocation Visit 3 Checklist**

Purpose: Maximise child's participation in their vocational activity

Person responsible: Fieldworker

Use the **Vocation Environment and Participation Action Record** to record progress.

MONTH 7:

1. Home Visit by Fieldworker

☐ Complete home modifications and assistive devices. Provide recommendations. Use the **Home and Community Environment and Participation Action Record** to record progress.

☐ Complete home participation training. Provide recommendations. Use the **Home and Community Environment and Participation Action Record** to record progress.

2. School Visit or Vocation Visit by Fieldworker

Goal = Admission to School

↓

School Visit

☐ Complete **School Visit 7 Checklist**

Purpose: Ensure that strategies are implemented, required assistive devices are in place, and discharge from Shishu Shorgo transition program

Person responsible: Fieldworker

Use the **School Environment and Participation Action Record** to record progress.

Goal = Vocational Skills Training

↓

Vocation Visit

☐ Complete **Vocation Visit 4 Checklist**

Purpose: Ensure that strategies are implemented, required assistive devices are in place, and discharge from Shishu Shorgo transition program

Person responsible: Fieldworker

Use the **Vocation Environment and Participation Action Record** to record progress.

Community Follow-up

Home Environment Checklist

Name of Child: ______________________________ Date conducted: ________________

Functional Mobility:

Can the child move independently from one place to the other, even a little way? ☐ Yes ☐ No

How does the child move (e.g. rolling, dragging body in semi sitting, crawling….)?

____

How far can they move independently? ____

Does the child need assistive devices to make moving easier?

- ☐ Orthotics
- ☐ Walking frame
- ☐ Wheelchair
- ☐ Other: ____

Can the child enter and exit their home independently? ☐ Yes ☐ No

Are there any steps making moving difficult? ☐ Yes ☐ No, it is level

Toileting

Can the child use the toilet independently? ☐ Yes ☐ No

Does the child have a way to sit safely and independently on the toilet? ☐ Yes ☐ No

Does the child have a way to sit down and stand up from the toilet safely and independently?

☐ Yes ☐ No

Does the child need equipment or rails to use the toilet more independently? ☐ Yes ☐ No

Bathing

Can the child bathe independently? ☐ Yes ☐ No

Where do they bathe, what does it look like? ____

____

Does the child sit, squat or stand to bathe? ☐ Yes ☐ No

Does the child need a special seat to make bathing easier? ☐ Yes ☐ No

Does the child need rails to help them move safely in the bathing area? ☐ Yes ☐ No

Eating:

Can the child eat independently? ☐ Yes ☐ No

Can the child hold food in their hand? ☐ Yes ☐ No

Can the child bring their hand to their mouth? ☐ Yes ☐ No

Does the child need assistive devices to help them eat independently? ☐ Yes ☐ No

Play:

Who does the child play with? ____

Where do they play? ____

What do they play? ____

Does the child need equipment, assistive devices or special toys to help them play?

☐ Yes ☐ No

Important note: When working with local carpenters to make assistive devices, try and work with the same one so that they also learn with experience.

Box 1:

Does the child need assistive devices to make moving easier?

☒ Orthotics

☒ Walking frame

☒ Wheelchair

☒ Other: ______walking_stick____

- If you ticked orthotics, refer the child to CRP for fitting of orthotics. They have trained staff who custom make the orthotics.
- If you ticked walking frame, wheelchair or other: refer the child to CRP. They have trained staff who take the child's measurements and a workshop that makes the assistive devices. ALTERNATIVELY: Use the Bangla translation of 'Disabled Village Children' as a guide, and talk to a local carpenter about designing and making the assistive devices.

Box 2:

Are there any steps making moving difficult? ☒ Yes ☐ No, it is level

- If the child can walk, but is unsteady, organise for a handrail to be installed beside the doorway so that they can hold onto this while they step up and down. Install the rails at the position where the child is reaching, so that it is at the right height.
- If the child cannot walk, talk to the family about having a ramp installed. You could use concrete or timber. Make sure the ramp is not too steep and is not slippery when wet. Also install handrails / side barriers to prevent falling off.

Box 3:

Does the child need equipment or rails to use the toilet more independently? ☒ Yes ☐ No

- If the child can walk, but is unsteady, organise for rails to be installed beside the toilet. Install the rails at the position where the child is reaching, so that it is at the right height.
- If the child cannot walk, organise a special seat to use over the squat toilet. You can get this from CRP. ALTERNATIVELY, use the Bangla translation of 'Disabled Village Children' as a guide, and talk to a local carpenter about designing and making a special seat.

Box 4:

Does the child need a special seat to make bathing easier?	☒ Yes	☐ No

- Maybe you ticked yes because the child is able to walk, but has low endurance or the child is not able to stand at all.
- If the child can sit up independently, get a plastic children's chair to use in the shower area.
- If the child cannot sit up independently, use a waterproof corner chair with straps. You could buy this from CRP or talk to a local carpenter and make it. Make sure the corner chair has some padding so that it is not hard and uncomfortable for the child, and make sure the straps do not hurt the child. Make a chair that supports the child's body well so that they can use their hands!

Box 5:

Does the child need rails to help them move in the bathing area?	☒ Yes	☐ No

- Maybe a child need rails to help them stand up in the shower area, and move in and out of the shower area.
- Have rails installed where they are reaching, so that they are at the right height. Think about whether the rail is best positioned horizontal or vertical.

Box 6:

Does the child need assistive devices to help them eat independently?	☒ Yes	☐ No

- Maybe the child needs a device with a big or long handle, or one that is strapped to their hand to help them scoop food and bring it to their mouth. Maybe they need a special cup to drink from.
- Look in the Bangla translation of 'Disabled Village Children' about ideas of different assistive devices and how to make them.

Box 7:

Does the child need equipment, assistive devices or special toys to help them play?	☒ Yes	☐ No

- Maybe the child needs a special chair to help them sit and play, or a wheelchair to help them move and play. If this is the case, send a referral to CRP to have assistive devices made for them, or talk to a local carpenter about making it.
- Maybe the child has difficulty holding onto toys, or maybe the family do not know how to enable their child to play. If this is the case, look in the Bangla translation of 'Disabled Village Children' about ideas of different toys and how to make them. Demonstrate to the parents how they can interact and play with their child and enable their child to play with toys.

Community Follow-up

Home and Community Participation Checklist

Name of Child: ____________________________________ Date conducted: ____________________

Who are involved: Teacher and Fieldworker meet with family at the child's home.

Educate the family about the child's rights and potential. Discuss and decide the child's transition goal (use the flowchart in the Community Follow-up Checklist as a guide):

☐ Participation in school

☐ Vocational skills training

Show the family the already completed Initial Assessment form. Talk about the activities that have crosses saying that the child cannot do this. Explain that this session is about looking into what the child can do now, and identifying ways to increase their child's participation at home and in their community.

Communicating:

Can the child communicate with all family members? ☐ Yes ☐ No

How does the child communicate? (Tick all that apply) ☐ Verbally ☐ Sign language/ gesture

☐ Facial expressions ☐ Other: ____

Can the child communicate…

What they need and want? ☐ Yes, how? ____

☐ No

How they are feeling? ☐ Yes, how? ____

☐ No

What is the child's communication goal? ____

____

Helping at Home:

Does the child have a way to help at home in at least one age appropriate task?

☐ Yes, what do they do? ____

____

☐ No, what could they do? ____

____

What is the child's helping goal? ____

____

Participating in the Community:

Does the child interact and spend time with children in their community?

☐ Yes, how? ____

____

☐ No, why? ____

____

What is the child's goal to increase community participation? ______________________

____

Does the child participate in opportunities to gain work exposure/experience in their community?

☐ Yes, what opportunities is the child provided and how do they participate? ______________________________

____

____

☐ No, what opportunities exist for the child to gain work exposure/experience in their community?

____

____

What is the child's goal to increase work exposure/experience in their community? ____________________

____

Communicating:
The child's communication goal could be to (in order of least difficult to most difficult): Make choices, communicate words, sentences or stories. Maybe the child's goal is to be understood by others.

- If the goal is to make choices: Use a combination of simple sign language (e.g. more, finished, go, stop) with two giving two options. These could be two different toys/objects or picture cards. Recognise pointing, vocalisations and looking at a card, a person, or an object as making a choice. Make sure the child then experiences the effect of the choice they just made by looking/pointing/vocalisation. E.g. if the child was given a choice between a red or green pencil and they looked at the red pencil, then draw with the red pencil.

- If the goal is to be understood by others: This could be because the child is unable to speak, their speech is unclear or speaking is difficult for them. Teach the child other ways of communicating to support their verbal communication. This includes sign language, picture cards and communication boards. Grade what you teach to match the child's skill level.

- If the goal is to communicate words, sentences or stories: Give the child opportunities to learn and develop their communication using the variety of communication methods. Communication methods include verbal, written, sign language and communication boards.

- Responsibilities of the Teacher: Provide the family communication resources to use at home. This includes teaching the parents some sign language, and making each child a communication board. The teacher also needs to provide heaps opportunities for the child to develop their communication in the group therapy program.

- Responsibilities of the Fieldworker: Practice communication methods taught at Shishu Shorgo with the child and family in their home. This needs to be applied to the home context, and practiced/used during bathing, toileting, eating and drinking, playing and helping.

Helping at Home:
The child's goal could be to help put away (clean up), or to help with sorting fruit and vegetables after shopping and before cooking. Make the goal achievable for the child. E.g. if the child cannot walk, then make the goal for the child to help do a task sitting.

- Work out what makes this task difficult for the child. Does the child have difficult sitting? Then make sure the area is set up to help keep the child upright and free to use their hands.
- Adapt the environment so that it supports the child's participation. Make sure that the task is challenging, but achievable for the child.
- You may need to start by taking turns at doing the task, as one try may be very difficult for the child to do physically/understand.
- Educate the child's family about their child's potential and right to participate. Explain that children learn through experience, and helping at home will also help them learn.

- Responsibilities of the Teacher: Provide the child opportunities for the child to practice the goal task at Shishu Shorgo. E.g. if their goal is to help put away, the child needs to help put away after a class activity.

- Responsibilities of the Fieldworker: Practice this goal task with the child and family at home. Setup the environment to enable success for the child.

Participating in the Community:

- Educate the community about the rights of people with disabilities, their potential and how they can contribute to their community.
- Support the community to provide the child with opportunities to participate in their community. The community need to cater for the child's physical needs such as by providing a helper, and having an accessible environment for the child (with wheelchair access or special seating).
- Find out the child's interests. What activity would the child like to participate in to contribute to their community? Depending on the child's age and interests, this could be through play or work.
- Identify opportunities for the child to gain work exposure/experience in their community. This will help all children identify and transition to vocational activities when they are of an appropriate age. This will also help community members recognise the potential of children with disabilities and how they can contribute in a workplace.

  Work exposure/experience involves 'interning' with a community member at their work place by following and helping them in small ways. For example, a child may intern with a tea walla by sitting behind the teashop counter every day for an hour and helping wash cups/stack plates/pass money/interact with customers.

- <u>Responsibilities of the Teacher:</u> Provide the child opportunities to play and interact with other children attending Shishu Shorgo (they are also members of the community!). If the child has a helping goal, provide the child opportunities to practice the task at Shishu Shorgo. E.g. if their goal is to help wash plates in water, the child needs to practice washing objects in water – they could help clean up after nasta break.

- <u>Responsibilities of the Fieldworker:</u> Practice the goals tasks with the child and family in their home and community. Identify opportunities for the child to participate in activities with other children and gain work exposure/experience in their community. Setup the environment to enable success for the child. Advocate for children with disabilities in the community – run awareness campaigns/activities to bring the community together and experience the potential of people with disabilities and how they can contribute.

Home and Community Environment and Participation Action Record

Action	Date Started	Date Completed

Community Follow-up

School Participation Checklist

Month 1: School Visit 1 Checklist

Name of Child: ___________________________________ Date conducted: _____________________

Purpose: Introductions and goal setting.

Who are involved: Shishu Shorgo teacher and fieldworker meet with the schoolteacher, child and caregiver at the child's local school.

Educate the schoolteacher about the child's rights and potential. Explain to the schoolteacher the child's goals for the transition program at Shishu Shorgo. Explain the skills that the child has now and the skills that will be developed through the program and how they will assist the child participate at school. Explain the child's potential for school participation, and also explain how barriers to their participation are mainly external (environmental, social). The Shishu Shorgo teacher and fieldworker will provide support to the school to enable the child's admission and participation in school. Provide the schoolteacher contact details of Shishu Shorgo's teacher and fieldworker.

Discuss and decide, what is the child's goal for school participation?

- ☐ Graded entry to school (start with meal times, then morning classes, then whole day)
- ☐ Morning classes
- ☐ Afternoon classes
- ☐ Meal and play times
- ☐ Whole day

Set date for next school visit

Date of School Visit 2: ______________________________

Month 2: School Visit 2 Checklist

Name of Child: ___________________________________ Date conducted: _____________________

Purpose: Prepare school environment for child's admission.

Who are involved: Fieldworker, schoolteacher and caregiver meet at child's local school.

Discuss and decide based on the child's physical and intellectual abilities:

Where will the child's classroom be? (Consider will they have to go up/down stairs to access it; how will they access the toilet, meal area and play area?)

____

____

Where will the child sit in the classroom? (Consider will they be able to see the board from where they are sitting; do they have difficulty maintaining attention to task – if yes where will they be less distracted e.g. not facing windows, close to the teacher; will they need regular assistance from the teacher?)

____

____

What does the child's school table and chair look like? Do any changes need to be made so that they can sit on the chair independently, or sit in their wheelchair at the table? (Do they need a supportive seat? Do they need a tilt board for their desk?) **Organise changes to be made.**

____

____

Do any changes need to be made to the toilet so that the child can use it? (Do they need a special seat or rails? Will they need someone to assist? If yes, who will help?) **Organise changes to be made.**

____

____

Can the child access their classroom, toilet, meal area, play area? Does anything need to be changed to help? (e.g. rails, ramps, do they need a special seat in the meal area? Will they need someone to assist with meals? If yes, who will help?) **Organise changes to be made.**

____

____

Are there any other concerns that need to be discussed? (Fieldworker to raise additional concerns, and also ask teacher, caregiver if they have concerns)

____

____

Plan for School Visit 3; introducing child to school. Decide:

What date will visit 3 occur? ______________________________

What session will they attend? (Decide based on child's school participation goal)

- ☐ Morning class
- ☐ Meal break
- ☐ Morning class plus meal break

Will they need assistance?

- ☐ Yes, Who will help? ______________________________
- ☐ No

Explain to the schoolteacher what will happen during school visit 3.

Month 3: School Visit 3 Checklist

Name of Child: ___________________________________ Date conducted: _____________________

Purpose: Introduce child to school.

Who are involved: Child and fieldworker meet with child's future schoolteacher and class.

Fieldworker and Schoolteacher must:

- ☐ Introduce child to class, and have students introduce themselves to child. E.g. the fieldworker and schoolteacher could lead an activity where everyone says their name and something that they like.
- ☐ Child is shown their seat and table. Child sits at their table for a session. Fieldworker helps the child participate in class activities.
- ☐ Fieldworker introduces schoolteacher and student's the child's method of communication/mobility and demonstrates positive interaction. Fieldworker does this by playing an inclusive game or activity.
- ☐ Child shares a meal break with peers. Fieldworker provides support and facilitates inclusive mealtime and play.
- ☐ Child tries toilet facilities with modifications/assistive devices in place.

Following sessions, fieldworker, schoolteacher and parent meet and discuss the outcome of the child's school visit. Discuss the following:

Did the child enjoy the experience? Why?

____

____

Are there any changes that need to be made to help facilitate the child's participation and inclusion? Think about the classroom setup, toilet setup, and access. **Organise changes to be made.**

____

____

What further support does the Schoolteacher think they need to help provide inclusive education?

____

____

The Fieldworker relays this information back to the Shishu Shorgo teacher. Together the fieldworker and teacher plan for School Visit 4 during which the Shishu Shorgo teacher provides the Schoolteacher information about the child's abilities, developing skills, and learning styles.

Month 4: School Visit 4 Checklist

Name of Child: ______________________________ Date conducted: __________________

Purpose: Provide schoolteacher recommendations to facilitate the child's learning, and set admittance date.

Who are involved: Shishu Shorgo teacher, fieldworker, schoolteacher and caregiver.

Shishu Shorgo teacher to provide schoolteacher and discuss:

- ☐ Child's Assessment and Goal Setting
- ☐ Child's monthly progress records
- ☐ Recommendations for increased participation at school

Sihsu Shorgo teacher and Fieldworker explain to Schoolteacher and Caregiver how CSF can provide support for the next 2 months for the child's admission and participation in school.

Set admittance date:

______________________________ (name of child) will start school on the ___________________ (date)

Decide on the child's attendance:

- ☐ Graded entry to school (start with meal times, then morning classes, then whole day)
- ☐ Morning classes
- ☐ Afternoon classes
- ☐ Meal and playtime
- ☐ Whole day

Month 5: School Visit 5 Checklist

Name of Child: ________________________ Date conducted: ________________

Purpose: Check-in following child's admission to school.

Who are involved: Fieldworker, schoolteacher and child.

Meet with schoolteacher before class. Ask the Schoolteacher, are they experiencing any issues limiting the child's participation in school?

____

____

Fieldworker observes child in class and during meal break. Do you see any issues limiting the child's participation in school?

____

____

Fieldworker's list of recommendations to provide to schoolteacher to increase the child's opportunities to participate in school:

____

____

____

____

____

Explain and provide a written copy of recommendations to schoolteacher based on observations.

Help implement recommendations.

Month 6: School Visit 6 Checklist

Name of Child: ___________________________________ Date conducted: _____________________

Purpose: Maximise child's participation in school activities.

Who are involved: Fieldworker, schoolteacher and child.

Meet with schoolteacher before class. Ask the Schoolteacher, are they experiencing any issues limiting the child's participation in school?

____

____

Fieldworker observes child in class and during meal break. Do you see any issues limiting the child's participation in school?

____

____

Fieldworker's list of recommendations to provide to schoolteacher to increase the child's opportunities to participate in school:

____

____

____

____

____

Explain and provide a written copy of recommendations to schoolteacher based on observations.

Help implement recommendations.

Month 7: School Visit 7 Checklist

Name of Child: ______________________________ Date conducted: __________________

Purpose: Ensure that strategies are implemented, required assistive devices are in place, and discharge from Shishu Shorgo transition program.

Who are involved: Fieldworker, schoolteacher, child and caregiver.

Meet with schoolteacher before class. Ask the Schoolteacher, are they experiencing any issues limiting the child's participation in school?

____

____

Fieldworker observes child in class and during meal break. Do you see any issues limiting the child's participation in school?

____

____

Fieldworker's list of recommendations to provide to schoolteacher to increase the child's opportunities to participate in school:

____

____

Explain and provide a written copy of recommendations to schoolteacher based on observations. **Help implement recommendations.**

Discuss discharge from Shishu Shorgo program. Explain that this means the fieldworker will stop making regular, monthly visits. However, if any issues arise in the future regarding the child's participation/health, the schoolteacher/caregiver can contact CSF and the fieldworker will provide support in a one-off session. The fieldworker can phase out support by checking-in with the family and school once in three months, twice a year, once a year etc.

School Environment and Participation Action Record

Action	Date Started	Date Completed

Community Follow-up

Vocation Checklists

Month 1: Identify transition goal

1. Decide child's transition goal using the flowchart in the community follow-up checklist:

Is the child under 14 years old?

Yes

No, the child over 14 years old

Is the child and family's goal for admission to school?

Is the child capable of attending a class appropriate for their age group?

Yes

No

Goal = Participation in School

Goal = Vocational Skills Training

2. For all children, the fieldworker will identify and facilitate participation in opportunities for the child to gain work exposure/experience in their community, using the **Home and Community Participation Checklist**. This will help all children identify and transition to vocational activities when they are of an appropriate age. This will also help community members recognise the potential of children with disabilities and how they can contribute in a workplace.

   Work exposure/experience involves 'interning' with a community member at their work place by following and helping them in small ways. For example, a child may intern with a tea walla by sitting behind the teashop counter every day for an hour and helping wash cups/stack plates/pass money/interact with customers.

3. If the child's transition goal involves Vocation Skills Training, continue to Month 2: **Vocation Planning 1 Checklist**.

Month 2: Vocation Planning 1 Checklist

Name of Child: ______________________________ Date conducted: __________________

Purpose: To identify a suitable vocation for the child.

Who are involved: Field worker, child and caregivers.

1. Ask and discuss the answer to each question. The questions are designed to generate possible ideas for the child's vocation.

What are common jobs in your community? (E.g. weaving, agriculture, tea stall)

____

____

What could the child do that would be helpful to their family and community? (E.g. Children could go to the market with the elderly ladies and help them carry home wholesale items that they then either resold or distributed in the community. Children could collect shopping lists from family or other community members and purchase and deliver items for a fee. Children could mix floor cleaning fluids and dishwashing soap to sell).

____

____

What raw materials are available in your community? (E.g. you could create a new occupation for the child that involves weaving the leaves from the palm tree, or by growing medicinal herbs in a fertile unused patch of land).

____

____

Who has time to help the child learn and assist them to participate in a vocation? What are their skills? (Think beyond the immediate family and consider others in the community. Consider also people such as the schoolteacher, or government worker posted nearby who may consider supporting the child and family with their knowledge).

____

____

What does the child enjoy? Do they enjoy company and interacting with others? (If they don't enjoy interacting with others, think of a more solitary job such as packing. If they do enjoy interacting with others, think of a job where they are regularly interacting such as collecting orders and selling items).

____

____

What are barriers to the child performing the vocation? How can these barriers be overcome? (E.g. provide assistive devices, assistance to setup and pack up).

____

____

Which vocation seems most suitable for the child? (One that the child will enjoy, that they have access to required materials and supervision/assistance from a caregiver).

____

____

2. **Fieldworker to talk to community members involved in the child's vocation and organise a meeting for next month.** E.g. if the child is to help wash cups at a tea stall, the Fieldworker must talk to tea wallas and arrange a meeting with them, the child, caregiver and fieldworker during Month 3.

Month 3: Vocation Planning 2 Checklist

Name of Child: ________________________________ Date conducted: ____________________

Purpose: Plan for how the child will participate in their vocation.

Who are involved: Fieldworker, child, caregivers, and other community members involved in vocation e.g. if the child is to help wash cups at a tea stall, then involve the tea walla.

1. Hold a meeting to discuss and decide the following:

Describe the child's vocational activity. Will they sit, stand, move around? How will they need to use their hands? What will they need to remember? What will they need to communicate?

____

____

What days of the week will the child do their vocation?

____

____

What time will they start their vocation?

____

____

How long will they do their vocational activity? (E.g. one hour, two hours)

____

____

Where will they do their vocation? (E.g. outside their home, at a tea stall, at a hand loom)

____

____

How will they travel to and from their vocation?

____

____

What assistive devices will they need to do their vocation? (E.g. do they need a floor mat, special chair, big handles, picture instructions)

____

____

Who will assist/supervise them while they do their vocation? What assistance/supervision will they provide?

____

____

When will the child start?

____

____

Month 4: Vocation Visit 1 Checklist

Name of Child: ___________________________________ Date conducted: ______________________

Purpose: Child to commence their vocation.

Who are present: Fieldworker, child, and caregiver and community member involved in providing the child assistance/supervision during their vocation time.

Fieldworker to:

1. Assist the community member and caregiver to setup the vocation environment (i.e. with required assistive devices such as mats, special chairs). Describe what you did:

   ____

   ____

   ____

   ____

2. Demonstrate the assistance/supervision the child needs to participate. Describe what you did:

   ____

   ____

   ____

   ____

3. Provide recommendations:

   ____

   ____

   ____

   ____

Month 5: Vocation Visit 2 Checklist

Name of Child: ___________________________________ Date conducted: _____________________

Purpose: Maximise child's participation in their vocational activity.

Who are involved: Fieldworker to visit the child during their vocation time. Caregiver and community member involved in providing the child assistance/supervision, to also be present.

1. Ask the caregivers and community members involved in assisting/supervising the child the following questions:

What can the child do well?

____

____

____

____

What are the barriers to their participation?

____

____

____

____

What support would you like from Shishu Shorgo to help maximise the child's participation in their vocation?

____

____

____

____

2. Fieldworker to observe the child and review the following:

Skills	What parts of the vocational activity can the child do?	What parts of the vocational activity does the child have difficulty doing?	What would make the vocational activity easier for the child?
Communicating (Verbal and AAC)			
Arm and hand control			
Sitting			
Mobility			
Cognition (Memory, sequencing)			

3. Provide recommendations (based on observations and feedback from caregiver and community members):

____

____

____

____

____

____

Month 6: Vocation Visit 3 Checklist

Name of Child: ______________________________ Date conducted: __________________

Purpose: Maximise child's participation in their vocational activity.

Who are involved: Fieldworker to visit the child during their Vocation time. Caregiver and community member involved in providing the child assistance/supervision, to also be present.

1. Ask the caregivers and community members involved in assisting/supervising the child the following questions:

What can the child do well?

____

____

____

____

What are the barriers to their participation?

____

____

____

____

What support would you like from Shishu Shorgo to help maximise the child's participation in their vocation?

____

____

____

____

2. Fieldworker to observe the child and review the following:

Skills	What parts of the vocational activity can the child do?	What parts of the vocational activity does the child have difficulty doing?	What would make the vocational activity easier for the child?
Communicating (Verbal and AAC)			
Arm and hand control			
Sitting			
Mobility			
Cognition (Memory, sequencing)			

3. Provide recommendations (based on observations and feedback from caregiver and community members):

____

____

____

____

____

____

Month 7: Vocation Visit 5 Checklist

Name of Child: ______________________________ Date conducted: __________________

Purpose: Ensure that strategies are implemented, required assistive devices are in place, and discharge from Shishu Shorgo transition program.

Who are involved: Fieldworker to visit the child during their Vocation time. Caregiver and community member involved in providing the child assistance/supervision, to also be present.

1. Ask the caregivers and community members involved in assisting/supervising the child the following questions:

What can the child do well?

____

____

____

____

What are the barriers to their participation?

____

____

____

____

What support would you like from Shishu Shorgo to help maximise the child's participation in their vocation?

____

____

____

____

2. Fieldworker to observe the child and review the following:

Skills	What parts of the vocational activity can the child do?	What parts of the vocational activity does the child have difficulty doing?	What would make the vocational activity easier for the child?
Communicating (Verbal and AAC)			
Arm and hand control			
Sitting			
Mobility			
Cognition (Memory, sequencing)			

3. Provide recommendations (based on observations and feedback from caregiver and community members):

____

____

____

____

____

____

4. **Discuss discharge from Shishu Shorgo program.** Explain that this means the fieldworker will stop making monthly visits. However, if any issues arise in the future regarding the child's participation/health, the community member or caregiver can contact CSF and the fieldworker will provide support in a one-off session. The fieldworker can phase out support by checking-in with the family once in three months, twice a year, once a year etc.

Vocation Environment and Participation Action Record

Action	Date Started	Date Completed

APPENDIX:

Communication Resources

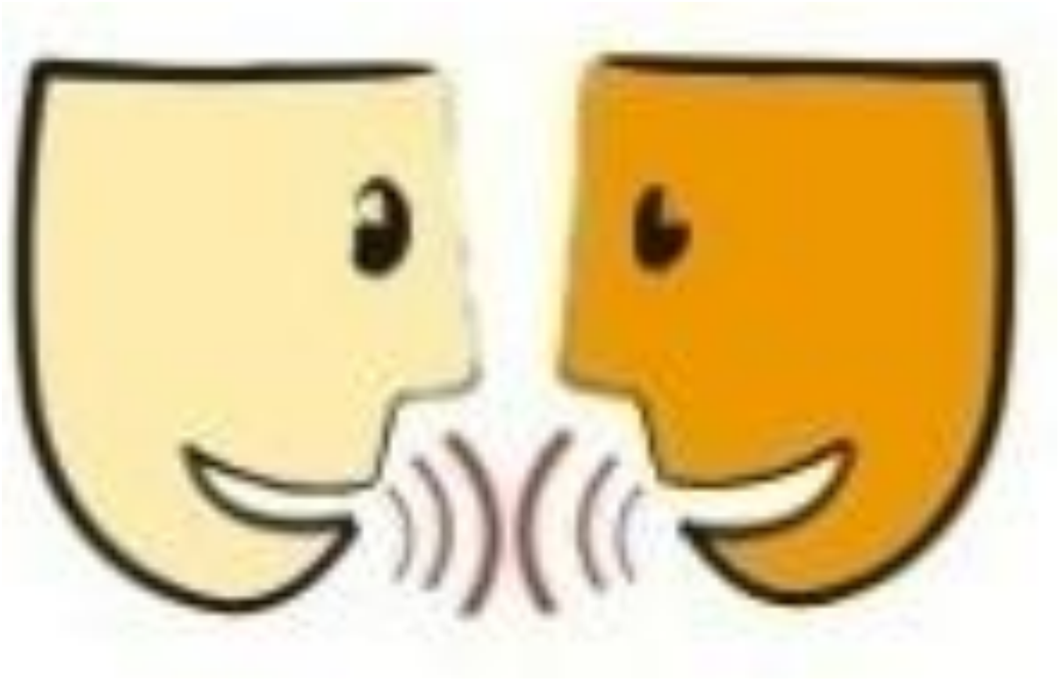

Augmentative and Alternative Communication (AAC) Fact Sheet

What is Augmentative Alternative Communication?

Augmentative and alternative communication (AAC) includes all other forms of communication than verbal speech that are used to express thoughts, needs, wants and ideas.

People with difficulty communicating verbally rely on AAC to be understood by others. AAC can also be used to help the person with a disability understand what others are saying and asking. AAC is not intended to replace speech but to assist in the understanding of speech.

Types of Augmentative Alternative Communication:

- Pointing, gestures and body language
- Signing
- Object symbols
- Chat books
- Communication boards
- Spelling
- Speech generation devices

When teaching a child how to use AAC effectively in their everyday lives, ensure the child receives plenty of opportunities to **practice.**

Pointing, Gestures and Body Language

Pointing, gestures and body language are non-verbal methods of communication that are used by all people to help communicate a message. They are a useful means of communication for children who have difficulty speaking as most people understand what pointing, gestures and body language mean.

Gestures are developed as a part of culture. Thumbs-up may mean "yes" or "good". Shrugging shoulders may mean, "I don't know" or "maybe".

Body language can communicate different emotions such as excited, happy, sad, angry.

Pointing is used to single out something seen, such as an activity, a person, an animal or object. It can be used to bring someone else's attention to it, or make a choice. Children with physical disabilities often have difficulty pointing with their hands. For these children other methods may be used, such as:

- Eye-pointing i.e. looking at things
- Full body movements to show agreement or rejection of what has been said. For example, a child might stiffen their body and smile for yes and turn away to reject or say no.

Pointing, gestures and body language should be encouraged as a form of communication for all children who have difficulty speaking.

Signing

Signing is a visual and gestural communication system used with or without speech between people who know signs. Signing can be used to help children understand speech as the sign can be held up to look at whereas speech 'disappears' as it is spoken. Signing also assists children with difficulty speaking verbally to be understood by others. A collection of signs are often formalised in different cultures as sign language.

To obtain Bangla sign language resources, refer to the Bangla translation of 'Disabled Village Children' by David Werner, and contact the Centre for Disability in Development (CDD) for copies of their resources.

Key Word Signing

Key Word Signing is a method of signing that facilitates both communication and cognitive development. In this method, only Key words are signed. Key words are the important words that convey meaning. For example, when conveying the message "I would like to eat a banana", the words: I, banana and eat are signed.

The same sign is used to mean similar objects, for example the same sign could be used for chair, seat, lounge and bench. Speech is always used together with the signs. Gestures, facial expressions, body language and intonation are used to assist conveying the meaning.

Important things to remember when Key Word Signing:

- Always speak as you sign and use facial expression
- Only sign key words (words that convey meaning)
- Use movement to demonstrate concepts, such as big compared to little
- Use the same hand for signing
- Create a reason for the person to sign, for example, place items slightly out of reach so that they have to ask for them
- Practice using functional signs in everyday situations e.g. go, finish, more, toilet, eat, drink
- Both the child as well as their communication partner should sign because signing helps to improve the child's understanding of speech

Key Word Signing should be encouraged as a form of communication for all children who have difficulty speaking.

Object Symbols

Object symbols are tangible objects that represent an activity, object or person. Object symbols can be:

- Miniature: A miniature version of the real object. For example, a small plastic bus to represent travelling by bus.
- Partial: A small part of a real object. For example, one puzzle piece to represent a puzzle.
- Associated: An object that could be associated with the real object or activity. For example, a spoon could represent eating food.
- Symbolic: An object not directly related to the real thing, requiring the child to think more about the object. For example, a small plastic banana to represent snack time.

Object symbols can be used to help the child:

- Understand what activities they are going to do that day, what is going to happen next
- Understand different options so that they can then choose what comes next

- Communicate what they want
- Understand the symbols on a communication board, in a chat book or in a calendar/schedule.

Important things to remember when using object symbols:

- Object symbols can be used to represent activities that the child does regularly during their day or week.
- Choose object symbols especially for that child so that they will be relevant to them.
- When teaching the child how to use object symbols for communication, choose a few of the most important symbols first.
- Always use the same object symbol so that the child learns the association between the symbol and the meaning.
- Match the symbol consistently with the activity it is representing. For example, every time the child has a meal, help them to feel, look at, smell and listen to the object symbol for mealtime before they have their meal.

Object symbols should be encouraged as a form of communication for children who have difficulty speaking and having difficulty understanding simple signs and communication boards.

Chat Books

Chat books are small 'All About Me' books that contain photos, pictures, symbols, words and messages about the child. They provide important, meaningful information about the child such as:

- Their family members, friends, teachers
- Favourite toys, colours, foods,
- Special occasions like birthdays
- Messages about what the child has done that day. It could include samples of their work.

The chat book provides greater opportunities for conservation between the child with a disability and their communication partner (e.g. teacher, friend, parent).

Important things to remember when using a chat book:

- Make interactions fun and casual
- Use the book when the child shows that they are interested, for example when the child reaches for their chat book and is looking at the pictures/words in their chat book
- Talk about the pictures, symbols, and words in the book as though you are reading a story together
- Encourage the child to say, sign or point to content on the pages that are being talked about
- Make the book available to the child throughout the day so that they can start interactions by opening the book or looking at it
- Ask questions that may be answered by using the book, for example, "What did you do at school today?"
- Prompt the child to use the book by asking appropriate questions, for example, "What did you do today? Let's look at your book to find out".
- Keep the book up-to-date. Add things to the chat book to represent activities done that day, so that it can facilitate conversation between child-parent, child-teacher etc.

Chat books should be should be encouraged as a form of communication for all children who have difficulty speaking.

Communication boards

Communication boards comprise a number of boxes on a piece of paper that each contains a word and corresponding picture/symbol. Communication boards can be made as one single sheet of paper, a booklet of different communication board pages or in a wallet. Using a communication board involves the child pointing (with their hand or eye) to a picture/word box on a page. It also involves the communication partner to look at the communication board, identify the child's choice and read that choice aloud.

Refer to **Example Communication Boards** for examples of communication boards that you can adapt for the child you're communicating with.

Important things to remember when using a communication board:

- Always have the word and corresponding picture in a box
- The pictures describing the word can be a photo (e.g. of the child doing the activity, or of the child's wheelchair), or a symbol (e.g. a clip-art wheelchair, or a banana to represent snack time)
- Read aloud the child's choice
- Provide plenty of opportunities to practice

Activity ideas to help a child learn how to use a communication board:

- Start with 2 boxes on one page and get the child to make a choice from these boxes. Once the child has grasped the meaning of two boxes, then introduce more boxes. Remember, the more boxes there are on the page, the more difficult it is for the child (you need to grade the task for each child).

- Use object symbols on top of the picture/word box to help the child understand it's meaning.

- Make two copies of the communication board boxes and cut them out. Play matching games. Give the cards upside down or on one side to see if the child is able to correct and identify the match with ease.

    - If the child has perceptual problems, they may need lots of real experience with their own face and a mirror. Play games with the faces of their friends, teachers or parents and drawing or making faces using cut out eyes, eyebrows and mouth to match emotions. Children need to grasp this concept before learning to use a communication board.

- Use the **'I Feel' activity pages**. Print out two copies of the biggest size sheet. Cut one into individual cards. Give the child the sheet with the biggest of the three sizes. The teacher than hands the child one of the face cards and asks the child to find the same in the sheet. Watch for ease of matching and wether the child has a systematic scanning strategy.

    - If the child is struggling with scanning, you may need to give markers such as a line the child can follow or change the arrangement of pictures.

    - If the child is struggling to match, verbally ask them to look at different details (e.g. is the mouth turned up or down, what shape are the eyebrows making) and see if the child is having difficulty seeing detail.
      If the child is having difficulty seeing detail, you may need larger or more simplified pictures.

- Use the 'I Feel' activity pages. Print out two copies of each sheet and cut out the boxes. Play matching games with the different sized picture/word cards to see what size is most effective.
    - If the child has difficulty with scanning, cut the chart into strips and increase distance between the rows or change the arrangement.

Once the child is able to match the emotion with the faces of the children shown without any guidance hints or prompting from the adult, (unknown faces and no context - can only match if they are able to see and comprehend the emotion expressed), you know they have the capacity to understand and use these symbols.

Communication boards should be should be encouraged as a form of communication for all children who have difficulty speaking.

Spelling

Spelling is the most familiar way of communicating a message other than speech, it lets people communicate anything they want, and is a very effective method of communicating. Spelling can involve:

- The child writing a message on a piece of paper for the other person to read.
- The child pointing (with finger or eyes) to letters on a laminated letter chart to spell out their message. Refer to the **ABC Alphabet Board** as an example of a letter chart designed for communication.
- The adult reading out the letters and the child signalling when they have the correct letter e.g. by tapping the table.

The spelling method can present problems because:

- Many children with disabilities have difficulty learning how to spell
- It is a slow way of putting together messages

Spelling should be encouraged as a form of communication for children who are able to recognise letters and know how to read and spell some words (communicating using spelling also helps the child develop their reading and writing skills).

Speech Generation Devices

Speech generation devices use technology to generate speech by selecting a series of symbols/pictures and words. To find out what speech generated devices are available in Bangladesh, contact local Disability organisations who make/import speech generated devices for information. Speech generated devices are usually expensive and require intensive training for the child, family and teachers to learn how to use the device effectively.

Picture cards for Group Therapy Activities

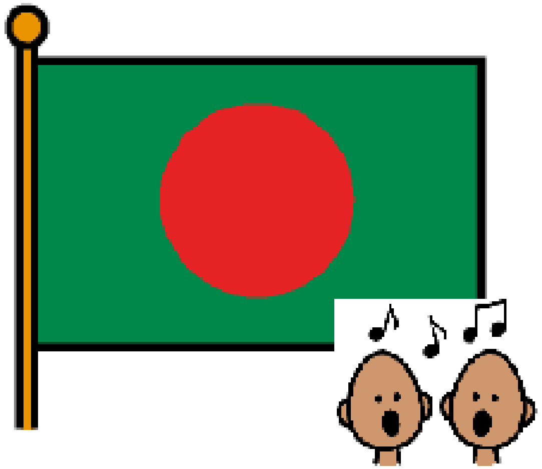

জাতীয় সঙ্গীত

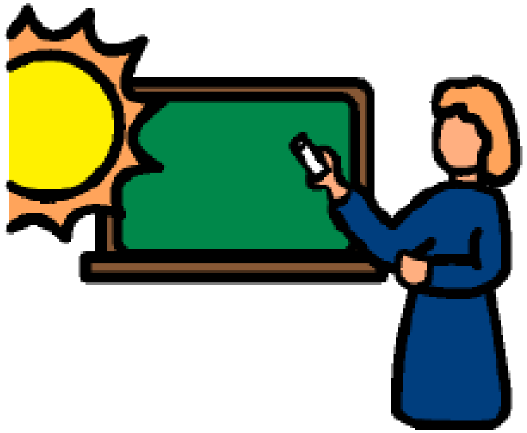

দিন সম্পর্কে জানা

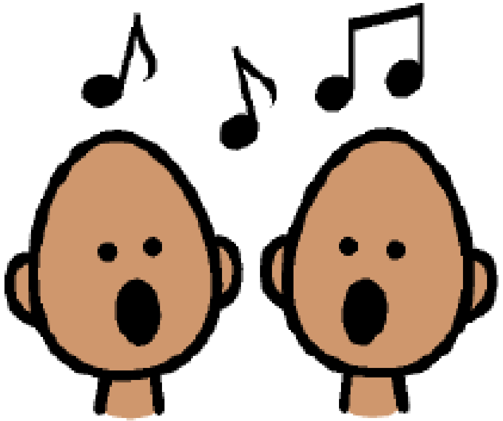

গান করা

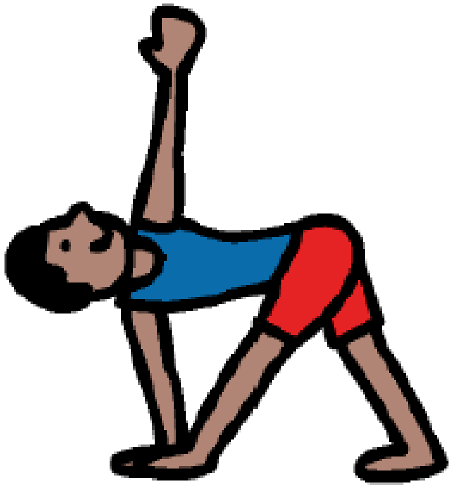

অঙ্গভঙ্গি

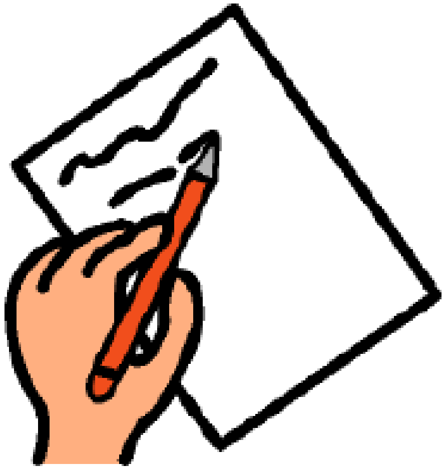

লেখা

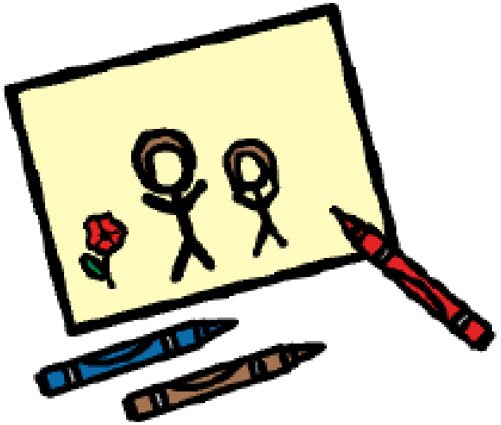

ছবি আঁকা

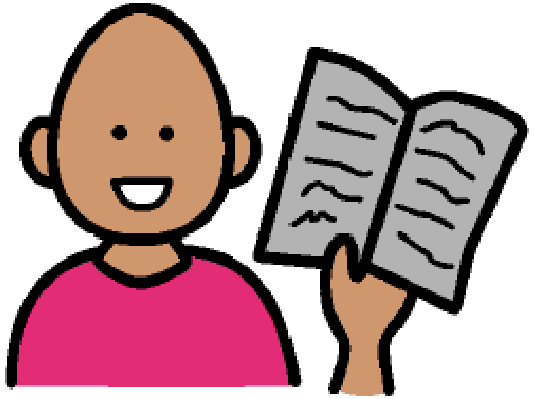

গল্প

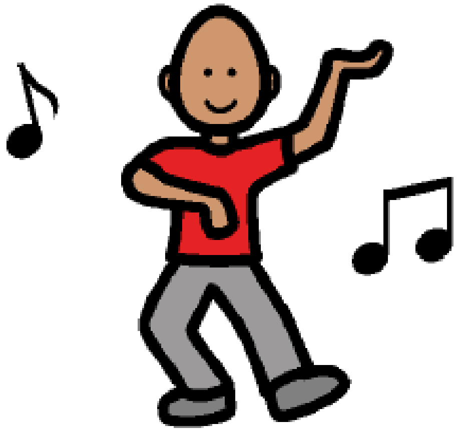

নাচছে

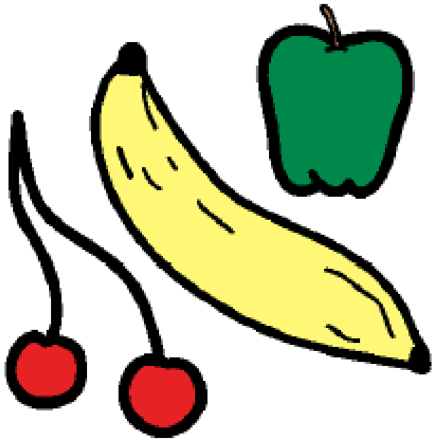

নাস্তা

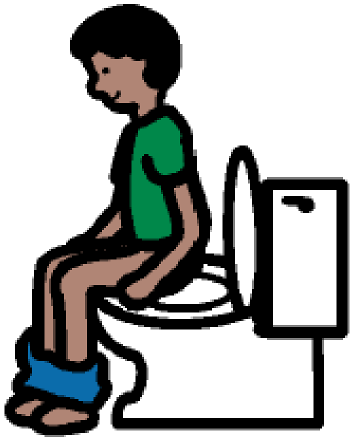

টয়লেট

Orientation Communication Board

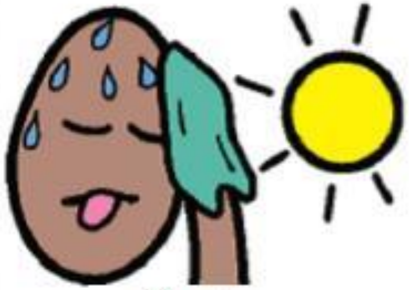
গ্রীষ্মকাল
(গরম)

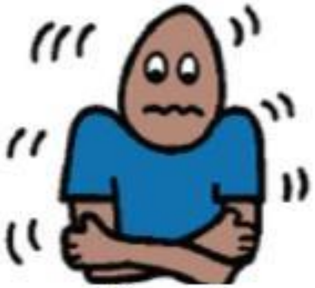
শীতকাল
(ঠান্ডা)

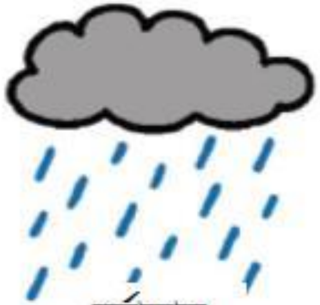
বর্ষাকাল
(বৃষ্টি)

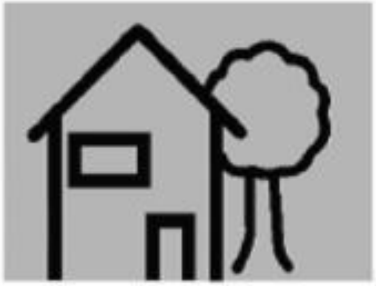
কুয়াশা

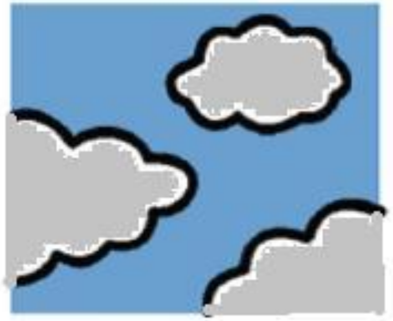
মেঘাচ্ছন্ন

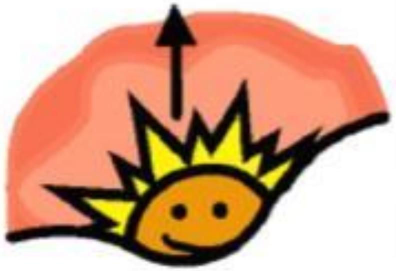
সকাল

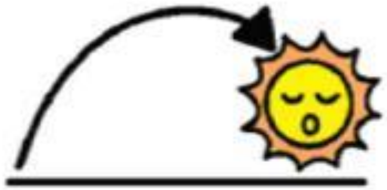
বিকেল

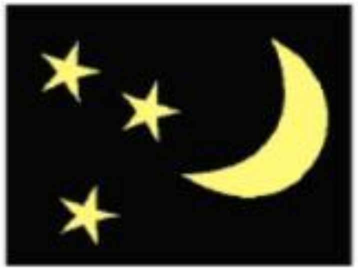
রাত

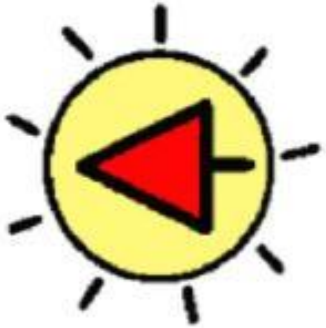
গতকাল

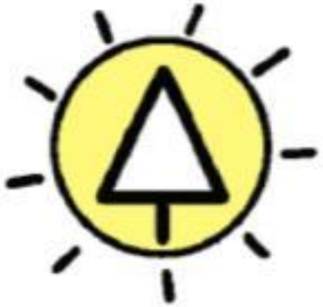
আজ

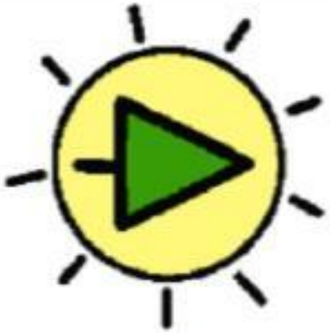
আগামীকা

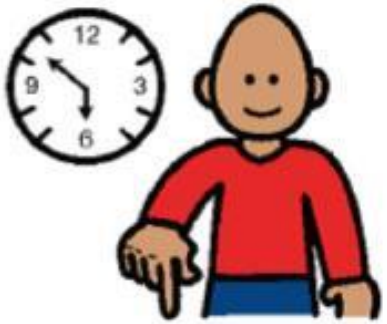

এখন

“I feel” Picture Cards

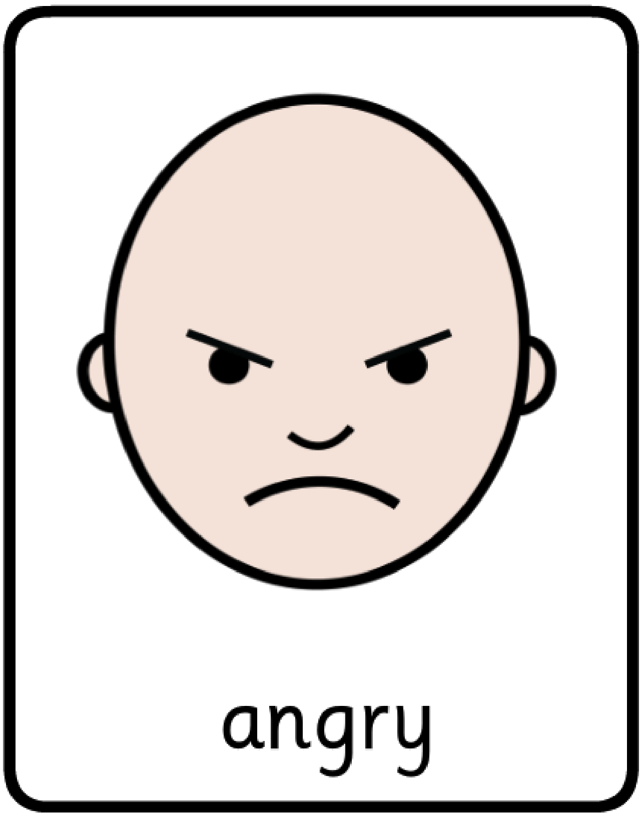

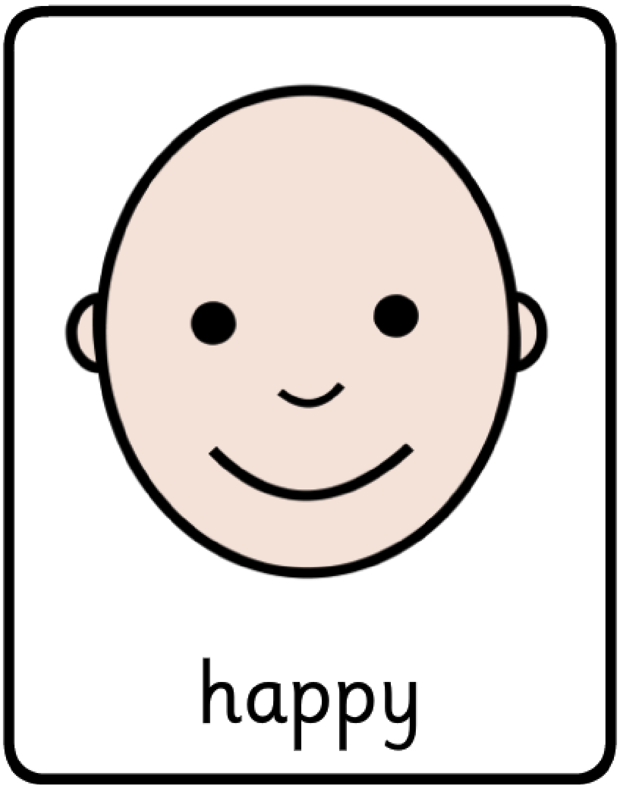

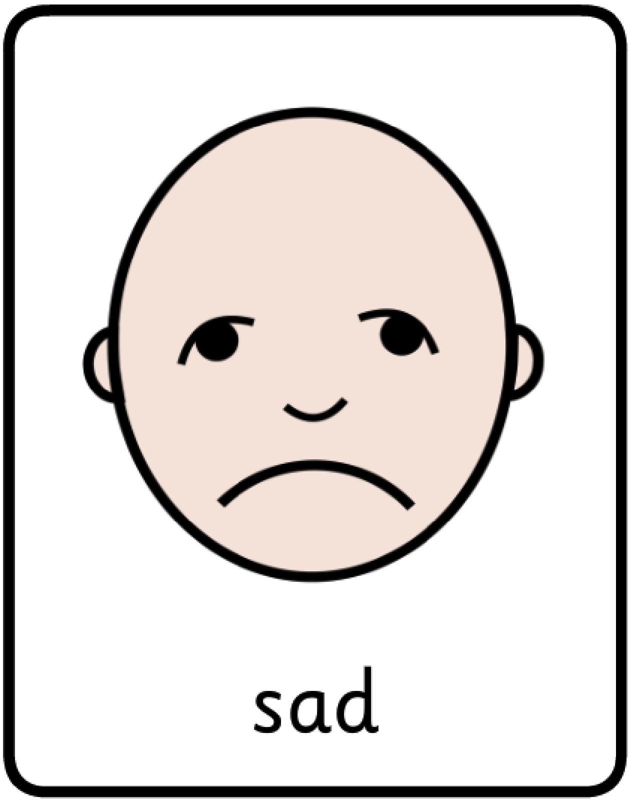

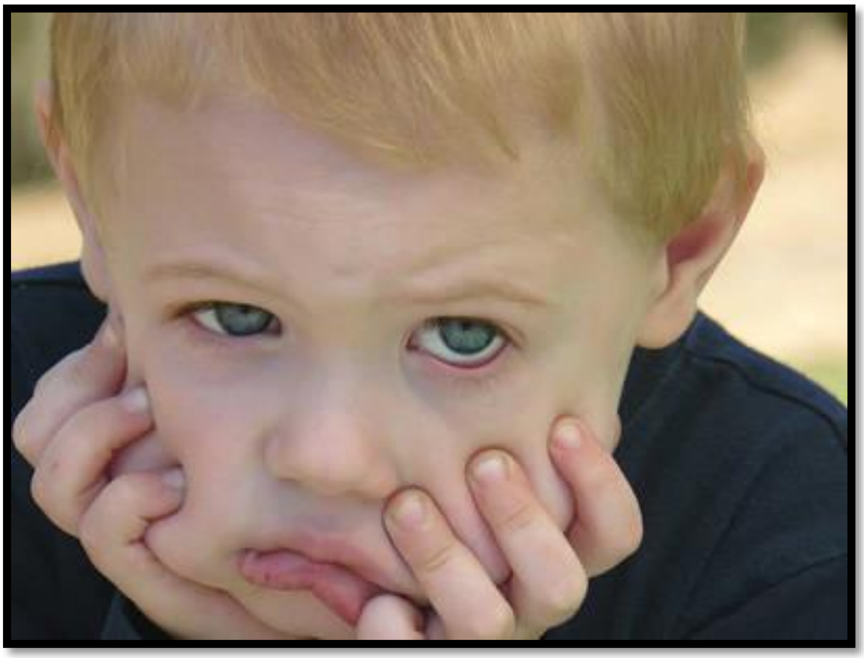

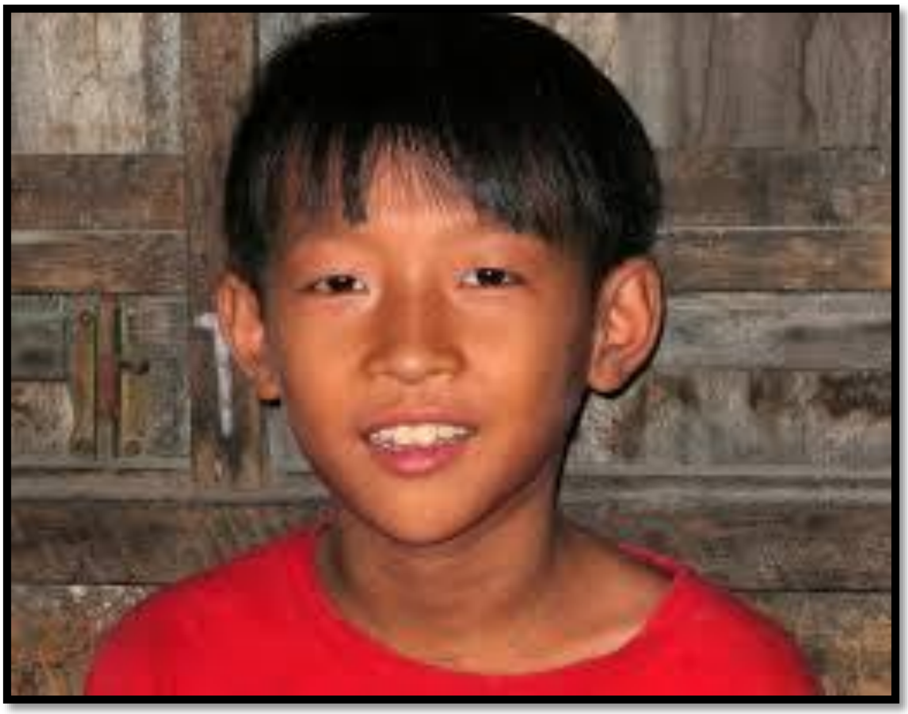

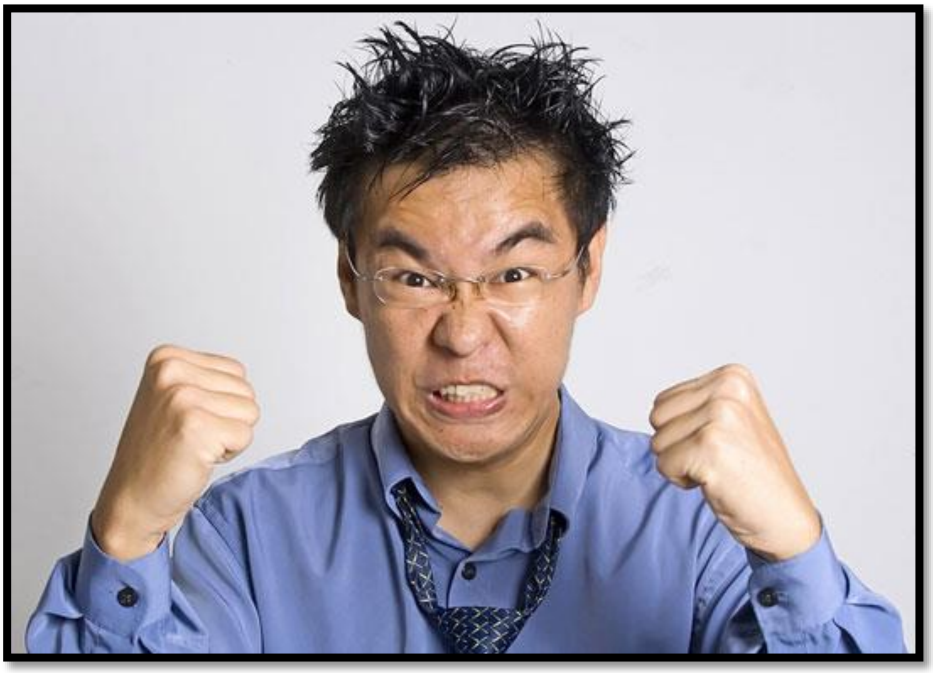

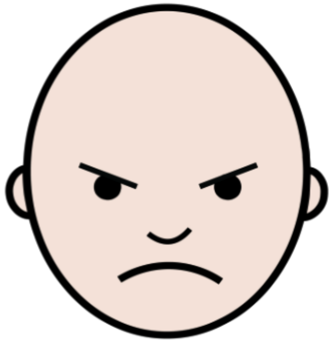

angry

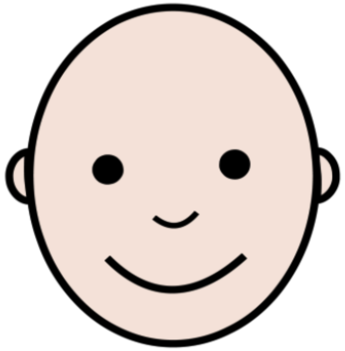

happy

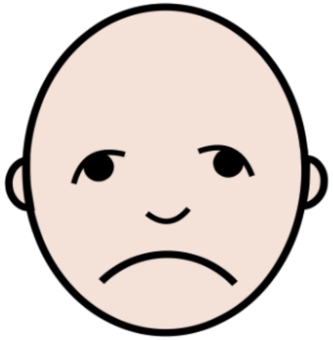

sad

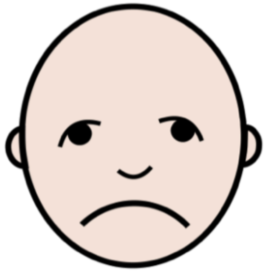

sad

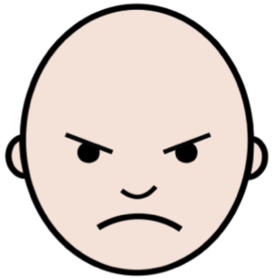

angry

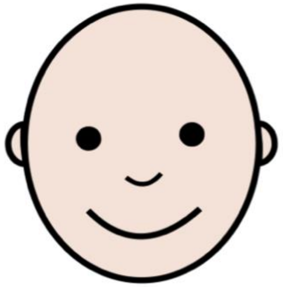

happy

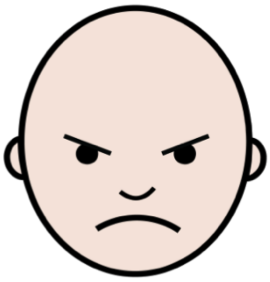

angry

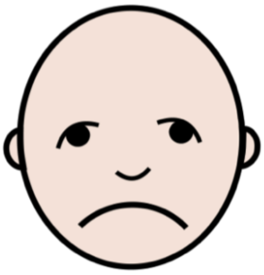

sad

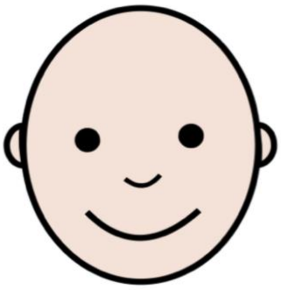

happy

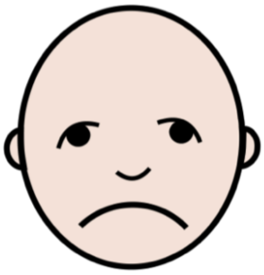

sad

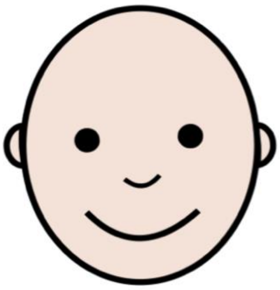

happy

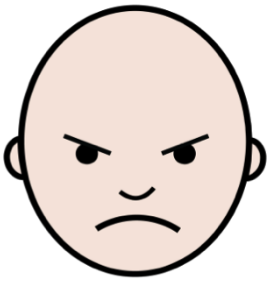

angry

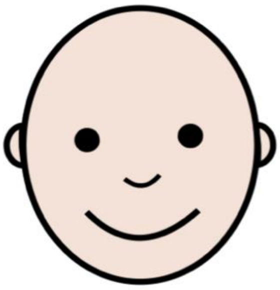

happy

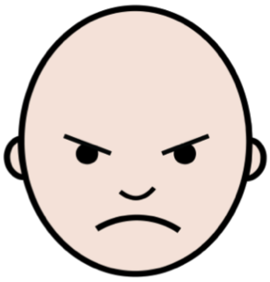

angry

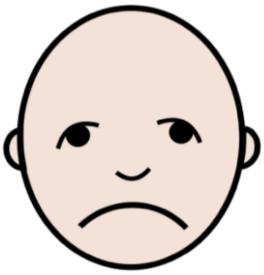

sad

Feelings Symbols

afraid

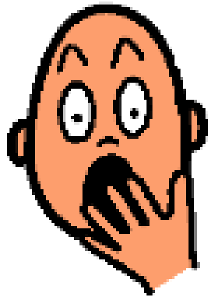

afraid

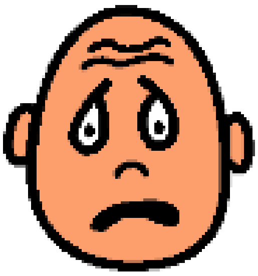

bored

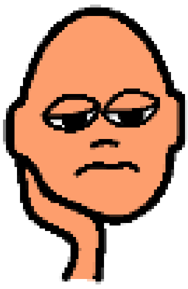

cold

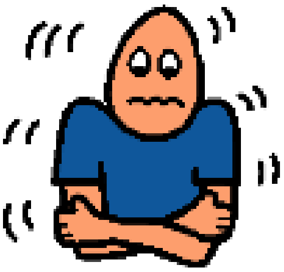

confused

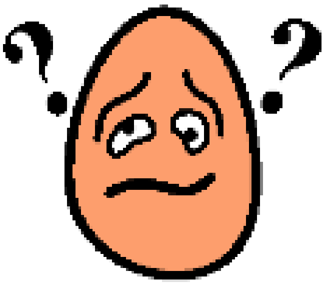

disappointed

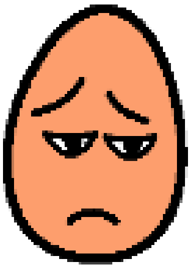

dizzy

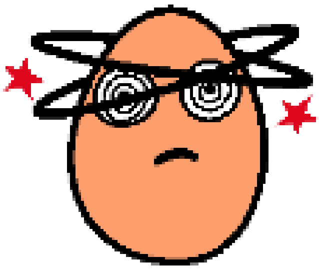

embarrassed

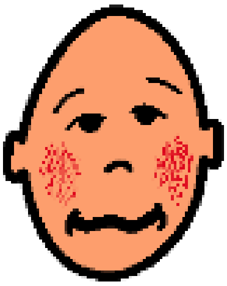

excited

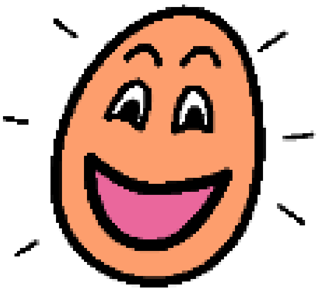

frustrated

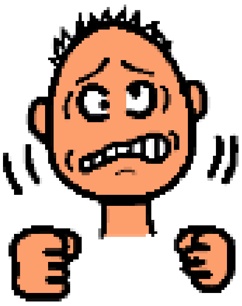

happy

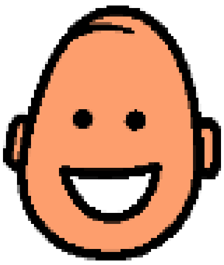

hot

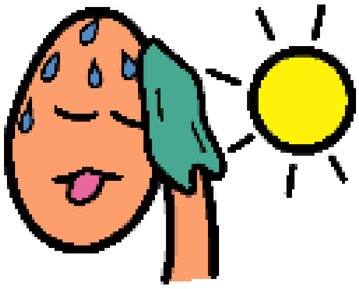

Feelings Symbols

hungry

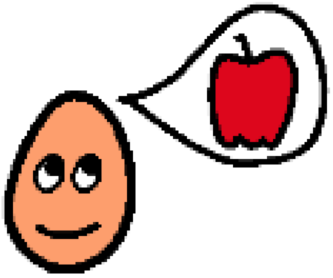

lonely

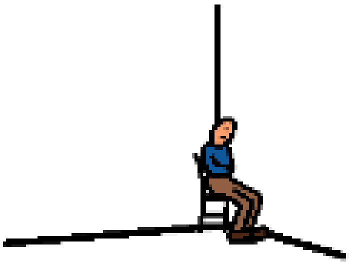

mad

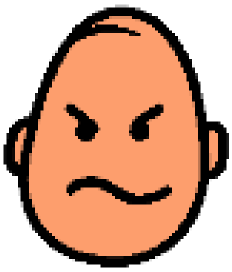

proud

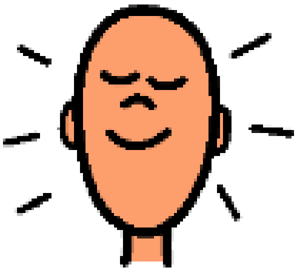

sad

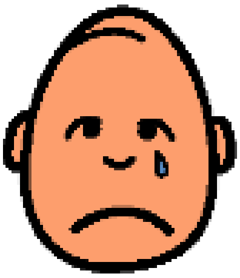

sick

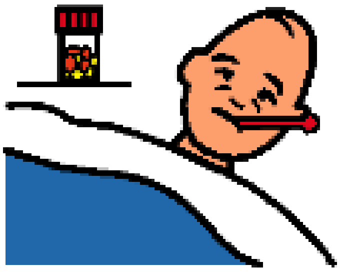

sick

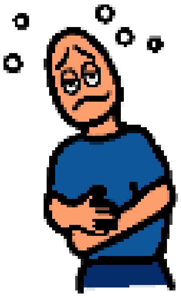

surprised

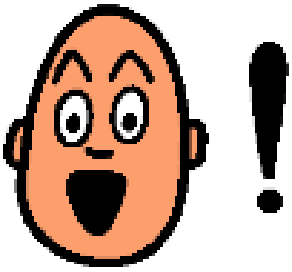

thirsty

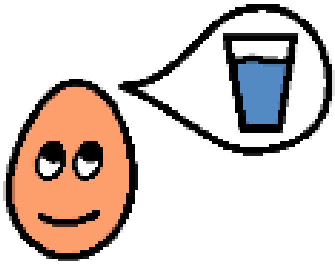

tired

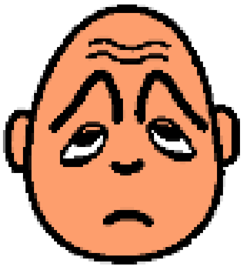

worried

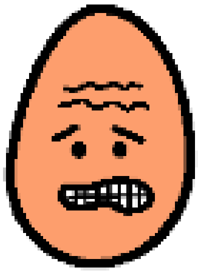

feelings

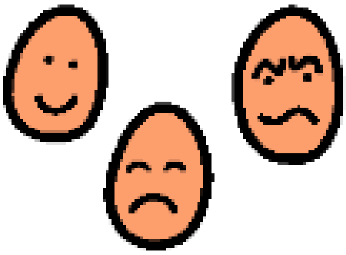

Actions Symbols

eat

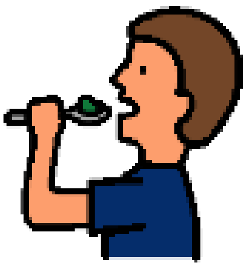

drink

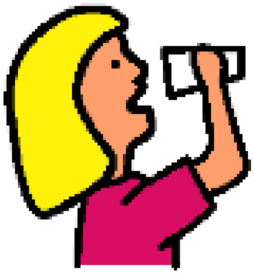

sleep

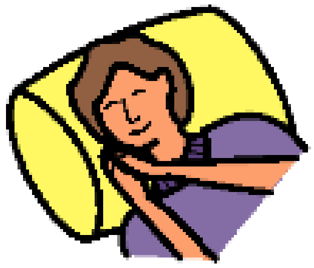

walk

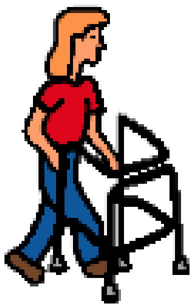

walk

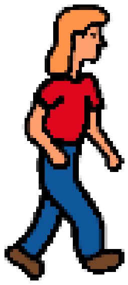

push

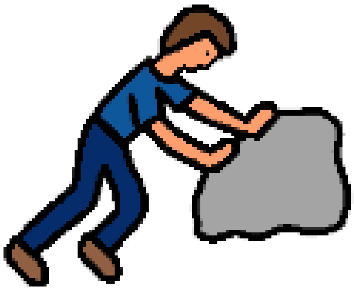

pull

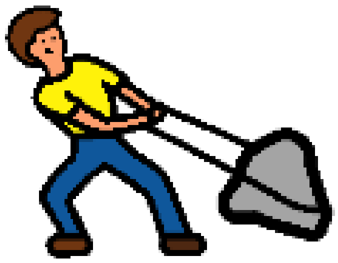

crawl

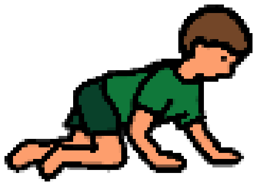

roll

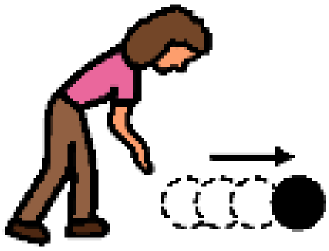

catch

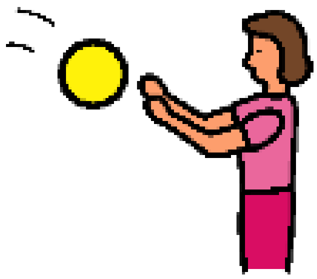

throw

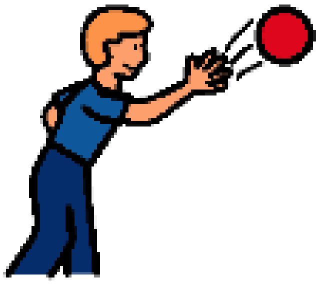

blow

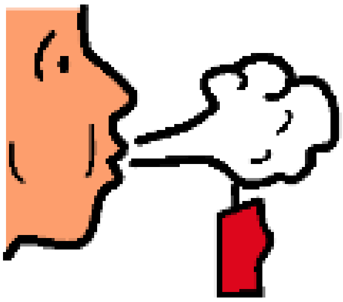

ABC Alphabet and Number Board

YES	1	2	3	4	5	6	7	8	9	0	NO
It starts with...	A	B	C	D	E	F	G	H	I	J	I'm not sure.
New word	K	L	M	N	O	P	Q	R	S		I made a mistake I'll start again
I'd like to ask you something	T	U	V	W	X	Y	Z	?			Thank you

Example Communication Boards

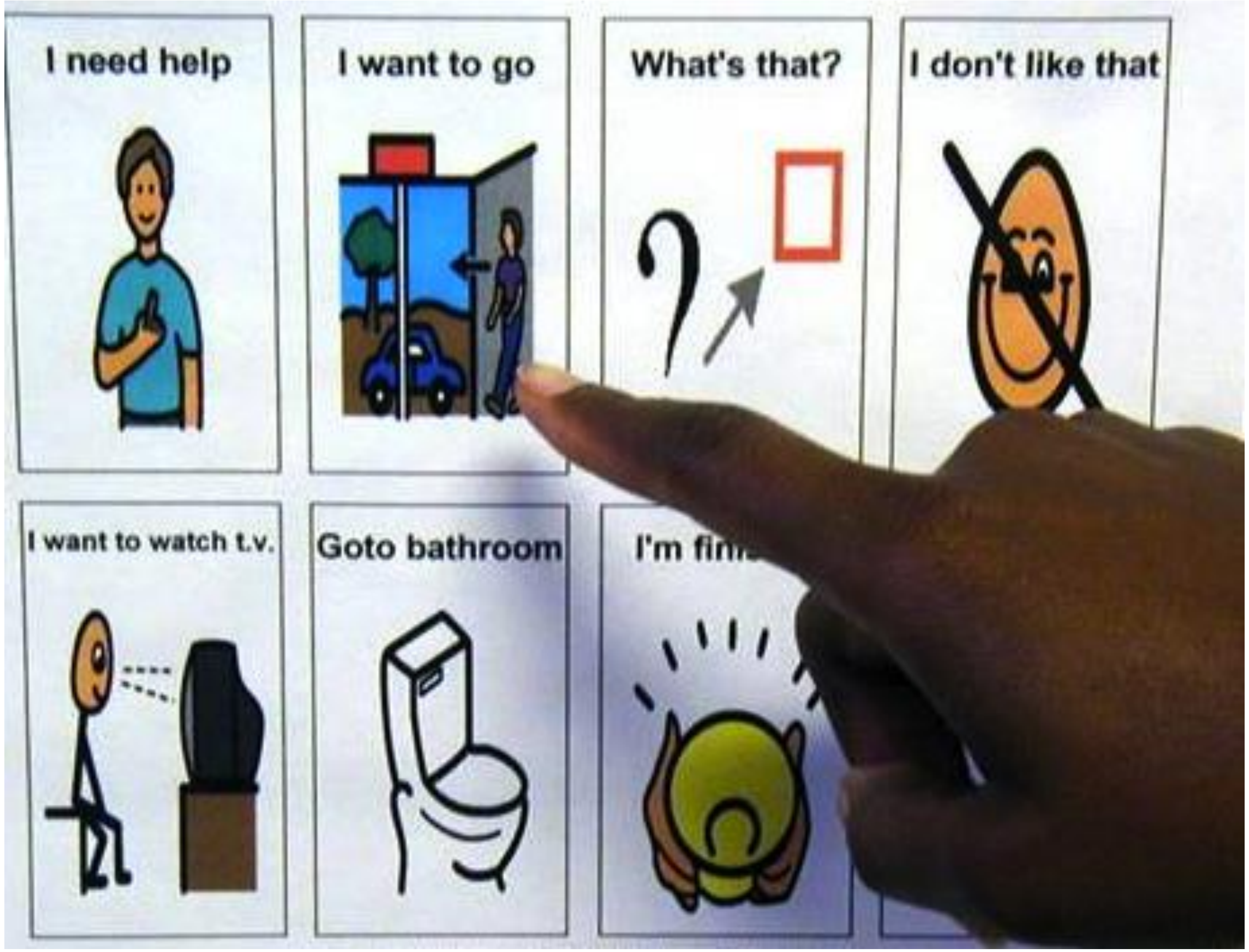

Priya’s Communication Board for Basic Needs

(This is an example)

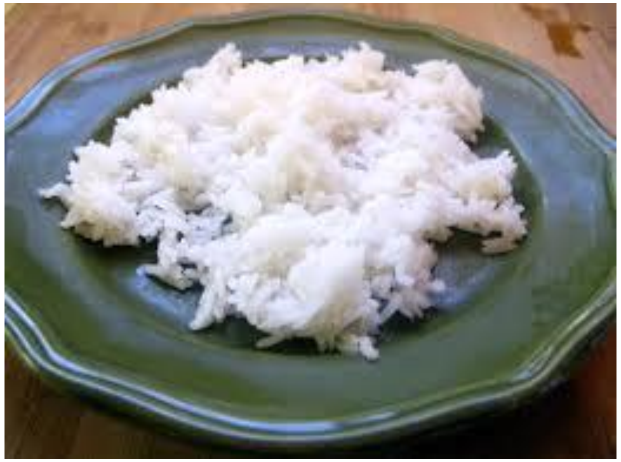 **Eat food**	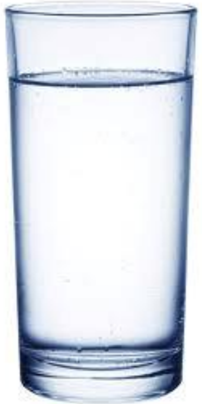 **Drink Water**
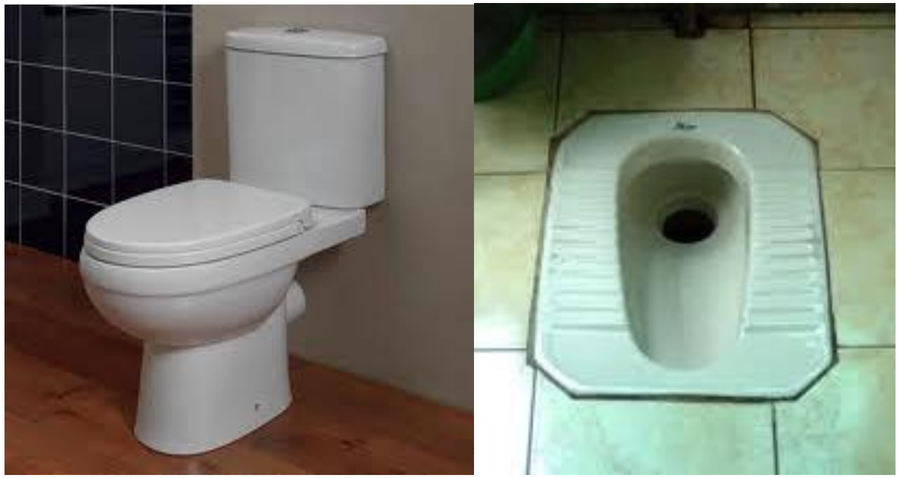 **Toilet Time**	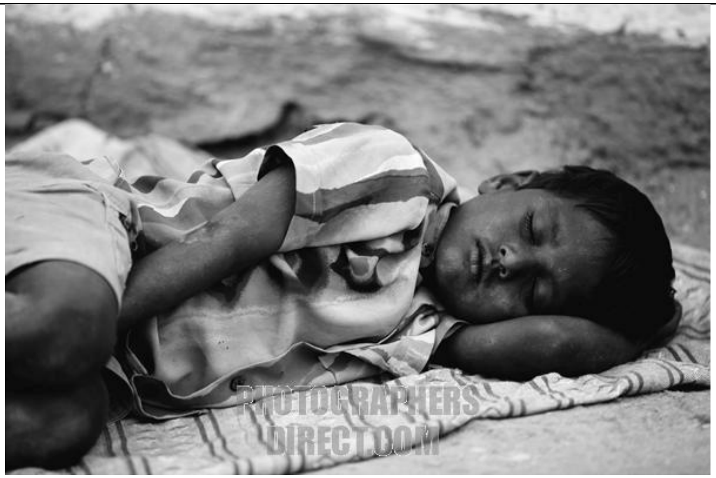 **Sleeping Time**

Priya’s Communication Board for Play Choices

(This is an example)

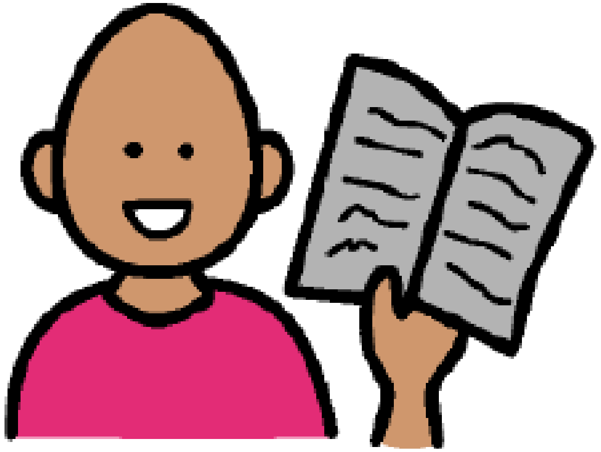

Read a Story

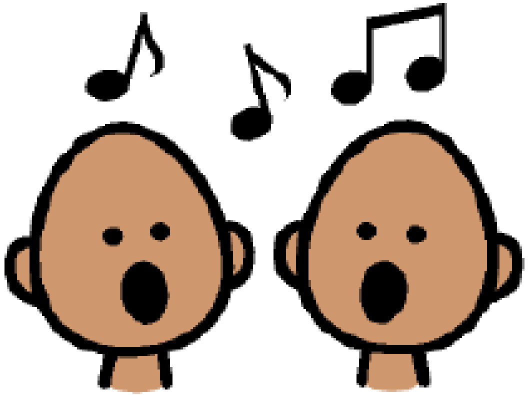

Sing a Song

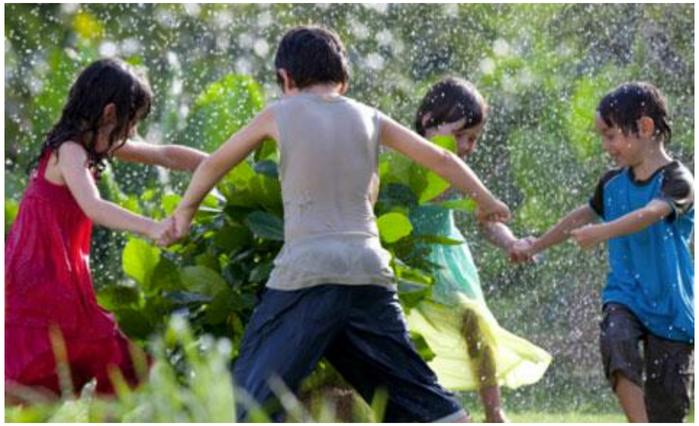

Play outside

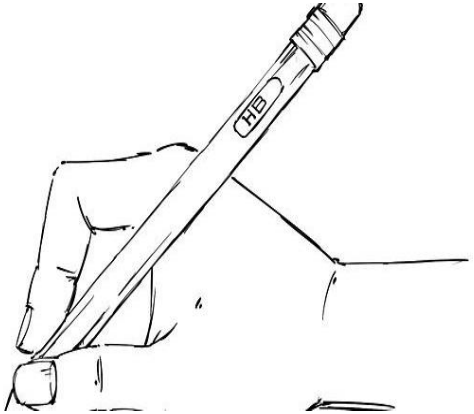

Draw/Write

Communication Board for Colour Choices

(This is an example)

Red	Yellow	Pink	Green	White
Purple	Orange	Blue	Brown	Black

Communication Board for More and Finished

(This is an example)

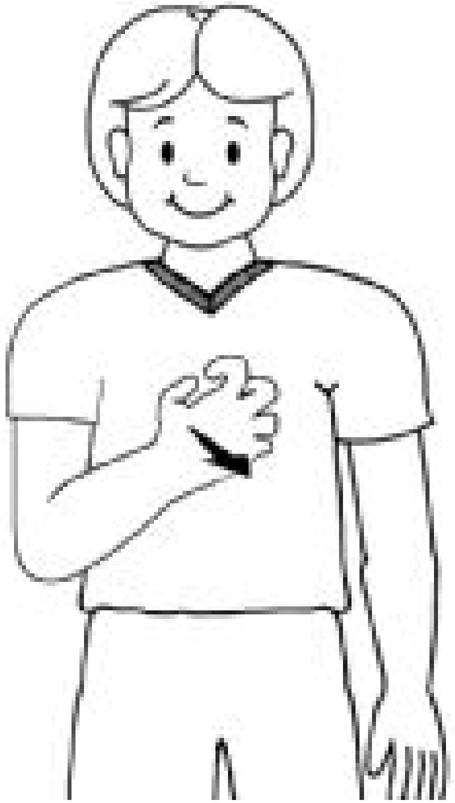 **More**	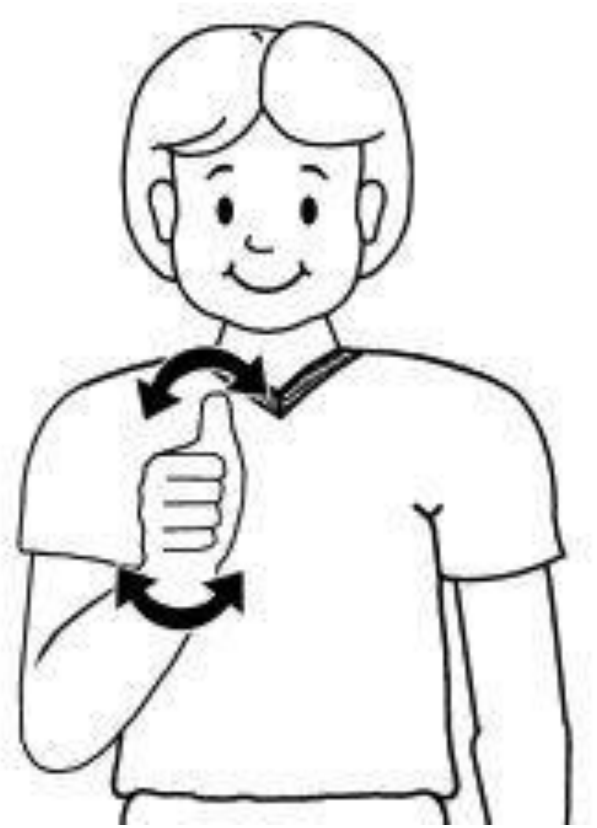 **Finished**

Visual Timetables

Getting ready for school 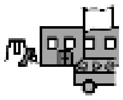

✓ Tick when completed

1	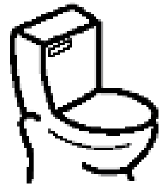 Go to the toilet.	
2	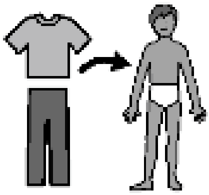 Get dressed.	
3	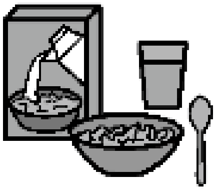 Have breakfast.	
4	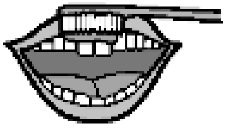 Brush teeth.	
5	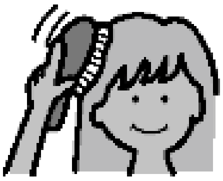 Brush hair.	
6	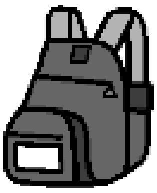 Pack bag.	

Getting ready for bed 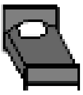

✓ Tick when completed

1	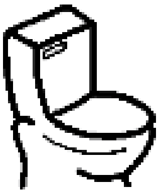 Go to the toilet.	
2	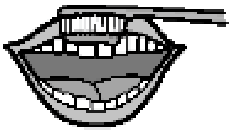 Brush teeth.	
3	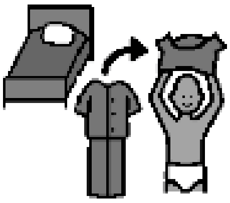 Put on pyjamas.	
4	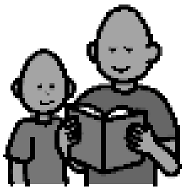 Read a book.	
5	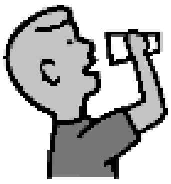 Have a drink.	
6	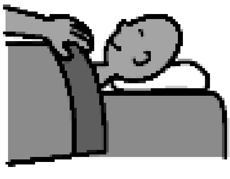 Go to bed/sleep.	
